# Decoding the genetic symphony: Profiling protein-coding and long noncoding RNA expression in T-acute lymphoblastic leukemia for clinical insights

**DOI:** 10.1093/pnasnexus/pgae011

**Published:** 2024-01-12

**Authors:** Deepak Verma, Shruti Kapoor, Sarita Kumari, Disha Sharma, Jay Singh, Mercilena Benjamin, Sameer Bakhshi, Rachna Seth, Baibaswata Nayak, Atul Sharma, Raja Pramanik, Jayanth Kumar Palanichamy, Sridhar Sivasubbu, Vinod Scaria, Mohit Arora, Rajive Kumar, Anita Chopra

**Affiliations:** Laboratory Oncology, Dr BRAIRCH, All India Institute of Medical Sciences, New Delhi-110029, India; CSIR-Institute of Genomics and Integrative Biology, New Delhi-110025, India; Laboratory Oncology, Dr BRAIRCH, All India Institute of Medical Sciences, New Delhi-110029, India; CSIR-Institute of Genomics and Integrative Biology, New Delhi-110025, India; Laboratory Oncology, Dr BRAIRCH, All India Institute of Medical Sciences, New Delhi-110029, India; Laboratory Oncology, Dr BRAIRCH, All India Institute of Medical Sciences, New Delhi-110029, India; Department of Medical Oncology, Dr BRAIRCH, All India Institute of Medical Sciences, New Delhi-110029, India; Department of Pediatrics, All India Institute of Medical Sciences, New Delhi-110029, India; Department of Gastroenterology, All India Institute of Medical Science, New Delhi-110029, India; Department of Medical Oncology, Dr BRAIRCH, All India Institute of Medical Sciences, New Delhi-110029, India; Department of Medical Oncology, Dr BRAIRCH, All India Institute of Medical Sciences, New Delhi-110029, India; Department of Biochemistry, All India Institute of Medical Sciences, New Delhi-110029, India; CSIR-Institute of Genomics and Integrative Biology, New Delhi-110025, India; CSIR-Institute of Genomics and Integrative Biology, New Delhi-110025, India; Department of Biochemistry, All India Institute of Medical Sciences, New Delhi-110029, India; Laboratory Oncology, Dr BRAIRCH, All India Institute of Medical Sciences, New Delhi-110029, India; Laboratory Oncology, Dr BRAIRCH, All India Institute of Medical Sciences, New Delhi-110029, India

**Keywords:** leukemia, T-ALL, ETP-ALL, gene expression, immunophenotype, transcriptomics

## Abstract

T-acute lymphoblastic leukemia (T-ALL) is a heterogeneous malignancy characterized by the abnormal proliferation of immature T-cell precursors. Despite advances in immunophenotypic classification, understanding the molecular landscape and its impact on patient prognosis remains challenging. In this study, we conducted comprehensive RNA sequencing in a cohort of 35 patients with T-ALL to unravel the intricate transcriptomic profile. Subsequently, we validated the prognostic relevance of 23 targets, encompassing (i) protein-coding genes—*BAALC*, *HHEX*, *MEF2C*, *FAT1*, *LYL1*, *LMO2*, *LYN*, and *TAL1*; (ii) epigenetic modifiers—*DOT1L*, *EP300*, *EML4*, *RAG1*, *EZH2*, and *KDM6A*; and (iii) long noncoding RNAs (lncRNAs)—*XIST*, *PCAT18*, *PCAT14*, *LINC00202*, *LINC00461*, *LINC00648*, *ST20*, *MEF2C-AS1*, and *MALAT1* in an independent cohort of 99 patients with T-ALL. Principal component analysis revealed distinct clusters aligning with immunophenotypic subtypes, providing insights into the molecular heterogeneity of T-ALL. The identified signature genes exhibited associations with clinicopathologic features. Survival analysis uncovered several independent predictors of patient outcomes. Higher expression of *MEF2C*, *BAALC*, *HHEX*, and *LYL1* genes emerged as robust indicators of poor overall survival (OS), event-free survival (EFS), and relapse-free survival (RFS). Higher *LMO2* expression was correlated with adverse EFS and RFS outcomes. Intriguingly, increased expression of lncRNA *ST20* coupled with *RAG1* demonstrated a favorable prognostic impact on OS, EFS, and RFS. Conclusively, several hitherto unreported associations of gene expression patterns with clinicopathologic features and prognosis were identified, which may help understand T-ALL's molecular pathogenesis and provide prognostic markers.

**Significance Statement**

This T-acute lymphoblastic leukemia (T-ALL) transcriptomics study holds substantial significance in advancing our understanding of the molecular landscape and clinical implications of this heterogeneous malignancy. Despite strides in immunophenotypic classification, unraveling the intricate transcriptomic profile of T-ALL remains challenging. This study, through comprehensive RNA sequencing in a cohort of 35 patients with T-ALL, not only addresses this gap but also goes on to validate the prognostic relevance of a curated set of 23 targets, including protein-coding genes, epigenetic modifiers, and long noncoding RNAs. The identified signature genes exhibit meaningful associations with clinicopathologic features, and survival analysis uncovers several independent predictors of patient outcomes. These findings contribute significantly to the clinical management of T-ALL, offering potential prognostic markers that can guide risk stratification and therapeutic decision-making.

## Introduction

T-lineage acute lymphoblastic leukemia (T-ALL) represents a formidable challenge in the field of oncology, with its unique genetic and clinical features. It is characterized by the malignant transformation of immature T-cell precursors. It accounts for 20–25% of adults and 10–15% of pediatric ALL cases in Europe, the United States, and Japan (1, 2). It has been reported to be more prevalent in developing countries than in developed countries, possibly due to genetic and environmental factors that are yet unidentified (3). It is more prevalent in males than in females (1). Although the prognosis of T-ALL has improved considerably over the years, the outcomes remain inferior to those of B-lineage ALL, particularly in relapsed and refractory settings (4).

Understanding the molecular intricacies that drive T-ALL is paramount for advancing our knowledge and improving patient outcomes. The emergence of high-throughput techniques has provided immense insights into the genomic organization of functional elements in the human genome. This, combined with cell biology techniques applied to T-ALL, has led to significant advances in our understanding of the disease and has allowed the development of novel therapeutic approaches (5). The malignant transformation that culminates in T-ALL is a multistep process in which genetic alterations occurring in crucial cellular pathways work together to produce the T-ALL phenotype. Activating mutations in the *NOTCH1* gene, mutations in *FBXW7* tumor suppressor, and loss of *CDKN2A* locus frequently occur in T-ALL (6–9), which also impact patient survival (9, 10). Besides genetic mutations, gene expression profiling in patients with T-ALL has revealed aberrant expression of a diverse group of transcription factors such as *LYL1*, *LMO1*, *LMO2*, *TAL1*, *TLX1*, *TLX3*, *HOXA*, *NKX2.1*, *NKX2.2*, *NKX2.5*, *MYC*, *MYB*, and *SPI1* in distinct T-ALL subtypes (7, 11). Several common genetic defects have also been observed among distinct genetic subgroups, which commonly involve oncogenic signaling cascades, including IL7R/JAK/STAT, PI3K/AKT, and RAS/MEK/ERK signaling (1, 12). Some less understood facets in T-ALL include epigenetic deregulation, ribosomal dysfunction, and altered expression of oncogenic miRNAs or long noncoding RNA (lncRNA).

Although a better understanding of molecular pathophysiology and immunophenotyping led to the refinement of the classification of T-ALL, it could not translate into their application in the management of patients as the clinical relevance of these subtypes remains either unclear or controversial (1, 7, 13, 14). None of the currently known genetic markers is used for risk stratification of patients with T-ALL. The only subtype of T-ALL that has a place in the 2022 revision of WHO is early thymic precursor ALL (ETP-ALL) (13, 15, 16). This study delves into the intricate landscape of protein-coding and lncRNA expression in T-ALL. By dissecting the molecular signatures of T-ALL at the RNA level, we aim to shed light on the underlying mechanisms that drive this disease. Furthermore, we seek to elucidate the clinical relevance of these targets in T-ALL diagnosis, prognosis, and treatment response, with the ultimate goal of paving the way for more precise and effective therapeutic interventions.

## Results

**Fig. 1.**
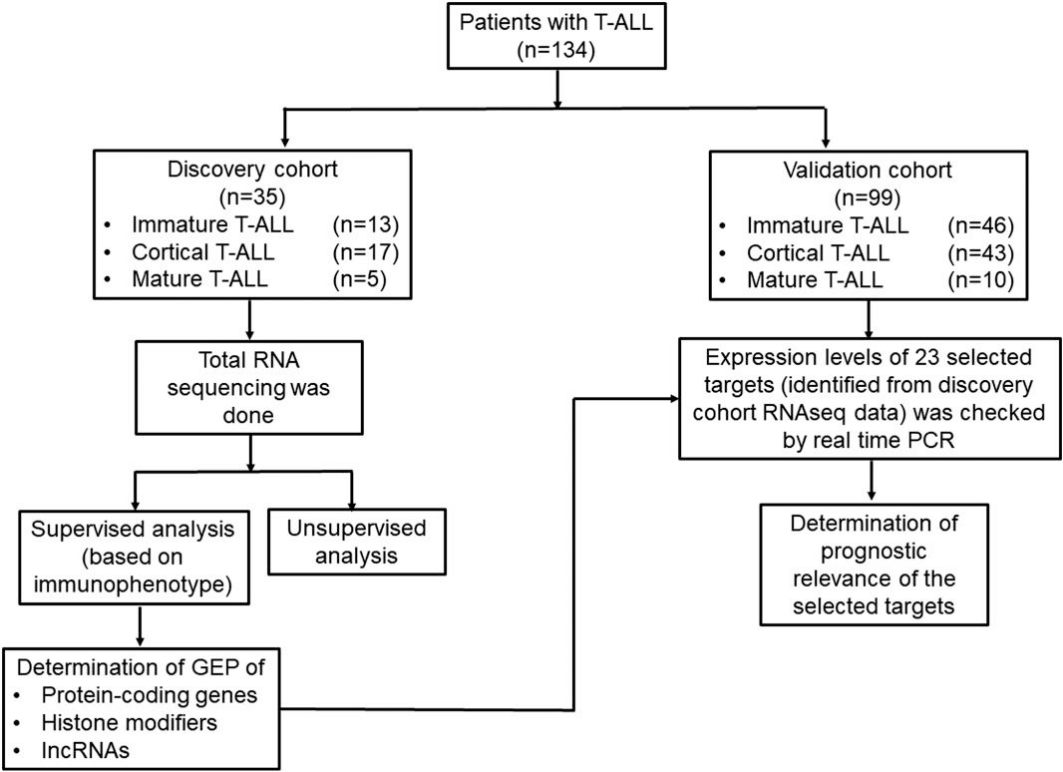
Workplan of the study. One hundred and thirty-four patients with T-ALL were recruited in the study. The patients were divided into two cohorts: discovery cohort (*n* = 35) and validation cohort (*n* = 99). Total RNA sequencing was done in the discovery cohort. RNAseq data were analyzed using both supervised and unsupervised approaches. The supervised analysis was based on immunophenotype (immature, cortical, and mature) of the patients at the diagnosis. The gene expression profiles (GEP) of protein-coding genes, histone modifiers, and lncRNAs for the three subtypes of T-ALL were determined. The clinical significance of the 23 selected targets (identified from the RNAseq data of the discovery cohort) was tested in the validation cohort.

This study included 134 subjects with de novo T-ALL. The patients were immunophenotypically classified into immature (pro-T-ALL and pre-T-ALL), cortical, and mature T-ALL based on the European Group for the Immunological Characterization of Leukemias criteria (17, 18). ETP-ALL was recognized based on previously defined criteria (cCD3 positive, CD1a negative, CD5dim/negative; lack of expression of both CD4 and CD8; and positivity of stem cell and/or myeloid markers [HLA-DR, CD13, CD33, CD34, or CD117]) (16, 18, 19). The patients were divided into two cohorts: discovery cohort (*n* = 35) and validation cohort (*n* = 99). Total RNA sequencing was performed in the discovery cohort. Further, the clinical and prognostic relevance of 23 selected targets identified in the discovery cohort was checked in the validation cohort (Fig. 1).

### Discovery cohort

#### Clinical characteristics of patients in the discovery cohort

In the discovery cohort (*n* = 35), there were 13 immature (including 5 ETP-ALL), 17 cortical, and 5 mature T-ALL cases. The median age of the patients was 10 years (range, 2–40 years). There were 27 males (20 children, 7 adults) and 8 females (6 children, 2 adults). The median hemoglobin was 7.6 g/dL (range, 3.9–14 g/dL), median total leukocyte count (TLC) 134.8 × 10^9^/L (range, 0.7–663 × 10^9^/L), and median platelet counts 42 × 10^9^/L (range, 16–285 × 10^9^/L). The cytogenetics data were available in 11 patients. The cytogenetics was normal in 7/11 (63.33%) patients. Fourteen patients were treated with ICiCLE protocol (20), 3 with the Berlin–Frankfurt–Munster-90 (BFM-90) protocol (21), 1 with INCTR (22), 1 with the Holzer’s protocol (19), and 2 with Hyper CVAD protocol (23). Fourteen patients did not receive treatment/succumbed to the disease before the initiation of treatment. A detailed description of baseline characteristics and outcome of the patients in the discovery cohort is given in the Supplementary Excel file.

#### Analysis of the RNA expression profile in the discovery cohort

We identified the gene expression profile of our patients with T-ALL. We did a supervised analysis based on the immunophenotype of the T-ALL cases to identify differentially expressed RNAs (protein-coding genes, epigenetic modifiers, and lncRNAs) for the three T-ALL subtypes viz. immature, cortical, and mature T-ALL. In addition, we used an unsupervised approach to classify our cases using principal component analysis (PCA) on BioVinci Version: 1.1.5, r20181005 (BioTuring, USA; https://vinci.bioturing.com/feature).

##### Supervised approach

###### Determination of the expression profile of protein-coding genes

**Fig. 2.**
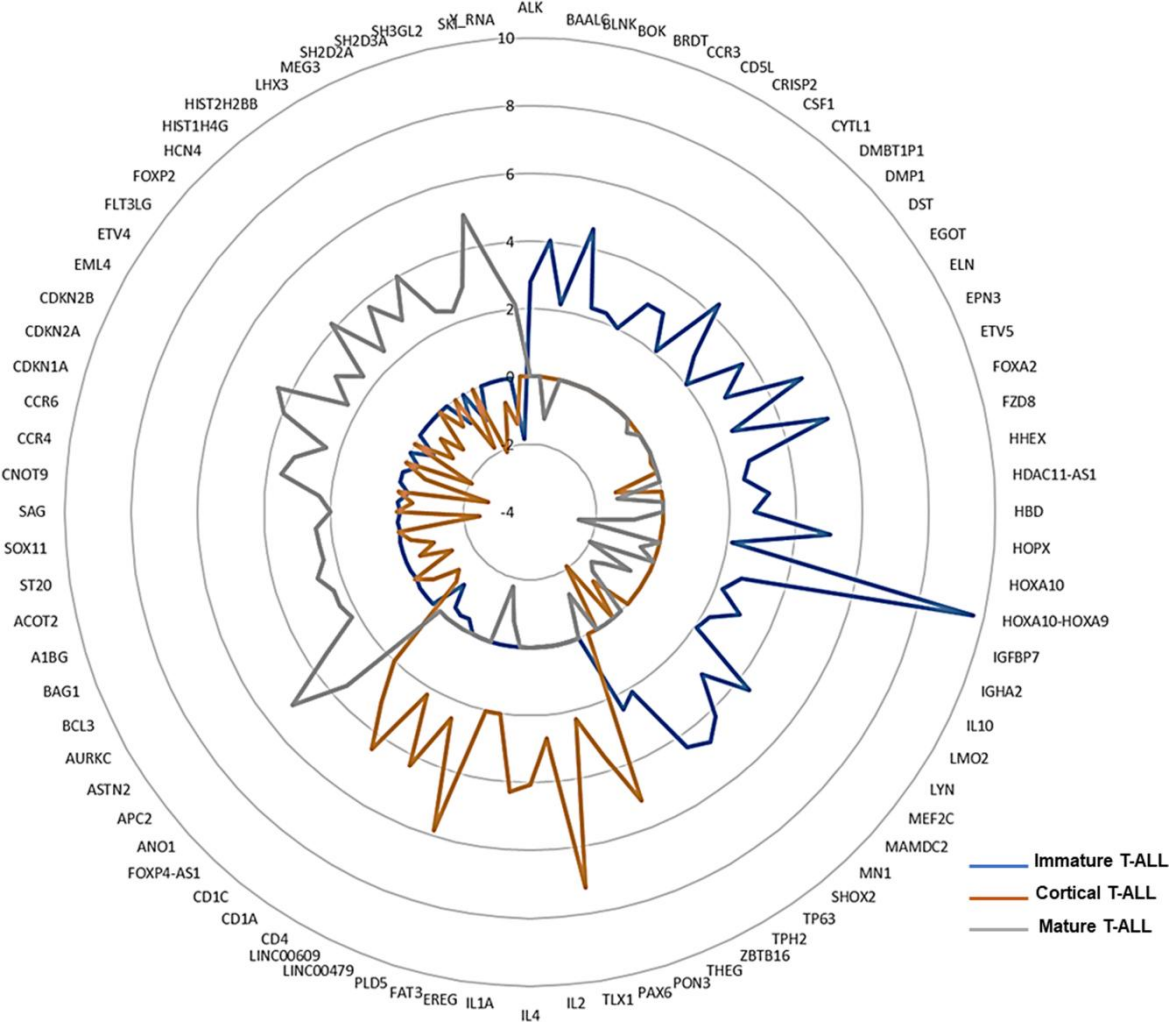
Radar plot displaying fold changes of selected differentially expressed protein-coding genes in T-ALL subtypes—immature, cortical, and mature. The three subgroups are represented in different colors. Each circle represents the log_2_-fold change of expression for the differentially expressed genes.

We found 2,318 differentially expressed protein-coding genes in the three subtypes of T-ALL (Fig. 2, Tables S1–S3, and Fig. S1A). In immature T-ALL cases, transcription factors controlling early hematopoiesis, including *MEF2C*, *TP63*, *HHEX*, *RUNX2*, *HOXA10*, *HOXA9*, *RUNX1T1*, and *ZBTB16*; homeobox genes including *HOPX, LYL1*, *LMO2*, and *LYN* (a tyrosine kinase–coding gene) were highly expressed. *BAALC* and *MN1* genes, previously reported in AML (24), were also overexpressed in patients with immature T-ALL. *BAALC*-associated genes *IGFBP7* and *PROM1* (CD133), known to confer chemoresistance, were also overexpressed (25). In addition, *MAML3*, *NT5E* (CD73), and *ARID5B* exhibited significant overexpression in immature T-ALL. Moreover, genes not previously described in T-ALL, like *PLD4* and *TP63*, were also overexpressed. As expected, the cortical thymocytes—defining *CD1A* gene was overexpressed in cortical T-ALL. Homeobox domain genes *NKX2-1*, *TLX1*, and *TLX3* known to be involved in T-cell development were up-regulated. In addition, *FAT1*, *FAT3*, *RAG1*, *EREG*, *CD1C*, *AKAP-2*, *IL-4*, *PRTG*, *TCL-6*, *ZP1*, and *TRAV* genes were overexpressed. In mature T-ALL, *APC2*, *BCL3*, *CCR4*, *CDKN2A*, *HIST1H4G*, *HIST2H2B*, *NCOR2*, and *TRAV22* genes were overexpressed (Fig. 2). *TAL1* overexpression was seen in T-ALL cases with cortical and mature immunophenotype.

###### Determination of expression profile of epigenetic modifiers

We also investigated the transcriptomic profile of various epigenetic-modifying genes. Our analysis revealed overexpression of histone methyltransferases like *SETD2*, *ASH1L*, and *SUZ12*, along with overexpression of histone demethylase, *KDM6a*, and transcription regulators like *ATM*, and *PHF6* in all subtypes of T-ALL. Histone deacetylase *HDAC4* was also found to be overexpressed in all the subtypes, pointing toward increased methylation and repression of the target genes in T-ALL. On comparative analysis among three subtypes, *HDAC9* and *SMYD3* were found to be up-regulated, while *EZH2* was down-regulated in immature T-ALL subtype. *HDAC10* was underexpressed in cortical T-ALL, and *EP300*, *PKN1*, *EML4*, and *DOT1L* were overexpressed in the mature subtype of T-ALL. *HDAC7* was underexpressed in immature and cortical T-ALL (Fig. S1B and Table S4).

###### Determination of expression profile of lncRNAs

Two thousand two hundred and forty-three lncRNAs were found to be differentially expressed, out of which 223 lncRNAs were filtered based on >2 FPKM score (Fragments Per Kilobase of transcript per Million mapped reads). Unique lncRNA signatures were found to be associated with each subtype of T-ALL. Previously reported lncRNAs in T-ALL, like *XIST*, were expressed in immature T-ALL (26), while *LUNAR1*, known as specific *NOTCH1*-regulated lncRNA, was expressed in cortical and mature T-ALL (27). In addition, we observed an overexpression of *HOTTIP* and *MEF2C-AS1* in immature T-ALL, *LINC01221*, *LINC00202*, *LINC00461*, and *LINC00648* in cortical T-ALL and *MALAT1*, *ST20*, and *PCAT14* in mature T-ALL. *PCAT18* was overexpressed in all subtypes of T-ALL (Fig. S1C and Table S5).

##### Principal component analysis

**Fig. 3.**
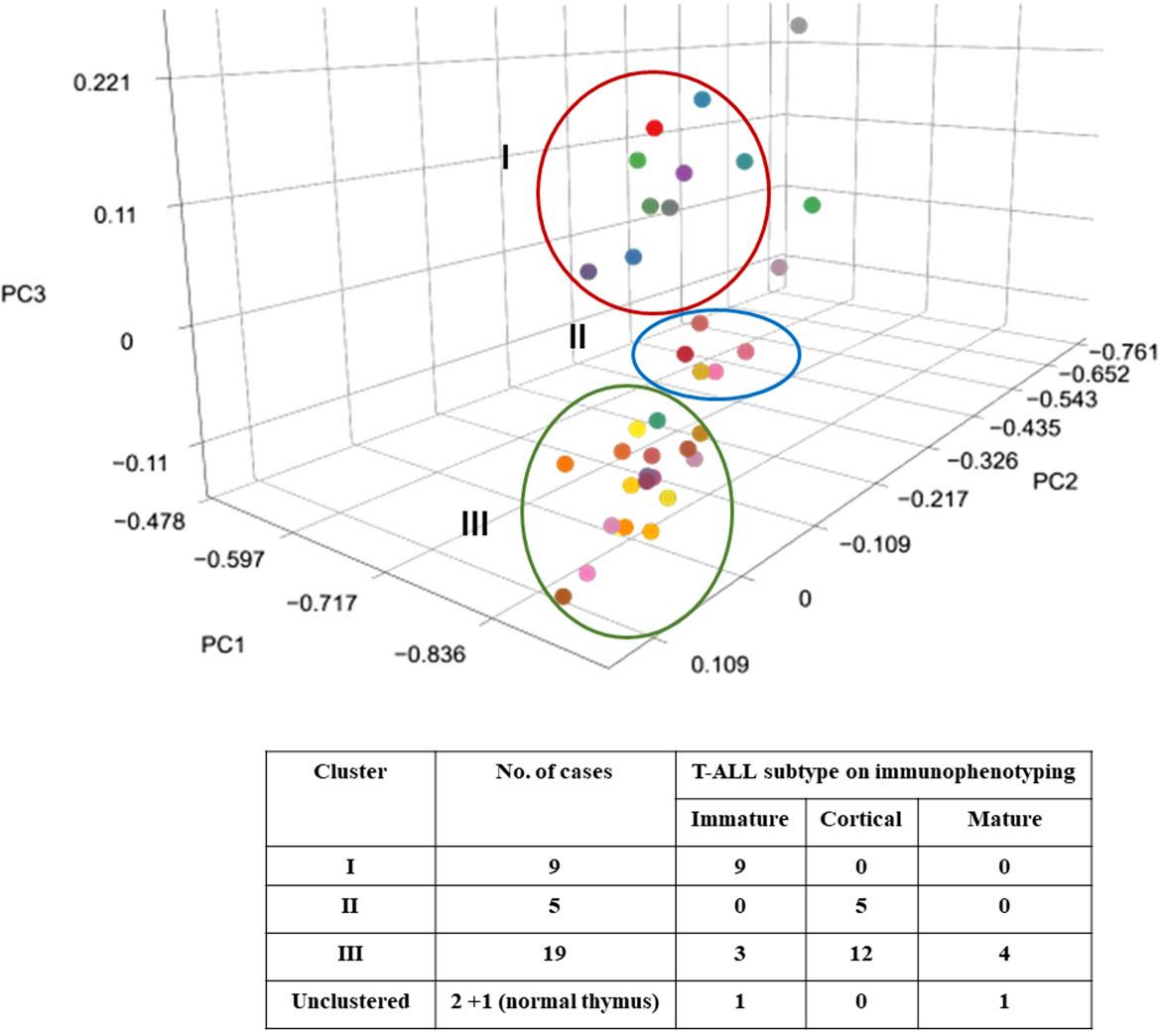
PCA of 35 T-ALL samples and 1 normal thymus sample based on the normalized FPKM count. The samples were clustered into three major clusters—I, II, and III. The detail of the immunophenotype of the samples is given in the table. Two T-ALL samples and one normal thymus did not group with any of the clusters. The different colored dots represent samples from different patients.

In PCA, the input was normalized gene expression per patient sample and one normal thymus sample (kind courtesy, Dr Jan Cools, Belgium). We found three separate clusters, as shown in Fig. 3, in which cluster 1, cluster 2, and cluster 3 comprised 9, 5, and 19 T-ALL cases, respectively. Three samples, including the normal thymus, did not group with any of the clusters. The patients with immature T-ALL immunophenotype were clustered together as cluster 1. Cluster 2 consisted of five samples, and all were cortical T-ALL. In cluster 3, out of 19 samples, 3 were immature, 4 were mature, and the remaining were cortical T-ALL. Three samples which did not fall into any cluster belonged to the normal thymus, immature T-ALL, and mature T-ALL, respectively.

#### Identification of fusion transcripts in the discovery cohort

**Fig. 4.**
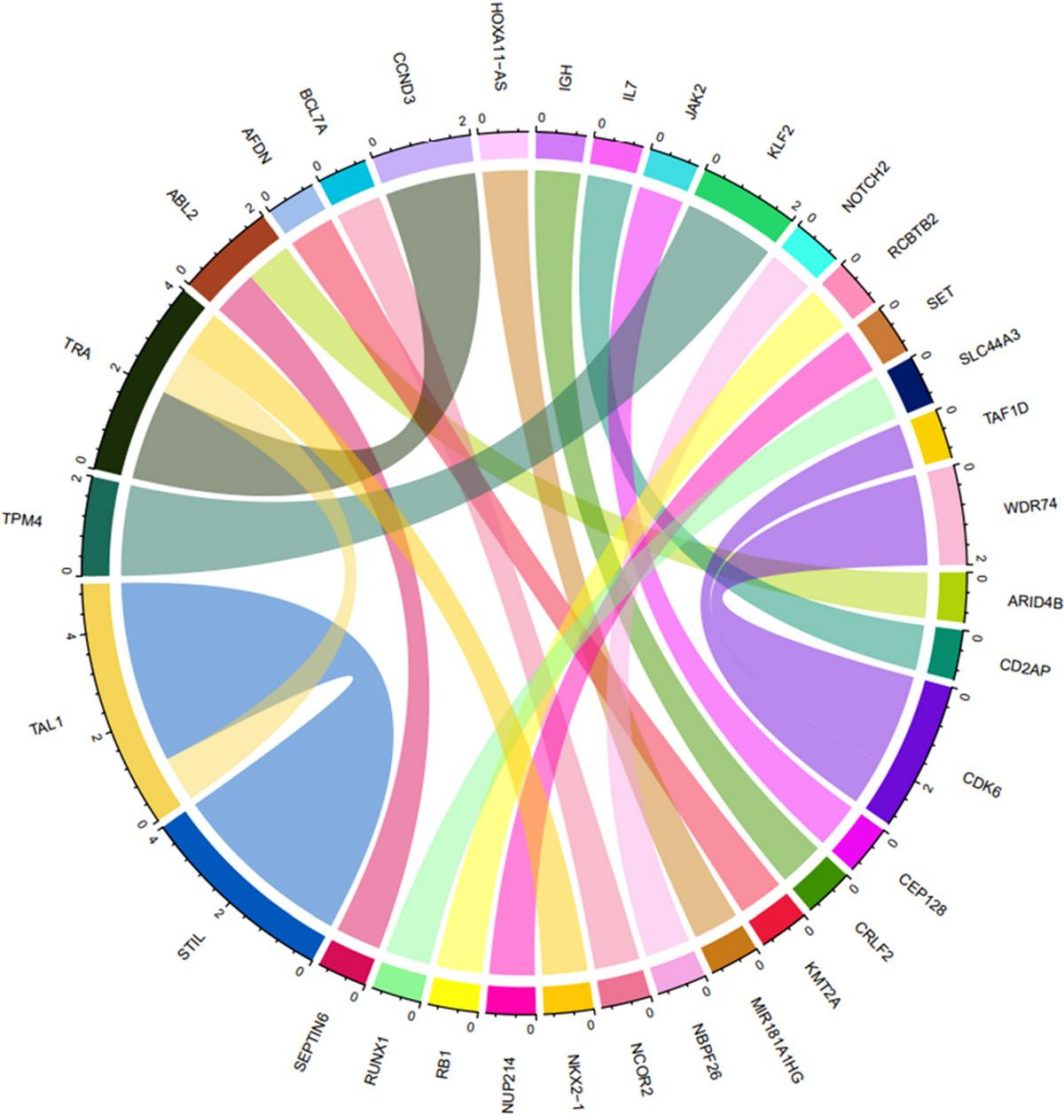
Circos plot depicting the fusion transcripts found in T-ALL cases in the discovery cohort.

Based on RNAseq data analysis, 23/35 (65.71%) patients harbored fusion transcripts. A total of 19 different fusion transcripts were identified, including 13 known and 6 novel fusion transcripts. Detailed results are presented in Table 1 and Fig. 4. Specifically, immature T-ALL cases were found to have fusion transcripts such as *KMT2A*::*AFDN*, *ARID4B*::*ABL2*, *SET*::*NUP214*, *TPM4*::*KLF2*, *MIR181A1HG*::*HOXA11-AS*, and *CDK6*::*WDR74*. Additionally, the fusion transcripts, such as *NKK2-1*::*TRA*, *CEB128*::*JAK2*, *CRLF2*::*IGH*, *TAL1*::*TRA*, *NBPF26*::*NOTCH2*, *CDK6*::*TAF1D*, *RB1*::*RCBTB2*, and *SEPTIN6*::*ABL2* were identified in cortical T-ALL cases. The fusions *TRA*::*CCND3* and *STIL*::*TAL1* were not exclusive to any specific T-ALL subtype. Interestingly, we detected more than one fusion transcript in two cases (one cortical and one immature). The cortical case had *CD2AP*::*IL7* and *NCOR2*::*BCL7A* fusion transcripts. The immature case had *MIR181A1HG*::*HOXA11-AS* and *CDK6*::*WDR74* fusion transcripts.

**Table 1.** Fusion transcripts observed in T-ALL samples in the discovery cohort by RNA sequencing.

Fusion transcript	5′ fusion partner	3′ fusion partner	No. of samples
Known fusions			
* STIL*::*TAL1*	*STIL*	*TAL1*	4
* CRLF2*::*IGH*	*CRLF2*	*IGH*	1
* TAL1*::*TRA*	*TAL1*	*TRA*	1
* SET*::*NUP214*	*SET*	*NUP214*	1
* SEPTIN6*::*ABL2*	*SEPTIN6*	*ABL2*	1
* NKX2-1*::*TRA*	*NKX2-1*	*TRA*	1
* KMT2A*::*AFDN*	*KMT2A*	*AFDN*	1
* MIR181A1HG*::*HOXA11-AS*	*MIR181A1HG*	*HOXA11-AS*	1
* TRA*::*CCND3*	*TRA*	*CCND3*	2
* RB1*::*RCBTB2*	*RB1*	*RCBTB2*	1
* NCOR2*::*BCL7A*	*NCOR2*	*BCL7A*	1
* CDK6*::*TAF1D*	*CDK6*	*TAF1D*	1
* TPM4*::*KLF2*	*TPM4*	*KLF2*	2
Novel fusions			
* CEP128*::*JAK2*	*CEP128*	*JAK2*	1
* CDK6*::*WDR74*	*CDK6*	*WDR74*	2
* ARID4B*::*ABL2*	*ARID4B*	*ABL2*	1
* NBPF26*::*NOTCH2*	*NBPF26*	*NOTCH2*	1
* RUNX1*::*SLC44A3*	*RUNX1*	*SLC44A3*	1
* CD2AP*::*IL7*	*CD2AP*	*IL7*	1

### Validation cohort

#### Clinical characteristics of patients in the validation cohort

In the validation cohort (*n* = 99), there were 47 immature (including 12 ETP-ALL), 36 cortical, and 16 mature T-ALL cases. The median age of the patients was 12 years (range, 1–65 years). There were 88 males (65 children, 23 adults) and 11 females (8 children, 3 adults). The median hemoglobin was 9.2 g/dL (range, 3.6–14.4 g/dL), median TLC 26.5 × 10^9^/L (range, 0.58–500 × 10^9^/L), and median platelet counts 50 × 10^9^/L (range, 5–391 × 10^9^/L). The cytogenetics data were available in 26 patients. The cytogenetics was normal in 21 (80.76%) patients. A detailed description of the baseline characteristics of the patients in the validation cohort is given in Supplementary Excel file.

We checked the expression levels of 23 RNA targets which were found to be differentially expressed in the discovery cohort. These targets were selected for each subtype of T-ALL, i.e. immature, cortical, and mature T-ALL, using available literature for T-ALL and other hematological and solid malignancies. They were chosen from different classes: (i) protein coding genes—*BAALC*, *HHEX*, *MEF2C*, *FAT1*, *LYL1*, *LMO2*, *LYN*, and *TAL1*; (ii) epigenetic modifiers—*DOT1L*, *EP300*, *EML4*, *EZH2*, *RAG1*, and *KDM6A*; and (iii) lncRNAs—*XIST*, *ST20*, PCAT18, *PCAT14*, *LINC00202*, *LINC00461*, *LINC00648*, *MEF2C-AS1*, and *MALAT1*. The expression of these targets was estimated by real-time PCR in the validation cohort to assess their clinical and prognostic significance in T-ALL.

#### Association between gene expression and immunophenotype in the validation cohort

We found a significant association between immature T-ALL immunophenotype with high expression of *BAALC* (*P* = 0.001), *MEF2C* (*P* = 0.002), *LYL1* (0.018), and *HHEX* (*P* = 0.007); and low expression of *EZH2* (*P* = 0.005). *RAG1* and *FAT1* expression was higher in cortical T-ALL (*P* = 0.004 and 0.033, respectively). *DOT1L* expression was higher in mature T-ALL (*P* = 0.025). ETP-ALL immunophenotype was associated with high levels of *BAALC* (*P* = 0.003), *MEF2C* (*P* = 0.003), *LYL1* (*P* = 0.01), *LYN* (*P* = 0.01), *XIST* (*P* = 0.02), and lower levels of *ST20* (*P* = 0.007) and *EML4* (*P* = 0.03). We also found an association between CD34 positivity on immunophenotyping with expression levels of *BAALC* (*P* = 0.032) and *MEF2C* (*P* = 0.012). Myeloid markers (CD13/CD33) expression on immunophenotyping was associated with high *BAALC* (*P* = 0.021) and low *ST20* (*P* = 0.007), and low *KDM6A* (*P* = 0.026). We did not find any significant association between the T-ALL subtype and expression levels of *PCAT14*, *PCAT18*, *TAL1*, *LMO2*, *XIST*, *EP300*, *MALAT1*, *LINC00202*, *LINC00461*, and *LINC00648*.

#### Association between gene expression and patients' variables in the validation cohort

On analysis of the association of patients' characteristics with expression levels of protein and noncoding RNAs, we found an association between *RAG1* expression and the age of the patients. Specifically, *RAG1* expression was higher in patients younger than 12 years compared with those older than 12 years (*P* = 0.034). Higher expression of *XIST* was observed in females (*P* = 0.011). Low *MALAT1* expression was associated with low TLC at diagnosis (*P* = 0.02). Patients with low *XIST*, low *KDM6A*, and high *TAL1* more frequently had NCI high risk (*P* = 0.01, 0.018, and 0.04, respectively). Prednisolone resistance was associated with high *MEF2C* and *HHEX* expressions (*P* = 0.048 and 0.018, respectively). Postinduction minimal residual disease (MRD) positivity (≥0.01%) was associated with high *PCAT18* (*P* = 0.04), *HHEX* (*P* = 0.027), and *MEF2C* (*P* = 0.007) expression.

#### Association between gene expression and treatment outcome in the validation cohort

In the validation cohort, 77 patients were treated with ICiCLe protocol (20), 19 with BFM-90 protocol (21), and 3 with hyper CVAD therapy (23). Complete remission was defined as bone marrow blasts <5% with a recovery of blood counts at the end of 4 weeks of induction chemotherapy. Any failure to do so (including the persistence of leukemic blasts at an extramedullary site) or death during induction therapy for any reason was considered induction failure. Patients who failed with one protocol were re-induced with another. The median follow-up was 27 months (range, 0.5–78 months). Complete remission was achieved in 84 (84.84%) patients with induction chemotherapy. Two patients died during induction therapy.

On survival analysis, the higher expression of *MEF2C* (hazard ratio [HR] 3.61, 95% CI 1.35–9.66, *P* = 0.006), *BAALC* (HR 3.58, 95% CI 1.36–9.42, *P* = 0.005), *HHEX* (HR 4.94, 95% CI 1.44–16.95, *P* = 0.005), *MALAT1* (HR 2.72, 95% CI 1.04–7.08, *P* = 0.033), and *LINC00202* (HR 3.27, 95% CI 1.13–9.42, *P* = 0.02) was found to be associated with worse overall survival (OS), while the higher expression of *ST20* (HR 0.3, 95% CI 0.12–0.77, *P* = 0.008), *TAL1* (HR 0.25, 95% CI 0.06–1.07, *P* = 0.042), *FAT1* (HR 0.35, 95% CI 0.14–0.89, *P* = 0.022), and *RAG1* (HR 0.3, 95% CI 0.12–0.77, *P* = 0.007) was associated with better OS (Table 2).

**Table 2.** Univariate analysis for prognostic association of RNA targets and other covariates in patients with T-ALL in the validation cohort.

Variables	Overall survival			Event-free survival			Relapse-free survival		
	HR	*P*-value	95% CI	HR	*P*-value	95% CI	HR	*P-*value	95% CI
Age (pediatrics vs. adults)	0.997	0.995	0.392–2.535	**2**.**072**	**0**.**016**	**1.148–3.739**	**2**.**484**	**0**.**006**	**1.299–4.751**
Sex (male vs. female)	1.312	0.661	0.390–4.419	0.658	0.423	0.236–1.832	0.596	0.389	0.183–1.937
TLC (<50 × 10^9^/L vs. >50 × 10^9^/L)	1.086	0.847	0.470–2.510	1.690	0.071	0.956–2.990	**1**.**915**	**0**.**043**	**1.020–3.596**
NCI risk (standard vs. high)	1.500	0.512	0.446–5.050	1.774	0.19	0.753–4.180	2.325	0.11	0.825–6.552
Immunophenotype									
* *Immature	REF			REF			REF		
* *Cortical	1.033	0.944	0.415–2.574	0.803	0.509	0.419–1.539	0.744	0.427	0.359–1.544
* *Mature	1.183	0.773	0.376–3.723	1.162	0.712	0.525–2.572	1.263	0.593	0.536–2.976
Prednisolone response (sensitive vs. resistant)	0.928	0.895	0.308–2.799	1.005	0.991	0.459–2.201	0.974	0.955	0.396–2.396
ETP immunophenotype (ETP vs. non-ETP)	0.947	0.93	0.281–3.193	**2**.**022**	**0**.**05**	**0.994–4.113**	**2**.**561**	**0**.**016**	**1.194–5.493**
MRD (negative vs. positive)	0.416	0.091	0.150–1.150	0.487	0.061	0.229–1.034	0.470	0.074	0.205–1.077
Protein-coding genes (low vs. high)									
* BAALC*	**3**.**58**	**0**.**0058**	**1.36–9.42**	**2**.**01**	**0**.**022**	**1.09–3.72**	**1**.**96**	**0**.**04**	**1.02–3.75**
* HHEX*	**4**.**94**	**0**.**0049**	**1.44–16.95**	**3**.**29**	**0**.**017**	**1.17–9.23**	**3**.**52**	**0**.**011**	**1.24–9.95**
* MEF2C*	**3**.**61**	**0**.**0063**	**1.35–9.66**	**2**.**19**	**0**.**013**	**1.16–4.13**	**2**.**49**	**0**.**009**	**1.23–5.05**
* LYL1*	2.29	0.064	0.93–5.65	**1**.**96**	**0**.**027**	**1.07–3.59**	**2**.**51**	**0**.**004**	**1.32–4.8**
* TAL1*	**0**.**25**	**0**.**042**	**0.06–1.07**	**0**.**45**	**0**.**014**	**0.23–0.87**	0.61	0.22	0.28–1.35
* FAT1*	**0**.**35**	**0**.**022**	**0.14–0.89**	0.56	0.06	0.31–1.03	0.55	0.063	0.29–1.04
* LYN*	2.25	0.1	0.83–6.11	1.71	0.11	0.87–3.34	1.91	0.14	0.79–4.61
* LMO2*	0.53	0.26	0.18–1.62	**2**.**33**	**0**.**006**	**1.24–4.35**	**2**.**64**	**0**.**0037**	**1.34–5.2**
Epigenetic modifiers (low vs. high)									
* DOT1L*	1.88	0.19	0.71–4.94	1.42	0.27	0.76–2.67	1.61	0.16	0.83–3.12
* EP300*	0.73	0.51	0.28–1.88	1.54	0.22	0.77–3.06	1.52	0.25	0.74–3.15
* EML4*	0.5	0.16	0.19–1.33	0.71	0.31	0.37–1.37	0.66	0.23	0.34–1.9
* EZH2*	0.53	0.2	0.19–1.43	0.71	0.31	0.36–1.38	1.41	0.31	0.73–2.73
* KDM6A*	1.42	0.49	0.52–3.86	0.69	0.23	0.37–1.28	1.37	0.41	0.61–2.92
* RAG1*	**0**.**3**	**0**.**007**	**0.12–0.77**	**0**.**52**	**0**.**04**	**0.28–0.98**	**0**.**44**	**0**.**013**	**0.22–0.85**
lncRNAs (low vs. high)									
* MALAT1*	**2**.**72**	**0**.**033**	**1.04–7.08**	0.57	0.08	0.3–1.08	1.91	0.058	0.99–3.69
* ST20*	**0**.**3**	**0**.**008**	**0.12–0.77**	**0**.**52**	**0**.**049**	**0.27–1.01**	**0**.**5**	**0**.**045**	**0.25–1**
* XIST*	1.93	0.15	0.78–4.81	**0**.**38**	**0**.**001**	**0.2–0.7**	**0**.**43**	**0**.**01**	**0.22–0.83**
* LINC00648*	2.38	0.095	0.83–6.82	1.56	0.21	0.78–3.1	1.51	0.25	0.74–3.08
* PCAT18*	0.18	0.061	0.02–1.37	0.8	0.51	0.4–1.57	1.26	0.51	0.63–2.51
* LINC00461*	0.19	0.074	0.02–1.46	**0**.**32**	**<0**.**001**	**0.16–0.63**	**0**.**3**	**0**.**0004**	**0.15–0.61**
* LINC00202*	**3**.**27**	**0**.**02**	**1.13–9.42**	1.49	0.22	0.76–2.82	0.7	0.31	0.36–1.38
* MEF2C-AS1*	2.37	0.08	0.88–6.39	1.72	0.094	0.91–3.27	1.68	0.13	0.65–3.32
* PCAT14*	0.54	0.28	0.17–1.67	0.53	0.092	0.25–1.12	0.52	0.098	0.23–1.14

All values in bold indicates the statistical significant parameters.

**Fig. 5.**
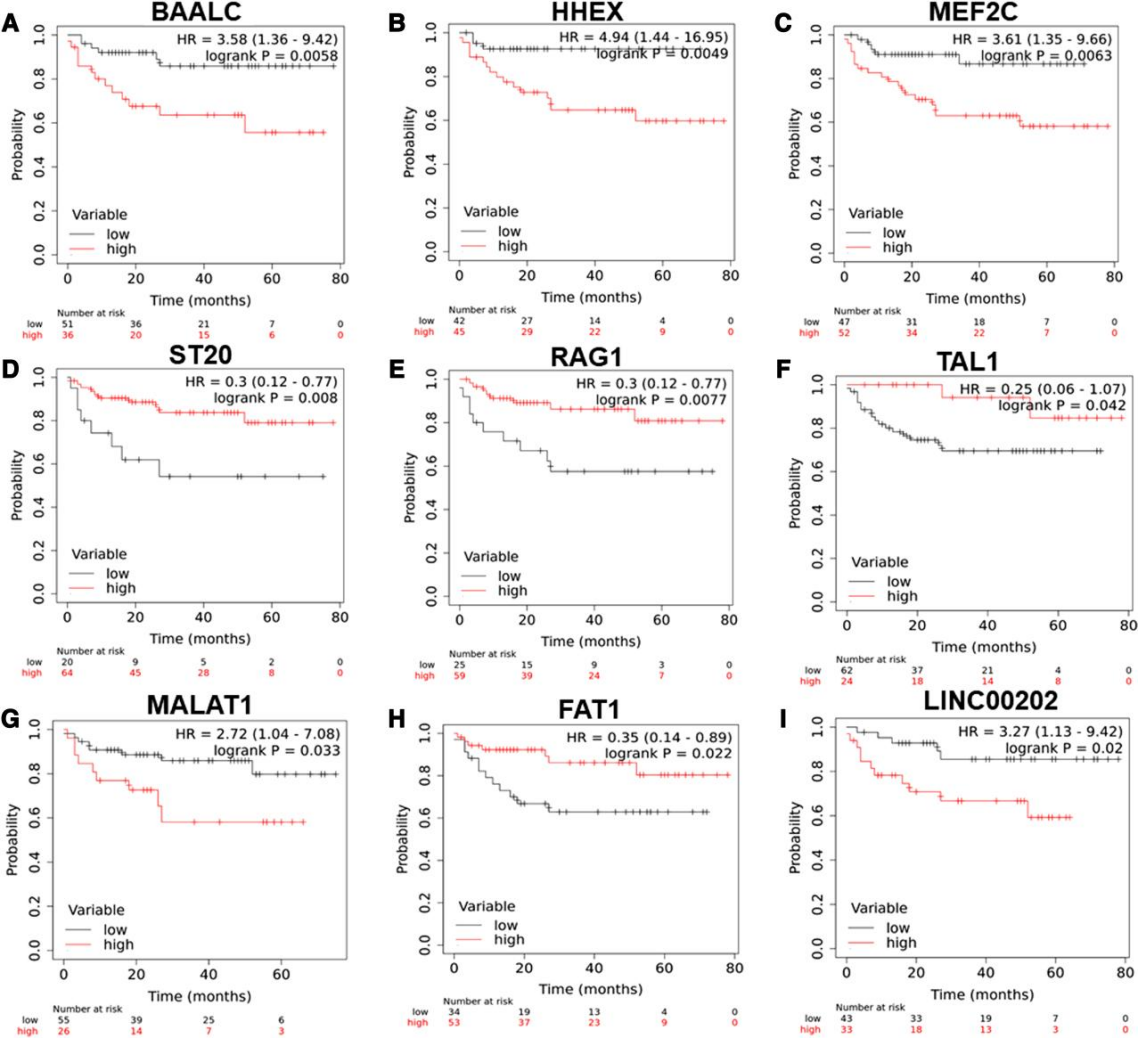
Kaplan–Meier analysis for OS for expression of A) *BAALC*, B) *HHEX*, C) *MEF2C*, D) *ST20*, E) *RAG1*, F) *TAL1*, G) *MALAT1*, H) *FAT1*, and I) *LINC00202* in patients with T-ALL in the validation cohort.

**Fig. 6.**
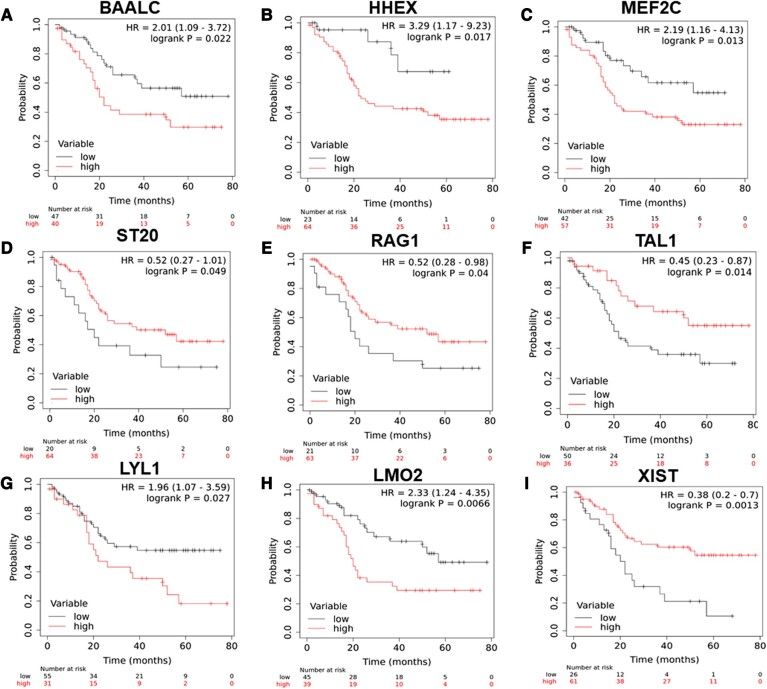
Kaplan–Meier analysis for EFS for expression of A) *BAALC*, B) *HHEX*, C) *MEF2C*, D) *ST20*, E) *RAG1*, F) *TAL1*, G) *LYL1*, H) *LMO2*, and I) *XIST* in patients with T-ALL in the validation cohort.

**Fig. 7.**
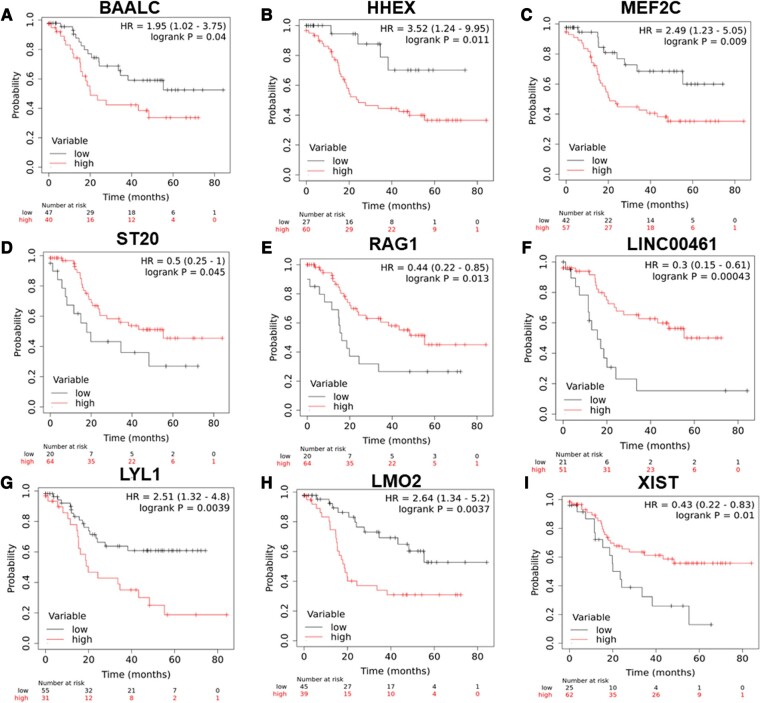
Kaplan–Meier analysis for RFS for expression of A) *BAALC*, B) *HHEX*, C) *MEF2C*, D) *ST20*, E) *RAG1*, F) *LINC00461*, G) *LYL1*, H) *LMO2*, and I) *XIST* genes in patients with T-ALL in the validation cohort.

Additionally, age ≥18 years and ETP-ALL immunophenotype were associated with poor event-free survival (EFS; HR 2.07, 95% CI 1.14–3.73, *P* = 0.016; HR 2.02, 95% CI 0.99–4.11, *P* = 0.05, respectively) and relapse-free survival (RFS; HR 2.48, 95% CI 1.29–4.75, *P* = 0.006; HR 2.56, 95% CI 1.19–5.49, *P* = 0.016, respectively). Higher TLC count (HR 1.91, 95% CI 1.01–3.59, *P* = 0.043) was also associated with poor RFS. In addition, higher expression of *BAALC, MEF2C*, *HHEX*, and *LYL1* was associated with poor EFS (HR 2.01, 95% CI 1.09–3.72, *P* = 0.022; HR 2.19, 95% CI 1.16–4.13, *P* = 0.013; HR 3.29, 95% CI 1.17–9.23, *P* = 0.017; and HR 1.96, 95% CI 1.07–3.59, *P* = 0.027, respectively) and RFS (HR 1.96, 95% CI 1.02–3.75, *P* = 0.04; HR 2.49, 95% CI 1.23–5.05, *P* = 0.009; HR 3.52, 95% CI 1.24–9.95, *P* = 0.011; and HR 2.51, 95% CI 1.32–4.8, *P* = 0.004, respectively). High *LMO2* expression was associated with poor EFS (HR 2.33, 95% CI 1.24–4.35, *P* = 0.006) and RFS (HR 2.64, 95% CI 1.34–5.2, *P* = 0.0037). Furthermore, low *XIST* (HR 0.38, 95% CI 0.2–0.7, *P* = 0.001; HR 0.43, 95% CI 0.22–0.83, *P* = 0.01), *LINC00461* (HR 0.3, 95% CI 0.15–0.59, *P* = 0.001; HR 0.3, 95% CI 0.15–0.61, *P* = 0.0004), and *ST20* (HR 0.52, 95% CI 0.27–1.01, *P* = 0.049; HR 0.5, 95% CI 0.25–1, *P* = 0.045) were associated with worse EFS and RFS, respectively. Low *TAL1* was associated with poor EFS (HR 0.46, 95% CI 0.23–0.89, *P* = 0.023), but not associated with the RFS (Table 2, Figs. 5–7 and S2–S4).

On multivariate analysis, high *BAALC* (HR 11.57, 95% CI 1.97–67.79, *P* = 0.007; HR 5.42, 95% CI 1.71–17.31, *P* = 0.004; HR 7.28, 95% CI 1.78–29.72, *P* = 0.006; Table S6), *MEF2C* (HR 4.64, 95% CI 1.22–17.63, *P* = 0.024; HR 2.75, 95% CI 1.00–7.55, *P* = 0.049; HR 4.58, 95% CI 1.24–16.85, *P* = 0.022; Table S7), *HHEX* (HR 9.25, 95% CI 1.62–52.65, *P* = 0.012; HR 8.63, 95% CI 1.63–45.52, *P* = 0.011; HR 7.56, 95% CI 1.43–39.90, *P* = 0.016; Table S8), and *LYL1* expression (HR 6.66, 95% CI 1.56–28.46, *P* = 0.01; HR 3.51, 95% CI 1.28–9.62, *P* = 0.014; HR 6.35, 95% CI 1.98–20.32, *P* = 0.002; Table S9) and low *ST20* (HR 0.08, 95% CI 0.018–0.37, *P* = 0.001; HR 0.08, 95% CI 0.021–0.35, *P* = 0.001; HR 0.1, 95% CI 0.019–0.50, *P* = 0.005; Table S10) and *RAG1* (HR 0.20, 95% CI 0.053–0.79, *P* = 0.021; HR 0.26, 95% CI 0.089–0.78, *P* = 0.017; HR 0.20, 95% CI 0.059–0.72, *P* = 0.014; Table S11) were found to be associated with worse OS, EFS and RFS, respectively. Furthermore, on multivariate analysis, higher *LMO2* expression (HR 3.34, 95% CI 1.05–10.57, *P* = 0.04; HR 6.05, 95% CI 1.49–24.42, *P* = 0.011; Table S12) was found to be associated with inferior EFS and RFS, respectively. In addition, higher expression of *DOT1L* (HR 4.96, 95% CI 1.11–22.10, *P* = 0.035; Table S13) and lower expression of *PCAT14* (HR 0.066, 95% CI 0.006–0.73, *P* = 0.027; Table S14) emerged as independent prognostic markers for RFS.

## Discussion

T-ALL is a genetically heterogeneous malignancy that poses significant challenges in risk stratification and clinical management. In our study, we employed RNA sequencing to comprehensively analyze the expression profiles of protein-coding genes, epigenetic modifiers and lncRNAs in patients with T-ALL. The aim was to unravel the molecular landscape of T-ALL and assess the clinical relevance of identified RNA targets. Our findings revealed distinct expression profiles of protein-coding genes, epigenetic modifiers, and lncRNA transcripts across immunophenotypic subtypes of T-ALL. The key genes which served as transcription factors in early hematopoiesis, such as *MEF2C*, *LYL1*, *LMO2*, *HHEX*, *RUNX2*, *HOXA10*, *HOXA9*, *RUNX1T1*, and *ZBTB16*, were up-regulated in immature T-ALL. The dysregulation of *MEF2C* has been previously shown in immature T-ALL (28–34). Colomer-Lahiguera et al. (29). reported that *MEF2C* dysregulation is associated with *CDKN1B* deletion and poor prednisolone response in T-ALL. We also found an association between prednisolone resistance and high *MEF2C* expression. La Starza et al. (35) showed its up-regulation in T-ALL cases with interstitial deletion of 5q. Nagel et al. (34) proposed distinct mechanisms for aberrant *MEF2C* gene expression, either by NKX2-5 signaling or by chromosomal deletion of 5q. They also showed that *MEF2C* inhibits BCL2-regulated apoptosis by inhibition of NR4A1/NUR77 (34). In addition, Kawashima-Goto et al. (30) reported that BCL*2* inhibitors might be helpful for treating T-ALL with high expression levels of *MEF2C*. In our study, MEF2C gene expression emerged as a significant predictor of EFS, RFS, and OS. Although MEF2C overexpression is associated with chemoresistance and poor outcomes in AML (36), its prognostic relevance in T-ALL is not clear. It was recently reported by our group for the first time (37).

In our study, *MN1*, *BAALC*, and *IGFBP7* were overexpressed in immature T-ALL. Up-regulation of these genes is believed to arise from T-cell progenitors retaining myeloid differentiation potential (25, 38, 39). Like previous studies, we found *BAALC* overexpression to be associated with the expression of CD34 and myeloid markers (25, 38). Previous studies showed overexpression of these genes was associated with poor outcome and resistance to chemotherapy (38, 40). We did not find any significant association between *BAALC* expression and prednisolone sensitivity. Interestingly, higher *BAALC* expression was associated with worse patient outcome. Increased *BAALC* expression is an important marker for chemoresistance and poor patient prognosis in myeloid and lymphoid malignancies. Baldus et al. (40) reported that low expression of *ERG* and *BAALC* predicts a favorable outcome in T-ALL; however, another report suggested no association of *BAALC* with the prognosis in a larger cohort of 232 adult patients with T-ALL (41).

*HHEX* (hematopoietically expressed homeobox transcription factor) plays a pivotal role in the development of various hematological malignancies, most notably T-ALL and AML (42). It has been shown to act as direct transcriptional target of *LMO2* and concordantly expressed with *LYL1* in human ETP-ALL (43). It has also been reported to be required for radio-resistance of leukemic stem cells. Its expression is known to be down-regulated by deacetylation treatment signifying the role of this therapy in T-ALL (43). In our study, *HHEX* overexpression was associated with worse OS, EFS, and RFS. This has never been reported in the literature before.

*LYL1* codes for a transcriptional factor involved in leukemia progression (44, 45). *LYL1* interacts with *LMO2* expressed in ETP-ALL. It regulates the stem cells' signature of thymocytes, and generates T-cell leukemia (46). Therefore, *LYL1* is an essential factor for *LMO2*-driven T-cell leukemia (47). We observed that *LYL1* overexpression in T-ALL was associated with the unfavorable EFS and RFS. In myeloid malignancies, *LYL1* has been shown to be associated with a lower remission rate, higher relapse rate, and poor patient survival (48). In addition, we also observed higher *LMO2* expression to be associated with poor EFS and RFS. Contrary to this, previous studies have suggested *LMO2* expression to be associated with a better prognosis in B-ALL and T-ALL (49, 50).

We also found overexpression of *ZBTB16 (PLZF)* in immature T-ALL, although not stressed in previous western studies, was a notable finding in a Chinese study (28, 51). *ZBTB16* (or promyelocytic leukemia zinc finger, PLZF) contains one BTB domain and nine zinc fingers. Its overexpression was shown in that study to result from *ZBTB16::ABL1* translocation and occurred in different patients along with other mutations, including *NOTCH1*, *ZEB2*, *PTEN*, *MYCN,* and *PIK3CD*. In addition, laboratory studies in Jurkat cells and mice showed that *ZBTB16::ABL1* to be a leukemogenic driver lesion that caused increased proliferation and a 4-fold heightened protein tyrosine kinase activity that was amenable to tyrosine kinase inhibitor (TKI) activity (28). Although we did not find this translocation in our patients, our finding is also significant because, along with *LYN* overexpression, ZBTB16 overexpression means that our patients of immature T-ALL may benefit from TKIs.

Apart from these known genes, we identified aberrantly expressed unreported genes such as *RUNX1T1*, *RUNX2*, *PLD4*, *NT5E (CD73)*, *HOPX*, *TP63*, and *HOXA11-AS.* Furthermore, a role for *RUNX2* in T-ALL has been suggested in a study by Nagel et al. (34), who, in order to uncover additional target genes, investigated in detail the aberrant expression of *MEF2C* mediated by complex deletion at 5q, del(5)(q14) in T-ALL cell line LOUCY. This could be an evidence that *RUNX2* instead of *RUNX1* could be involved in the manifestation of ETP-ALL that allows in vivo functional evaluation of putative oncogenes and preclinical drug testing.

Further, analysis of cortical T-ALL yielded differentially expressed genes with *CD1A*, *CD1C*, *CD4*, *CFTR*, *FAT3*, *NKX2-1*, *TLX1*, *TLX3*, and *RAG1* being reported earlier in various reports, while *EREG*, *PAX6*, and *ZIC2* were identified to be up-regulated in the present study. Neumann et al. (52), in a study of adult ETP-ALL, showed that cadherins *FAT1* (25%) and *FAT3* (20%) were mutated, implicating alterations in cell adhesion and activation of the Wnt pathway. In another study, Neumann et al. (53) showed that *FAT1* expression was correlated with a more mature leukemic immunophenotype in T-ALL, with 74% of patients with thymic T-ALL being *FAT1* positive compared with 45% of patients with mature T-ALL and only 4% of patients with early T-ALL. Expression of *FAT1* in our cortical T-ALL is in keeping with this finding. Like the previous study (53), we also observed a correlation between FAT1 expression and patient outcome.

Mature T-ALL is a rare subgroup immunophenotypically diagnosed by CD1a^−^ and sCD3^+^. Molecularly, *TAL1* is a driver gene for late cortical T-ALL (1). We found *TAL1* to be overexpressed in both mature and cortical T-ALL. Among the protein-coding genes, *APC2*, *BCL3*, *CCR4*, *ST20*, *EML4*, and *NCOR2* were some of the key up-regulated genes. TAL1 underexpression was found to be associated with poor OS and EFS in our study.

Aberrant histone modifications are the hallmark of cancer and are associated with dysregulated expression of histone modifiers. We also studied their expression to identify a set of histones modifying enzymes to be up-regulated or explicitly down-regulated in different subtypes of T-ALL. *EZH2*, a member of the polycomb repressor complex, was underexpressed in our immature T-ALL cases. This may be related to their mutations in immature T-ALL (54). Danis et al. (55) mechanistically linked *EZH2* inactivation to stem-cell-associated transcriptional programs and increased growth/survival signaling, features that convey an adverse prognosis in patients. However, we did not find a correlation between *EZH2* expression and outcome. Loss-of-function mutations and deletions in *SETD2* have been shown to lead to chemotherapy tolerance and clonal survival by cell cycle arrest followed by apoptosis. Hence, the overexpression of *SETD2* has been postulated to develop chemotherapy resistance in many cancers, including leukemias (54, 56–58). We found overexpression of *SETD2* in T-ALL when compared with normal thymus. *SETD2* in leukemic patients may develop chemotherapy resistance. This can be further investigated to see the involvement of histone methylation at the genomic level to correlate it with the transcriptional inferences. In pediatric cases, higher expression of *HDAC7* and *HDAC9* in ALL can be associated with poor prognosis. In our study, we observed overexpression of *HDAC9* in our immature and cortical cases. *CREBBP*, *EP300*, *ASH1L*, *ATM*, *PKN1*, *KDM2B*, *KDM4B*, and *DOT1L* showed significant differential expression in mature T-ALL. *EP300* and *CREBBP* have lysine acetyltransferase activity in transcription coactivation (59–62). Targeted histone lysine acetylation of *EP300* and *CREBBP* can influence chromatin conformation (60), and concomitant binding of *EP300* and acetylation of H3K27 are hallmarks of promoter or enhancer activation (63). For the first time, we observed that the low expression of *RAG1* is associated with poor OS, EFS, and RFS.

DOT1-like (*DOT1L*) histone lysine methyltransferase methylates H3K79 and plays a significant role in embryogenesis and hematopoiesis. Its function is unknown in T-ALL, but its aberrant activation is associated with other acute leukemia (64, 65). *DOT1L* catalytic activity depends on the monoubiquitination of lysine120 in histone H2B (H2BK120Ub), which provides crosstalk between histone posttranslational modifications (66). Recent studies suggested the role of *DOT1L* in H3K79 methylation and monoubiquitination of lysine (H2BK120Ub) that may pave the way for developing novel *DOT1L*-driven antileukemia therapies (67, 68). *DOT1L* was overexpressed in our patients with mature T-ALL, and it may be worth investigating if they could be subjects for *DOT1L*-driven antileukemia therapy.

Apart from proteins, noncoding repertoire forms another layer of the regulatory paradigm in normal cell hemostasis. Using RNAseq, we tried to identify the differentially expressed noncoding RNAs, especially lncRNAs, which were well documented earlier for their role in cancers. Recent studies have revealed lncRNA's aberrant expression profile in T-ALL, leading to deregulated downstream signaling pathways (69). An in-depth analysis revealed 2,243 lncRNAs, with 223 showing differential expression. *NOTCH1*-regulated lncRNA, *LUNAR1*, was overexpressed in cortical and mature T-ALL (27). This may be related to a higher incidence of activating *NOTCH1*1 mutations in these T-ALL subtypes (8, 70). *HOTTIP* and *MEF2C-AS1* were overexpressed in immature; *LINC00202*, *LINC0648*, and *LINC00461* in cortical T-ALL and *MALAT1* in mature T-ALL. We also observed higher *LINC00202* expression to be associated with poor OS, while lower expression of *LINC00461* was significantly associated with adverse patient outcomes. Both of these associations have not been reported previously. *XIST* was associated with worse EFS and RFS. *HOTTIP* has been reported to be aberrantly activated in AML. It promotes hematopoietic stem-cell renewal leading to AML-like disease in mice (71). This may explain its overexpression in immature T-ALL, which has myeloid potential in our study. We found the overexpression of *ST20* (Suppressor of Tumorigenicity 20) in T-ALL cases with better OS, EFS, and RFS. This has never been reported before.

*MALAT1* (metastasis-associated lung adenocarcinoma transcript 1) is known to be involved in a plethora of biological processes ranging from alternative splicing, nuclear organization, and epigenetic regulation of gene expression. It is also associated with various pathological complications such as breast cancer, lung adenocarcinomas, hepatocellular carcinomas, bladder cancers, and diabetes (72–74). The up-regulated level of *MALAT1* is often used as a prognostic marker for various cancer types (75). At a molecular level, *MALAT1* plays an important role in modulating several signaling pathways like *MAPK/ERK*, *PI3K/AKT*, *WNT*, and *NF-kB*, leading to a modification of proliferation, cell death, cell cycle, migration, invasion, immunity, angiogenesis, and tumorigenicity. We also report the association of *MALAT1* with the adverse OS in T-ALL. The exact mechanism of how *MALAT1* helps in cancer development and progression is unknown. *MALAT1* can be a therapeutic target, potential diagnostic, and prognostic biomarker for cancers (73, 76, 77).

Our PCA results identified three separate clusters. The clustering seems to somewhat reflect the immunophenotypic characteristics of the leukemia samples, with cluster 1 being associated with the immature immunophenotype, cluster 2 being exclusive to cortical T-ALL, and cluster 3, being more heterogeneous, included a mix of immature, mature, and cortical T-ALL cases. The presence of outliers, the two T-ALL samples, that did not fall into any cluster, adds complexity to the analysis. These outliers may represent unique molecular or genetic profiles that differ from the main clusters identified. Overall, our results provide valuable insights into the heterogeneity of gene expression patterns in T-ALL. Our results also indicate that the immunophenotyping of T-ALL, based on currently available immunomarkers, does not fully capture the molecular phenotype of the leukemic cells. This may prompt further research to identify and validate novel biomarkers or integrate multiple data types (genomic, proteomic, etc.) to enhance the precision of T-ALL diagnosis and potentially guide more targeted therapeutic approaches.

We also investigated for fusion transcripts in our patients and found many known and novel fusion transcripts. We found *TCR* gene to be fused with known oncogenes like *NKX2-1*, *CCND3*, and *TAL1* (28, 78). *STIL*::*TAL1* was identified in four cases but it was not specific for any particular T-ALL subtype. We also found a previously reported *MIR181A1HG*::*HOXA11-AS* fusion in a case of immature T-ALL (78). *MIR181A1HG* gene is located on chromosome 1q32. This region has also been reported to be rearranged with *MYC* gene in a case of T-ALL (78). Interestingly, we found *SEPTIN6*::*ABL2* fusion in a cortical T-ALL case. This has been recently described in a T-ALL case at diagnosis and relapse (79). This fusion was shown to have oncogenic potential and responded to TKI highlighting the fact that this fusion oncoprotein can be used as therapeutic target in T-ALL (79). We found *CRLF2*::*IGH* fusion in a case with cortical immunophenotype. Although *CRLF2* overexpression has been described in T-ALL, *CRLF2*::*IGH* has not been reported (80). In contrast to our finding, *CRLF2* overexpression has been reported to be associated with immature-like immunophenotype (80). *TPM4*::*KLF2*, *RB1*::*RCBTB2*, and *NCOR2*::*BCL7A* fusions have been reported in B-ALL (81–83). The novel fusion transcripts found in our study were *CEP128*::*JAK2*, *CDK6*::*WDR74*, *ARID4B*::*ABL2*, *NBPF26*::*NOTCH2*, *RUNX1*::*SLC44A3*, and *CD2AP*::*IL7*. These novel fusions highlight the power of RNA sequencing in identifying fusion transcripts even in cases with normal karyotype (78). The oncogenic potential of these fusions needs to be validated in future studies.

Our results suggest that certain RNA signatures may have prognostic value, potentially aiding in risk stratification and personalized treatment approaches for patients with T-ALL. The study also addresses the gap in knowledge regarding the prognostic relevance of lncRNAs and histone modifiers in T-ALL. By shedding light on these unexplored aspects, our findings contribute to a more comprehensive understanding of the molecular landscape of T-ALL, paving the way for future research and potential clinical applications. Our findings also provide insights into the heterogeneity of fusion transcripts in T-ALL, including their distribution across different subtypes and the presence of multiple fusion transcripts in some cases. The data suggest a complex landscape of genetic alterations in T-ALL, which could have implications for understanding the disease and developing targeted therapies.

While our study provides valuable insights, it is important to acknowledge its limitations. The relatively modest sample size and the need for further validation in larger cohorts are recognized. Additionally, the dynamic nature of RNA expression patterns in leukemia necessitates longitudinal studies to elucidate the temporal evolution of these molecular signatures during disease progression and treatment response. Furthermore, the oncogenicity of the novel fusion transcripts identified in our study needs to be validated by in-vitro studies. The patient-derived animal models should be used to investigate the leukemogenesis and drug response.

In conclusion, our study elucidates the profile of protein-coding genes, epigenetic modifiers and lncRNA expression in T-ALL, revealing potential clinical implications. These findings not only advance our understanding of the molecular basis of T-ALL but also open avenues for the development of targeted therapies and improved risk stratification in the clinical management of this challenging disease. Future investigations building upon these results may uncover additional layers of complexity in T-ALL biology, ultimately guiding the development of more effective and personalized treatment strategies.

## Materials and methods

### Patients

A total of 134 T-ALL cases diagnosed by morphology, cytochemistry, and immunophenotyping were enrolled in this study. The patients with T-ALL were divided into two cohorts: discovery (*n* = 35) and validation (*n* = 99) (Fig. 1). All patients or legal guardians gave their informed consent for blood/bone marrow collection and biological analyses in accordance with the Declaration of Helsinki. The All India Institute of Medical Sciences, New Delhi Institutional Ethical Committee approved the study. The transcriptome data from pooled RNA of 5 normal human thymus samples were made available from European Genome-phenome Archive (EGA, http://www.ebi.ac.uk/ega/, kind courtesy of Dr Jan Cools, Belgium). The data were used as a control in the required analysis, like in PCA. These thymus samples were de-identified prior to use in our study.

### RNA sequencing and analysis

RNA was isolated from patient samples by the TRIzol method (Thermo Fisher Scientific, MA, USA). Paired-end whole transcriptome sequencing was performed on the Illumina HiSeq2000 platform using the Truseq RNA sample preparation kit (Illumina, San Diego, CA, USA). Sequence reads were processed to identify the expression profile of protein-coding and noncoding RNAs by supervised and unsupervised approaches (Supplementary Methods). To investigate the role of histone modifiers in T-ALL development, the transcript abundance of epigenetic modifiers was measured across mature, cortical, and immature subtypes of T-ALL (Supplementary Methods). To know the novel lncRNA transcript, a computational pipeline combining open reading frame prediction coupled with the coding potential calculator (CPC algorithm) to annotate the *protein-coding* potential of transcripts was used (Supplementary Methods). The examination of fusion transcripts across all samples was conducted using the freely accessible online tool FusionCatcher (https://doi.org/10.1101/011650; Supplemental Methods). The novel fusions were validated by RT-PCR followed by Sanger sequencing.

### Determination of the expression of RNA targets by real-time PCR

Total RNA was extracted from blast enriched mononuclear cells isolated from bone marrow samples collected at the time of diagnosis using the TRIzol method (Thermo Fisher Scientific). The concentration and quality of RNA were determined with spectrophotometer. RNA was reverse transcribed to cDNA using random hexamers, RNase inhibitor, dNTPs, and M-MuLV reverse transcriptase enzyme (Fermentas, USA). The expression levels of the targets were measured by real-time PCR (CFX96, Bio-Rad, Hercules, CA, USA) using TaqMan probe PCR master mix (Bio-Rad). The primers and probes used are given in Supplementary Excel file. In all cases, the samples were run in triplicates. The Ct values were normalized with housekeeping genes. The relative expressions were calculated using the comparative cycle threshold method.

### Statistical analysis

Fisher's exact test for categorical data and the nonparametric Mann–Whitney *U* test for continuous variables were used to compare baseline clinical variables across groups in the validation cohort. Kruskal–Wallis test was used to determine the association between gene expression and immunophenotype. A *P*-value ≤0.05 (two-sided) was considered significant. EFS was defined as the time from diagnosis to the date of the last follow-up in complete remission or the first event (i.e. induction failure, relapse, secondary neoplasm, or death from any cause). OS was defined as the time from diagnosis to death or the last follow-up. Patients lost to follow-up were censored at the last contact. RFS is the time from the remission date to the last follow-up, relapse date, or death from any cause. The last follow-up was carried out on 2022 May 15. The Kaplan–Meier (KM) survival analysis was performed to estimate survival rates, with the differences compared using a two-sided log-rank test. Cox proportional hazard models were constructed as univariate and multivariate analyses for association with EFS, RFS, and OS. Covariates included in the full model of OS, EFS, and RFS were gender, WBC (<50 × 10^9^/L, ≥ 50 × 10^9^/L), age, gene expression, immunophenotype, response to prednisolone treatment, and presence of MRD after the end of induction chemotherapy. Patients with high and low expressions were delineated using KM plotter tool (84) for each target (*BAALC*, *HHEX*, *MEF2C*, *FAT1*, *LYL1*, *LMO2*, *LYN*, *TAL1*, *DOT1L*, *XIST*, *PCAT18*, *PCAT14*, *LINC00202*, *LINC00461, LINC00648*, *MEF2C-AS1*, *ST20*, *RAG1*, *EP300*, *EML4*, *EZH2*, *MALAT1*, and *KDM6A)*. All analyses were performed using the SPSS statistical software package, version 20.0/STATA software, version 11.

## Supplementary Material

pgae011_Supplementary_Data

## Data Availability

The raw and processed RNA sequencing data have been submitted in GEO (http://www.ncbi.nlm.nih.gov/geo/; accession no.: GSE216117). Normal thymus RNAseq data are available at EBI-EGA with accession no. EGAS00001000536.

## Preprints

The initial version of this manuscript was posted on a preprint MEDRXIV/2021/255823; doi: https://doi.org/10.1101/2021.05.06.21255823.

## References

[1] Belver L, Ferrando A. 2016. The genetics and mechanisms of T cell acute lymphoblastic leukaemia. Nat Rev Cancer. 16(8):494–507. 27451956 10.1038/nrc.2016.63

[2] Dores GM, Devesa SS, Curtis RE, Linet MS, Morton LM. 2012. Acute leukemia incidence and patient survival among children and adults in the United States, 2001–2007. Blood. 119(1):34–43. 22086414 10.1182/blood-2011-04-347872 PMC3251235

[3] Arya LS, et al 2011. Childhood T-lineage acute lymphoblastic leukemia: management and outcome at a tertiary care center in North India. Indian Pediatr. 48(10):785–790. 21555798 10.1007/s13312-011-0129-3

[4] O’Connor D, et al 2023. The clinicogenomic landscape of induction failure in childhood and young adult T-cell acute lymphoblastic leukemia. J Clin Oncol. 41(19):3545–3556. 37098241 10.1200/JCO.22.02734 PMC10306434

[5] Durinck K, et al 2015. Novel biological insights in T-cell acute lymphoblastic leukemia. Exp Hematol. 43(8):625–639. 26123366 10.1016/j.exphem.2015.05.017

[6] Van Vlierberghe P, et al 2013. Prognostic relevance of integrated genetic profiling in adult T-cell acute lymphoblastic leukemia. Blood. 122(1):74–82. 23687089 10.1182/blood-2013-03-491092 PMC3701905

[7] Gianni F, Belver L, Ferrando A. 2020. The genetics and mechanisms of T-cell acute lymphoblastic leukemia. Cold Spring Harb Perspect Med. 10(3):a035246. 31570389 10.1101/cshperspect.a035246 PMC7050584

[8] Salmeron-Villalobos J, et al 2022. Diverse mutations and structural variations contribute to Notch signaling deregulation in paediatric T-cell lymphoblastic lymphoma. Pediatr Blood Cancer. 69(11):e29926. 36000950 10.1002/pbc.29926

[9] Asnafi V, et al 2009. NOTCH1/FBXW7 mutation identifies a large subgroup with favorable outcome in adult T-cell acute lymphoblastic leukemia (T-ALL): a Group for Research on Adult Acute Lymphoblastic Leukemia (GRAALL) study. Blood. 113(17):3918–3924. 19109228 10.1182/blood-2008-10-184069

[10] Baldus CD, et al 2009. Prognostic implications of NOTCH1 and FBXW7 mutations in adult acute T-lymphoblastic leukemia. Haematologica. 94(10):1383–1390. 19794083 10.3324/haematol.2008.005272 PMC2754954

[11] Van Vlierberghe P, Ferrando A. 2012. The molecular basis of T cell acute lymphoblastic leukemia. J Clin Invest. 122(10):3398–3406. 23023710 10.1172/JCI61269 PMC3461904

[12] Martelli AM, et al 2012. PI3K/AKT/mTORC1 and MEK/ERK signaling in T-cell acute lymphoblastic leukemia: new options for targeted therapy. Adv Biol Regul. 52(1):214–227. 21983557 10.1016/j.advenzreg.2011.09.019

[13] Taylor J, Xiao W, Abdel-Wahab O. 2017. Diagnosis and classification of hematologic malignancies on the basis of genetics. Blood. 130(4):410–423. 28600336 10.1182/blood-2017-02-734541 PMC5533199

[14] Iacobucci I, Mullighan CG. 2017. Genetic basis of acute lymphoblastic leukemia. J Clin Oncol. 35(9):975–983. 28297628 10.1200/JCO.2016.70.7836 PMC5455679

[15] Alaggio R, et al 2022. The 5th edition of the World Health Organization classification of haematolymphoid tumours: lymphoid neoplasms. Leukemia. 36(7):1720–1748. 35732829 10.1038/s41375-022-01620-2 PMC9214472

[16] Coustan-Smith E, et al 2009. Early T-cell precursor leukaemia: a subtype of very high-risk acute lymphoblastic leukaemia. Lancet Oncol. 10(2):147–156. 19147408 10.1016/S1470-2045(08)70314-0 PMC2840241

[17] Bene MC, et al 1995. Proposals for the immunological classification of acute leukemias. European Group for the Immunological Characterization of Leukemias (EGIL). Leukemia. 9(10):1783–1786. 7564526

[18] Hefazi M, Litzow MR. 2018. Recent advances in the biology and treatment of T cell acute lymphoblastic leukemia. Curr Hematol Malig Rep. 13(4):265–274. 29948644 10.1007/s11899-018-0455-9

[19] Chopra A, et al 2014. Immunophenotypic analysis of T-acute lymphoblastic leukemia. A CD5-based ETP-ALL perspective of non-ETP T-ALL. Eur J Haematol. 92(3):211–218. 24329989 10.1111/ejh.12238

[20] Das N, et al 2022. Protocol for ICiCLe-ALL-14 (InPOG-ALL-15-01): a prospective, risk stratified, randomised, multicentre, open label, controlled therapeutic trial for newly diagnosed childhood acute lymphoblastic leukaemia in India. Trials. 23(1):102. 35101099 10.1186/s13063-022-06033-1 PMC8805436

[21] Reiter A, et al 2000. Intensive ALL-type therapy without local radiotherapy provides a 90% event-free survival for children with T-cell lymphoblastic lymphoma: a BFM group report. Blood. 95(2):416–421. 10627444

[22] Magrath I, et al 2005. Treatment of acute lymphoblastic leukaemia in countries with limited resources; lessons from use of a single protocol in India over a twenty year period [corrected]. Eur J Cancer. 41(11):1570–1583. 16026693 10.1016/j.ejca.2004.11.004

[23] Lee BJ, et al 2023. HyperCVAD versus pegaspargase-containing regimens for Hispanic adults with newly diagnosed B-cell acute lymphoblastic leukemia. Eur J Haematol. 10.1111/ejh.14125. PMC12577190 37933194

[24] Haferlach C, et al 2012. Gene expression of BAALC, CDKN1B, ERG, and MN1 adds independent prognostic information to cytogenetics and molecular mutations in adult acute myeloid leukemia. Genes Chromosomes Cancer. 51(3):257–265. 22072540 10.1002/gcc.20950

[25] Heesch S, et al 2010. BAALC-associated gene expression profiles define IGFBP7 as a novel molecular marker in acute leukemia. Leukemia. 24(8):1429–1436. 20535151 10.1038/leu.2010.130

[26] Yin S, Dou J, Yang G, Chen F. 2019. Long non-coding RNA *XIST* expression as a prognostic factor in human cancers: a meta-analysis. Int J Biol Markers. 34(4):327–333. 31566056 10.1177/1724600819873010

[27] Trimarchi T, et al 2014. Genome-wide mapping and characterization of Notch-regulated long noncoding RNAs in acute leukemia. Cell. 158(3):593–606. 25083870 10.1016/j.cell.2014.05.049 PMC4131209

[28] Chen B, et al 2018. Identification of fusion genes and characterization of transcriptome features in T-cell acute lymphoblastic leukemia. Proc Natl Acad Sci U S A. 115(2):373–378. 29279377 10.1073/pnas.1717125115 PMC5777070

[29] Colomer-Lahiguera S, et al 2017. MEF2C-dysregulated pediatric T-cell acute lymphoblastic leukemia is associated with CDKN1B deletions and a poor response to glucocorticoid therapy. Leuk Lymphoma. 58(12):2895–2904. 28482719 10.1080/10428194.2017.1312383

[30] Kawashima-Goto S, et al 2015. BCL2 inhibitor (ABT-737): a restorer of prednisolone sensitivity in early T-cell precursor-acute lymphoblastic leukemia with high MEF2C expression? PLoS One. 10(7):e0132926. 26172269 10.1371/journal.pone.0132926 PMC4501565

[31] Zuurbier L, et al 2014. Immature MEF2C-dysregulated T-cell leukemia patients have an early T-cell precursor acute lymphoblastic leukemia gene signature and typically have non-rearranged T-cell receptors. Haematologica. 99(1):94–102. 23975177 10.3324/haematol.2013.090233 PMC4007923

[32] Homminga I, et al 2011. Integrated transcript and genome analyses reveal NKX2-1 and MEF2C as potential oncogenes in T cell acute lymphoblastic leukemia. Cancer Cell. 19(4):484–497. 21481790 10.1016/j.ccr.2011.02.008

[33] Nagel S, et al 2012. Transcriptional deregulation of homeobox gene ZHX2 in Hodgkin lymphoma. Leuk Res. 36(5):646–655. 22078940 10.1016/j.leukres.2011.10.019

[34] Nagel S, et al 2008. MEF2C is activated by multiple mechanisms in a subset of T-acute lymphoblastic leukemia cell lines. Leukemia. 22(3):600–607. 18079734 10.1038/sj.leu.2405067

[35] La Starza R, et al 2016. Deletions of the long arm of chromosome 5 define subgroups of T-cell acute lymphoblastic leukemia. Haematologica. 101(8):951–958. 27151989 10.3324/haematol.2016.143875 PMC4967574

[36] Laszlo GS, et al 2015. High expression of myocyte enhancer factor 2C (MEF2C) is associated with adverse-risk features and poor outcome in pediatric acute myeloid leukemia: a report from the Children's Oncology Group. J Hematol Oncol. 8:115. 26487643 10.1186/s13045-015-0215-4 PMC4618184

[37] Singh J, et al 2020. MEF2C expression, but not absence of bi-allelic deletion of TCR gamma chains (ABD), is a predictor of patient outcome in Indian T-acute lymphoblastic leukemia. Am J Blood Res. 10(5):294–304. 33224573 PMC7675123

[38] Heesch S, et al 2011. Expression of IGFBP7 in acute leukemia is regulated by DNA methylation. Cancer Sci. 102(1):253–259. 21040219 10.1111/j.1349-7006.2010.01760.x

[39] Neumann M, et al 2012. Clinical and molecular characterization of early T-cell precursor leukemia: a high-risk subgroup in adult T-ALL with a high frequency of FLT3 mutations. Blood Cancer J. 2(1):e55. 22829239 10.1038/bcj.2011.49 PMC3270253

[40] Baldus CD, et al 2007. Low ERG and BAALC expression identifies a new subgroup of adult acute T-lymphoblastic leukemia with a highly favorable outcome. J Clin Oncol. 25(24):3739–3745. 17646667 10.1200/JCO.2007.11.5253

[41] Ben Abdelali R, et al 2011. Pediatric-inspired intensified therapy of adult T-ALL reveals the favorable outcome of NOTCH1/FBXW7 mutations, but not of low ERG/BAALC expression: a GRAALL study. Blood. 118(19):5099–5107. 21835957 10.1182/blood-2011-02-334219

[42] Jackson JT, Nutt SL, McCormack MP. 2023. The haematopoietically-expressed homeobox transcription factor: roles in development, physiology and disease. Front Immunol. 14:1197490. 37398663 10.3389/fimmu.2023.1197490 PMC10313424

[43] Smith S, et al 2014. LIM domain only-2 (LMO2) induces T-cell leukemia by two distinct pathways. PLoS One. 9(1):e85883. 24465765 10.1371/journal.pone.0085883 PMC3897537

[44] Lukov GL, Rossi L, Souroullas GP, Mao R, Goodell MA. 2011. The expansion of T-cells and hematopoietic progenitors as a result of overexpression of the lymphoblastic leukemia gene, Lyl1 can support leukemia formation. Leuk Res. 35(3):405–412. 20705338 10.1016/j.leukres.2010.07.023 PMC2980862

[45] Zhong Y, Jiang L, Hiai H, Toyokuni S, Yamada Y. 2007. Overexpression of a transcription factor LYL1 induces T- and B-cell lymphoma in mice. Oncogene. 26(48):6937–6947. 17486074 10.1038/sj.onc.1210494

[46] Wang W, et al 2022. A comprehensive analysis of LMO2 pathogenic regulatory profile during T-lineage development and leukemic transformation. Oncogene. 41(34):4079–4090. 35851847 10.1038/s41388-022-02414-7

[47] McCormack MP, et al 2013. Requirement for Lyl1 in a model of Lmo2-driven early T-cell precursor ALL. Blood. 122(12):2093–2103. 23926305 10.1182/blood-2012-09-458570

[48] Fang F, et al 2022. Super-enhancer profiling identifies novel critical and targetable cancer survival gene LYL1 in pediatric acute myeloid leukemia. J Exp Clin Cancer Res. 41(1):225. 35842703 10.1186/s13046-022-02428-9 PMC9288051

[49] Latchmansingh KA, et al 2022. LMO2 expression is frequent in T-lymphoblastic leukemia and correlates with survival, regardless of T-cell stage. Mod Pathol. 35(9):1220–1226. 35322192 10.1038/s41379-022-01063-1 PMC9427670

[50] Malumbres R, et al 2011. LMO2 expression reflects the different stages of blast maturation and genetic features in B-cell acute lymphoblastic leukemia and predicts clinical outcome. Haematologica. 96(7):980–986. 21459790 10.3324/haematol.2011.040568 PMC3128216

[51] Ferrando AA, et al 2002. Gene expression signatures define novel oncogenic pathways in T cell acute lymphoblastic leukemia. Cancer Cell. 1(1):75–87. 12086890 10.1016/s1535-6108(02)00018-1

[52] Neumann M, et al 2013. FLT3 mutations in early T-cell precursor ALL characterize a stem cell like leukemia and imply the clinical use of tyrosine kinase inhibitors. PLoS One. 8(1):e53190. 23359050 10.1371/journal.pone.0053190 PMC3554732

[53] Neumann M, et al 2014. FAT1 expression and mutations in adult acute lymphoblastic leukemia. Blood Cancer J. 4(6):e224. 24972153 10.1038/bcj.2014.44 PMC4080215

[54] Zhang J, et al 2012. The genetic basis of early T-cell precursor acute lymphoblastic leukaemia. Nature. 481(7380):157–163. 22237106 10.1038/nature10725 PMC3267575

[55] Danis E, et al 2016. Ezh2 controls an early hematopoietic program and growth and survival signaling in early T cell precursor acute lymphoblastic leukemia. Cell Rep. 14(8):1953–1965. 26904942 10.1016/j.celrep.2016.01.064 PMC4790111

[56] Mar BG, et al 2014. Mutations in epigenetic regulators including SETD2 are gained during relapse in paediatric acute lymphoblastic leukaemia. Nat Commun. 5:3469. 24662245 10.1038/ncomms4469 PMC4016990

[57] Zhu X, et al 2014. Identification of functional cooperative mutations of SETD2 in human acute leukemia. Nat Genet. 46(3):287–293. 24509477 10.1038/ng.2894 PMC4440318

[58] Wang S, et al 2019. Genetic polymorphisms of histone methyltransferase SETD2 predicts prognosis and chemotherapy response in Chinese acute myeloid leukemia patients. J Transl Med. 17(1):101. 30922329 10.1186/s12967-019-1848-9 PMC6437967

[59] Ait-Si-Ali S, et al 1998. Histone acetyltransferase activity of CBP is controlled by cycle-dependent kinases and oncoprotein E1A. Nature. 396(6707):184–186. 9823900 10.1038/24190

[60] Liu X, et al 2008. The structural basis of protein acetylation by the p300/CBP transcriptional coactivator. Nature. 451(7180):846–850. 18273021 10.1038/nature06546

[61] Iyer NG, Ozdag H, Caldas C. 2004. P300/CBP and cancer. Oncogene. 23(24):4225–4231. 15156177 10.1038/sj.onc.1207118

[62] Qian M, et al 2017. Whole-transcriptome sequencing identifies a distinct subtype of acute lymphoblastic leukemia with predominant genomic abnormalities of EP300 and CREBBP. Genome Res. 27(2):185–195. 27903646 10.1101/gr.209163.116 PMC5287225

[63] Vo N, Goodman RH. 2001. CREB-binding protein and p300 in transcriptional regulation. J Biol Chem. 276(17):13505–13508. 11279224 10.1074/jbc.R000025200

[64] Bernt KM, et al 2011. MLL-rearranged leukemia is dependent on aberrant H3K79 methylation by DOT1L. Cancer Cell. 20(1):66–78. 21741597 10.1016/j.ccr.2011.06.010 PMC3329803

[65] Zhang Y, Kutateladze TG. 2019. Methylation of histone H3K79 by Dot1L requires multiple contacts with the ubiquitinated nucleosome. Mol Cell. 74(5):862–863. 31173720 10.1016/j.molcel.2019.05.013

[66] McGinty RK, Kim J, Chatterjee C, Roeder RG, Muir TW. 2008. Chemically ubiquitylated histone H2B stimulates hDot1L-mediated intranucleosomal methylation. Nature. 453(7196):812–816. 18449190 10.1038/nature06906 PMC3774535

[67] Jang S, et al 2019. Structural basis of recognition and destabilization of the histone H2B ubiquitinated nucleosome by the DOT1L histone H3 Lys79 methyltransferase. Genes Dev. 33(11–12):620–625. 30923167 10.1101/gad.323790.118 PMC6546062

[68] Valencia-Sanchez MI, et al 2019. Structural basis of Dot1L stimulation by histone H2B lysine 120 ubiquitination. Mol Cell. 74(5):1010–1019.e6. 30981630 10.1016/j.molcel.2019.03.029 PMC7009778

[69] Yousefi H, et al 2022. Long non-coding RNA signatures and related signaling pathway in T-cell acute lymphoblastic leukemia. Clin Transl Oncol. 24(11):2081–2089. 35852681 10.1007/s12094-022-02886-9

[70] Correia NC, Girio A, Antunes I, Martins LR, Barata JT. 2014. The multiple layers of non-genetic regulation of PTEN tumour suppressor activity. Eur J Cancer. 50(1):216–225. 24054978 10.1016/j.ejca.2013.08.017

[71] Luo H, et al 2019. HOTTIP lncRNA promotes hematopoietic stem cell self-renewal leading to AML-like disease in mice. Cancer Cell. 36(6):645–659.e8. 31786140 10.1016/j.ccell.2019.10.011 PMC6917035

[72] Yoshimoto R, Mayeda A, Yoshida M, Nakagawa S. 2016. *MALAT1* long non-coding RNA in cancer. Biochim Biophys Acta. 1859(1):192–199. 26434412 10.1016/j.bbagrm.2015.09.012

[73] Liu J, Peng WX, Mo YY, Luo D. 2017. *MALAT1*-mediated tumorigenesis. Front Biosci (Landmark Ed). 22(1):66–80. 27814602 10.2741/4472

[74] Sun W, Yang Y, Xu C, Guo J. 2017. Regulatory mechanisms of long noncoding RNAs on gene expression in cancers. Cancer Genet. 216–217:105–110. 10.1016/j.cancergen.2017.06.003 29025584

[75] Wei Y, Niu B. 2015. Role of *MALAT1* as a prognostic factor for survival in various cancers: a systematic review of the literature with meta-analysis. Dis Markers. 2015:164635. 26420912 10.1155/2015/164635 PMC4572489

[76] Zhao M, et al 2018. *MALAT1*: a long non-coding RNA highly associated with human cancers. Oncol Lett. 16(1):19–26. 29928382 10.3892/ol.2018.8613 PMC6006327

[77] Li ZX, et al 2018. *MALAT1*: a potential biomarker in cancer. Cancer Manag Res. 10:6757–6768. 30584369 10.2147/CMAR.S169406 PMC6289210

[78] Gianfelici V, et al 2016. RNA sequencing unravels the genetics of refractory/relapsed T-cell acute lymphoblastic leukemia. Prognostic and therapeutic implications. Haematologica. 101(8):941–950. 27151993 10.3324/haematol.2015.139410 PMC4967573

[79] Lahera A, et al 2023. Comprehensive characterization of a novel, oncogenic and targetable SEPTIN6::ABL2 fusion in T-ALL. Br J Haematol. 202(3):693–698. 37264982 10.1111/bjh.18901

[80] Maciel ALT, Wolch K, Emerenciano M, Mansur MB. 2022. CRLF2 overexpression defines an immature-like subgroup which is rescued through restoration of the PRC2 function in T-cell precursor acute lymphoblastic leukemia. Genes Chromosomes Cancer. 61(7):437–442. 35253299 10.1002/gcc.23036

[81] Grioni A, et al 2019. A simple RNA target capture NGS strategy for fusion genes assessment in the diagnostics of pediatric B-cell acute lymphoblastic leukemia. Hemasphere. 3(3):e250. 31723839 10.1097/HS9.0000000000000250 PMC6746019

[82] Marincevic-Zuniga Y, et al 2017. Transcriptome sequencing in pediatric acute lymphoblastic leukemia identifies fusion genes associated with distinct DNA methylation profiles. J Hematol Oncol. 10(1):148. 28806978 10.1186/s13045-017-0515-y PMC5557398

[83] Zou P, et al 2022. The long-term outcome and risk factors for precursor B cell acute lymphoblastic leukemia without specific fusion genes in Chinese children: experiences from multiple centers. Bosn J Basic Med Sci. 22(2):238–246. 34392828 10.17305/bjbms.2021.5879 PMC8977091

[84] Györffy B, et al 2010. An online survival analysis tool to rapidly assess the effect of 22,277 genes on breast cancer prognosis using microarray data of 1,809 patients. Breast Cancer Res Treat. 123(3):725–731. 20020197 10.1007/s10549-009-0674-9

