# Supplementary material for: Decoding the genetic symphony: Profiling protein-coding and long noncoding RNA expression in T-acute lymphoblastic leukemia for clinical insights
Source: PNAS Nexus. 2024 Jan 12;3(2):pgae011. doi: 10.1093/pnasnexus/pgae011 (PMC10847906; doi:10.1093/pnasnexus/pgae011)
Supplement: pgae011_Supplementary_Data [file pgae011_supplementary_data.zip › PNASNEXUS-PNASNEXUS-2023-00530R-s03.docx]

**Supplemental Methods**

***RNA* sequencing**

PBMC isolation performed by density gradient method using Histopaque (Sigma-Aldrich, USA) and 1 ml TRIzol (Thermo Fisher Scientific, USA) for per one million cells was added in each vial immediately and mixed by syringing.TRIzol mixed samples were stored at -80°C for RNA isolation. RNA was isolated by TRIzol (Guanidinium thiocyanate-phenol-chloroform extraction) method (Rio Dc Fau - Ares, Ares M Jr Fau - Hannon et al.). RNA was dissolved in DEPC-treated water. RNA quantity was checked by nanodrop and Qubit RNA broad range assay kit on dye-based qubit spectrophotometer (Thermo Fisher Scientific, U.S.).Agilent 2100 Bioanalyzer (Agilent Technologies, USA) with RNA chips was used to check the RNA Integrity Score (RIN).We selected samples with ≥ 7 RIN score for RNA sequencing library preparation.

**RNA seq library preparation**

Sample preparation for sequencing was carried out using strand specific Truseq RNA sample preparation kit (Illumina, San Diego, California, U.S.) as per supplier’s instructions and 8.0 picomol of the pooled library was sequenced on the Illumina HiSeq2000 system (Figure 1).


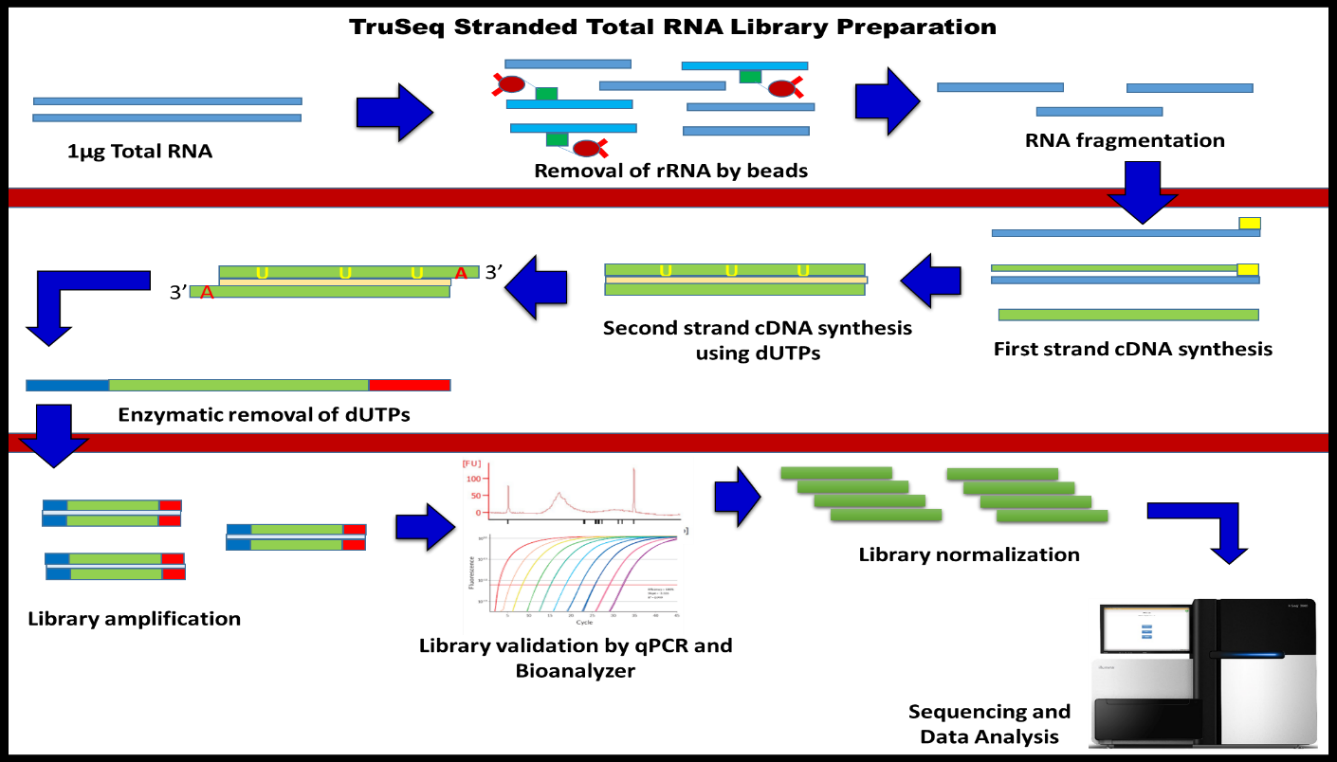


**Figure 1: Schematic diagram of library preparation by using Illumina TruSeq Stranded Total RNA library preparation kit**

**Data collection and Quality analysis**

A total of 480 GB raw data was collected in the form of “. fastq” file which contained 50 million average reads perfile.The quality of reads was checked using FastQC v0.11.8(Andrews 2010)and trimming were implemented using Trimmomatic PE (Bolger, Lohse et al. 2014). We used a quality filter of Phred quality cut-off 30 and ahead crop of 15. The overall percentage of reads surviving after Trimmomatic was 70.83% to 86.36% (Figure 2 and 3).


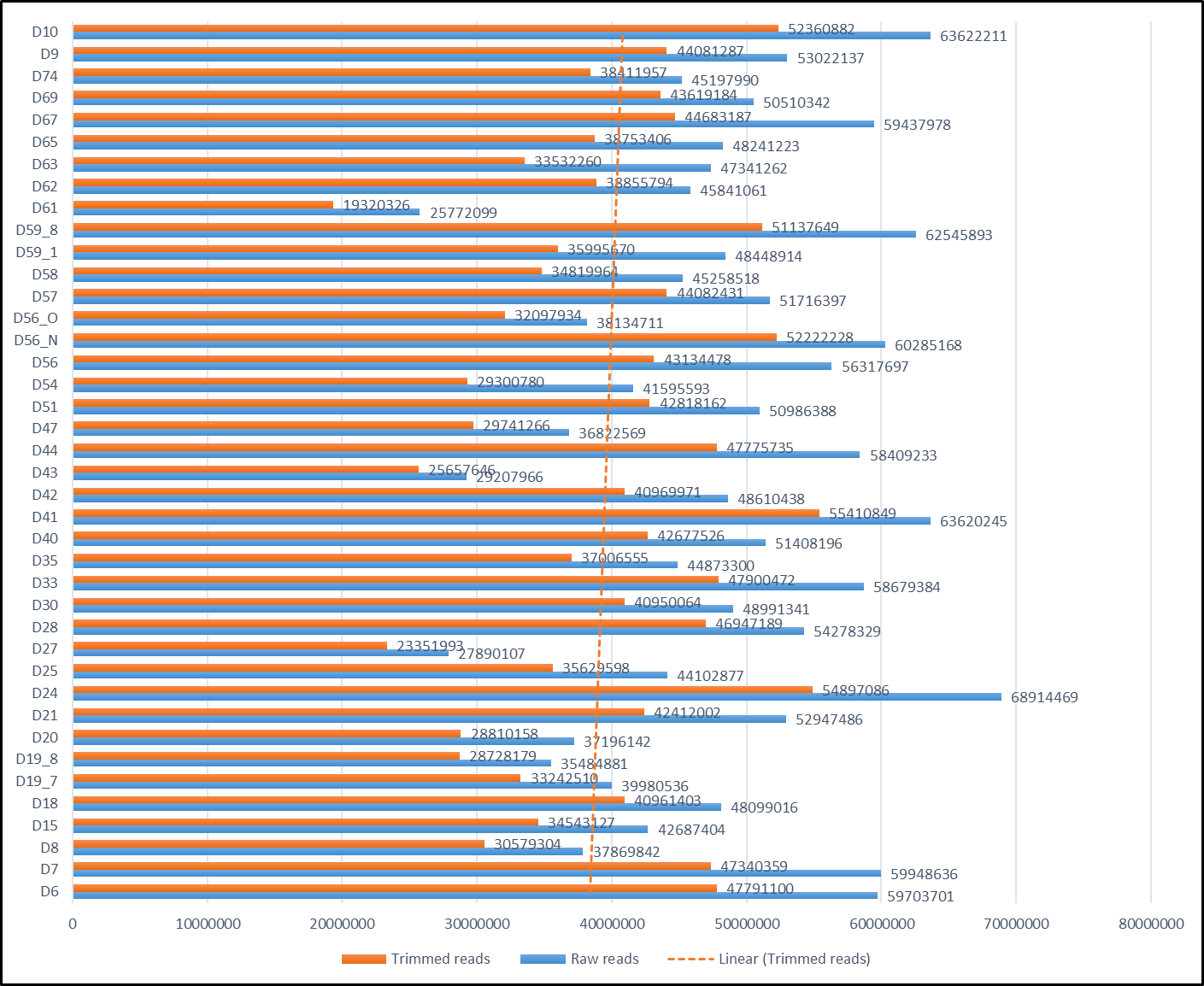


**Figure2 : Graphical representation of raw and trimmed reads**


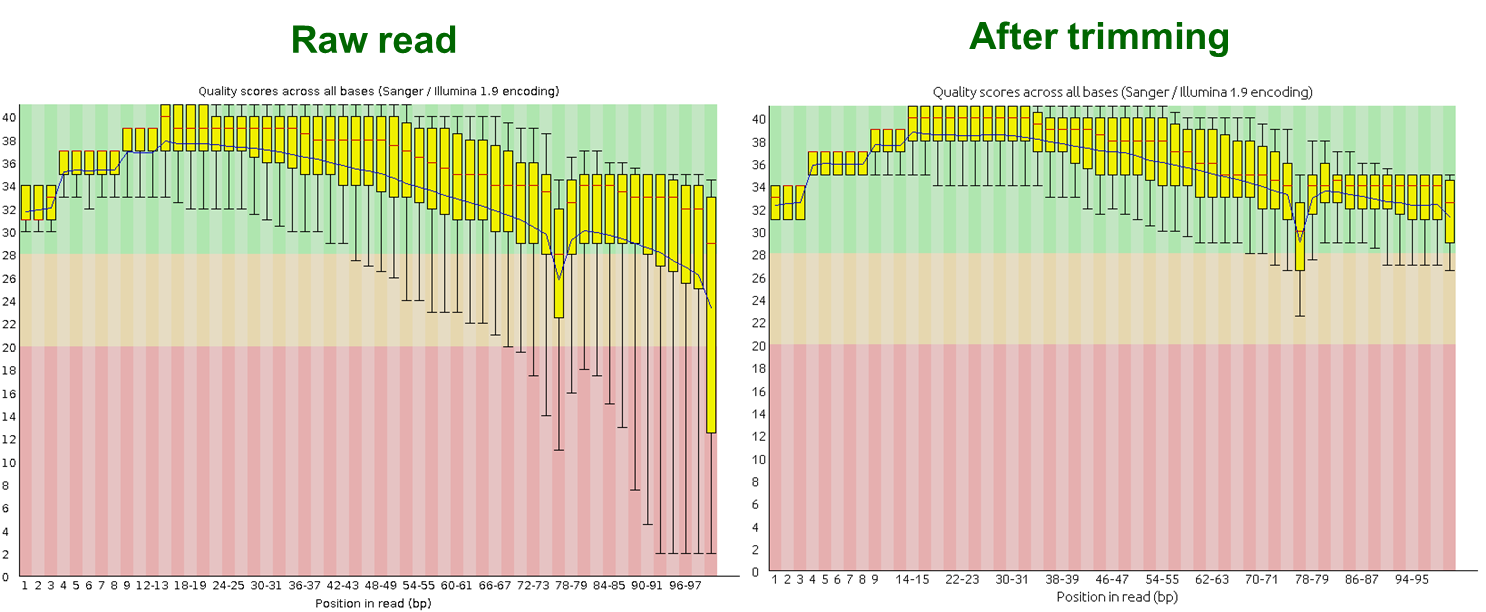
**Figure 3: Quality check of sample D6 raw reads and after running Trimmomatic software by FastQC.**


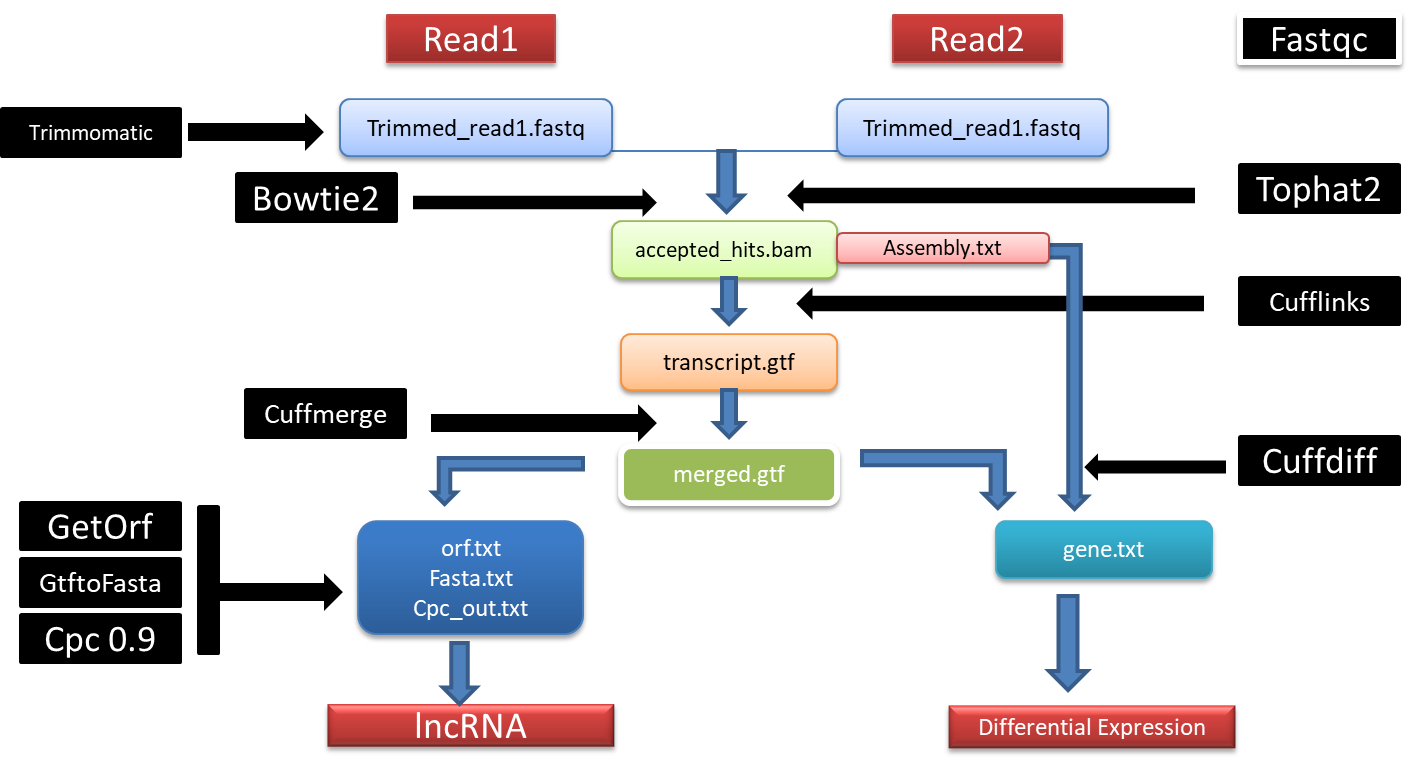


**Figure 4: Data analysis pipeline for differentially expressed coding genes and long non-coding RNA**

**Mapping and alignment of reads**

A brief analysis pipeline is described in Figure 4. The human reference genome, hg38, was used for mapping and alignment, downloaded from UCSC genome browser gateway. Reads were aligned to the indexed hg38 genome using splice junction mapper: Tophat2.1.1 (Trapnell, Pachter et al. 2009, Kim, Pertea et al. 2013). Total Avg 82.275 % of reads were aligned to the human genome hg38.

**Transcriptome assembly and merging**

Cufflinks 2.2.1 (Trapnell, Williams et al. 2010) was used to assemble the transcripts, estimate transcripts’ abundance and calculate differential gene expression and regulation. These assemblies .gtf files are then merged into a single file by using the Cuffmerge utility. Finally, the merged files were stored in merged.gtf format.

**Differential gene expression and functional analysis by the supervised approach**

Differential expression of the assembled transcripts across 3 types of T-ALL (immature, cortical and mature T-ALL based on immunophenotypic features) were analyzed using module Cuffdiff from the Cufflinks package (Trapnell, Williams et al. 2010). Samples of cortical subgroup were also analyzed in sCD3 positive and negative conditions by Cuffdiff.The Fragment per Kilobase Exon per Million Reads (FPKM) measure was used to quantify the expression of the transcript. For better understanding, these FPKM values were converted into fold changes. We filtered in all genes that were ≥ 2-fold changes as significantly important genes for a given condition.

**Principal component analysis**

The output of cufflink was used in cuffnorm to produce several output files that contained normalized fragment counts and expression levels at the level of transcripts, primary transcripts, and genes. In this approach, 35 patients and transcriptome from normal thymus was used. We found 58,224 genes that were expressed in all 35 patient samples including normal thymus. In cuffnorm output, we removed all transcripts with < 2 FPKM scores and found 31,694 genes that were ≥2 FPKM. All samples were used as an input feature to perform principal component analysis (PCA) on BioVinciVersion: 1.1.5, r20181005 (Bioturing, USA). We found maximum principal component equal to the samples and only 6 were statistically significant. To apply an unsupervised approach all 6 principal components were analyzed, and a graph was plotted. In this graph, all samples were clustered into 3 major clusters. After this, all samples were compared with immunophenotypic phenotypes of samples.

**Differential expression analysis of non-coding RNA**

**Known lncRNA**

The long noncoding RNAs were identified and characterized by “Biomart” (https://www.ensembl.org/biomart). Later their differential gene expression analysis was performed. All transcripts which had more than 1 FPKM were used to calculate fold changes. Transcripts those were more than 2-FC and less than -2 FC was selected as a putative transcript in a given condition. The results were plotted on a heatmap using multiple experiment viewer (MeV, http://mev.tm4.org). Further, their gene function co-expression network analysis was performed.

**Novel lncRNA**

**Annotation of novel long non-coding RNA**

For the identification of novel lncRNAs, the resulting output of cuffmerge was used to analyze lncRNA in the data. The transcripts with a nucleotide length greater than 200 were filtered. These transcripts were further classified as coding and non-coding by evaluating the length of their open reading frames (ORF) and coding potentials using two independent approaches, ORF finder and CPC calculator (Kong, Zhang et al. 2007).The ORFs of all the transcripts were predicted using getORF utility of EMBOSS toolkit. Transcripts with an ORF of length greater than or equal to 30 amino acids (aa) were removed to remove all protein-coding and micro peptide coding transcripts, thus giving more stringent criteria to predict only noncoding transcripts as lncRNAs. The coding potential of the filtered transcripts was further calculated using the Coding Potential Calculator (CPC). A CPC scores less than zero indicates a low coding potential of a transcript while of less than zero indicates a low coding potential of a transcript while a positive CPC score indicates a high coding potential of a transcript. The transcripts with a score less than 0 were retained and the final set represented the long non-coding RNAs of zebrafish while the one with orf more than30 aa and CPC score of more than 1 were considered as protein-coding transcripts. The pipeline used in the analysis of novel lncRNA has been summarized in Figure 4.

Differential expression analysis of lncRNAs: Differential expression of the assembled transcripts across various types of T-ALL (immature, cortical and mature T-ALL) was analyzed using Cuffdiff. The FPKM measure was used to quantify the expression of lncRNAs. lncRNAs which have at least >5 FPKM in one type of T-ALL and at least a 2-fold difference between other conditions were prioritized for further validation.

**Identification of fusion transcripts**

The examination of fusion transcripts across all samples was conducted through the utilization of freely accessible online tool FusionCatcher (<https://doi.org/10.1101/011650>). The analysis involved the use of raw RNA-seq reads, which were neither trimmed nor mapped. FusionCatcher software was employed with specific parameters: **1.** Fusion breakpoints necessitated support from a minimum of 3 distinct covered reads and a minimum of 3 spanning reads. **2.** Fusion genes identified in healthy populations and controls were systematically excluded. **3.** Blacklisted fusion genes and promiscuous genes were rigorously filtered out. 4. Fusion genes reported in T-ALL, as well as those documented in the ChimerDB 4.0 (1) and Mitelman database (https://mitelmandatabase.isb-cgc.org/), were retained throughout the filtration process. This predictive tool identified a total of 789 fusion transcripts, categorized into Immature (390), Mature (93), and Cortical (306), encompassing both protein coding and non-coding genes. The breakpoints implicated in these fusions were situated in diverse genomic regions, including UTRs, intronic regions, intergenic regions, and exonic portions. To refine the findings, false positive fusions were meticulously excluded based on criteria established in the previous study, resulting in the identification of 19 fusion transcripts across 23 T-ALL samples.
