# Supplementary material for: Decoding the genetic symphony: Profiling protein-coding and long noncoding RNA expression in T-acute lymphoblastic leukemia for clinical insights
Source: PNAS Nexus. 2024 Jan 12;3(2):pgae011. doi: 10.1093/pnasnexus/pgae011 (PMC10847906; doi:10.1093/pnasnexus/pgae011)
Supplement: pgae011_Supplementary_Data [file pgae011_supplementary_data.zip › PNASNEXUS-PNASNEXUS-2023-00530R-s04.docx]

**Supplementary Tables**

**Supplementary Table 1A: List of upregulated genes in immature T-ALL**

| **Ensembl_ID** | **Genes** | **Log_2_fold change in T-ALL subtypes** | | |
| --- | --- | --- | --- | --- |
|  |  | **Immature** | **Cortical** | **Mature** |
| ENSG00000085563 | *ABCB1* | 2.915696175 | 0 | -1.23053 |
| ENSG00000100439 | *ABHD4* | 2.035455138 | 0 | -0.27633 |
| ENSG00000225792 | *AC004540.4* | 2.248856816 | -0.46123 | 0 |
| ENSG00000243004 | *AC005062.2* | 2.614275901 | 0 | 0 |
| ENSG00000231916 | *AC006033.22* | 2.715155869 | 0 | 0 |
| ENSG00000213083 | *AC010731.6* | 4.071221777 | 0 | 0 |
| ENSG00000233766 | *AC098617.1* | 5.075344826 | 0 | 0 |
| ENSG00000107796 | *ACTA2* | 2.790388863 | 0 | -2.54819 |
| ENSG00000163017 | *ACTG2* | 5.018605592 | -1.43309 | 0 |
| ENSG00000132744 | *ACY3* | 3.459731872 | 0 | -1.28968 |
| ENSG00000140873 | *ADAMTS18* | 2.279992724 | -3.59108 | 0 |
| ENSG00000141433 | *ADCYAP1* | 2.04997848 | 0 | -2.4819 |
| ENSG00000112414 | *ADGRG6* | 4.048197749 | 0 | -1.96367 |
| ENSG00000111863 | *ADTRP* | 3.29114314 | 0 | -0.83665 |
| ENSG00000253981 | *ALG1L13P* | 4.396039189 | 0 | 0 |
| ENSG00000091879 | *ANGPT2* | 5.6078003 | 0 | 0 |
| ENSG00000151150 | *ANK3* | 2.508519408 | 0 | -1.17461 |
| ENSG00000230539 | *AOAH-IT1* | 2.059049729 | 0 | -0.72564 |
| ENSG00000233236 | *AP001171.1* | 2.658115904 | 0 | 0 |
| ENSG00000234906 | *APOC2* | 5.505666649 | 0 | 0 |
| ENSG00000142192 | *APP* | 2.083374019 | 0 | -0.94985 |
| ENSG00000169083 | *AR* | 5.398060117 | -3.94799 | 0 |
| ENSG00000165801 | *ARHGEF40* | 2.028544584 | 0 | -1.64241 |
| ENSG00000166183 | *ASPG* | 2.226467188 | -2.02928 | 0 |
| ENSG00000104043 | *ATP8B4* | 2.066398527 | 0 | -2.28386 |
| ENSG00000177191 | *B3GNT8* | 2.144646949 | 0 | 0 |
| ENSG00000164929 | *BAALC* | 4.047149873 | 0 | 0 |
| ENSG00000284128 | *BCRP3* | 2.190539306 | 0 | -0.32385 |
| ENSG00000095585 | *BLNK* | 2.184989907 | 0 | -1.21065 |
| ENSG00000236323 | *BMP6P1* | 3.050733689 | -0.56389 | 0 |
| ENSG00000186265 | *BTLA* | 2.567973319 | 0 | -2.24232 |
| ENSG00000204161 | *C10orf128* | 2.78479347 | 0 | -3.75211 |
| ENSG00000110665 | *C11orf21* | 2.171075124 | -0.22089 | 0 |
| ENSG00000172247 | *C1QTNF4* | 2.392731202 | -1.32202 | 0 |
| ENSG00000180539 | *C9orf139* | 2.586098481 | -0.0134 | 0 |
| ENSG00000256812 | *CAPNS2* | 2.930167727 | -0.09778 | 0 |
| ENSG00000204682 | *CASC10* | 2.286130354 | 0 | -2.43507 |
| ENSG00000108702 | *CCL1* | 4.630406589 | 0 | -2.39899 |
| ENSG00000133101 | *CCNA1* | 2.441184286 | 0 | 0 |
| ENSG00000204936 | *CD177* | 2.053477254 | 0 | -0.13401 |
| ENSG00000134061 | *CD180* | 3.237189881 | 0 | -1.0072 |
| ENSG00000105383 | *CD33* | 2.062764008 | 0 | -0.71369 |
| ENSG00000135218 | *CD36* | 2.014080848 | 0 | -1.95368 |
| ENSG00000113100 | *CDH9* | 7.471533563 | -1.75216 | 0 |
| ENSG00000230666 | *CEACAM22P* | 2.918434418 | -2.73654 | 0 |
| ENSG00000101938 | *CHRDL1* | 3.322688669 | 0 | -4.78354 |
| ENSG00000179583 | *CIITA* | 2.881182589 | 0 | -0.57 |
| ENSG00000256660 | *CLEC12B* | 2.309260464 | 0 | 0 |
| ENSG00000176435 | *CLEC14A* | 2.602557001 | -4.90337 | 0 |
| ENSG00000184293 | *CLECL1* | 6.991126722 | 0 | -1.7924 |
| ENSG00000155052 | *CNTNAP5* | 6.266332333 | -6.99281 | 0 |
| ENSG00000258551 | *CRAT37* | 3.01078969 | 0 | -0.84167 |
| ENSG00000145708 | *CRHBP* | 2.165741971 | -0.08222 | 0 |
| ENSG00000184371 | *CSF1* | 3.10203424 | 0 | 0 |
| ENSG00000126545 | *CSN1S1* | 2.927193385 | -2.10068 | 0 |
| ENSG00000175183 | *CSRP2* | 3.327443248 | 0 | -1.826 |
| ENSG00000267771 | *CTC-260E6.7* | 2.177836779 | 0 | 0 |
| ENSG00000269243 | *CTD-2231E14.8* | 2.223395062 | 0 | -2.20305 |
| ENSG00000250574 | *CTD-2275D10.2* | 2.19310109 | -0.35962 | 0 |
| ENSG00000188897 | *CTD-3088G3.8* | 2.654472303 | 0 | -0.74065 |
| ENSG00000170891 | *CYTL1* | 2.086502308 | 0 | 0 |
| ENSG00000276644 | *DACH1* | 2.149177705 | 0 | -0.882 |
| ENSG00000089876 | *DHX32* | 2.432443253 | -0.12617 | 0 |
| ENSG00000205683 | *DPF3* | 2.104078399 | 0 | -0.01825 |
| ENSG00000175497 | *DPP10* | 2.279709167 | -3.98821 | 0 |
| ENSG00000121570 | *DPPA4* | 2.1462129 | 0 | 0 |
| ENSG00000151914 | *DST* | 2.027295931 | 0 | -0.26812 |
| ENSG00000125821 | *DTD1* | 2.029216762 | 0 | -0.3792 |
| ENSG00000108861 | *DUSP3* | 2.093376395 | -0.05674 | 0 |
| ENSG00000231513 | *E2F6P4* | 2.725314428 | 0 | 0 |
| ENSG00000183690 | *EFHC2* | 2.567655232 | -0.53796 | 0 |
| ENSG00000106991 | *ENG* | 2.147756486 | 0 | -2.32808 |
| ENSG00000116106 | *EPHA4* | 2.035447298 | 0 | -0.84876 |
| ENSG00000244405 | *ETV5* | 2.532759486 | -0.09444 | 0 |
| ENSG00000167880 | *EVPL* | 3.365327243 | 0 | -1.06335 |
| ENSG00000185614 | *FAM212A* | 2.683576413 | 0 | -0.57129 |
| ENSG00000112773 | *FAM46A* | 2.845744624 | 0 | -0.32782 |
| ENSG00000179639 | *FCER1A* | 5.673169695 | 0 | -1.64912 |
| ENSG00000054598 | *FOXC1* | 2.42537423 | -0.30544 | 0 |
| ENSG00000176678 | *FOXL1* | 2.710392458 | 0 | 0 |
| ENSG00000188738 | *FSIP2* | 2.473560107 | 0 | 0 |
| ENSG00000148803 | *FUOM* | 2.314293418 | -0.08164 | 0 |
| ENSG00000175857 | *GAPT* | 3.888807483 | 0 | -0.02396 |
| ENSG00000171766 | *GATM* | 2.142438634 | 0 | -0.47685 |
| ENSG00000225492 | *GBP1P1* | 2.364782086 | 0 | 0 |
| ENSG00000162645 | *GBP2* | 2.049884344 | 0 | -2.61174 |
| ENSG00000152661 | *GJA1* | 8.725652904 | 0 | 0 |
| ENSG00000156689 | *GLYATL2* | 3.593132311 | 0 | 0 |
| ENSG00000127955 | *GNAI1* | 3.245071628 | 0 | 0 |
| ENSG00000166123 | *GPT2* | 2.632119695 | 0 | -0.35972 |
| ENSG00000196277 | *GRM7* | 2.374547126 | 0 | -2.69154 |
| ENSG00000152804 | *HHEX* | 2.499185075 | 0 | -1.34457 |
| ENSG00000196126 | *HLA-DRB1* | 3.098892093 | 0 | -0.32568 |
| ENSG00000198502 | *HLA-DRB5* | 3.59451695 | 0 | 0 |
| ENSG00000236946 | *HNRNPA1P70* | 2.073150773 | 0 | -1.94953 |
| ENSG00000171476 | *HOPX* | 5.056027771 | 0 | -0.87796 |
| ENSG00000243766 | *HOTTIP* | 9.117008651 | 0 | 0 |
| ENSG00000253293 | *HOXA10* | 2.137373366 | 0 | -2.51429 |
| ENSG00000240990 | *HOXA11-AS* | 5.353198426 | 0 | 0 |
| ENSG00000106031 | *HOXA13* | 8.711550341 | 0 | 0 |
| ENSG00000197576 | *HOXA4* | 4.04388059 | -1.65188 | 0 |
| ENSG00000106004 | *HOXA5* | 3.681519584 | -0.69626 | 0 |
| ENSG00000106006 | *HOXA6* | 3.44210011 | 0 | -1.16494 |
| ENSG00000078399 | *HOXA9* | 4.026380525 | 0 | -0.45339 |
| ENSG00000163106 | *HPGDS* | 2.829072299 | -0.14922 | 0 |
| ENSG00000196684 | *HSH2D* | 2.26478815 | 0 | -0.01921 |
| ENSG00000179097 | *HTR1F* | 2.765650205 | 0 | 0 |
| ENSG00000115738 | *ID2* | 2.083285231 | 0 | -2.20304 |
| ENSG00000163453 | *IGFBP7* | 2.666912006 | 0 | -0.51956 |
| ENSG00000240382 | *IGKV1-17* | 2.021902623 | 0 | 0 |
| ENSG00000243238 | *IGKV2-30* | 2.415948101 | 0 | 0 |
| ENSG00000206066 | *IGLL3P* | 3.489887195 | 0 | 0 |
| ENSG00000211642 | *IGLV10-54* | 3.128430096 | 0 | 0 |
| ENSG00000211639 | *IGLV4-60* | 2.693518262 | 0 | -0.10717 |
| ENSG00000211640 | *IGLV6-57* | 2.923249972 | 0 | 0 |
| ENSG00000253637 | *IGLVV-58* | 4.098062975 | 0 | 0 |
| ENSG00000136634 | *IL10* | 2.99035706 | 0 | -2.00098 |
| ENSG00000081985 | *IL12RB2* | 2.398114003 | 0 | -4.49714 |
| ENSG00000150782 | *IL18* | 2.974672092 | 0 | -1.91588 |
| ENSG00000115604 | *IL18R1* | 2.287718462 | 0 | -0.32205 |
| ENSG00000128604 | *IRF5* | 2.057945418 | 0 | -1.54015 |
| ENSG00000144668 | *ITGA9* | 2.638893935 | 0 | -0.16696 |
| ENSG00000233844 | *KCNQ5-IT1* | 2.38460423 | 0 | -1.29903 |
| ENSG00000122548 | *KIAA0087* | 5.784597553 | 0 | 0 |
| ENSG00000003096 | *KLHL13* | 2.18738994 | 0 | -2.71448 |
| ENSG00000185271 | *KLHL33* | 3.273934056 | 0 | -0.34025 |
| ENSG00000256797 | *KLRF2* | 5.865328037 | -1.14654 | 0 |
| ENSG00000187772 | *LIN28B* | 4.520255771 | 0 | 0 |
| ENSG00000242258 | *LINC00996* | 2.365456184 | -0.38849 | 0 |
| ENSG00000250337 | *LINC01021* | 5.128457788 | 0 | -3.90851 |
| ENSG00000249464 | *LINC01091* | 2.047199919 | 0 | -2.32975 |
| ENSG00000226673 | *LINC01108* | 4.434768081 | -1.06353 | 0 |
| ENSG00000233723 | *LINC01122* | 2.120343001 | 0 | -0.91758 |
| ENSG00000233776 | *LINC01251* | 2.010453902 | 0 | -0.9006 |
| ENSG00000204603 | *LINC01257* | 3.124112052 | 0 | 0 |
| ENSG00000280560 | *LINC01374* | 5.834921946 | 0 | 0 |
| ENSG00000231106 | *LINC01436* | 2.537319765 | 0 | -0.50182 |
| ENSG00000174482 | *LINGO2* | 5.006272026 | 0 | 0 |
| ENSG00000227710 | *LL22NC03-30E12.13* | 2.252712842 | 0 | -1.18164 |
| ENSG00000135363 | *LMO2* | 2.229610423 | 0 | -0.52773 |
| ENSG00000167210 | *LOXHD1* | 3.941758457 | -0.32468 | 0 |
| ENSG00000087253 | *LPCAT2* | 2.565898289 | 0 | -1.05944 |
| ENSG00000154237 | *LRRK1* | 2.316871541 | 0 | -4.9096 |
| ENSG00000062524 | *LTK* | 2.408775312 | 0 | -0.54212 |
| ENSG00000112799 | *LY86* | 2.830257532 | 0 | -1.19609 |
| ENSG00000254087 | *LYN* | 2.057457449 | 0 | -1.29631 |
| ENSG00000183742 | *MACC1* | 2.244909901 | 0 | -0.08192 |
| ENSG00000111885 | *MAN1A1* | 2.27034673 | 0 | -1.6065 |
| ENSG00000135525 | *MAP7* | 3.288661432 | 0 | -0.39762 |
| ENSG00000081189 | *MEF2C* | 4.446601537 | 0 | -1.64372 |
| ENSG00000248309 | *MEF2C-AS1* | 4.6451988 | 0 | 0 |
| ENSG00000143995 | *MEIS1* | 3.194308855 | 0 | -0.80915 |
| ENSG00000270069 | *MIR222HG* | 2.080962512 | 0 | -0.24007 |
| ENSG00000253522 | *MIR3142HG* | 2.010374864 | 0 | -1.71033 |
| ENSG00000284179 | *MIR7-1* | 2.09452357 | 0 | 0 |
| ENSG00000138722 | *MMRN1* | 2.427299298 | 0 | -2.09628 |
| ENSG00000169184 | *MN1* | 4.221733768 | -1.22263 | 0 |
| ENSG00000214787 | *MS4A4E* | 4.887575336 | 0 | 0 |
| ENSG00000255823 | *MTRNR2L8* | 2.401591146 | 0 | -2.77595 |
| ENSG00000205592 | *MUC19* | 3.85479086 | 0 | -3.0445 |
| ENSG00000198336 | *MYL4* | 3.51881759 | 0 | 0 |
| ENSG00000007944 | *MYLIP* | 2.197865769 | 0 | -0.38578 |
| ENSG00000133454 | *MYO18B* | 5.701112061 | -3.90402 | 0 |
| ENSG00000157483 | *MYO1E* | 2.051631237 | 0 | -0.86317 |
| ENSG00000196586 | *MYO6* | 4.105119008 | -1.19532 | 0 |
| ENSG00000149294 | *NCAM1* | 3.745164334 | 0 | -2.32716 |
| ENSG00000197168 | *NEK5* | 2.53612431 | 0 | -0.73049 |
| ENSG00000119408 | *NEK6* | 2.053217366 | 0 | -1.16514 |
| ENSG00000256331 | *NIFKP3* | 2.168690551 | 0 | -1.71485 |
| ENSG00000113389 | *NPR3* | 3.674058292 | 0 | -1.21822 |
| ENSG00000135318 | *NT5E* | 3.274639746 | 0 | -1.07463 |
| ENSG00000205978 | *NYNRIN* | 4.047078093 | -0.5345 | 0 |
| ENSG00000119900 | *OGFRL1* | 2.037456958 | 0 | -1.69841 |
| ENSG00000184221 | *OLIG1* | 2.746032783 | 0 | 0 |
| ENSG00000079482 | *OPHN1* | 4.164370219 | -0.74295 | 0 |
| ENSG00000224227 | *OR2L1P* | 2.210200621 | -0.05795 | 0 |
| ENSG00000168126 | *OR2W6P* | 2.786324216 | -1.19071 | 0 |
| ENSG00000180090 | *OR3A1* | 2.529895537 | 0 | -0.39341 |
| ENSG00000185345 | *PARK2* | 3.202644463 | 0 | -0.85082 |
| ENSG00000132849 | *PATJ* | 2.638441186 | -0.06162 | 0 |
| ENSG00000184588 | *PDE4B* | 3.33754888 | 0 | -1.5925 |
| ENSG00000145431 | *PDGFC* | 4.798551706 | 0 | 0 |
| ENSG00000163737 | *PF4* | 2.696080743 | 0 | 0 |
| ENSG00000213997 | *PGAM1P7* | 2.696733753 | 0 | 0 |
| ENSG00000112137 | *PHACTR1* | 2.457747429 | -0.22514 | 0 |
| ENSG00000229508 | *PHKG1P4* | 2.311755533 | 0 | 0 |
| ENSG00000154864 | *PIEZO2* | 2.578160726 | 0 | -2.41293 |
| ENSG00000166428 | *PLD4* | 3.628166167 | 0 | -0.39115 |
| ENSG00000100979 | *PLTP* | 4.210989178 | 0 | -0.23441 |
| ENSG00000124429 | *POF1B* | 2.859997474 | 0 | -2.51021 |
| ENSG00000163736 | *PPBP* | 2.050833679 | 0 | -2.77155 |
| ENSG00000007062 | *PROM1* | 3.711682551 | 0 | -1.96589 |
| ENSG00000135362 | *PRR5L* | 2.018564206 | 0 | -0.74778 |
| ENSG00000010438 | *PRSS3* | 2.66059105 | 0 | 0 |
| ENSG00000095303 | *PTGS1* | 3.405783347 | 0 | -1.21252 |
| ENSG00000276600 | *RAB7B* | 8.008634058 | 0 | 0 |
| ENSG00000132329 | *RAMP1* | 5.056182814 | 0 | 0 |
| ENSG00000201620 | *RNA5SP51* | 2.264528196 | 0 | 0 |
| ENSG00000141576 | *RNF157* | 2.749660544 | 0 | -0.9422 |
| ENSG00000199426 | *RNU1-108P* | 3.358279753 | 0 | 0 |
| ENSG00000185008 | *ROBO2* | 4.467610776 | 0 | 0 |
| ENSG00000145491 | *ROPN1L* | 2.434654926 | 0 | -1.41758 |
| ENSG00000205746 | *RP11-1212A22.1* | 2.107788204 | -0.70809 | 0 |
| ENSG00000254687 | *RP11-162D9.3* | 2.09475506 | -5.61857 | 0 |
| ENSG00000279311 | *RP11-170K4.2* | 3.063659287 | -0.12087 | 0 |
| ENSG00000249790 | *RP11-20D14.6* | 2.212535436 | 0 | -0.32531 |
| ENSG00000261997 | *RP11-212I21.4* | 3.276172601 | 0 | -1.87336 |
| ENSG00000116883 | *RP11-268J15.5* | 2.121927002 | 0 | -0.42291 |
| ENSG00000227706 | *RP11-301G19.1* | 8.185209824 | 0 | 0 |
| ENSG00000230537 | *RP11-305L7.1* | 2.406865591 | 0 | -0.15579 |
| ENSG00000254325 | *RP11-318K15.2* | 2.150319608 | 0 | -1.97361 |
| ENSG00000284237 | *RP11-328D5.4* | 2.049955596 | -0.76062 | 0 |
| ENSG00000233968 | *RP11-354E11.2* | 2.720256774 | 0 | 0 |
| ENSG00000251136 | *RP11-37B2.1* | 2.588727157 | -0.24707 | 0 |
| ENSG00000273108 | *RP11-416N2.4* | 2.593983611 | 0 | -2.13611 |
| ENSG00000225761 | *RP11-417O11.5* | 2.095733589 | -1.3219 | 0 |
| ENSG00000279142 | *RP11-463O9.2* | 3.362528113 | 0 | 0 |
| ENSG00000223722 | *RP11-467L13.5* | 2.079161618 | 0 | 0 |
| ENSG00000276259 | *RP11-481J2.4* | 2.610338501 | -0.50772 | 0 |
| ENSG00000257883 | *RP11-497G19.1* | 3.823936816 | -2.91085 | 0 |
| ENSG00000271981 | *RP11-573G6.8* | 2.364676918 | 0 | 0 |
| ENSG00000261161 | *RP11-58A18.1* | 2.899221621 | 0 | 0 |
| ENSG00000256615 | *RP11-59N23.3* | 4.846193012 | 0 | 0 |
| ENSG00000250696 | *RP11-704M14.1* | 4.89512259 | 0 | 0 |
| ENSG00000273493 | *RP11-80H18.4* | 2.530584573 | 0 | 0 |
| ENSG00000280153 | *RP11-876N24.3* | 3.473329186 | 0 | -0.99004 |
| ENSG00000267311 | *RP11-99A1.2* | 2.371716619 | 0 | -1.4271 |
| ENSG00000271857 | *RP1-244F24.1* | 3.975419139 | 0 | 0 |
| ENSG00000216316 | *RP3-354N19.3* | 2.158438162 | 0 | -2.16201 |
| ENSG00000251508 | *RP5-862P8.3* | 3.844531357 | 0 | 0 |
| ENSG00000278231 | *RP5-906C1.1* | 2.019573164 | 0 | -0.66355 |
| ENSG00000274536 | *RP6-159A1.4* | 2.130299447 | 0 | -0.43322 |
| ENSG00000269927 | *RP6-91H8.3* | 2.944460199 | 0 | -1.11335 |
| ENSG00000124813 | *RUNX2* | 2.752794381 | 0 | -1.43557 |
| ENSG00000171643 | *S100Z* | 2.189207436 | 0 | -0.27002 |
| ENSG00000069188 | *SDK2* | 2.16691378 | -0.6776 | 0 |
| ENSG00000168497 | *SDPR* | 2.088611943 | 0 | -4.47243 |
| ENSG00000124570 | *SERPINB6* | 2.595305345 | 0 | -2.51756 |
| ENSG00000230438 | *SERPINB9P1* | 2.216582 | 0 | -3.17708 |
| ENSG00000105251 | *SHD* | 2.448240476 | -2.4635 | 0 |
| ENSG00000180592 | *SKIDA1* | 2.679544982 | 0 | -2.69671 |
| ENSG00000185052 | *SLC24A3* | 4.042009493 | -0.10434 | 0 |
| ENSG00000172139 | *SLC9C1* | 5.465377457 | 0 | 0 |
| ENSG00000281010 | *snoR1* | 13.75414967 | 0 | 0 |
| ENSG00000212214 | *SNORA48* | 2.042928274 | 0 | 0 |
| ENSG00000207297 | *SNORD7* | 2.052555924 | 0 | 0 |
| ENSG00000077327 | *SPAG6* | 2.149030273 | 0 | -1.66473 |
| ENSG00000217330 | *SSXP10* | 5.144194023 | 0 | 0 |
| ENSG00000035720 | *STAP1* | 2.469828839 | 0 | -2.85902 |
| ENSG00000198829 | *SUCNR1* | 5.758500263 | 0 | -0.97305 |
| ENSG00000157703 | *SVOPL* | 2.135222908 | -0.78429 | 0 |
| ENSG00000152760 | *TCTEX1D1* | 4.10595961 | 0 | -3.52226 |
| ENSG00000263400 | *TMEM220-AS1* | 2.632293206 | 0 | -0.22918 |
| ENSG00000187653 | *TMSB4XP8* | 2.060279405 | 0 | 0 |
| ENSG00000173530 | *TNFRSF10D* | 2.006249882 | 0 | -4.09707 |
| ENSG00000130598 | *TNNI2* | 2.552902722 | 0 | 0 |
| ENSG00000073282 | *TP63* | 4.394898596 | -2.05405 | 0 |
| ENSG00000241911 | *TRBVB* | 4.941065231 | 0 | 0 |
| ENSG00000256590 | *TRDV3* | 4.570837696 | -0.65984 | 0 |
| ENSG00000211689 | *TRGC1* | 2.90523625 | -0.26434 | 0 |
| ENSG00000170893 | *TRH* | 4.36303827 | 0 | 0 |
| ENSG00000104447 | *TRPS1* | 2.464157426 | 0 | -0.86762 |
| ENSG00000237361 | *TUSC8* | 4.562645186 | -0.29811 | 0 |
| ENSG00000230266 | *XXYLT1-AS2* | 2.19038067 | -0.55538 | 0 |
| ENSG00000226048 | *YBX1P8* | 3.554148526 | 0 | -0.08623 |
| ENSG00000109906 | *ZBTB16* | 2.466185184 | 0 | -1.29225 |
| ENSG00000140948 | *ZCCHC14* | 2.360398041 | 0 | -0.94483 |
| ENSG00000147394 | *ZNF185* | 2.750665624 | 0 | -0.39151 |
| ENSG00000145908 | *ZNF300* | 4.115592159 | 0 | -0.13524 |
| ENSG00000168916 | *ZNF608* | 3.68659597 | -0.55422 | 0 |

**Supplementary Table 1B: List of genes downregulated in immature T-ALL**

| **Ensembl_ID** | **Genes** | **Log_2_fold change in T-ALL subtypes** | | |
| --- | --- | --- | --- | --- |
|  |  | **Immature** | **Cortical** | **Mature** |
| ENSG00000278404 | *MIAT_exon1* | -11.1397168 | 0 | 0 |
| ENSG00000184486 | *POU3F2* | -8.34432644 | 0 | 0 |
| ENSG00000260345 | *AC009052.12* | -7.91024503 | 0 | 0 |
| ENSG00000234200 | *U82671.8* | -7.82472584 | 0 | 0 |
| ENSG00000283602 | *AC011380.8* | -7.50835583 | 0 | 0 |
| ENSG00000064835 | *POU1F1* | -7.40780948 | 0 | 0 |
| ENSG00000132854 | *KANK4* | -7.3935734 | 0 | 0 |
| ENSG00000284418 | *RP11-370B11.4* | -7.26056426 | 0 | 0 |
| ENSG00000279590 | *AC005786.6* | -7.21662599 | 0 | 0 |
| ENSG00000107807 | *TLX1* | -7.21557651 | 0 | 0 |
| ENSG00000176920 | *FUT2* | -7.20705639 | 0 | 0 |
| ENSG00000255569 | *TRAV1-1* | -6.80504021 | 0 | 0 |
| ENSG00000229415 | *SFTA3* | -6.54552208 | 0 | 0 |
| ENSG00000255992 | *RP11-417L19.4* | -6.38088645 | 0 | 0 |
| ENSG00000264767 | *RN7SL237P* | -6.31370425 | 0 | 0 |
| ENSG00000275741 | *RP11-40E6.1* | -6.10154857 | 0 | 0 |
| ENSG00000259353 | *RP11-30K9.5* | -6.0928697 | 0 | 0 |
| ENSG00000239718 | *HLTF-AS1* | -6.06332053 | 0 | 0 |
| ENSG00000183117 | *CSMD1* | -6.01783905 | 0.176483 | 0 |
| ENSG00000235120 | *BSN-AS1* | -5.93698797 | 0 | 0 |
| ENSG00000260048 | *IGHV1OR16-3* | -5.92885523 | 0 | 0 |
| ENSG00000180287 | *PLD5* | -5.85247377 | 0 | 0 |
| ENSG00000228021 | *RP11-383C5.3* | -5.83597175 | 0 | 0 |
| ENSG00000229298 | *TUBB8P1* | -5.78074926 | 0 | 0 |
| ENSG00000113396 | *SLC27A6* | -5.76150923 | 0 | 0 |
| ENSG00000281910 | *SNORA50A* | -5.70280082 | 0 | 0 |
| ENSG00000232265 | *XXyac-YX155B6.5* | -5.68163993 | 0 | 0 |
| ENSG00000253140 | *RP11-567J20.3* | -5.5284577 | 0 | 0 |
| ENSG00000242770 | *RP11-180K7.1* | -5.48776252 | 0 | 0 |
| ENSG00000102854 | *MSLN* | -5.38523278 | 0 | 0 |
| ENSG00000277635 | *Metazoa_SRP* | -5.32920013 | 0 | 0 |
| ENSG00000234296 | *RP13-16H11.7* | -5.27802909 | 0 | 0 |
| ENSG00000248473 | *LINC01962* | -5.26679317 | 0 | 0 |
| ENSG00000207693 | *MIR602* | -5.25411935 | 0 | 0 |
| ENSG00000259129 | *LINC00648* | -4.94861414 | 0 | 0 |
| ENSG00000211710 | *TRBV4-1* | -4.92165128 | 0 | 0 |
| ENSG00000278862 | *RP11-571O6.2* | -4.90724068 | 0 | 0 |
| ENSG00000121440 | *PDZRN3* | -4.86170895 | 0 | 0 |
| ENSG00000244510 | *GS1-124K5.7* | -4.76879817 | 0 | 0 |
| ENSG00000229088 | *MTND1P10* | -4.73289435 | 0 | 0 |
| ENSG00000237211 | *SETP4* | -4.69986639 | 0 | 0 |
| ENSG00000235426 | *RP11-342M3.5* | -4.69766722 | 0 | 0 |
| ENSG00000211780 | *TRAV6* | -4.64073413 | 0 | 0 |
| ENSG00000255916 | *RP13-895J2.7* | -4.59025014 | 0 | 0 |
| ENSG00000011465 | *DCN* | -4.41608632 | 0 | 0 |
| ENSG00000198703 | *OR10R3P* | -4.19999001 | 0 | 0 |
| ENSG00000259899 | *CTD-3037G24.3* | -4.12400389 | 2.820184 | 0 |
| ENSG00000113520 | *IL4* | -4.06434631 | 0 | 0 |
| ENSG00000232891 | *RP11-136K14.1* | -4.06426091 | 0 | 0 |
| ENSG00000232034 | *AC092168.2* | -4.0453355 | 0 | 1.885519 |
| ENSG00000241073 | *RP4-714D9.2* | -4.02864317 | 0 | 0 |
| ENSG00000258869 | *RP11-204N11.1* | -4.01524951 | 0 | 0 |
| ENSG00000251138 | *RP11-81H3.2* | -4.00374745 | 0 | 0 |
| ENSG00000235329 | *RP5-1168A5.1* | -4.00319631 | 0 | 0 |
| ENSG00000211782 | *TRAV8-1* | -3.96294681 | 0 | 0 |
| ENSG00000172350 | *ABCG4* | -3.87710065 | 0 | 3.091848 |
| ENSG00000211714 | *TRBV7-3* | -3.85844511 | 0 | 0.222991 |
| ENSG00000186306 | *OR10T2* | -3.81612573 | 0 | 0 |
| ENSG00000115705 | *TPO* | -3.806104 | 0 | 1.314704 |
| ENSG00000182916 | *TCEAL7* | -3.7851187 | 0 | 0 |
| ENSG00000259876 | *CTD-3037G24.4* | -3.78011236 | 1.490444 | 0 |
| ENSG00000218582 | *GAPDHP63* | -3.758563 | 0 | 0 |
| ENSG00000266710 | *RN7SL48P* | -3.74302735 | 0 | 0 |
| ENSG00000258515 | *RP11-203M5.7* | -3.73756016 | 0 | 0 |
| ENSG00000259771 | *RP11-429D19.1* | -3.70336587 | 0 | 0 |
| ENSG00000184785 | *SMIM10* | -3.64508451 | 0 | 0 |
| ENSG00000252326 | *SNORD116-25* | -3.63834796 | 0 | 0 |
| ENSG00000274758 | *RP1-59D14.10* | -3.6324168 | 0 | 0 |
| ENSG00000267054 | *CTD-2540B15.10* | -3.62285897 | 0 | 0 |
| ENSG00000268987 | *CTC-435M10.10* | -3.56965388 | 0 | 0 |
| ENSG00000211801 | *TRAV21* | -3.50863757 | 0 | 0 |
| ENSG00000231873 | *RP11-761N21.1* | -3.47367039 | 0 | 0 |
| ENSG00000258728 | *RP11-195F19.29* | -3.42849619 | 0 | 0 |
| ENSG00000227459 | *AC079612.2* | -3.37946978 | 0 | 0 |
| ENSG00000265194 | *RP11-70L8.4* | -3.37868196 | 0 | 0 |
| ENSG00000273368 | *RP11-376P6.3* | -3.31096037 | 0 | 0 |
| ENSG00000211784 | *TRAV10* | -3.23274128 | 0 | 0 |
| ENSG00000206878 | *SNORA51* | -3.22585933 | 0 | 0 |
| ENSG00000207626 | *MIR562* | -3.22543045 | 0 | 0 |
| ENSG00000221333 | *MIR548K* | -3.213584 | 0 | 0 |
| ENSG00000275901 | *Metazoa_SRP* | -3.21002313 | 0 | 0 |
| ENSG00000232195 | *TOMM22P2* | -3.16481516 | 1.012883 | 0 |
| ENSG00000265272 | *RN7SL693P* | -3.13987287 | 0 | 0 |
| ENSG00000211819 | *TRAV40* | -3.07657423 | 0 | 0 |
| ENSG00000211779 | *TRAV5* | -3.0730276 | 0 | 0 |
| ENSG00000251226 | *RP11-469N6.1* | -3.05992734 | 0 | 2.94041 |
| ENSG00000256553 | *TRAV1-2* | -3.05615143 | 0 | 0 |
| ENSG00000278732 | *Metazoa_SRP* | -3.05506515 | 0 | 0 |
| ENSG00000201208 | *Y_RNA* | -2.98384315 | 0 | 0 |
| ENSG00000279180 | *RP11-417O18.1* | -2.97296425 | 0 | 0 |
| ENSG00000128510 | *CPA4* | -2.9497791 | 0.783721 | 0 |
| ENSG00000229330 | *AC006947.1* | -2.93643091 | 0 | 2.176786 |
| ENSG00000255864 | *RP11-444D3.1* | -2.91649034 | 0 | 0 |
| ENSG00000203758 | *OR10T1P* | -2.90979785 | 0 | 0 |
| ENSG00000170160 | *CCDC144A* | -2.87407382 | 0 | 0.722658 |
| ENSG00000280238 | *RP11-574K11.26* | -2.83930589 | 0 | 0 |
| ENSG00000284057 | *RP11-606E8.2* | -2.80942253 | 0 | 0 |
| ENSG00000223414 | *LINC00473* | -2.76380774 | 0 | 0.034859 |
| ENSG00000114200 | *BCHE* | -2.73865346 | 0 | 0 |
| ENSG00000211697 | *TRGV5* | -2.7324591 | 0 | 0 |
| ENSG00000166407 | *LMO1* | -2.6994791 | 0.489285 | 0 |
| ENSG00000102245 | *CD40LG* | -2.69780528 | 0 | 0.217818 |
| ENSG00000199363 | *SNORA63* | -2.68345363 | 0 | 0.299344 |
| ENSG00000276771 | *HOTAIRM1_2* | -2.64869739 | 0 | 0 |
| ENSG00000135750 | *KCNK1* | -2.63020356 | 0 | 0.77958 |
| ENSG00000198723 | *C19orf45* | -2.63017632 | 0 | 0 |
| ENSG00000222268 | *RNA5SP425* | -2.62627251 | 0 | 1.280184 |
| ENSG00000211820 | *TRAV41* | -2.62392174 | 0 | 0 |
| ENSG00000182230 | *FAM153B* | -2.60366346 | 0 | 1.531691 |
| ENSG00000211788 | *TRAV13-1* | -2.5638249 | 0 | 0 |
| ENSG00000250125 | *RP11-707A18.1* | -2.5532056 | 0 | 5.849783 |
| ENSG00000249458 | *RP11-624A4.1* | -2.55225933 | 0 | 0 |
| ENSG00000284503 | *MIR3652* | -2.51551815 | 0 | 0 |
| ENSG00000211777 | *TRAV3* | -2.5060833 | 0 | 0 |
| ENSG00000212290 | *RNA5SP424* | -2.49493484 | 0 | 0 |
| ENSG00000221803 | *SNORD23* | -2.48041256 | 0 | 1.697214 |
| ENSG00000275529 | *SNORD116-4* | -2.464136 | 0 | 0 |
| ENSG00000021645 | *NRXN3* | -2.4213291 | 0 | 0.471738 |
| ENSG00000275038 | *RP11-546B8.6* | -2.42123832 | 0 | 0.414698 |
| ENSG00000225030 | *RP4-784A16.3* | -2.41802075 | 0 | 0 |
| ENSG00000230942 | *HMGN1P5* | -2.41692986 | 1.250726 | 0 |
| ENSG00000222267 | *RNU6-892P* | -2.41667973 | 0 | 0 |
| ENSG00000207001 | *SNORD116-2* | -2.40468225 | 0 | 0 |
| ENSG00000269190 | *FBXO17* | -2.38826807 | 0 | 4.100089 |
| ENSG00000211776 | *TRAV2* | -2.38813249 | 0 | 0 |
| ENSG00000222343 | *RN7SKP139* | -2.36071392 | 0 | 0 |
| ENSG00000169715 | *MT1E* | -2.3593637 | 0 | 0.100015 |
| ENSG00000211785 | *TRAV12-1* | -2.35186523 | 0 | 0 |
| ENSG00000222960 | *RNU6-272P* | -2.32732484 | 0 | 0 |
| ENSG00000235127 | *AC068286.1* | -2.3269229 | 0 | 0 |
| ENSG00000205089 | *CCNI2* | -2.3259285 | 2.190709 | 0 |
| ENSG00000201545 | *RNU4-85P* | -2.32508254 | 0 | 0 |
| ENSG00000211786 | *TRAV8-2* | -2.30793928 | 0 | 0 |
| ENSG00000197826 | *C4orf22* | -2.30685579 | 0 | 1.593122 |
| ENSG00000252291 | *SNORA31* | -2.30241511 | 0 | 0 |
| ENSG00000126467 | *TSKS* | -2.27934218 | 0 | 0 |
| ENSG00000207116 | *RNU6-31P* | -2.27172699 | 0 | 0 |
| ENSG00000155974 | *GRIP1* | -2.26280273 | 0.369419 | 0 |
| ENSG00000007372 | *PAX6* | -2.25737787 | 0 | 0 |
| ENSG00000253649 | *PRSS51* | -2.24813974 | 0 | 0 |
| ENSG00000207802 | *MIR646* | -2.23024905 | 0 | 0 |
| ENSG00000200972 | *RNU5A-8P* | -2.22760602 | 0.314446 | 0 |
| ENSG00000252118 | *RNU6ATAC39P* | -2.21932837 | 0 | 0 |
| ENSG00000277568 | *uc_338* | -2.21787914 | 0 | 0 |
| ENSG00000206834 | *SNORA1* | -2.21035304 | 0 | 1.739745 |
| ENSG00000259396 | *RP11-16O9.2* | -2.20312138 | 0.193666 | 0 |
| ENSG00000268352 | *AC007228.5* | -2.19998872 | 0 | 4.198745 |
| ENSG00000253668 | *RP11-463C14.1* | -2.19954055 | 0 | 0.468036 |
| ENSG00000240194 | *CYMP* | -2.19455526 | 0 | 0 |
| ENSG00000230006 | *ANKRD36BP2* | -2.19441958 | 0 | 0.044991 |
| ENSG00000270474 | *IGHV3-29* | -2.18518302 | 1.058663 | 0 |
| ENSG00000237152 | *DLEU7-AS1* | -2.1781543 | 0.658903 | 0 |
| ENSG00000272096 | *RN7SL715P* | -2.17700533 | 0 | 2.252818 |
| ENSG00000259404 | *EFTUD1P1* | -2.1747706 | 0 | 0.532954 |
| ENSG00000200090 | *Y_RNA* | -2.15754344 | 0 | 0 |
| ENSG00000165194 | *PCDH19* | -2.15481277 | 0 | 4.894485 |
| ENSG00000231062 | *AC103563.9* | -2.1466573 | 0.923834 | 0 |
| ENSG00000232063 | *RP11-307E17.8* | -2.14411189 | 0 | 0.764826 |
| ENSG00000187122 | *SLIT1* | -2.12701612 | 0 | 0.987144 |
| ENSG00000066382 | *MPPED2* | -2.12589634 | 0 | 2.282971 |
| ENSG00000256248 | *RP11-123O10.4* | -2.12514757 | 0 | 1.519245 |
| ENSG00000133169 | *BEX1* | -2.12392688 | 1.107807 | 0 |
| ENSG00000265737 | *RP11-1157N2__B.2* | -2.11915348 | 0 | 0 |
| ENSG00000253926 | *RP11-26E5.1* | -2.1176217 | 0 | 0 |
| ENSG00000277864 | *SCARNA15* | -2.11342557 | 0 | 0 |
| ENSG00000269053 | *CTD-2521M24.8* | -2.09514827 | 0 | 1.443197 |
| ENSG00000137962 | *ARHGAP29* | -2.09251824 | 1.102354 | 0 |
| ENSG00000224237 | *MINOS1P3* | -2.08858215 | 0 | 0 |
| ENSG00000268089 | *GABRQ* | -2.08518233 | 0 | 7.001033 |
| ENSG00000235244 | *DANT2* | -2.06161695 | 0 | 0.246998 |
| ENSG00000252311 | *RNU1-103P* | -2.05784988 | 0 | 0.750263 |
| ENSG00000284324 | *MIR3175* | -2.05511007 | 0 | 0 |
| ENSG00000172824 | *CES4A* | -2.04239865 | 0 | 0.872274 |
| ENSG00000154229 | *PRKCA* | -2.04099711 | 0 | 0.776095 |
| ENSG00000249731 | *RP11-259O2.3* | -2.03970241 | 0 | 3.637644 |
| ENSG00000206712 | *RNU6-26P* | -2.03958577 | 0 | 1.173801 |
| ENSG00000080644 | *CHRNA3* | -2.03692607 | 0.267576 | 0 |
| ENSG00000266109 | *MIR4440* | -2.02960648 | 0 | 0 |
| ENSG00000202137 | *Y_RNA* | -2.01736507 | 0 | 0 |
| ENSG00000131773 | *KHDRBS3* | -2.01445593 | 0 | 1.284896 |
| ENSG00000221264 | *MIR1284* | -2.00960459 | 0 | 0 |
| ENSG00000117971 | *CHRNB4* | -2.00833519 | 0 | 3.289374 |
| ENSG00000246859 | *STARD4-AS1* | -2.00635236 | 0 | 0.053231 |

**Supplementary Table 2A: List of genes upregulated in cortical T-ALL**

| **Ensembl_ID** | **Gene Name** | **Log_2_fold change in T-ALL subtypes** | | |
| --- | --- | --- | --- | --- |
|  |  | **Immature** | **Cortical** | **Mature** |
| ENSG00000227459 | *AC079612.2* | 0 | 3.37947 | 0 |
| ENSG00000260089 | *ADAM3B* | -1.22678 | 3.702988 | 0 |
| ENSG00000140470 | *ADAMTS17* | 0 | 2.535605 | -1.21332 |
| ENSG00000131016 | *AKAP12* | -1.76204 | 3.110881 | 0 |
| ENSG00000237356 | *AL163953.3* | 0 | 2.219599 | -0.55841 |
| ENSG00000153930 | *ANKFN1* | 0 | 2.409537 | -0.46863 |
| ENSG00000114200 | *BCHE* | 0 | 2.738653 | 0 |
| ENSG00000114529 | *C3orf52* | -0.5958 | 2.319686 | 0 |
| ENSG00000205089 | *CCNI2* | -2.32593 | 2.190709 | 0 |
| ENSG00000158477 | *CD1A* | -0.2908 | 4.457019 | 0 |
| ENSG00000158481 | *CD1C* | -0.39005 | 3.1832 | 0 |
| ENSG00000010610 | *CD4* | -0.34644 | 2.222396 | 0 |
| ENSG00000001626 | *CFTR* | -1.25772 | 7.971822 | 0 |
| ENSG00000169031 | *COL4A3* | -0.03356 | 3.455007 | 0 |
| ENSG00000134376 | *CRB1* | 0 | 4.384765 | -0.80624 |
| ENSG00000259899 | *CTD-3037G24.3* | -4.124 | 2.820184 | 0 |
| ENSG00000240194 | *CYMP* | 0 | 2.194555 | 0 |
| ENSG00000136848 | *DAB2IP* | -1.02169 | 2.863171 | 0 |
| ENSG00000011465 | *DCN* | 0 | 4.416086 | 0 |
| ENSG00000115380 | *EFEMP1* | 0 | 3.237982 | -2.07838 |
| ENSG00000162374 | *ELAVL4* | 0 | 2.815305 | -1.03096 |
| ENSG00000119888 | *EPCAM* | 0 | 2.994865 | -0.94951 |
| ENSG00000124882 | *EREG* | 0 | 2.028371 | -0.71998 |
| ENSG00000165323 | *FAT3* | 0 | 2.040384 | -1.74142 |
| ENSG00000234753 | *FOXP4-AS1* | -1.08256 | 2.001204 | 0 |
| ENSG00000137726 | *FXYD6* | 0 | 2.490117 | -0.34645 |
| ENSG00000218582 | *GAPDHP63* | 0 | 3.758563 | 0 |
| ENSG00000168243 | *GNG4* | -0.16296 | 2.217804 | 0 |
| ENSG00000188536 | *HBA2* | -0.18333 | 3.38784 | 0 |
| ENSG00000108924 | *HLF* | -0.16478 | 2.703364 | 0 |
| ENSG00000113520 | *IL4* | 0 | 4.064346 | 0 |
| ENSG00000159556 | *ISL2* | 0 | 2.230497 | -0.12471 |
| ENSG00000132854 | *KANK4* | 0 | 7.393573 | 0 |
| ENSG00000267313 | *KC6* | -0.40988 | 3.195475 | 0 |
| ENSG00000162687 | *KCNT2* | 0 | 2.385619 | -1.31006 |
| ENSG00000232021 | *LEF1-AS1* | -0.09893 | 2.438425 | 0 |
| ENSG00000231976 | *LINC00202* | 0 | 4.98582 | -1.31089 |
| ENSG00000245526 | *LINC00461* | 0 | 2.636698 | -1.23286 |
| ENSG00000259129 | *LINC00648* | 0 | 4.948614 | 0 |
| ENSG00000170500 | *LONRF2* | 0 | 2.171102 | -1.28926 |
| ENSG00000134138 | *MEIS2* | -1.71765 | 2.056134 | 0 |
| ENSG00000224237 | *MINOS1P3* | 0 | 2.088582 | 0 |
| ENSG00000221264 | *MIR1284* | 0 | 2.009605 | 0 |
| ENSG00000266109 | *MIR4440* | 0 | 2.029606 | 0 |
| ENSG00000207802 | *MIR646* | 0 | 2.230249 | 0 |
| ENSG00000124615 | *MOCS1* | -0.18348 | 2.415924 | 0 |
| ENSG00000095777 | *MYO3A* | -0.65632 | 2.308105 | 0 |
| ENSG00000061676 | *NCKAP1* | 0 | 2.816908 | -4.19619 |
| ENSG00000136352 | *NKX2-1* | -0.54611 | 4.437979 | 0 |
| ENSG00000165246 | *NLGN4Y* | 0 | 2.067675 | -0.02812 |
| ENSG00000197444 | *OGDHL* | -0.73476 | 2.025053 | 0 |
| ENSG00000198703 | *OR10R3P* | 0 | 4.19999 | 0 |
| ENSG00000203758 | *OR10T1P* | 0 | 2.909798 | 0 |
| ENSG00000186306 | *OR10T2* | 0 | 3.816126 | 0 |
| ENSG00000007372 | *PAX6* | 0 | 2.257378 | 0 |
| ENSG00000121440 | *PDZRN3* | 0 | 4.861709 | 0 |
| ENSG00000183571 | *PGPEP1L* | 0 | 2.254345 | -0.88357 |
| ENSG00000180287 | *PLD5* | 0 | 5.852474 | 0 |
| ENSG00000120278 | *PLEKHG1* | -0.35476 | 2.607858 | 0 |
| ENSG00000124225 | *PMEPA1* | 0 | 4.025086 | -1.48474 |
| ENSG00000005421 | *PON1* | 0 | 3.690476 | -0.70696 |
| ENSG00000064835 | *POU1F1* | 0 | 7.407809 | 0 |
| ENSG00000184486 | *POU3F2* | 0 | 8.344326 | 0 |
| ENSG00000132170 | *PPARG* | 0 | 3.227021 | -0.83912 |
| ENSG00000253649 | *PRSS51* | 0 | 2.24814 | 0 |
| ENSG00000166450 | *PRTG* | 0 | 3.199745 | -3.76964 |
| ENSG00000173482 | *PTPRM* | 0 | 3.369522 | -1.41024 |
| ENSG00000136237 | *RAPGEF5* | -0.36426 | 2.077644 | 0 |
| ENSG00000212290 | *RNA5SP424* | 0 | 2.494935 | 0 |
| ENSG00000237659 | *RNASEH2CP1* | 0 | 2.83204 | -0.11654 |
| ENSG00000201545 | *RNU4-85P* | 0 | 2.325083 | 0 |
| ENSG00000222960 | *RNU6-272P* | 0 | 2.327325 | 0 |
| ENSG00000207116 | *RNU6-31P* | 0 | 2.271727 | 0 |
| ENSG00000222267 | *RNU6-892P* | 0 | 2.41668 | 0 |
| ENSG00000252118 | *RNU6ATAC39P* | 0 | 2.219328 | 0 |
| ENSG00000265737 | *RP11-1157N2__B.2* | 0 | 2.119153 | 0 |
| ENSG00000232891 | *RP11-136K14.1* | 0 | 4.064261 | 0 |
| ENSG00000236656 | *RP11-144L1.4* | 0 | 3.771936 | -2.88968 |
| ENSG00000242770 | *RP11-180K7.1* | 0 | 5.487763 | 0 |
| ENSG00000258869 | *RP11-204N11.1* | 0 | 4.01525 | 0 |
| ENSG00000253926 | *RP11-26E5.1* | 0 | 2.117622 | 0 |
| ENSG00000224593 | *RP11-30B1.1* | -0.72142 | 3.257475 | 0 |
| ENSG00000273368 | *RP11-376P6.3* | 0 | 3.31096 | 0 |
| ENSG00000269888 | *RP11-3P17.5* | 0 | 2.152562 | -1.36237 |
| ENSG00000229914 | *RP11-404O13.4* | -0.25465 | 2.499391 | 0 |
| ENSG00000176320 | *RP11-404O13.5* | -0.66575 | 2.125881 | 0 |
| ENSG00000255864 | *RP11-444D3.1* | 0 | 2.91649 | 0 |
| ENSG00000261823 | *RP11-48G14.1* | 0 | 2.186892 | -0.6204 |
| ENSG00000284057 | *RP11-606E8.2* | 0 | 2.809423 | 0 |
| ENSG00000235033 | *RP11-61I13.3* | 0 | 2.988045 | -3.73337 |
| ENSG00000259097 | *RP11-61O1.1* | 0 | 4.867539 | -0.70293 |
| ENSG00000249458 | *RP11-624A4.1* | 0 | 2.552259 | 0 |
| ENSG00000265194 | *RP11-70L8.4* | 0 | 3.378682 | 0 |
| ENSG00000279693 | *RP11-71L14.3* | -1.07 | 3.11617 | 0 |
| ENSG00000261488 | *RP11-757F18.5* | 0 | 2.774079 | -0.17992 |
| ENSG00000251138 | *RP11-81H3.2* | 0 | 4.003747 | 0 |
| ENSG00000248596 | *RP11-844P9.5* | 0 | 2.100356 | -0.3468 |
| ENSG00000234296 | *RP13-16H11.7* | 0 | 5.278029 | 0 |
| ENSG00000229771 | *RP4-644L1.2* | 0 | 2.678006 | -0.01501 |
| ENSG00000236975 | *RP5-1065P14.2* | 0 | 2.690627 | -0.72808 |
| ENSG00000277864 | *SCARNA15* | 0 | 2.113426 | 0 |
| ENSG00000171951 | *SCG2* | 0 | 2.086016 | -1.61846 |
| ENSG00000237211 | *SETP4* | 0 | 4.699866 | 0 |
| ENSG00000235101 | *SETP9* | 0 | 2.047511 | -0.13959 |
| ENSG00000229415 | *SFTA3* | 0 | 6.545522 | 0 |
| ENSG00000109686 | *SH3D19* | 0 | 2.591073 | -4.23318 |
| ENSG00000140600 | *SH3GL3* | -0.01856 | 2.638992 | 0 |
| ENSG00000165646 | *SLC18A2* | 0 | 2.123989 | -0.77682 |
| ENSG00000113396 | *SLC27A6* | 0 | 5.761509 | 0 |
| ENSG00000184785 | *SMIM10* | 0 | 3.645085 | 0 |
| ENSG00000103056 | *SMPD3* | 0 | 3.004948 | -0.81028 |
| ENSG00000019549 | *SNAI2* | 0 | 2.564905 | -2.46362 |
| ENSG00000252291 | *SNORA31* | 0 | 2.302415 | 0 |
| ENSG00000281910 | *SNORA50A* | 0 | 5.702801 | 0 |
| ENSG00000206878 | *SNORA51* | 0 | 3.225859 | 0 |
| ENSG00000112320 | *SOBP* | 0 | 2.531681 | -1.50123 |
| ENSG00000182916 | *TCEAL7* | 0 | 3.785119 | 0 |
| ENSG00000187621 | *TCL6* | 0 | 2.448857 | -0.53185 |
| ENSG00000107807 | *TLX1* | 0 | 7.215577 | 0 |
| ENSG00000211784 | *TRAV10* | 0 | 3.232741 | 0 |
| ENSG00000255569 | *TRAV1-1* | 0 | 6.80504 | 0 |
| ENSG00000256553 | *TRAV1-2* | 0 | 3.056151 | 0 |
| ENSG00000211785 | *TRAV12-1* | 0 | 2.351865 | 0 |
| ENSG00000211788 | *TRAV13-1* | 0 | 2.563825 | 0 |
| ENSG00000211776 | *TRAV2* | 0 | 2.388132 | 0 |
| ENSG00000211801 | *TRAV21* | 0 | 3.508638 | 0 |
| ENSG00000211807 | *TRAV26-1* | 0 | 3.234062 | -0.26031 |
| ENSG00000211777 | *TRAV3* | 0 | 2.506083 | 0 |
| ENSG00000211815 | *TRAV36DV7* | 0 | 2.688414 | -0.11158 |
| ENSG00000211819 | *TRAV40* | 0 | 3.076574 | 0 |
| ENSG00000211820 | *TRAV41* | 0 | 2.623922 | 0 |
| ENSG00000211779 | *TRAV5* | 0 | 3.073028 | 0 |
| ENSG00000211780 | *TRAV6* | 0 | 4.640734 | 0 |
| ENSG00000211782 | *TRAV8-1* | 0 | 3.962947 | 0 |
| ENSG00000211786 | *TRAV8-2* | 0 | 2.307939 | 0 |
| ENSG00000211790 | *TRAV8-4* | 0 | 2.026743 | -3.1538 |
| ENSG00000226660 | *TRBV2* | -1.29246 | 5.453272 | 0 |
| ENSG00000237254 | *TRBV30* | -0.98658 | 4.878612 | 0 |
| ENSG00000211710 | *TRBV4-1* | 0 | 4.921651 | 0 |
| ENSG00000211804 | *TRDV1* | 0 | 2.159575 | -0.46877 |
| ENSG00000233306 | *TRGV2* | 0 | 2.170041 | -1.21908 |
| ENSG00000211697 | *TRGV5* | 0 | 2.732459 | 0 |
| ENSG00000126467 | *TSKS* | 0 | 2.279342 | 0 |
| ENSG00000229298 | *TUBB8P1* | 0 | 5.780749 | 0 |
| ENSG00000277568 | *uc_338* | 0 | 2.217879 | 0 |
| ENSG00000188933 | *USP32P1* | -0.33638 | 2.410766 | 0 |
| ENSG00000202137 | *Y_RNA* | 0 | 2.017365 | 0 |
| ENSG00000201208 | *Y_RNA* | 0 | 2.983843 | 0 |
| ENSG00000102935 | *ZNF423* | -0.53774 | 2.085974 | 0 |
| ENSG00000149506 | *ZP1* | -0.4676 | 2.48384 | 0 |

**Supplementary Table 2B: List of genes downregulated in cortical T-ALL**

| **Ensembl_ID** | **Gene Name** | **Log_2_fold change in T-ALL subtypes** | | |
| --- | --- | --- | --- | --- |
|  |  | **Immature** | **Cortical** | **Mature** |
| ENSG00000281010 | *snoR1* | 0 | -13.7541 | 0 |
| ENSG00000243766 | *HOTTIP* | 0 | -9.11701 | 0 |
| ENSG00000152661 | *GJA1* | 0 | -8.72565 | 0 |
| ENSG00000106031 | *HOXA13* | 0 | -8.71155 | 0 |
| ENSG00000256508 | *MRGPRF-AS1* | 0 | -8.36603 | 0 |
| ENSG00000227706 | *RP11-301G19.1* | 0 | -8.18521 | 0 |
| ENSG00000276600 | *RAB7B* | 0 | -8.00863 | 0 |
| ENSG00000266446 | *RP11-149I2.4* | 0 | -7.23201 | 0.499324 |
| ENSG00000241657 | *TRBV11-2* | 0 | -7.05402 | 0.230311 |
| ENSG00000153086 | *ACMSD* | 0 | -7.00348 | 0 |
| ENSG00000155052 | *CNTNAP5* | 6.266332 | -6.99281 | 0 |
| ENSG00000269107 | *RP11-15H20.7* | 0 | -5.95236 | 1.456739 |
| ENSG00000152467 | *ZSCAN1* | 0 | -5.9016 | 0 |
| ENSG00000183248 | *PRR36* | 0 | -5.83826 | 3.069558 |
| ENSG00000280560 | *LINC01374* | 0 | -5.83492 | 0 |
| ENSG00000122548 | *KIAA0087* | 0 | -5.7846 | 0 |
| ENSG00000274859 | *RP11-136L23.2* | 0 | -5.73527 | 0 |
| ENSG00000253474 | *RP11-10H3.1* | 1.390869 | -5.63667 | 0 |
| ENSG00000254687 | *RP11-162D9.3* | 2.094755 | -5.61857 | 0 |
| ENSG00000091879 | *ANGPT2* | 0 | -5.6078 | 0 |
| ENSG00000203618 | *GP1BB* | 0 | -5.55085 | 1.990615 |
| ENSG00000234906 | *APOC2* | 0 | -5.50567 | 0 |
| ENSG00000260482 | *CTD-2196E14.9* | 0 | -5.50426 | 2.823506 |
| ENSG00000274177 | *LLNLR-284B4.1* | 0 | -5.46841 | 0 |
| ENSG00000172139 | *SLC9C1* | 0 | -5.46538 | 0 |
| ENSG00000225640 | *LINC01729* | 0 | -5.4553 | 0 |
| ENSG00000281991 | *TMEM265* | 0 | -5.43084 | 0.473675 |
| ENSG00000240990 | *HOXA11-AS* | 0 | -5.3532 | 0 |
| ENSG00000283269 | *AC005307.3* | 0 | -5.26691 | 3.480403 |
| ENSG00000217330 | *SSXP10* | 0 | -5.14419 | 0 |
| ENSG00000233766 | *AC098617.1* | 0 | -5.07534 | 0 |
| ENSG00000132329 | *RAMP1* | 0 | -5.05618 | 0 |
| ENSG00000249679 | *RP11-279O9.4* | 0 | -5.02742 | 0 |
| ENSG00000130307 | *USHBP1* | 0 | -5.0203 | 4.478697 |
| ENSG00000174482 | *LINGO2* | 0 | -5.00627 | 0 |
| ENSG00000100968 | *NFATC4* | 1.036222 | -4.98032 | 0 |
| ENSG00000241911 | *TRBVB* | 0 | -4.94107 | 0 |
| ENSG00000268133 | *AC003002.4* | 0 | -4.90801 | 0 |
| ENSG00000176435 | *CLEC14A* | 2.602557 | -4.90337 | 0 |
| ENSG00000250696 | *RP11-704M14.1* | 0 | -4.89512 | 0 |
| ENSG00000214787 | *MS4A4E* | 0 | -4.88758 | 0 |
| ENSG00000229996 | *AC093585.6* | 0 | -4.86851 | 2.265696 |
| ENSG00000256615 | *RP11-59N23.3* | 0 | -4.84619 | 0 |
| ENSG00000255439 | *RP11-196G11.1* | 0 | -4.81545 | 0.961193 |
| ENSG00000145431 | *PDGFC* | 0 | -4.79855 | 0 |
| ENSG00000273107 | *RP11-165A20.3* | 0 | -4.79048 | 0 |
| ENSG00000283828 | *RP11-98F14.12* | 0.0972 | -4.76317 | 0 |
| ENSG00000006638 | *TBXA2R* | 0 | -4.7555 | 1.263546 |
| ENSG00000214652 | *ZNF727* | 0 | -4.75297 | 2.84757 |
| ENSG00000260869 | *AC002310.13* | 0 | -4.71821 | 1.362376 |
| ENSG00000264659 | *CTD-2006K23.2* | 1.922949 | -4.69428 | 0 |
| ENSG00000235829 | *TBCAP2* | 1.901742 | -4.65913 | 0 |
| ENSG00000248309 | *MEF2C-AS1* | 0 | -4.6452 | 0 |
| ENSG00000255432 | *RP11-831H9.11* | 0.592029 | -4.62059 | 0 |
| ENSG00000187772 | *LIN28B* | 0 | -4.52026 | 0 |
| ENSG00000185008 | *ROBO2* | 0 | -4.46761 | 0 |
| ENSG00000101405 | *OXT* | 0 | -4.44623 | 1.776461 |
| ENSG00000085552 | *IGSF9* | 0.059079 | -4.4014 | 0 |
| ENSG00000253981 | *ALG1L13P* | 0 | -4.39604 | 0 |
| ENSG00000170893 | *TRH* | 0 | -4.36304 | 0 |
| ENSG00000233191 | *AC006372.6* | 0 | -4.35193 | 0 |
| ENSG00000258186 | *SLC7A5P2* | 0 | -4.28455 | 0 |
| ENSG00000224614 | *TNK2-AS1* | 0 | -4.22161 | 1.591194 |
| ENSG00000142235 | *LMTK3* | 0 | -4.20212 | 2.747916 |
| ENSG00000261459 | *AC002310.11* | 0 | -4.17014 | 1.195236 |
| ENSG00000235333 | *PVRIG2P* | 0 | -4.16048 | 1.46755 |
| ENSG00000273218 | *LLNLR-246C6.1* | 0 | -4.13098 | 3.261759 |
| ENSG00000253637 | *IGLVV-58* | 0 | -4.09806 | 0 |
| ENSG00000213083 | *AC010731.6* | 0 | -4.07122 | 0 |
| ENSG00000164929 | *BAALC* | 0 | -4.04715 | 0 |
| ENSG00000244588 | *RAD21L1* | 0 | -4.03853 | 4.173702 |
| ENSG00000275966 | *RP11-1055B8.9* | 0 | -4.02289 | 0.951611 |
| ENSG00000175497 | *DPP10* | 2.279709 | -3.98821 | 0 |
| ENSG00000271857 | *RP1-244F24.1* | 0 | -3.97542 | 0 |
| ENSG00000169083 | *AR* | 5.39806 | -3.94799 | 0 |
| ENSG00000196593 | *ANKRD20A19P* | 0 | -3.93879 | 1.917671 |
| ENSG00000250102 | *RP11-314N14.1* | 0 | -3.92651 | 1.072111 |
| ENSG00000282907 | *LA16c-407A10.3* | 0 | -3.91383 | 2.277519 |
| ENSG00000133454 | *MYO18B* | 5.701112 | -3.90402 | 0 |
| ENSG00000265458 | *RP13-20L14.6* | 0 | -3.9 | 0.901904 |
| ENSG00000225418 | *AKR1C5P* | 0 | -3.86302 | 0 |
| ENSG00000251508 | *RP5-862P8.3* | 0 | -3.84453 | 0 |
| ENSG00000225449 | *RAB6C-AS1* | 0 | -3.77084 | 1.765505 |
| ENSG00000211809 | *TRAV27* | 0 | -3.75786 | 2.092729 |
| ENSG00000279357 | *RP11-475D10.4* | 0 | -3.66964 | 1.714376 |
| ENSG00000162594 | *IL23R* | 0 | -3.66429 | 3.871252 |
| ENSG00000267406 | *CTD-2189E23.2* | 0 | -3.66193 | 0 |
| ENSG00000214369 | *AC009967.3* | 0 | -3.6372 | 0.82414 |
| ENSG00000167281 | *RBFOX3* | 0 | -3.62283 | 2.337595 |
| ENSG00000198502 | *HLA-DRB5* | 0 | -3.59452 | 0 |
| ENSG00000156689 | *GLYATL2* | 0 | -3.59313 | 0 |
| ENSG00000187068 | *C3orf70* | 0 | -3.59227 | 3.114511 |
| ENSG00000140873 | *ADAMTS18* | 2.279993 | -3.59108 | 0 |
| ENSG00000049089 | *COL9A2* | 0 | -3.57841 | 1.911435 |
| ENSG00000255177 | *RP11-532E4.2* | 0 | -3.56718 | 1.346279 |
| ENSG00000230778 | *ANKRD63* | 0 | -3.55567 | 3.349395 |
| ENSG00000170412 | *GPRC5C* | 0.994156 | -3.54564 | 0 |
| ENSG00000150281 | *CTF1* | 0 | -3.54494 | 2.682881 |
| ENSG00000259031 | *CTD-2062F14.3* | 0 | -3.54159 | 2.297482 |
| ENSG00000198336 | *MYL4* | 0 | -3.51882 | 0 |
| ENSG00000206066 | *IGLL3P* | 0 | -3.48989 | 0 |
| ENSG00000196557 | *CACNA1H* | 0 | -3.48528 | 2.466297 |
| ENSG00000226800 | *CACTIN-AS1* | 0 | -3.48233 | 2.578792 |
| ENSG00000279962 | *RP11-720L3.1* | 0 | -3.4791 | 4.462882 |
| ENSG00000252766 | *RNU6-255P* | 0 | -3.46912 | 0 |
| ENSG00000265566 | *RN7SL605P* | 0 | -3.45802 | 1.191465 |
| ENSG00000203286 | *Metazoa_SRP* | 0 | -3.45568 | 2.974477 |
| ENSG00000243566 | *UPK3B* | 0 | -3.43269 | 2.648062 |
| ENSG00000268262 | *CTC-246B18.8* | 0 | -3.42199 | 1.049915 |
| ENSG00000178531 | *CTXN1* | 0 | -3.42115 | 1.293745 |
| ENSG00000223118 | *RN7SKP102* | 0.841986 | -3.40265 | 0 |
| ENSG00000234120 | *AF228730.8* | 0 | -3.3863 | 0 |
| ENSG00000114923 | *SLC4A3* | 0 | -3.38223 | 4.438281 |
| ENSG00000279142 | *RP11-463O9.2* | 0 | -3.36253 | 0 |
| ENSG00000199426 | *RNU1-108P* | 0 | -3.35828 | 0 |
| ENSG00000227195 | *MIR663AHG* | 0 | -3.34015 | 2.387096 |
| ENSG00000114631 | *PODXL2* | 0.433558 | -3.33023 | 0 |
| ENSG00000050628 | *PTGER3* | 0 | -3.3031 | 0.009271 |
| ENSG00000179673 | *RPRML* | 0 | -3.28431 | 3.503872 |
| ENSG00000205863 | *C1QTNF9B* | 0 | -3.25414 | 1.394239 |
| ENSG00000229941 | *AC012499.1* | 0 | -3.25214 | 1.335946 |
| ENSG00000228526 | *MIR34AHG* | 0 | -3.25151 | 2.001922 |
| ENSG00000127955 | *GNAI1* | 0 | -3.24507 | 0 |
| ENSG00000267275 | *CTD-2562J15.6* | 0 | -3.21706 | 1.809591 |
| ENSG00000124657 | *OR2B6* | 0 | -3.20299 | 1.390797 |
| ENSG00000126562 | *WNK4* | 0 | -3.18824 | 1.742835 |
| ENSG00000284419 | *MIR663A* | 0 | -3.17706 | 1.089419 |
| ENSG00000185340 | *GAS2L1* | 0 | -3.16512 | 1.758953 |
| ENSG00000251656 | *PRELID3BP5* | 0 | -3.15622 | 1.521838 |
| ENSG00000134323 | *MYCN* | 1.135837 | -3.14833 | 0 |
| ENSG00000144785 | *RP11-977G19.10* | 0.093988 | -3.14615 | 0 |
| ENSG00000207428 | *RNU6-95P* | 0 | -3.14407 | 0 |
| ENSG00000211642 | *IGLV10-54* | 0 | -3.12843 | 0 |
| ENSG00000184702 | *SEPT5* | 0 | -3.12837 | 0.328723 |
| ENSG00000259735 | *RP11-356M20.3* | 0 | -3.12594 | 3.093333 |
| ENSG00000204603 | *LINC01257* | 0 | -3.12411 | 0 |
| ENSG00000260369 | *CTD-2526A2.2* | 0 | -3.11821 | 1.636553 |
| ENSG00000024422 | *EHD2* | 0 | -3.11394 | 0.929275 |
| ENSG00000267048 | *RP11-566K11.7* | 0 | -3.104 | 3.047909 |
| ENSG00000184371 | *CSF1* | 0 | -3.10203 | 0 |
| ENSG00000128040 | *SPINK2* | 1.111771 | -3.09988 | 0 |
| ENSG00000269590 | *CTD-2192J16.24* | 0 | -3.09468 | 0.559969 |
| ENSG00000259230 | *CTD-2555C10.3* | 0 | -3.05135 | 3.564538 |
| ENSG00000102109 | *PCSK1N* | 0 | -3.05117 | 5.540218 |
| ENSG00000279073 | *RP11-229P13.27* | 0 | -3.03578 | 2.196178 |
| ENSG00000274799 | *Metazoa_SRP* | 0 | -3.03287 | 3.99633 |
| ENSG00000267939 | *CTD-2325M2.1* | 0 | -3.00562 | 1.017735 |
| ENSG00000254631 | *RP11-702H23.4* | 0 | -3.00561 | 0 |
| ENSG00000249962 | *RP11-80H5.5* | 0 | -2.99949 | 0.893725 |
| ENSG00000251643 | *RP11-91J3.1* | 0 | -2.99365 | 1.911486 |
| ENSG00000279336 | *AL353662.1* | 0 | -2.99331 | 3.159934 |
| ENSG00000144407 | *PTH2R* | 0 | -2.9867 | 0.769124 |
| ENSG00000255186 | *RP11-514F3.5* | 0 | -2.92371 | 0.737969 |
| ENSG00000211640 | *IGLV6-57* | 0 | -2.92325 | 0 |
| ENSG00000234230 | *ZFX-AS1* | 0 | -2.92224 | 2.966031 |
| ENSG00000277958 | *Metazoa_SRP* | 0 | -2.91286 | 2.738781 |
| ENSG00000257883 | *RP11-497G19.1* | 3.823937 | -2.91085 | 0 |
| ENSG00000162931 | *TRIM17* | 0 | -2.90439 | 1.776206 |
| ENSG00000261161 | *RP11-58A18.1* | 0 | -2.89922 | 0 |
| ENSG00000273141 | *RP11-820I16.4* | 0 | -2.89691 | 1.272308 |
| ENSG00000277950 | *Metazoa_SRP* | 0 | -2.88715 | 1.378495 |
| ENSG00000186235 | *AC016757.3* | 0.2257 | -2.88619 | 0 |
| ENSG00000266835 | *GAPLINC* | 0 | -2.86189 | 2.801419 |
| ENSG00000259784 | *RP11-20I23.3* | 0 | -2.8594 | 2.579134 |
| ENSG00000249086 | *AC051649.12* | 0 | -2.85935 | 0.897431 |
| ENSG00000169992 | *NLGN2* | 0 | -2.85775 | 0.928866 |
| ENSG00000224830 | *OR2X1P* | 1.077031 | -2.84833 | 0 |
| ENSG00000201990 | *Y_RNA* | 0 | -2.84714 | 0 |
| ENSG00000066056 | *TIE1* | 0 | -2.84642 | 0.783262 |
| ENSG00000257696 | *RP11-175P13.2* | 0 | -2.84504 | 2.826223 |
| ENSG00000184414 | *IRS3P* | 0 | -2.84016 | 2.065694 |
| ENSG00000178882 | *RFLNA* | 0 | -2.8216 | 2.334855 |
| ENSG00000267598 | *CTC-250I14.6* | 0 | -2.8204 | 3.022683 |
| ENSG00000262481 | *TMEM256-PLSCR3* | 0 | -2.818 | 1.454611 |
| ENSG00000147799 | *ARHGAP39* | 0 | -2.81782 | 2.190978 |
| ENSG00000167889 | *MGAT5B* | 0 | -2.81352 | 1.671527 |
| ENSG00000182272 | *B4GALNT4* | 0 | -2.80902 | 3.491756 |
| ENSG00000006757 | *PNPLA4* | 0.502931 | -2.80766 | 0 |
| ENSG00000275807 | *RP11-1348G14.8* | 0 | -2.80562 | 1.932151 |
| ENSG00000242435 | *UPK3BP1* | 0.206677 | -2.79787 | 0 |
| ENSG00000277504 | *RP11-278A23.4* | 0 | -2.77528 | 2.491119 |
| ENSG00000204060 | *FOXO6* | 0 | -2.77516 | 2.45164 |
| ENSG00000179097 | *HTR1F* | 0 | -2.76565 | 0 |
| ENSG00000274505 | *Metazoa_SRP* | 0 | -2.75686 | 0.694797 |
| ENSG00000263847 | *RP11-143J12.3* | 0 | -2.75665 | 2.575991 |
| ENSG00000184221 | *OLIG1* | 0 | -2.74603 | 0 |
| ENSG00000230666 | *CEACAM22P* | 2.918434 | -2.73654 | 0 |
| ENSG00000214803 | *RP11-37N22.1* | 0 | -2.7351 | 3.327929 |
| ENSG00000136205 | *TNS3* | 0.060122 | -2.735 | 0 |
| ENSG00000280968 | *MIR3653* | 0 | -2.72777 | 2.570892 |
| ENSG00000231513 | *E2F6P4* | 0 | -2.72531 | 0 |
| ENSG00000233968 | *RP11-354E11.2* | 0 | -2.72026 | 0 |
| ENSG00000112486 | *CCR6* | 0 | -2.7164 | 3.275166 |
| ENSG00000231916 | *AC006033.22* | 0 | -2.71516 | 0 |
| ENSG00000176678 | *FOXL1* | 0 | -2.71039 | 0 |
| ENSG00000213997 | *PGAM1P7* | 0 | -2.69673 | 0 |
| ENSG00000163737 | *PF4* | 0 | -2.69608 | 0 |
| ENSG00000102996 | *MMP15* | 0 | -2.69201 | 0.609559 |
| ENSG00000010438 | *PRSS3* | 0 | -2.66059 | 0 |
| ENSG00000233236 | *AP001171.1* | 0 | -2.65812 | 0 |
| ENSG00000261431 | *RP4-616B8.4* | 0 | -2.65497 | 0.399256 |
| ENSG00000130300 | *PLVAP* | 0 | -2.64475 | 3.310543 |
| ENSG00000264188 | *RP11-13N13.5* | 0 | -2.63335 | 0.646766 |
| ENSG00000184895 | *SRY* | 0 | -2.63218 | 0.538476 |
| ENSG00000242173 | *ARHGDIG* | 0 | -2.63095 | 2.552884 |
| ENSG00000260064 | *RP11-18F14.4* | 0.84833 | -2.62636 | 0 |
| ENSG00000206120 | *EGFEM1P* | 0.229263 | -2.62001 | 0 |
| ENSG00000166833 | *NAV2* | 0 | -2.61464 | 1.051515 |
| ENSG00000277825 | *CTD-2013N17.7* | 0 | -2.61461 | 3.252983 |
| ENSG00000243004 | *AC005062.2* | 0 | -2.61428 | 0 |
| ENSG00000250999 | *RP11-1379J22.5* | 0 | -2.61333 | 1.353801 |
| ENSG00000226548 | *AC016722.3* | 0 | -2.60881 | 4.045464 |
| ENSG00000197454 | *OR2L5* | 0.926771 | -2.607 | 0 |
| ENSG00000159842 | *ABR* | 0 | -2.60395 | 0.047423 |
| ENSG00000148426 | *PROSER2* | 0.412321 | -2.6028 | 0 |
| ENSG00000205307 | *SAP25* | 0 | -2.57304 | 1.372919 |
| ENSG00000162512 | *SDC3* | 0 | -2.57169 | 1.513304 |
| ENSG00000145428 | *RNF175* | 0 | -2.56866 | 3.042402 |
| ENSG00000258650 | *HSBP1P1* | 0 | -2.56793 | 0.568658 |
| ENSG00000261131 | *RP11-93O14.2* | 0 | -2.56752 | 1.958791 |
| ENSG00000165702 | *GFI1B* | 0.325264 | -2.55319 | 0 |
| ENSG00000130598 | *TNNI2* | 0 | -2.5529 | 0 |
| ENSG00000234665 | *RP11-262H14.3* | 0 | -2.54763 | 3.00037 |
| ENSG00000213638 | *ADAT3* | 0 | -2.53665 | 1.141601 |
| ENSG00000128165 | *ADM2* | 0 | -2.53438 | 0.611438 |
| ENSG00000186994 | *KANK3* | 0 | -2.53231 | 0.484012 |
| ENSG00000251322 | *SHANK3* | 0.206481 | -2.53126 | 0 |
| ENSG00000273493 | *RP11-80H18.4* | 0 | -2.53058 | 0 |
| ENSG00000224424 | *PRKAR2A-AS1* | 0 | -2.52353 | 1.172954 |
| ENSG00000244198 | *RP4-545C24.1* | 0 | -2.52142 | 2.768353 |
| ENSG00000188396 | *TCTEX1D4* | 0 | -2.51799 | 2.3345 |
| ENSG00000267457 | *RP5-837J1.4* | 0 | -2.5161 | 3.761123 |
| ENSG00000070614 | *NDST1* | 0 | -2.51393 | 0.097681 |
| ENSG00000268189 | *AC005785.2* | 0 | -2.50641 | 2.435706 |
| ENSG00000174059 | *CD34* | 0 | -2.5037 | 0.039164 |
| ENSG00000269476 | *CTD-2583A14.9* | 0 | -2.48872 | 2.46394 |
| ENSG00000217275 | *RP1-34B20.4* | 0 | -2.48713 | 0.005812 |
| ENSG00000271907 | *SNORA35* | 0 | -2.47909 | 0 |
| ENSG00000260179 | *RP5-902P8.12* | 0 | -2.47514 | 3.225037 |
| ENSG00000188738 | *FSIP2* | 0 | -2.47356 | 0 |
| ENSG00000278965 | *RP11-39H3.2* | 0 | -2.47163 | 0.87063 |
| ENSG00000178568 | *ERBB4* | 0 | -2.47075 | 1.043907 |
| ENSG00000105251 | *SHD* | 2.44824 | -2.4635 | 0 |
| ENSG00000279573 | *RP11-855A2.1* | 0 | -2.462 | 3.000326 |
| ENSG00000099769 | *IGFALS* | 0 | -2.45921 | 1.539603 |
| ENSG00000160145 | *KALRN* | 0 | -2.45674 | 0.726365 |
| ENSG00000188365 | *AC092171.2* | 0 | -2.4553 | 4.548041 |
| ENSG00000242798 | *RP11-506M12.1* | 0.024256 | -2.44761 | 0 |
| ENSG00000180549 | *FUT7* | 0.218324 | -2.44526 | 0 |
| ENSG00000133101 | *CCNA1* | 0 | -2.44118 | 0 |
| ENSG00000174327 | *SLC16A13* | 0 | -2.43818 | 0.086892 |
| ENSG00000268510 | *IFNL3P1* | 0 | -2.43527 | 0.87215 |
| ENSG00000130635 | *COL5A1* | 0 | -2.43215 | 2.089879 |
| ENSG00000279670 | *RP11-70L8.5* | 0 | -2.43132 | 1.645026 |
| ENSG00000234546 | *LINC01759* | 0 | -2.42751 | 2.555382 |
| ENSG00000214128 | *TMEM213* | 0 | -2.42525 | 2.405428 |
| ENSG00000161682 | *FAM171A2* | 0.037018 | -2.42466 | 0 |
| ENSG00000184163 | *C1QTNF12* | 0 | -2.42391 | 3.02882 |
| ENSG00000241269 | *AC093620.5* | 0 | -2.4214 | 4.302591 |
| ENSG00000243238 | *IGKV2-30* | 0 | -2.41595 | 0 |
| ENSG00000115884 | *SDC1* | 0 | -2.41192 | 1.733966 |
| ENSG00000133317 | *LGALS12* | 0.579906 | -2.40664 | 0 |
| ENSG00000262903 | *RP11-235E17.6* | 0 | -2.4066 | 0.263638 |
| ENSG00000221946 | *FXYD7* | 0 | -2.40599 | 5.152354 |
| ENSG00000273413 | *RP11-96C23.15* | 0 | -2.39733 | 3.95539 |
| ENSG00000152689 | *RASGRP3* | 0 | -2.39625 | 0.116799 |
| ENSG00000213316 | *LTC4S* | 0 | -2.39226 | 3.403717 |
| ENSG00000130595 | *TNNT3* | 0.019936 | -2.38833 | 0 |
| ENSG00000143036 | *SLC44A3* | 0 | -2.38694 | 0.043541 |
| ENSG00000272836 | *RP3-402G11.27* | 0.337543 | -2.38652 | 0 |
| ENSG00000187904 | *AC097382.5* | 0 | -2.38383 | 2.712143 |
| ENSG00000224977 | *RP11-160H22.3* | 0 | -2.37964 | 1.714492 |
| ENSG00000278700 | *Metazoa_SRP* | 0 | -2.37106 | 2.179637 |
| ENSG00000198208 | *RPS6KL1* | 0 | -2.37033 | 0.012003 |
| ENSG00000279035 | *RP11-649A18.3* | 0 | -2.36929 | 2.029067 |
| ENSG00000154277 | *UCHL1* | 0 | -2.36925 | 2.361266 |
| ENSG00000183496 | *MEX3B* | 0 | -2.36623 | 0.822271 |
| ENSG00000225492 | *GBP1P1* | 0 | -2.36478 | 0 |
| ENSG00000271981 | *RP11-573G6.8* | 0 | -2.36468 | 0 |
| ENSG00000174788 | *PCP2* | 0 | -2.36404 | 0.136288 |
| ENSG00000179862 | *CITED4* | 0 | -2.36285 | 3.307888 |
| ENSG00000167371 | *PRRT2* | 0 | -2.35651 | 1.675434 |
| ENSG00000278520 | *MIR7851* | 0 | -2.35507 | 2.418745 |
| ENSG00000162722 | *TRIM58* | 0 | -2.34731 | 1.147079 |
| ENSG00000152253 | *SPC25* | 0 | -2.34568 | 3.431204 |
| ENSG00000180747 | *SMG1P3* | 0 | -2.34247 | 0 |
| ENSG00000262554 | *LA16c-360H6.2* | 0 | -2.33854 | 3.62372 |
| ENSG00000275468 | *LLNLR-304A6.2* | 0 | -2.33652 | 2.044529 |
| ENSG00000087116 | *ADAMTS2* | 0 | -2.33453 | 1.572955 |
| ENSG00000181982 | *CCDC149* | 0 | -2.32831 | 0.185589 |
| ENSG00000236535 | *RC3H1-IT1* | 0 | -2.32591 | 3.551731 |
| ENSG00000274272 | *RP11-44M6.7* | 0 | -2.32454 | 2.686537 |
| ENSG00000133246 | *PRAM1* | 0 | -2.32009 | 1.167434 |
| ENSG00000241057 | *AC004985.12* | 0 | -2.31378 | 1.41686 |
| ENSG00000229508 | *PHKG1P4* | 0 | -2.31176 | 0 |
| ENSG00000256660 | *CLEC12B* | 0 | -2.30926 | 0 |
| ENSG00000231327 | *LINC01816* | 0 | -2.3033 | 0.385104 |
| ENSG00000265692 | *LINC01970* | 0 | -2.294 | 2.322826 |
| ENSG00000238243 | *OR2W3* | 0 | -2.29134 | 0.436076 |
| ENSG00000257556 | *RP11-44N21.1* | 0 | -2.29041 | 1.54859 |
| ENSG00000111676 | *ATN1* | 0 | -2.28812 | 1.695495 |
| ENSG00000276786 | *WI2-8325B5.1* | 0 | -2.28005 | 1.599768 |
| ENSG00000270610 | *RP11-486O13.3* | 0 | -2.27515 | 1.73672 |
| ENSG00000108821 | *COL1A1* | 0 | -2.27198 | 3.850282 |
| ENSG00000272057 | *CTB-40H15.4* | 0 | -2.27026 | 1.813947 |
| ENSG00000251163 | *PRELID3BP4* | 0 | -2.26771 | 3.368431 |
| ENSG00000125657 | *TNFSF9* | 0 | -2.2649 | 3.815576 |
| ENSG00000162458 | *FBLIM1* | 0 | -2.26454 | 3.209384 |
| ENSG00000201620 | *RNA5SP51* | 0 | -2.26453 | 0 |
| ENSG00000269934 | *RP5-1139B12.3* | 0 | -2.26298 | 2.366577 |
| ENSG00000130304 | *SLC27A1* | 0 | -2.25755 | 1.534653 |
| ENSG00000244242 | *IFITM10* | 0 | -2.25621 | 2.686948 |
| ENSG00000242894 | *RN7SL634P* | 0.541404 | -2.24966 | 0 |
| ENSG00000105559 | *PLEKHA4* | 0 | -2.24786 | 2.230414 |
| ENSG00000229512 | *AC068580.5* | 0 | -2.23439 | 3.384889 |
| ENSG00000225205 | *AC093818.1* | 0.37703 | -2.23144 | 0 |
| ENSG00000182601 | *HS3ST4* | 0 | -2.22329 | 6.72663 |
| ENSG00000231485 | *RP4-535B20.1* | 0 | -2.2195 | 3.385702 |
| ENSG00000174885 | *NLRP6* | 0 | -2.21483 | 4.20176 |
| ENSG00000244486 | *SCARF2* | 0 | -2.21215 | 3.152138 |
| ENSG00000280248 | *CTD-2047H16.3* | 0 | -2.21023 | 2.595294 |
| ENSG00000242073 | *AC006014.7* | 0 | -2.21019 | 2.393701 |
| ENSG00000263905 | *RN7SL555P* | 0 | -2.20895 | 2.334349 |
| ENSG00000237296 | *SMG1P1* | 0 | -2.20573 | 0 |
| ENSG00000184985 | *SORCS2* | 0 | -2.20447 | 4.009866 |
| ENSG00000254503 | *CTD-2521M24.4* | 0 | -2.19603 | 2.406552 |
| ENSG00000205309 | *NT5M* | 0 | -2.1943 | 0.510646 |
| ENSG00000184058 | *TBX1* | 0 | -2.1939 | 2.633389 |
| ENSG00000142149 | *HUNK* | 0 | -2.18794 | 3.133447 |
| ENSG00000232375 | *RP11-136C24.1* | 0 | -2.18519 | 0.574924 |
| ENSG00000269560 | *CTD-2192J16.21* | 0 | -2.17922 | 2.914354 |
| ENSG00000178821 | *TMEM52* | 0 | -2.17788 | 6.070508 |
| ENSG00000267771 | *CTC-260E6.7* | 0 | -2.17784 | 0 |
| ENSG00000279549 | *AP000437.3* | 0 | -2.17756 | 1.904931 |
| ENSG00000154655 | *L3MBTL4* | 0.372663 | -2.17428 | 0 |
| ENSG00000167600 | *CYP2S1* | 0 | -2.17292 | 3.767221 |
| ENSG00000267474 | *CTC-548K16.6* | 0 | -2.1687 | 2.085522 |
| ENSG00000205022 | *PABPN1L* | 0 | -2.16369 | 3.542525 |
| ENSG00000115155 | *OTOF* | 1.91195 | -2.16159 | 0 |
| ENSG00000150457 | *LATS2* | 0.45685 | -2.15517 | 0 |
| ENSG00000259782 | *CTD-2270L9.2* | 0 | -2.15372 | 3.61219 |
| ENSG00000121570 | *DPPA4* | 0 | -2.14621 | 0 |
| ENSG00000177363 | *LRRN4CL* | 0 | -2.14467 | 1.994568 |
| ENSG00000177191 | *B3GNT8* | 0 | -2.14465 | 0 |
| ENSG00000166126 | *AMN* | 0 | -2.14401 | 0.556644 |
| ENSG00000090661 | *CERS4* | 0 | -2.14164 | 0.373475 |
| ENSG00000101665 | *SMAD7* | 0 | -2.14116 | 0.775261 |
| ENSG00000204323 | *SMIM5* | 0.081108 | -2.13752 | 0 |
| ENSG00000198835 | *GJC2* | 0 | -2.12854 | 3.003493 |
| ENSG00000237492 | *OR2L9P* | 1.17185 | -2.12506 | 0 |
| ENSG00000181444 | *ZNF467* | 0 | -2.12467 | 2.937149 |
| ENSG00000267470 | *ZNF571-AS1* | 0 | -2.12436 | 1.707642 |
| ENSG00000135299 | *ANKRD6* | 0 | -2.12148 | 0.35496 |
| ENSG00000148488 | *ST8SIA6* | 0 | -2.12073 | 0.863122 |
| ENSG00000226942 | *IL9RP3* | 0.18761 | -2.11385 | 0 |
| ENSG00000072071 | *ADGRL1* | 0 | -2.11279 | 1.638485 |
| ENSG00000276101 | *RP11-455O6.8* | 0 | -2.10989 | 1.18634 |
| ENSG00000187134 | *AKR1C1* | 0 | -2.10981 | 3.853222 |
| ENSG00000205181 | *LINC00654* | 0 | -2.10818 | 1.48407 |
| ENSG00000239702 | *RN7SL507P* | 0 | -2.10768 | 3.403236 |
| ENSG00000087495 | *PHACTR3* | 0 | -2.10628 | 2.900119 |
| ENSG00000237422 | *RP11-305L7.3* | 0.197655 | -2.10595 | 0 |
| ENSG00000205639 | *MFSD2B* | 0 | -2.10286 | 0.645273 |
| ENSG00000126545 | *CSN1S1* | 2.927193 | -2.10068 | 0 |
| ENSG00000166900 | *STX3* | 0 | -2.09561 | 0.151637 |
| ENSG00000284179 | *MIR7-1* | 0 | -2.09452 | 0 |
| ENSG00000280474 | *RP11-216B9.8* | 0 | -2.09184 | 1.140842 |
| ENSG00000027869 | *SH2D2A* | 0 | -2.09179 | 2.364824 |
| ENSG00000128011 | *LRFN1* | 0 | -2.09147 | 1.674287 |
| ENSG00000235370 | *DNM1P51* | 0 | -2.0902 | 4.05387 |
| ENSG00000170891 | *CYTL1* | 0 | -2.0865 | 0 |
| ENSG00000121380 | *BCL2L14* | 0.697607 | -2.08636 | 0 |
| ENSG00000162591 | *MEGF6* | 0 | -2.08406 | 0.507388 |
| ENSG00000274742 | *Metazoa_SRP* | 0 | -2.08346 | 3.058687 |
| ENSG00000241484 | *ARHGAP8* | 0 | -2.08316 | 2.031284 |
| ENSG00000143473 | *KCNH1* | 0 | -2.08066 | 2.08641 |
| ENSG00000223722 | *RP11-467L13.5* | 0 | -2.07916 | 0 |
| ENSG00000159958 | *TNFRSF13C* | 0 | -2.07912 | 2.178673 |
| ENSG00000099864 | *PALM* | 0.472251 | -2.07401 | 0 |
| ENSG00000130222 | *GADD45G* | 0 | -2.06693 | 1.921604 |
| ENSG00000214960 | *ISPD* | 0.215921 | -2.06493 | 0 |
| ENSG00000250497 | *AC007126.1* | 0 | -2.0628 | 4.494678 |
| ENSG00000187653 | *TMSB4XP8* | 0 | -2.06028 | 0 |
| ENSG00000176020 | *AMIGO3* | 0 | -2.05769 | 0.057396 |
| ENSG00000258555 | *SPECC1L-ADORA2A* | 0 | -2.05566 | 0.367047 |
| ENSG00000164120 | *HPGD* | 0 | -2.05497 | 0.542999 |
| ENSG00000073282 | *TP63* | 4.394899 | -2.05405 | 0 |
| ENSG00000147883 | *CDKN2B* | 0 | -2.05393 | 4.409909 |
| ENSG00000207297 | *SNORD7* | 0 | -2.05256 | 0 |
| ENSG00000260001 | *TGFBR3L* | 0 | -2.0514 | 3.221724 |
| ENSG00000164236 | *ANKRD33B* | 0.775804 | -2.04917 | 0 |
| ENSG00000166924 | *NYAP1* | 0 | -2.04877 | 4.482997 |
| ENSG00000107438 | *PDLIM1* | 1.241178 | -2.04644 | 0 |
| ENSG00000225963 | *AC009950.2* | 0 | -2.04512 | 1.770294 |
| ENSG00000212214 | *SNORA48* | 0 | -2.04293 | 0 |
| ENSG00000130383 | *FUT5* | 0 | -2.04154 | 3.035784 |
| ENSG00000104899 | *AMH* | 0 | -2.03905 | 0.467008 |
| ENSG00000136367 | *ZFHX2* | 0.400571 | -2.03802 | 0 |
| ENSG00000174292 | *TNK1* | 0 | -2.03748 | 1.224709 |
| ENSG00000225408 | *RP11-207C16.4* | 0.163807 | -2.03573 | 0 |
| ENSG00000269821 | *KCNQ1OT1* | 0.255828 | -2.03548 | 0 |
| ENSG00000261783 | *RP11-252K23.2* | 0 | -2.03496 | 2.353242 |
| ENSG00000180422 | *LINC00304* | 0 | -2.03446 | 1.880467 |
| ENSG00000267640 | *CTD-2554C21.2* | 0 | -2.03084 | 2.853056 |
| ENSG00000166183 | *ASPG* | 2.226467 | -2.02928 | 0 |
| ENSG00000244346 | *RP11-531F16.3* | 0 | -2.02834 | 4.024825 |
| ENSG00000283208 | *RP11-226E21.4* | 0 | -2.0273 | 2.685682 |
| ENSG00000144485 | *HES6* | 0 | -2.02372 | 2.24318 |
| ENSG00000240382 | *IGKV1-17* | 0 | -2.0219 | 0 |
| ENSG00000222419 | *RNA5SP511* | 0 | -2.01454 | 0.104058 |
| ENSG00000226758 | *DISC1-IT1* | 0 | -2.01008 | 2.810057 |
| ENSG00000124493 | *GRM4* | 0 | -2.0097 | 1.534173 |
| ENSG00000167772 | *ANGPTL4* | 0 | -2.00499 | 3.992095 |
| ENSG00000140859 | *KIFC3* | 0.227095 | -2.00196 | 0 |

**Supplementary Table 3A: List of genes upregulated in mature T-ALL**

| **Ensembl_ID** | **Gene Name** | **Log_2_fold change in T-ALL subtypes** | | |
| --- | --- | --- | --- | --- |
|  |  | **Immature** | **Cortical** | **Mature** |
| ENSG00000121410 | *A1BG* | 0 | -0.97229 | 2.700006 |
| ENSG00000245105 | *A2M-AS1* | 0 | -0.47608 | 2.182901 |
| ENSG00000128274 | *A4GALT* | -0.23718 | 0 | 2.715829 |
| ENSG00000283199 | *ABC13-47488600E17.1* | 0 | -1.19638 | 2.364362 |
| ENSG00000033050 | *ABCF2* | -0.00884 | 0 | 2.235616 |
| ENSG00000172350 | *ABCG4* | -3.8771 | 0 | 3.091848 |
| ENSG00000100997 | *ABHD12* | 0 | -0.63422 | 2.130974 |
| ENSG00000163995 | *ABLIM2* | -1.05719 | 0 | 2.030675 |
| ENSG00000273300 | *AC000068.9* | -0.05591 | 0 | 2.047617 |
| ENSG00000268193 | *AC002985.3* | 0 | -0.20685 | 4.941752 |
| ENSG00000268133 | *AC003002.4* | 0 | 0 | 4.908011 |
| ENSG00000268533 | *AC004076.7* | 0 | -0.44812 | 3.354722 |
| ENSG00000269694 | *AC005197.2* | 0 | -0.86654 | 4.126352 |
| ENSG00000283269 | *AC005307.3* | 0 | -5.26691 | 3.480403 |
| ENSG00000235852 | *AC005540.3* | 0 | -0.76887 | 2.329939 |
| ENSG00000268189 | *AC005785.2* | 0 | -2.50641 | 2.435706 |
| ENSG00000279590 | *AC005786.6* | 0 | 0 | 7.216626 |
| ENSG00000267436 | *AC005786.7* | 0 | -1.08913 | 2.145805 |
| ENSG00000242073 | *AC006014.7* | 0 | -2.21019 | 2.393701 |
| ENSG00000253392 | *AC006277.2* | 0 | -0.09416 | 3.316159 |
| ENSG00000233191 | *AC006372.6* | 0 | 0 | 4.351934 |
| ENSG00000229330 | *AC006947.1* | -2.93643 | 0 | 2.176786 |
| ENSG00000250497 | *AC007126.1* | 0 | -2.0628 | 4.494678 |
| ENSG00000268352 | *AC007228.5* | -2.19999 | 0 | 4.198745 |
| ENSG00000223935 | *AC008074.3* | 0 | -1.2655 | 4.012356 |
| ENSG00000260345 | *AC009052.12* | 0 | 0 | 7.910245 |
| ENSG00000265073 | *AC010761.6* | 0 | -0.60373 | 2.469771 |
| ENSG00000283602 | *AC011380.8* | 0 | 0 | 7.508356 |
| ENSG00000226548 | *AC016722.3* | 0 | -2.60881 | 4.045464 |
| ENSG00000236039 | *AC017060.1* | -0.88059 | 0 | 3.080007 |
| ENSG00000275981 | *AC018816.1* | 0 | -0.18933 | 2.143593 |
| ENSG00000268093 | *AC022154.7* | -0.39479 | 0 | 2.451332 |
| ENSG00000233006 | *AC034220.3* | 0 | -0.44558 | 2.075992 |
| ENSG00000267737 | *AC061992.2* | 0 | -0.21925 | 3.186764 |
| ENSG00000145063 | *AC062028.1* | -0.20503 | 0 | 3.303122 |
| ENSG00000235127 | *AC068286.1* | 0 | 0 | 2.326923 |
| ENSG00000229512 | *AC068580.5* | 0 | -2.23439 | 3.384889 |
| ENSG00000229267 | *AC072062.1* | 0 | -0.85715 | 2.114643 |
| ENSG00000228010 | *AC073343.13* | 0 | -0.27412 | 2.080754 |
| ENSG00000213148 | *AC073465.1* | 0 | -1.45668 | 3.216449 |
| ENSG00000235548 | *AC073551.1* | -1.73705 | 0 | 2.550154 |
| ENSG00000235077 | *AC073842.19* | -0.36498 | 0 | 2.391005 |
| ENSG00000222043 | *AC079305.10* | 0 | -1.86175 | 2.238823 |
| ENSG00000224875 | *AC083949.1* | -0.83724 | 0 | 4.174209 |
| ENSG00000188365 | *AC092171.2* | 0 | -2.4553 | 4.548041 |
| ENSG00000229996 | *AC093585.6* | 0 | -4.86851 | 2.265696 |
| ENSG00000241269 | *AC093620.5* | 0 | -2.4214 | 4.302591 |
| ENSG00000229750 | *AC096649.2* | 0 | -0.1555 | 3.737085 |
| ENSG00000187904 | *AC097382.5* | 0 | -2.38383 | 2.712143 |
| ENSG00000259635 | *AC100830.3* | 0 | -1.03786 | 2.413571 |
| ENSG00000215692 | *AC114730.8* | 0 | -0.53082 | 5.01962 |
| ENSG00000268601 | *AC115522.3* | -1.20386 | 0 | 5.329414 |
| ENSG00000279396 | *AC130469.1* | 0 | -1.09392 | 2.617173 |
| ENSG00000176244 | *ACBD7* | -0.07335 | 0 | 2.227311 |
| ENSG00000153086 | *ACMSD* | 0 | 0 | 7.003484 |
| ENSG00000119673 | *ACOT2* | 0 | -0.32132 | 2.373858 |
| ENSG00000140955 | *ADAD2* | 0 | -0.63089 | 3.422177 |
| ENSG00000203804 | *ADAMTSL4-AS1* | 0 | -1.21024 | 2.854936 |
| ENSG00000163485 | *ADORA1* | 0 | -0.80727 | 3.166046 |
| ENSG00000234120 | *AF228730.8* | 0 | 0 | 3.386305 |
| ENSG00000196526 | *AFAP1* | 0 | -0.1079 | 2.478152 |
| ENSG00000157985 | *AGAP1* | -0.9282 | 0 | 3.903977 |
| ENSG00000187134 | *AKR1C1* | 0 | -2.10981 | 3.853222 |
| ENSG00000151632 | *AKR1C2* | 0 | -1.77973 | 4.216645 |
| ENSG00000225418 | *AKR1C5P* | 0 | 0 | 3.863018 |
| ENSG00000279336 | *AL353662.1* | 0 | -2.99331 | 3.159934 |
| ENSG00000167772 | *ANGPTL4* | 0 | -2.00499 | 3.992095 |
| ENSG00000148677 | *ANKRD1* | -0.94602 | 0 | 2.256765 |
| ENSG00000230778 | *ANKRD63* | 0 | -3.55567 | 3.349395 |
| ENSG00000156381 | *ANKRD9* | 0 | -0.77888 | 2.102152 |
| ENSG00000249209 | *AP000304.12* | 0 | -0.69428 | 2.348311 |
| ENSG00000273199 | *AP000692.10* | 0 | -1.62655 | 2.490308 |
| ENSG00000255949 | *AP003419.16* | 0 | -0.7597 | 2.465317 |
| ENSG00000115266 | *APC2* | 0 | -1.27157 | 5.12723 |
| ENSG00000147799 | *ARHGAP39* | 0 | -2.81782 | 2.190978 |
| ENSG00000241484 | *ARHGAP8* | 0 | -2.08316 | 2.031284 |
| ENSG00000242173 | *ARHGDIG* | 0 | -2.63095 | 2.552884 |
| ENSG00000076928 | *ARHGEF1* | 0 | -1.30372 | 2.42089 |
| ENSG00000214944 | *ARHGEF28* | -0.29712 | 0 | 3.200721 |
| ENSG00000214087 | *ARL16* | 0 | -0.48234 | 2.126319 |
| ENSG00000100299 | *ARSA* | 0 | -1.55216 | 2.269894 |
| ENSG00000280543 | *ASAP1-IT2* | 0 | -0.19056 | 2.296649 |
| ENSG00000088280 | *ASAP3* | 0 | -0.02826 | 2.493015 |
| ENSG00000105011 | *ASF1B* | -0.02718 | 0 | 2.379851 |
| ENSG00000148219 | *ASTN2* | 0 | -0.45659 | 3.431802 |
| ENSG00000249222 | *ATP5L2* | 0 | -0.9903 | 2.09902 |
| ENSG00000105929 | *ATP6V0A4* | -0.5702 | 0 | 3.560903 |
| ENSG00000105146 | *AURKC* | -0.06524 | 0 | 2.200595 |
| ENSG00000182272 | *B4GALNT4* | 0 | -2.80902 | 3.491756 |
| ENSG00000186318 | *BACE1* | 0 | -0.6588 | 2.052163 |
| ENSG00000112182 | *BACH2* | 0 | -1.22264 | 2.250756 |
| ENSG00000107262 | *BAG1* | 0 | -0.44004 | 2.347052 |
| ENSG00000176788 | *BASP1* | 0 | -1.50286 | 2.13285 |
| ENSG00000069399 | *BCL3* | 0 | -1.38216 | 2.352595 |
| ENSG00000189114 | *BLOC1S3* | 0 | -1.51395 | 2.141096 |
| ENSG00000270325 | *BNIP3P9* | 0 | -0.28597 | 3.347919 |
| ENSG00000270316 | *BORCS7-ASMT* | 0 | -1.82074 | 2.045326 |
| ENSG00000235120 | *BSN-AS1* | 0 | 0 | 5.936988 |
| ENSG00000222009 | *BTBD19* | 0 | -1.46909 | 2.615782 |
| ENSG00000183346 | *C10orf107* | 0 | -0.68869 | 2.683144 |
| ENSG00000279020 | *C18orf15* | 0 | -0.42907 | 2.409282 |
| ENSG00000198723 | *C19orf45* | 0 | 0 | 2.630176 |
| ENSG00000235034 | *C19orf81* | 0 | -1.03771 | 2.58496 |
| ENSG00000184163 | *C1QTNF12* | 0 | -2.42391 | 3.02882 |
| ENSG00000163009 | *C2orf48* | 0 | -0.94337 | 2.944132 |
| ENSG00000182600 | *C2orf82* | 0 | -1.71986 | 2.572808 |
| ENSG00000163689 | *C3orf67* | -0.93562 | 0 | 2.9063 |
| ENSG00000187068 | *C3orf70* | 0 | -3.59227 | 3.114511 |
| ENSG00000197405 | *C5AR1* | 0 | -1.90969 | 2.441837 |
| ENSG00000186354 | *C9orf47* | 0 | -1.32748 | 2.250129 |
| ENSG00000241527 | *CA15P1* | 0 | -1.53044 | 2.137556 |
| ENSG00000175544 | *CABP4* | 0 | -0.47992 | 2.510638 |
| ENSG00000102001 | *CACNA1F* | -0.21585 | 0 | 3.86055 |
| ENSG00000196557 | *CACNA1H* | 0 | -3.48528 | 2.466297 |
| ENSG00000226800 | *CACTIN-AS1* | 0 | -3.48233 | 2.578792 |
| ENSG00000175868 | *CALCB* | 0 | -0.89519 | 3.128847 |
| ENSG00000183166 | *CALN1* | 0 | -0.11998 | 2.103117 |
| ENSG00000268001 | *CARD8-AS1* | 0 | -0.90247 | 2.003866 |
| ENSG00000236830 | *CBR3-AS1* | 0 | -1.38629 | 2.149167 |
| ENSG00000100307 | *CBX7* | 0 | -0.86189 | 2.405276 |
| ENSG00000161180 | *CCDC116* | -0.12104 | 0 | 2.898339 |
| ENSG00000187860 | *CCDC157* | 0 | -0.3608 | 2.10402 |
| ENSG00000198624 | *CCDC69* | 0 | -0.91554 | 3.113428 |
| ENSG00000110092 | *CCND1* | -0.59371 | 0 | 4.814916 |
| ENSG00000135083 | *CCNJL* | 0 | -1.17773 | 2.06085 |
| ENSG00000184451 | *CCR10* | 0 | -0.40508 | 3.574364 |
| ENSG00000238241 | *CCR12P* | 0 | -0.93113 | 3.753594 |
| ENSG00000183813 | *CCR4* | -0.27754 | 0 | 3.563653 |
| ENSG00000112486 | *CCR6* | 0 | -2.7164 | 3.275166 |
| ENSG00000174807 | *CD248* | 0 | -1.11255 | 2.110396 |
| ENSG00000013725 | *CD6* | 0 | -1.16779 | 2.257531 |
| ENSG00000230068 | *CDC42-IT1* | 0 | -1.24527 | 2.763748 |
| ENSG00000184661 | *CDCA2* | -0.26962 | 0 | 2.134648 |
| ENSG00000139880 | *CDH24* | 0 | -1.61513 | 2.026442 |
| ENSG00000124762 | *CDKN1A* | 0 | -0.69149 | 2.407386 |
| ENSG00000147889 | *CDKN2A* | -0.40147 | 0 | 3.941634 |
| ENSG00000147883 | *CDKN2B* | 0 | -2.05393 | 4.409909 |
| ENSG00000231561 | *CEACAMP5* | 0 | -0.12764 | 4.161213 |
| ENSG00000147457 | *CHMP7* | 0 | -0.00297 | 2.337847 |
| ENSG00000117971 | *CHRNB4* | -2.00834 | 0 | 3.289374 |
| ENSG00000179862 | *CITED4* | 0 | -2.36285 | 3.307888 |
| ENSG00000188603 | *CLN3* | 0 | -0.83031 | 2.352454 |
| ENSG00000175416 | *CLTB* | 0 | -0.45342 | 2.308772 |
| ENSG00000150656 | *CNDP1* | 0 | -1.8573 | 3.21313 |
| ENSG00000174871 | *CNIH2* | 0 | -0.26928 | 2.421957 |
| ENSG00000144580 | *CNOT9* | 0 | -0.44424 | 2.357271 |
| ENSG00000108797 | *CNTNAP1* | -0.18518 | 0 | 2.486845 |
| ENSG00000182871 | *COL18A1* | 0 | -1.72134 | 3.016024 |
| ENSG00000108821 | *COL1A1* | 0 | -2.27198 | 3.850282 |
| ENSG00000050767 | *COL23A1* | 0 | -0.46371 | 5.126272 |
| ENSG00000188153 | *COL4A5* | -0.44865 | 0 | 2.398805 |
| ENSG00000130635 | *COL5A1* | 0 | -2.43215 | 2.089879 |
| ENSG00000080573 | *COL5A3* | -0.32103 | 0 | 2.621121 |
| ENSG00000142156 | *COL6A1* | 0 | -0.64384 | 4.675232 |
| ENSG00000142173 | *COL6A2* | 0 | -0.15331 | 5.934171 |
| ENSG00000092758 | *COL9A3* | 0 | -1.90292 | 2.792999 |
| ENSG00000172725 | *CORO1B* | 0 | -0.66127 | 2.153403 |
| ENSG00000168993 | *CPLX1* | 0 | -1.21764 | 5.813602 |
| ENSG00000135678 | *CPM* | 0 | -0.03658 | 2.556543 |
| ENSG00000213145 | *CRIP1* | 0 | -0.362 | 2.094533 |
| ENSG00000182351 | *CRIP1P4* | 0 | -0.3503 | 3.857991 |
| ENSG00000160741 | *CRTC2* | 0 | -0.50473 | 2.024149 |
| ENSG00000141551 | *CSNK1D* | 0 | -0.63801 | 2.133408 |
| ENSG00000114646 | *CSPG5* | 0 | -0.74227 | 2.80142 |
| ENSG00000227519 | *CTA-342B11.1* | 0 | -1.79043 | 3.848225 |
| ENSG00000249593 | *CTB-46B19.2* | 0 | -0.5867 | 3.867698 |
| ENSG00000277450 | *CTB-96E2.10* | -0.1721 | 0 | 2.217383 |
| ENSG00000267598 | *CTC-250I14.6* | 0 | -2.8204 | 3.022683 |
| ENSG00000269814 | *CTC-273B12.10* | 0 | -0.56412 | 2.649673 |
| ENSG00000274114 | *CTC-281F24.3* | 0 | -1.20402 | 2.206473 |
| ENSG00000248103 | *CTC-338M12.9* | 0 | -1.44486 | 2.692475 |
| ENSG00000268401 | *CTC-344H19.4* | 0 | -0.22635 | 2.136107 |
| ENSG00000269044 | *CTC-429P9.3* | 0 | -0.83723 | 2.100043 |
| ENSG00000268987 | *CTC-435M10.10* | 0 | 0 | 3.569654 |
| ENSG00000253424 | *CTC-436K13.3* | -0.37688 | 0 | 2.911644 |
| ENSG00000267827 | *CTC-471J1.8* | -0.38768 | 0 | 2.124569 |
| ENSG00000267550 | *CTC-482H14.5* | 0 | -1.72741 | 3.75745 |
| ENSG00000272869 | *CTC-487M23.8* | 0 | -0.82879 | 2.179122 |
| ENSG00000279108 | *CTC-490E21.11* | 0 | -1.30135 | 2.430244 |
| ENSG00000266922 | *CTC-499B15.6* | 0 | -1.06498 | 2.830786 |
| ENSG00000267474 | *CTC-548K16.6* | 0 | -2.1687 | 2.085522 |
| ENSG00000219665 | *CTD-2006C1.2* | 0 | -0.33014 | 2.596514 |
| ENSG00000277825 | *CTD-2013N17.7* | 0 | -2.61461 | 3.252983 |
| ENSG00000274383 | *CTD-2017F17.2* | 0 | -1.3934 | 4.378356 |
| ENSG00000267121 | *CTD-2020K17.1* | 0 | -1.06985 | 3.082201 |
| ENSG00000260892 | *CTD-2026K11.1* | -1.59521 | 0 | 4.639649 |
| ENSG00000280248 | *CTD-2047H16.3* | 0 | -2.21023 | 2.595294 |
| ENSG00000259031 | *CTD-2062F14.3* | 0 | -3.54159 | 2.297482 |
| ENSG00000267261 | *CTD-2132N18.3* | -0.52227 | 0 | 2.744019 |
| ENSG00000267406 | *CTD-2189E23.2* | 0 | 0 | 3.661934 |
| ENSG00000269560 | *CTD-2192J16.21* | 0 | -2.17922 | 2.914354 |
| ENSG00000260751 | *CTD-2196E14.6* | 0 | -1.15598 | 3.410193 |
| ENSG00000260482 | *CTD-2196E14.9* | 0 | -5.50426 | 2.823506 |
| ENSG00000259782 | *CTD-2270L9.2* | 0 | -2.15372 | 3.61219 |
| ENSG00000279901 | *CTD-2270P14.2* | 0 | -0.46648 | 2.04767 |
| ENSG00000267672 | *CTD-2293H3.1* | 0 | -0.78787 | 2.789307 |
| ENSG00000279757 | *CTD-2326C4.1* | 0 | -0.76096 | 2.553083 |
| ENSG00000265791 | *CTD-2349P21.10* | 0 | -1.0677 | 2.601267 |
| ENSG00000280069 | *CTD-2349P21.3* | 0 | -0.41868 | 2.679428 |
| ENSG00000279400 | *CTD-2353F22.2* | -0.13505 | 0 | 4.417184 |
| ENSG00000176593 | *CTD-2368P22.1* | 0 | -0.30137 | 2.815212 |
| ENSG00000271714 | *CTD-2377O17.1* | -0.2068 | 0 | 3.57912 |
| ENSG00000274904 | *CTD-2515O10.5* | 0 | -1.04229 | 2.190647 |
| ENSG00000254503 | *CTD-2521M24.4* | 0 | -2.19603 | 2.406552 |
| ENSG00000267096 | *CTD-2537I9.13* | 0 | -0.9305 | 2.557585 |
| ENSG00000267054 | *CTD-2540B15.10* | 0 | 0 | 3.622859 |
| ENSG00000279812 | *CTD-2545G14.4* | 0 | -0.36536 | 5.718843 |
| ENSG00000279329 | *CTD-2553L13.9* | 0 | -0.49407 | 2.131864 |
| ENSG00000267640 | *CTD-2554C21.2* | 0 | -2.03084 | 2.853056 |
| ENSG00000259230 | *CTD-2555C10.3* | 0 | -3.05135 | 3.564538 |
| ENSG00000268119 | *CTD-2561J22.5* | 0 | -0.46629 | 2.966955 |
| ENSG00000268375 | *CTD-2568A17.8* | 0 | -0.4244 | 2.547483 |
| ENSG00000268750 | *CTD-2583A14.10* | 0 | -0.67465 | 2.432233 |
| ENSG00000269476 | *CTD-2583A14.9* | 0 | -2.48872 | 2.46394 |
| ENSG00000260066 | *CTD-2587M23.1* | 0 | -0.40786 | 4.822943 |
| ENSG00000280067 | *CTD-2600H12.2* | 0 | -0.65623 | 2.402503 |
| ENSG00000268230 | *CTD-2619J13.8* | 0 | -0.35702 | 3.414407 |
| ENSG00000260366 | *CTD-2639E6.4* | 0 | -0.76382 | 2.33079 |
| ENSG00000274460 | *CTD-2649C14.2* | 0 | -0.29653 | 2.101599 |
| ENSG00000278972 | *CTD-3022L24.1* | 0 | -0.08395 | 2.106515 |
| ENSG00000268970 | *CTD-3099C6.11* | -0.03279 | 0 | 4.155412 |
| ENSG00000269680 | *CTD-3128G10.6* | 0 | -1.41055 | 3.863813 |
| ENSG00000273189 | *CTD-3148I10.15* | 0 | -0.71451 | 2.31787 |
| ENSG00000232442 | *CTD-3184A7.4* | 0 | -0.09718 | 3.053504 |
| ENSG00000269399 | *CTD-3222D19.12* | 0 | -0.96353 | 2.055798 |
| ENSG00000267519 | *CTD-3252C9.4* | 0 | -1.74757 | 3.189255 |
| ENSG00000150281 | *CTF1* | 0 | -3.54494 | 2.682881 |
| ENSG00000107562 | *CXCL12* | 0 | -0.38595 | 2.32672 |
| ENSG00000186810 | *CXCR3* | 0 | -0.88277 | 2.719531 |
| ENSG00000167600 | *CYP2S1* | 0 | -2.17292 | 3.767221 |
| ENSG00000186529 | *CYP4F3* | 0 | -0.73381 | 2.460429 |
| ENSG00000231784 | *DBIL5P* | -0.10501 | 0 | 2.949812 |
| ENSG00000132017 | *DCAF15* | 0 | -1.15814 | 2.585536 |
| ENSG00000226758 | *DISC1-IT1* | 0 | -2.01008 | 2.810057 |
| ENSG00000186047 | *DLEU7* | -0.56578 | 0 | 4.298716 |
| ENSG00000158856 | *DMTN* | 0 | -1.64393 | 2.35603 |
| ENSG00000236756 | *DNAJC9-AS1* | 0 | -0.05181 | 3.559547 |
| ENSG00000235370 | *DNM1P51* | 0 | -2.0902 | 4.05387 |
| ENSG00000129932 | *DOHH* | 0 | -0.69013 | 2.040516 |
| ENSG00000232542 | *DPYD-IT1* | 0 | -0.34566 | 2.043331 |
| ENSG00000079393 | *DUSP13* | 0 | -1.31788 | 4.190079 |
| ENSG00000121310 | *ECHDC2* | -0.56219 | 0 | 2.038035 |
| ENSG00000130159 | *ECSIT* | 0 | -0.66743 | 2.390676 |
| ENSG00000180385 | *EMC3-AS1* | 0 | -0.45542 | 2.763986 |
| ENSG00000197774 | *EME2* | 0 | -0.6611 | 2.366213 |
| ENSG00000149499 | *EML3* | 0 | -0.7782 | 2.166527 |
| ENSG00000143924 | *EML4* | -0.65853 | 0 | 2.32552 |
| ENSG00000105131 | *EPHX3* | 0 | -0.11816 | 4.340696 |
| ENSG00000240755 | *ERLEC1P1* | 0 | -0.43016 | 2.683175 |
| ENSG00000179044 | *EXOC3L1* | 0 | -1.95381 | 2.868295 |
| ENSG00000277150 | *F8A3* | 0 | -0.85177 | 3.262689 |
| ENSG00000131944 | *FAAP24* | 0 | -0.16934 | 2.805491 |
| ENSG00000203950 | *FAM127B* | 0 | -0.70819 | 2.330311 |
| ENSG00000109794 | *FAM149A* | -0.57742 | 0 | 3.27798 |
| ENSG00000219626 | *FAM228B* | 0 | -0.50051 | 2.35248 |
| ENSG00000274026 | *FAM27E3* | 0 | -0.31128 | 2.027198 |
| ENSG00000101447 | *FAM83D* | -0.15337 | 0 | 2.226902 |
| ENSG00000164845 | *FAM86FP* | -0.48238 | 0 | 2.6635 |
| ENSG00000221829 | *FANCG* | 0 | -0.15399 | 2.257807 |
| ENSG00000162458 | *FBLIM1* | 0 | -2.26454 | 3.209384 |
| ENSG00000127452 | *FBXL12* | 0 | -0.69739 | 2.950553 |
| ENSG00000107872 | *FBXL15* | 0 | -1.57085 | 2.127803 |
| ENSG00000269190 | *FBXO17* | -2.38827 | 0 | 4.100089 |
| ENSG00000105701 | *FKBP8* | 0 | -1.68798 | 2.773593 |
| ENSG00000181027 | *FKRP* | 0 | -0.80124 | 2.377132 |
| ENSG00000232774 | *FLJ22447* | 0 | -0.58454 | 2.552737 |
| ENSG00000090554 | *FLT3LG* | 0 | -0.55037 | 2.411281 |
| ENSG00000204060 | *FOXO6* | 0 | -2.77516 | 2.45164 |
| ENSG00000169933 | *FRMPD4* | -1.21739 | 0 | 2.22786 |
| ENSG00000106328 | *FSCN3* | 0 | -0.80464 | 5.545338 |
| ENSG00000053108 | *FSTL4* | 0 | -0.91813 | 3.132169 |
| ENSG00000157353 | *FUK* | 0 | -0.98069 | 2.564097 |
| ENSG00000176920 | *FUT2* | 0 | 0 | 7.207056 |
| ENSG00000130383 | *FUT5* | 0 | -2.04154 | 3.035784 |
| ENSG00000221946 | *FXYD7* | 0 | -2.40599 | 5.152354 |
| ENSG00000268089 | *GABRQ* | -2.08518 | 0 | 7.001033 |
| ENSG00000266835 | *GAPLINC* | 0 | -2.86189 | 2.801419 |
| ENSG00000244300 | *GATA2-AS1* | 0 | -1.373 | 3.043391 |
| ENSG00000130513 | *GDF15* | 0 | -1.115 | 2.336587 |
| ENSG00000123159 | *GIPC1* | 0 | -0.54452 | 2.089898 |
| ENSG00000198835 | *GJC2* | 0 | -2.12854 | 3.003493 |
| ENSG00000076716 | *GPC4* | 0 | -0.30161 | 4.334685 |
| ENSG00000178015 | *GPR150* | 0 | -0.8487 | 2.791971 |
| ENSG00000244510 | *GS1-124K5.7* | 0 | 0 | 4.768798 |
| ENSG00000272115 | *GS1-393G12.14* | -0.92063 | 0 | 5.472844 |
| ENSG00000230267 | *HERC2P4* | -0.25602 | 0 | 2.56021 |
| ENSG00000144485 | *HES6* | 0 | -2.02372 | 2.24318 |
| ENSG00000179111 | *HES7* | 0 | -0.94755 | 2.634917 |
| ENSG00000275663 | *HIST1H4G* | 0 | -0.79106 | 3.742905 |
| ENSG00000240929 | *HIST2H2BB* | -0.83983 | 0 | 2.845563 |
| ENSG00000239718 | *HLTF-AS1* | 0 | 0 | 6.063321 |
| ENSG00000150540 | *HNMT* | 0 | -0.37298 | 2.199137 |
| ENSG00000276771 | *HOTAIRM1_2* | 0 | 0 | 2.648697 |
| ENSG00000105707 | *HPN* | 0 | -1.30133 | 7.457321 |
| ENSG00000182601 | *HS3ST4* | 0 | -2.22329 | 6.72663 |
| ENSG00000167733 | *HSD11B1L* | 0 | -1.43642 | 3.467016 |
| ENSG00000258153 | *HSPE1P4* | 0 | -0.99066 | 4.291948 |
| ENSG00000142149 | *HUNK* | 0 | -2.18794 | 3.133447 |
| ENSG00000114378 | *HYAL1* | 0 | -1.6267 | 2.36608 |
| ENSG00000231908 | *IDH1-AS1* | 0 | -0.04523 | 2.935421 |
| ENSG00000244242 | *IFITM10* | 0 | -2.25621 | 2.686948 |
| ENSG00000159128 | *IFNGR2* | 0 | -0.83437 | 2.29537 |
| ENSG00000146674 | *IGFBP3* | 0 | -1.44829 | 2.283448 |
| ENSG00000260048 | *IGHV1OR16-3* | 0 | 0 | 5.928855 |
| ENSG00000080854 | *IGSF9B* | -0.52681 | 0 | 2.651644 |
| ENSG00000103522 | *IL21R* | 0 | -0.91529 | 2.036318 |
| ENSG00000162594 | *IL23R* | 0 | -3.66429 | 3.871252 |
| ENSG00000104951 | *IL4I1* | 0 | -0.83114 | 2.234476 |
| ENSG00000224975 | *INE1* | 0 | -1.52724 | 2.210383 |
| ENSG00000106012 | *IQCE* | 0 | -0.43324 | 2.280503 |
| ENSG00000256902 | *IQSEC3P1* | -0.55464 | 0 | 3.704281 |
| ENSG00000184414 | *IRS3P* | 0 | -2.84016 | 2.065694 |
| ENSG00000132470 | *ITGB4* | 0 | -0.10689 | 2.784487 |
| ENSG00000260231 | *JHDM1D-AS1* | 0 | -0.43593 | 2.125538 |
| ENSG00000274430 | *JPX_1* | 0 | -0.32382 | 2.478818 |
| ENSG00000167476 | *JSRP1* | 0 | -0.30353 | 2.921888 |
| ENSG00000131398 | *KCNC3* | 0 | -1.50423 | 2.741995 |
| ENSG00000143473 | *KCNH1* | 0 | -2.08066 | 2.08641 |
| ENSG00000055118 | *KCNH2* | 0 | -0.55884 | 2.557248 |
| ENSG00000276015 | *KCNQ1OT1_5* | 0 | -0.90258 | 2.49617 |
| ENSG00000184156 | *KCNQ3* | 0 | -1.97813 | 2.779627 |
| ENSG00000117013 | *KCNQ4* | 0 | -0.08035 | 2.65031 |
| ENSG00000135835 | *KIAA1614* | 0 | -0.88337 | 3.690995 |
| ENSG00000131650 | *KREMEN2* | -0.59351 | 0 | 3.199689 |
| ENSG00000256937 | *KRT17P8* | 0 | -0.97971 | 2.920254 |
| ENSG00000172867 | *KRT2* | -0.00572 | 0 | 2.915779 |
| ENSG00000279930 | *LA16c-312E8.4* | 0 | -1.27659 | 3.391542 |
| ENSG00000284395 | *LA16c-312E8.5* | 0 | -0.15821 | 4.609305 |
| ENSG00000260425 | *LA16c-316G12.2* | 0 | -0.21481 | 2.753257 |
| ENSG00000262554 | *LA16c-360H6.2* | 0 | -2.33854 | 3.62372 |
| ENSG00000261641 | *LA16c-390E6.5* | 0 | -1.83893 | 4.651184 |
| ENSG00000282907 | *LA16c-407A10.3* | 0 | -3.91383 | 2.277519 |
| ENSG00000268836 | *LA16c-OS12.2* | -0.28744 | 0 | 3.198653 |
| ENSG00000166173 | *LARP6* | 0 | -1.18206 | 3.17778 |
| ENSG00000172954 | *LCLAT1* | 0 | -0.20994 | 2.677656 |
| ENSG00000184925 | *LCN12* | 0 | -0.08247 | 2.547663 |
| ENSG00000107187 | *LHX3* | 0 | -1.81569 | 4.025907 |
| ENSG00000196421 | *LINC00176* | 0 | -1.23013 | 2.114497 |
| ENSG00000248360 | *LINC00504* | 0 | -1.50922 | 2.912157 |
| ENSG00000263874 | *LINC00672* | 0 | -0.80548 | 2.084476 |
| ENSG00000242759 | *LINC00882* | 0 | -1.19965 | 3.00834 |
| ENSG00000226091 | *LINC00937* | 0 | -1.4723 | 4.507723 |
| ENSG00000282508 | *LINC01002* | 0 | -0.98476 | 2.891969 |
| ENSG00000182648 | *LINC01006* | 0 | -0.37071 | 4.097029 |
| ENSG00000234199 | *LINC01191* | 0 | -1.84348 | 2.543899 |
| ENSG00000233806 | *LINC01237* | -0.4208 | 0 | 2.273704 |
| ENSG00000229891 | *LINC01315* | 0 | -0.89122 | 3.784676 |
| ENSG00000223704 | *LINC01422* | 0 | -0.71332 | 2.220708 |
| ENSG00000225640 | *LINC01729* | 0 | 0 | 5.455302 |
| ENSG00000234546 | *LINC01759* | 0 | -2.42751 | 2.555382 |
| ENSG00000232164 | *LINC01873* | 0 | -0.73711 | 6.646755 |
| ENSG00000234183 | *LINC01952* | 0 | -0.77856 | 2.173898 |
| ENSG00000248473 | *LINC01962* | 0 | 0 | 5.266793 |
| ENSG00000265692 | *LINC01970* | 0 | -2.294 | 2.322826 |
| ENSG00000204277 | *LINC01993* | 0 | -0.65092 | 3.170738 |
| ENSG00000267321 | *LINC02001* | 0 | -0.22221 | 2.029416 |
| ENSG00000273356 | *LINC02019* | 0 | -1.04426 | 2.038513 |
| ENSG00000248489 | *LINC02062* | -0.06537 | 0 | 2.080925 |
| ENSG00000273218 | *LLNLR-246C6.1* | 0 | -4.13098 | 3.261759 |
| ENSG00000274177 | *LLNLR-284B4.1* | 0 | 0 | 5.468407 |
| ENSG00000279653 | *LLNLR-304A6.1* | 0 | -1.97835 | 3.257572 |
| ENSG00000275468 | *LLNLR-304A6.2* | 0 | -2.33652 | 2.044529 |
| ENSG00000160789 | *LMNA* | 0 | -0.10331 | 2.268242 |
| ENSG00000261105 | *LMO7-AS1* | -0.39368 | 0 | 4.544133 |
| ENSG00000142235 | *LMTK3* | 0 | -4.20212 | 2.747916 |
| ENSG00000175556 | *LONRF3* | 0 | -0.12678 | 4.278392 |
| ENSG00000126243 | *LRFN3* | 0 | -0.77712 | 2.036837 |
| ENSG00000105699 | *LSR* | 0 | -0.95837 | 3.915343 |
| ENSG00000213316 | *LTC4S* | 0 | -2.39226 | 3.403717 |
| ENSG00000269153 | *LYPLA2P2* | 0 | -0.39398 | 2.560276 |
| ENSG00000154545 | *MAGED4* | 0 | -0.24489 | 2.711454 |
| ENSG00000076984 | *MAP2K7* | 0 | -0.70152 | 2.353039 |
| ENSG00000116871 | *MAP7D1* | 0 | -0.78066 | 2.453244 |
| ENSG00000116141 | *MARK1* | -0.49434 | 0 | 9.042913 |
| ENSG00000186056 | *MATN1-AS1* | 0 | -1.26314 | 2.178138 |
| ENSG00000214309 | *MBLAC1* | 0 | -0.67116 | 2.63264 |
| ENSG00000076706 | *MCAM* | -0.72393 | 0 | 2.713704 |
| ENSG00000213999 | *MEF2B* | 0 | -0.26423 | 3.570137 |
| ENSG00000214548 | *MEG3* | -0.99243 | 0 | 2.565863 |
| ENSG00000275041 | *Metazoa_SRP* | -1.49621 | 0 | 5.657 |
| ENSG00000277635 | *Metazoa_SRP* | 0 | 0 | 5.3292 |
| ENSG00000274008 | *Metazoa_SRP* | 0 | -0.96865 | 4.28733 |
| ENSG00000274799 | *Metazoa_SRP* | 0 | -3.03287 | 3.99633 |
| ENSG00000273913 | *Metazoa_SRP* | 0 | -0.86259 | 3.743168 |
| ENSG00000268154 | *Metazoa_SRP* | 0 | -0.89718 | 3.553622 |
| ENSG00000197334 | *Metazoa_SRP* | 0 | -1.63568 | 3.283662 |
| ENSG00000275901 | *Metazoa_SRP* | 0 | 0 | 3.210023 |
| ENSG00000274742 | *Metazoa_SRP* | 0 | -2.08346 | 3.058687 |
| ENSG00000278732 | *Metazoa_SRP* | 0 | 0 | 3.055065 |
| ENSG00000203286 | *Metazoa_SRP* | 0 | -3.45568 | 2.974477 |
| ENSG00000276645 | *Metazoa_SRP* | 0 | -1.23808 | 2.828822 |
| ENSG00000277958 | *Metazoa_SRP* | 0 | -2.91286 | 2.738781 |
| ENSG00000275070 | *Metazoa_SRP* | 0 | -1.8267 | 2.708238 |
| ENSG00000278700 | *Metazoa_SRP* | 0 | -2.37106 | 2.179637 |
| ENSG00000276746 | *Metazoa_SRP* | -0.38147 | 0 | 2.003894 |
| ENSG00000138111 | *MFSD13A* | 0 | -0.11429 | 2.117309 |
| ENSG00000278404 | *MIAT_exon1* | 0 | 0 | 11.13972 |
| ENSG00000143409 | *MINDY1* | 0 | -1.13746 | 3.08726 |
| ENSG00000254349 | *MIR2052HG* | 0 | -0.23844 | 2.550005 |
| ENSG00000284324 | *MIR3175* | 0 | 0 | 2.05511 |
| ENSG00000228526 | *MIR34AHG* | 0 | -3.25151 | 2.001922 |
| ENSG00000284503 | *MIR3652* | 0 | 0 | 2.515518 |
| ENSG00000280968 | *MIR3653* | 0 | -2.72777 | 2.570892 |
| ENSG00000221333 | *MIR548K* | 0 | 0 | 3.213584 |
| ENSG00000207626 | *MIR562* | 0 | 0 | 3.22543 |
| ENSG00000207697 | *MIR573* | -0.17126 | 0 | 2.497439 |
| ENSG00000207693 | *MIR602* | 0 | 0 | 5.254119 |
| ENSG00000227195 | *MIR663AHG* | 0 | -3.34015 | 2.387096 |
| ENSG00000274932 | *MIR7150* | 0 | -1.73497 | 2.058182 |
| ENSG00000278520 | *MIR7851* | 0 | -2.35507 | 2.418745 |
| ENSG00000100985 | *MMP9* | 0 | -1.29731 | 2.486775 |
| ENSG00000070444 | *MNT* | 0 | -0.95039 | 2.293158 |
| ENSG00000116151 | *MORN1* | 0 | -0.32271 | 2.13504 |
| ENSG00000139714 | *MORN3* | -0.38044 | 0 | 2.282558 |
| ENSG00000066382 | *MPPED2* | -2.1259 | 0 | 2.282971 |
| ENSG00000256508 | *MRGPRF-AS1* | 0 | 0 | 8.366026 |
| ENSG00000102854 | *MSLN* | 0 | 0 | 5.385233 |
| ENSG00000120832 | *MTERF2* | 0 | -0.20108 | 2.539846 |
| ENSG00000229088 | *MTND1P10* | 0 | 0 | 4.732894 |
| ENSG00000226543 | *MYL6P1* | 0 | -1.78069 | 3.518641 |
| ENSG00000176658 | *MYO1D* | 0 | -0.27831 | 2.143607 |
| ENSG00000103174 | *NAGPA* | 0 | -0.77576 | 2.075584 |
| ENSG00000090971 | *NAT14* | 0 | -0.70632 | 2.648688 |
| ENSG00000225578 | *NCBP2-AS1* | -0.17336 | 0 | 3.300678 |
| ENSG00000196498 | *NCOR2* | 0 | -1.59008 | 2.562206 |
| ENSG00000049759 | *NEDD4L* | 0 | -0.04867 | 2.038389 |
| ENSG00000077150 | *NFKB2* | 0 | -0.89077 | 2.620068 |
| ENSG00000174885 | *NLRP6* | 0 | -2.21483 | 4.20176 |
| ENSG00000103024 | *NME3* | 0 | -0.73258 | 2.077158 |
| ENSG00000198929 | *NOS1AP* | -1.15424 | 0 | 4.1033 |
| ENSG00000107281 | *NPDC1* | 0 | -0.93375 | 2.29287 |
| ENSG00000131697 | *NPHP4* | 0 | -0.88564 | 5.03113 |
| ENSG00000183971 | *NPW* | 0 | -1.95346 | 2.347555 |
| ENSG00000126368 | *NR1D1* | -0.94832 | 0 | 2.340403 |
| ENSG00000154146 | *NRGN* | 0 | -1.57971 | 3.402993 |
| ENSG00000165802 | *NSMF* | 0 | -0.6963 | 2.673988 |
| ENSG00000101188 | *NTSR1* | 0 | -1.0691 | 2.682099 |
| ENSG00000167693 | *NXN* | 0 | -0.0565 | 2.523455 |
| ENSG00000166924 | *NYAP1* | 0 | -2.04877 | 4.482997 |
| ENSG00000184232 | *OAF* | 0 | -0.79803 | 2.329363 |
| ENSG00000122417 | *ODF2L* | 0 | -0.01512 | 2.175502 |
| ENSG00000125741 | *OPA3* | 0 | -0.47452 | 2.236528 |
| ENSG00000188124 | *OR2AG2* | -0.65106 | 0 | 3.630466 |
| ENSG00000242180 | *OR51B5* | 0 | -1.59741 | 3.520489 |
| ENSG00000149716 | *ORAOV1* | 0 | -0.34905 | 2.024603 |
| ENSG00000253559 | *OSGEPL1-AS1* | 0 | -1.09865 | 2.251523 |
| ENSG00000248668 | *OXCT1-AS1* | -0.28577 | 0 | 3.615569 |
| ENSG00000205022 | *PABPN1L* | 0 | -2.16369 | 3.542525 |
| ENSG00000280789 | *PAGR1* | 0 | -1.12368 | 2.052313 |
| ENSG00000099260 | *PALMD* | -1.74039 | 0 | 3.568512 |
| ENSG00000253438 | *PCAT1* | 0 | -1.24767 | 2.927534 |
| ENSG00000280623 | *PCAT14* | -1.80705 | 0 | 3.511927 |
| ENSG00000165194 | *PCDH19* | -2.15481 | 0 | 4.894485 |
| ENSG00000106333 | *PCOLCE* | 0 | -0.02671 | 3.000289 |
| ENSG00000102109 | *PCSK1N* | 0 | -3.05117 | 5.540218 |
| ENSG00000261377 | *PDCD6IPP2* | 0 | -0.45229 | 2.111967 |
| ENSG00000112541 | *PDE10A* | -0.73508 | 0 | 2.230311 |
| ENSG00000186642 | *PDE2A* | 0 | -0.36692 | 2.540115 |
| ENSG00000255185 | *PDXDC2P* | 0 | -0.68972 | 2.098782 |
| ENSG00000067840 | *PDZD4* | 0 | -0.79479 | 3.687282 |
| ENSG00000179094 | *PER1* | 0 | -0.98265 | 3.176543 |
| ENSG00000049246 | *PER3* | 0 | -0.56455 | 2.319176 |
| ENSG00000087495 | *PHACTR3* | 0 | -2.10628 | 2.900119 |
| ENSG00000174307 | *PHLDA3* | 0 | -1.2778 | 2.405565 |
| ENSG00000254681 | *PKD1P5* | 0 | -1.49674 | 4.910288 |
| ENSG00000123143 | *PKN1* | 0 | -1.39359 | 2.214889 |
| ENSG00000103066 | *PLA2G15* | 0 | -0.80767 | 2.840856 |
| ENSG00000105499 | *PLA2G4C* | 0 | -1.21684 | 2.064288 |
| ENSG00000105559 | *PLEKHA4* | 0 | -2.24786 | 2.230414 |
| ENSG00000102934 | *PLLP* | 0 | -0.55285 | 3.011652 |
| ENSG00000141934 | *PLPP2* | 0 | -1.26539 | 2.34666 |
| ENSG00000187838 | *PLSCR3* | 0 | -0.70479 | 2.417325 |
| ENSG00000130300 | *PLVAP* | 0 | -2.64475 | 3.310543 |
| ENSG00000076356 | *PLXNA2* | 0 | -0.84061 | 2.011196 |
| ENSG00000198753 | *PLXNB3* | -0.7446 | 0 | 2.317684 |
| ENSG00000130822 | *PNCK* | 0 | -0.99232 | 3.269822 |
| ENSG00000104866 | *PPP1R37* | 0 | -1.44018 | 2.528184 |
| ENSG00000049769 | *PPP1R3F* | 0 | -1.51926 | 2.192317 |
| ENSG00000251163 | *PRELID3BP4* | 0 | -2.26771 | 3.368431 |
| ENSG00000170955 | *PRKCDBP* | -0.41391 | 0 | 4.92913 |
| ENSG00000259205 | *PRKXP1* | -0.15235 | 0 | 2.154952 |
| ENSG00000117707 | *PROX1* | -0.45174 | 0 | 5.286907 |
| ENSG00000224383 | *PRR29* | 0 | -1.16992 | 3.633086 |
| ENSG00000183248 | *PRR36* | 0 | -5.83826 | 3.069558 |
| ENSG00000131188 | *PRR7* | 0 | -0.81707 | 2.409359 |
| ENSG00000007038 | *PRSS21* | -1.79427 | 0 | 2.017345 |
| ENSG00000156011 | *PSD3* | 0 | -1.58639 | 4.683456 |
| ENSG00000243410 | *PSMD6-AS1* | 0 | -0.92498 | 2.803263 |
| ENSG00000218902 | *PTMAP3* | -1.02491 | 0 | 3.914628 |
| ENSG00000159335 | *PTMS* | 0 | -1.14048 | 4.809191 |
| ENSG00000144724 | *PTPRG* | 0 | -1.59116 | 2.312615 |
| ENSG00000187024 | *PTRH1* | 0 | -1.55826 | 2.56294 |
| ENSG00000167994 | *RAB3IL1* | 0 | -0.63422 | 3.07931 |
| ENSG00000223525 | *RABGAP1L-IT1* | 0 | -1.97457 | 2.291048 |
| ENSG00000244588 | *RAD21L1* | 0 | -4.03853 | 4.173702 |
| ENSG00000108557 | *RAI1* | 0 | -1.57325 | 2.169505 |
| ENSG00000173166 | *RAPH1* | -1.05485 | 0 | 3.872016 |
| ENSG00000172819 | *RARG* | 0 | -1.01745 | 3.516281 |
| ENSG00000100276 | *RASL10A* | 0 | -1.55194 | 2.168531 |
| ENSG00000273313 | *RBAKDN* | -0.05789 | 0 | 3.735667 |
| ENSG00000167281 | *RBFOX3* | 0 | -3.62283 | 2.337595 |
| ENSG00000248643 | *RBM14-RBM4* | 0 | -0.90387 | 2.239428 |
| ENSG00000132819 | *RBM38* | 0 | -0.0939 | 2.067299 |
| ENSG00000177483 | *RBM44* | -0.01063 | 0 | 2.21377 |
| ENSG00000236535 | *RC3H1-IT1* | 0 | -2.32591 | 3.551731 |
| ENSG00000104918 | *RETN* | -0.05194 | 0 | 3.331361 |
| ENSG00000229276 | *REV3L-IT1* | 0 | -0.45182 | 2.248866 |
| ENSG00000178882 | *RFLNA* | 0 | -2.8216 | 2.334855 |
| ENSG00000174136 | *RGMB* | -0.43211 | 0 | 2.888839 |
| ENSG00000005486 | *RHBDD2* | 0 | -0.98475 | 2.977251 |
| ENSG00000168421 | *RHOH* | 0 | -0.66956 | 2.259445 |
| ENSG00000187994 | *RINL* | 0 | -0.93393 | 2.035737 |
| ENSG00000222343 | *RN7SKP139* | 0 | 0 | 2.360714 |
| ENSG00000264767 | *RN7SL237P* | 0 | 0 | 6.313704 |
| ENSG00000266710 | *RN7SL48P* | 0 | 0 | 3.743027 |
| ENSG00000239702 | *RN7SL507P* | 0 | -2.10768 | 3.403236 |
| ENSG00000243642 | *RN7SL526P* | -0.22199 | 0 | 2.063858 |
| ENSG00000263905 | *RN7SL555P* | 0 | -2.20895 | 2.334349 |
| ENSG00000265272 | *RN7SL693P* | 0 | 0 | 3.139873 |
| ENSG00000272096 | *RN7SL715P* | -2.17701 | 0 | 2.252818 |
| ENSG00000199523 | *RNA5SP226* | 0 | -0.05655 | 2.541871 |
| ENSG00000201041 | *RNA5SP242* | -1.23221 | 0 | 2.815236 |
| ENSG00000239021 | *RNA5SP246* | -0.44193 | 0 | 3.044668 |
| ENSG00000200278 | *RNA5SP352* | 0 | -0.34323 | 3.058993 |
| ENSG00000252623 | *RNA5SP481* | 0 | -0.37277 | 2.528626 |
| ENSG00000158717 | *RNF166* | 0 | -1.37152 | 2.774016 |
| ENSG00000145428 | *RNF175* | 0 | -2.56866 | 3.042402 |
| ENSG00000063978 | *RNF4* | 0 | -0.21516 | 2.102739 |
| ENSG00000199805 | *RNU1-134P* | 0 | -0.38128 | 2.517266 |
| ENSG00000206908 | *RNU1-136P* | -0.3397 | 0 | 2.237594 |
| ENSG00000199846 | *RNU1-72P* | 0 | -0.71646 | 2.402132 |
| ENSG00000252255 | *RNU2-35P* | -0.92069 | 0 | 2.884039 |
| ENSG00000222414 | *RNU2-59P* | 0 | -1.79883 | 3.018789 |
| ENSG00000222686 | *RNU4-72P* | -0.18214 | 0 | 3.016258 |
| ENSG00000201628 | *RNU4-7P* | -0.86728 | 0 | 3.661092 |
| ENSG00000251988 | *RNU4ATAC18P* | 0 | -0.60258 | 2.35213 |
| ENSG00000206969 | *RNU6-1316P* | -1.58917 | 0 | 2.055049 |
| ENSG00000222932 | *RNU6-172P* | -0.08265 | 0 | 2.914691 |
| ENSG00000252766 | *RNU6-255P* | 0 | 0 | 3.469121 |
| ENSG00000206992 | *RNU6-574P* | -0.1456 | 0 | 2.13196 |
| ENSG00000207428 | *RNU6-95P* | 0 | 0 | 3.144071 |
| ENSG00000154134 | *ROBO3* | 0 | -0.44414 | 2.539687 |
| ENSG00000185483 | *ROR1* | 0 | -0.19645 | 2.511708 |
| ENSG00000224825 | *RORB-AS1* | 0 | -0.56866 | 3.629965 |
| ENSG00000261187 | *RP11-1007O24.2* | 0 | -0.18831 | 2.490006 |
| ENSG00000259240 | *RP11-108K3.1* | -0.41722 | 0 | 4.566459 |
| ENSG00000280035 | *RP11-10J21.2* | 0 | -0.47771 | 2.529964 |
| ENSG00000228801 | *RP11-110G21.1* | 0 | -1.15079 | 2.171726 |
| ENSG00000263916 | *RP11-110H1.4* | 0 | -0.50083 | 2.432816 |
| ENSG00000232611 | *RP11-1114A5.4* | 0 | -0.62886 | 2.365308 |
| ENSG00000258526 | *RP11-111A21.1* | 0 | -0.02521 | 3.677877 |
| ENSG00000237595 | *RP11-112L6.3* | -0.93764 | 0 | 2.658214 |
| ENSG00000250131 | *RP11-130F10.1* | 0 | -0.15117 | 3.059819 |
| ENSG00000279179 | *RP11-131M11.3* | 0 | -0.77205 | 2.146421 |
| ENSG00000279432 | *RP11-135N5.3* | 0 | -0.84172 | 2.240475 |
| ENSG00000274859 | *RP11-136L23.2* | 0 | 0 | 5.735272 |
| ENSG00000263847 | *RP11-143J12.3* | 0 | -2.75665 | 2.575991 |
| ENSG00000226349 | *RP11-145A3.2* | 0 | -1.27816 | 4.20052 |
| ENSG00000233926 | *RP11-154D17.1* | 0 | -1.26278 | 2.894773 |
| ENSG00000278376 | *RP11-158I9.8* | 0 | -0.74832 | 2.823242 |
| ENSG00000259762 | *RP11-158M2.4* | 0 | -1.91206 | 2.37246 |
| ENSG00000248554 | *RP11-159F24.6* | 0 | -0.18811 | 3.475046 |
| ENSG00000284428 | *RP11-15H20.6* | 0 | -0.80551 | 2.130844 |
| ENSG00000260807 | *RP11-161M6.2* | 0 | -1.77942 | 3.436979 |
| ENSG00000273107 | *RP11-165A20.3* | 0 | 0 | 4.790481 |
| ENSG00000255847 | *RP11-167N4.2* | 0 | -0.06859 | 2.744602 |
| ENSG00000270165 | *RP11-167P11.2* | 0 | -0.8484 | 2.103662 |
| ENSG00000272483 | *RP11-169K17.3* | -0.84081 | 0 | 4.172355 |
| ENSG00000257696 | *RP11-175P13.2* | 0 | -2.84504 | 2.826223 |
| ENSG00000203546 | *RP11-176H8.1* | 0 | -1.53023 | 2.781608 |
| ENSG00000131152 | *RP11-178L8.4* | -0.2309 | 0 | 6.408848 |
| ENSG00000279464 | *RP11-17P16.1* | 0 | -0.75676 | 2.596466 |
| ENSG00000275963 | *RP11-180M15.6* | 0 | -1.30968 | 2.303903 |
| ENSG00000258472 | *RP11-192H23.4* | 0 | -1.39341 | 2.420124 |
| ENSG00000258728 | *RP11-195F19.29* | 0 | 0 | 3.428496 |
| ENSG00000275186 | *RP11-196B3.3* | -0.45056 | 0 | 2.100167 |
| ENSG00000254006 | *RP11-1D12.2* | -0.13058 | 0 | 3.504368 |
| ENSG00000227218 | *RP11-203J24.8* | 0 | -0.17838 | 4.563939 |
| ENSG00000258515 | *RP11-203M5.7* | 0 | 0 | 3.73756 |
| ENSG00000228327 | *RP11-206L10.2* | 0 | -0.02525 | 2.372741 |
| ENSG00000280402 | *RP11-20I23.10* | 0 | -0.20716 | 3.997534 |
| ENSG00000279520 | *RP11-20I23.2* | 0 | -1.11059 | 2.7445 |
| ENSG00000259784 | *RP11-20I23.3* | 0 | -2.8594 | 2.579134 |
| ENSG00000271762 | *RP11-213H15.4* | 0 | -1.67936 | 2.745783 |
| ENSG00000228395 | *RP11-216B9.6* | 0 | -0.7265 | 3.441685 |
| ENSG00000280758 | *RP11-216B9.9* | 0 | -0.60418 | 2.061227 |
| ENSG00000283208 | *RP11-226E21.4* | 0 | -2.0273 | 2.685682 |
| ENSG00000274341 | *RP11-227G15.10* | 0 | -0.99161 | 3.980541 |
| ENSG00000279073 | *RP11-229P13.27* | 0 | -3.03578 | 2.196178 |
| ENSG00000111788 | *RP11-22B23.1* | -0.69773 | 0 | 2.254474 |
| ENSG00000233895 | *RP1-122P22.2* | 0 | -1.86311 | 3.731023 |
| ENSG00000263338 | *RP11-235E17.3* | 0 | -1.20633 | 2.328199 |
| ENSG00000225750 | *RP11-242O24.3* | 0 | -0.26941 | 3.441762 |
| ENSG00000261058 | *RP11-252E2.2* | 0 | -0.77187 | 3.93692 |
| ENSG00000261783 | *RP11-252K23.2* | 0 | -2.03496 | 2.353242 |
| ENSG00000266677 | *RP11-258F1.1* | 0 | -1.23231 | 2.306861 |
| ENSG00000249731 | *RP11-259O2.3* | -2.0397 | 0 | 3.637644 |
| ENSG00000234665 | *RP11-262H14.3* | 0 | -2.54763 | 3.00037 |
| ENSG00000279147 | *RP11-271K21.12* | 0 | -0.27679 | 2.894505 |
| ENSG00000277504 | *RP11-278A23.4* | 0 | -2.77528 | 2.491119 |
| ENSG00000249679 | *RP11-279O9.4* | 0 | 0 | 5.027419 |
| ENSG00000251152 | *RP11-281P23.1* | -1.71472 | 0 | 2.214355 |
| ENSG00000279632 | *RP11-286N22.6* | 0 | -0.56121 | 2.363486 |
| ENSG00000278963 | *RP11-294J22.7* | 0 | -0.39646 | 2.155797 |
| ENSG00000266111 | *RP11-296K13.4* | 0 | -0.96611 | 2.008224 |
| ENSG00000230534 | *RP11-297A16.2* | 0 | -0.85407 | 2.612954 |
| ENSG00000280137 | *RP11-297C4.6* | -1.08999 | 0 | 3.336325 |
| ENSG00000270008 | *RP11-298P3.4* | 0 | -1.97861 | 2.87345 |
| ENSG00000259967 | *RP11-2E17.2* | 0 | -1.94856 | 2.084065 |
| ENSG00000261061 | *RP11-303E16.2* | -0.06236 | 0 | 2.295715 |
| ENSG00000260778 | *RP11-304L19.13* | 0 | -1.28203 | 2.147557 |
| ENSG00000261532 | *RP11-304L19.8* | 0 | -0.10056 | 2.2573 |
| ENSG00000259353 | *RP11-30K9.5* | 0 | 0 | 6.09287 |
| ENSG00000267583 | *RP11-322E11.5* | 0 | -1.73349 | 2.103375 |
| ENSG00000271335 | *RP11-324I22.4* | 0 | -0.84066 | 2.250008 |
| ENSG00000255328 | *RP11-326C3.12* | 0 | -0.81218 | 3.434931 |
| ENSG00000255026 | *RP11-326C3.2* | 0 | -1.79553 | 2.78039 |
| ENSG00000274281 | *RP11-326N17.2* | 0 | -0.34802 | 6.620465 |
| ENSG00000249734 | *RP11-332J15.1* | 0 | -1.01392 | 3.351851 |
| ENSG00000272071 | *RP11-332J15.4* | 0 | -0.59771 | 3.073027 |
| ENSG00000262580 | *RP11-334C17.5* | -0.41777 | 0 | 2.492371 |
| ENSG00000279133 | *RP11-342K2.1* | 0 | -0.98721 | 2.937631 |
| ENSG00000235426 | *RP11-342M3.5* | 0 | 0 | 4.697667 |
| ENSG00000259410 | *RP11-34F13.3* | 0 | -0.59752 | 3.150856 |
| ENSG00000268903 | *RP11-34P13.15* | -0.90776 | 0 | 2.973138 |
| ENSG00000259735 | *RP11-356M20.3* | 0 | -3.12594 | 3.093333 |
| ENSG00000279917 | *RP11-360N9.2* | 0 | -0.47243 | 2.370777 |
| ENSG00000284418 | *RP11-370B11.4* | 0 | 0 | 7.260564 |
| ENSG00000267520 | *RP11-373L24.1* | 0 | -0.12259 | 5.395493 |
| ENSG00000272267 | *RP11-375N15.2* | 0 | -0.30105 | 2.025801 |
| ENSG00000272440 | *RP11-379F4.6* | -0.82626 | 0 | 2.031506 |
| ENSG00000214803 | *RP11-37N22.1* | 0 | -2.7351 | 3.327929 |
| ENSG00000228021 | *RP11-383C5.3* | 0 | 0 | 5.835972 |
| ENSG00000266538 | *RP11-385D13.3* | 0 | -1.01573 | 2.113031 |
| ENSG00000260060 | *RP11-388M20.1* | 0 | -0.24345 | 2.818725 |
| ENSG00000247498 | *RP11-392P7.6* | -0.78195 | 0 | 2.107437 |
| ENSG00000266473 | *RP11-401F2.3* | 0 | -0.5156 | 3.549359 |
| ENSG00000259083 | *RP11-407N17.4* | 0 | -0.83918 | 2.989728 |
| ENSG00000275741 | *RP11-40E6.1* | 0 | 0 | 6.101549 |
| ENSG00000233216 | *RP11-414C16.1* | -0.38395 | 0 | 3.566008 |
| ENSG00000255992 | *RP11-417L19.4* | 0 | 0 | 6.380886 |
| ENSG00000283057 | *RP11-417N10.5* | -0.1846 | 0 | 4.266057 |
| ENSG00000279180 | *RP11-417O18.1* | 0 | 0 | 2.972964 |
| ENSG00000273062 | *RP11-428K3.1* | 0 | -0.86202 | 2.060706 |
| ENSG00000259713 | *RP11-429B14.1* | 0 | -0.88178 | 2.855571 |
| ENSG00000259771 | *RP11-429D19.1* | 0 | 0 | 3.703366 |
| ENSG00000259843 | *RP11-429P3.3* | -0.32969 | 0 | 4.240634 |
| ENSG00000279356 | *RP11-429P3.8* | 0 | -0.25257 | 2.418578 |
| ENSG00000230105 | *RP11-431N15.2* | -0.14466 | 0 | 3.829561 |
| ENSG00000231705 | *RP11-432J24.2* | 0 | -0.867 | 3.476895 |
| ENSG00000280037 | *RP11-434P11.1* | 0 | -0.73023 | 2.055772 |
| ENSG00000271623 | *RP11-435I10.5* | 0 | -1.27869 | 3.471083 |
| ENSG00000279344 | *RP11-44F14.7* | 0 | -0.51672 | 3.180518 |
| ENSG00000274272 | *RP11-44M6.7* | 0 | -2.32454 | 2.686537 |
| ENSG00000225850 | *RP11-452K12.4* | 0 | -0.61446 | 3.367021 |
| ENSG00000261416 | *RP11-455F5.5* | -0.1106 | 0 | 3.0043 |
| ENSG00000273521 | *RP11-45A17.4* | -0.50069 | 0 | 2.652521 |
| ENSG00000270302 | *RP11-461L13.4* | 0 | -0.23963 | 2.743456 |
| ENSG00000251226 | *RP11-469N6.1* | -3.05993 | 0 | 2.94041 |
| ENSG00000278171 | *RP11-476I15.5* | -0.60955 | 0 | 5.968672 |
| ENSG00000251535 | *RP11-478C6.1* | 0 | -1.60539 | 2.725484 |
| ENSG00000251379 | *RP11-484O2.1* | 0 | -0.0871 | 5.009536 |
| ENSG00000279691 | *RP11-485M7.2* | 0 | -1.25138 | 2.755743 |
| ENSG00000232347 | *RP11-488L18.8* | 0 | -0.76844 | 3.650183 |
| ENSG00000282418 | *RP11-496N12.9* | 0 | -0.00323 | 4.406665 |
| ENSG00000266957 | *RP11-49K24.4* | -0.44281 | 0 | 2.118624 |
| ENSG00000229930 | *RP11-504P24.5* | -0.41728 | 0 | 2.322289 |
| ENSG00000279665 | *RP11-507J18.5* | 0 | -0.46735 | 2.670319 |
| ENSG00000273507 | *RP11-50D16.4* | 0 | -0.61596 | 2.401667 |
| ENSG00000230555 | *RP11-517P14.2* | 0 | -0.77515 | 2.407696 |
| ENSG00000244346 | *RP11-531F16.3* | 0 | -2.02834 | 4.024825 |
| ENSG00000227741 | *RP11-536C5.7* | 0 | -0.47669 | 3.379772 |
| ENSG00000225302 | *RP11-539I5.1* | -1.00119 | 0 | 3.26866 |
| ENSG00000251246 | *RP11-540D14.8* | 0 | -0.54789 | 4.673885 |
| ENSG00000235652 | *RP11-545I5.3* | 0 | -0.04752 | 2.26954 |
| ENSG00000266980 | *RP11-552F3.4* | -0.1288 | 0 | 2.587574 |
| ENSG00000280274 | *RP11-556H2.1* | 0 | -1.60358 | 3.075555 |
| ENSG00000215244 | *RP11-563J2.2* | 0 | -0.67751 | 2.886887 |
| ENSG00000272716 | *RP11-563N4.1* | 0 | -0.66425 | 2.175073 |
| ENSG00000267048 | *RP11-566K11.7* | 0 | -3.104 | 3.047909 |
| ENSG00000253140 | *RP11-567J20.3* | 0 | 0 | 5.528458 |
| ENSG00000278862 | *RP11-571O6.2* | 0 | 0 | 4.907241 |
| ENSG00000268509 | *RP11-572B2.1* | 0 | -0.77917 | 2.431723 |
| ENSG00000251536 | *RP11-572C21.1* | 0 | -0.58277 | 2.32607 |
| ENSG00000280238 | *RP11-574K11.26* | 0 | 0 | 2.839306 |
| ENSG00000279806 | *RP11-58O9.2* | -0.84058 | 0 | 4.36087 |
| ENSG00000257553 | *RP11-603J24.17* | -1.06543 | 0 | 2.416531 |
| ENSG00000256325 | *RP11-611O2.1* | 0 | -0.15198 | 2.350026 |
| ENSG00000213272 | *RP11-613M5.1* | 0 | -0.06766 | 2.37965 |
| ENSG00000235051 | *RP11-637O19.2* | 0 | -1.06034 | 3.206013 |
| ENSG00000278867 | *RP11-640N20.4* | 0 | -0.45942 | 2.434055 |
| ENSG00000264164 | *RP11-640N20.6* | 0 | -0.14789 | 3.29255 |
| ENSG00000254141 | *RP11-642D21.1* | -0.55609 | 0 | 3.93963 |
| ENSG00000279035 | *RP11-649A18.3* | 0 | -2.36929 | 2.029067 |
| ENSG00000265800 | *RP11-649A18.5* | 0 | -0.97427 | 2.643012 |
| ENSG00000262810 | *RP11-667K14.5* | 0 | -1.12617 | 4.586117 |
| ENSG00000259570 | *RP11-671M22.4* | -1.17558 | 0 | 2.348502 |
| ENSG00000246308 | *RP11-685M7.3* | 0 | -0.7797 | 2.730465 |
| ENSG00000253115 | *RP11-6I2.4* | 0 | -1.87459 | 3.025374 |
| ENSG00000254631 | *RP11-702H23.4* | 0 | 0 | 3.005608 |
| ENSG00000249614 | *RP11-703G6.1* | 0 | -0.42709 | 5.814611 |
| ENSG00000250125 | *RP11-707A18.1* | -2.55321 | 0 | 5.849783 |
| ENSG00000279962 | *RP11-720L3.1* | 0 | -3.4791 | 4.462882 |
| ENSG00000255234 | *RP11-727A23.10* | 0 | -1.82344 | 2.302596 |
| ENSG00000240211 | *RP11-758P17.3* | 0 | -1.14519 | 2.4721 |
| ENSG00000279801 | *RP11-75C10.6* | 0 | -0.12761 | 2.020642 |
| ENSG00000231873 | *RP11-761N21.1* | 0 | 0 | 3.47367 |
| ENSG00000259562 | *RP11-762H8.2* | 0 | -0.28825 | 2.012417 |
| ENSG00000264635 | *RP11-769O8.3* | 0 | -1.1699 | 2.447247 |
| ENSG00000273450 | *RP11-76P2.4* | 0 | -1.02573 | 2.420917 |
| ENSG00000229065 | *RP11-80I15.4* | 0 | -0.26409 | 4.01578 |
| ENSG00000259453 | *RP11-815J21.1* | 0 | -0.48831 | 2.35389 |
| ENSG00000240652 | *RP11-832N8.1* | -0.0221 | 0 | 2.503572 |
| ENSG00000279573 | *RP11-855A2.1* | 0 | -2.462 | 3.000326 |
| ENSG00000279434 | *RP11-85K15.3* | 0 | -1.31034 | 2.204202 |
| ENSG00000271600 | *RP11-864G5.8* | -0.41445 | 0 | 5.422661 |
| ENSG00000279827 | *RP11-886P16.10* | 0 | -1.46282 | 2.298738 |
| ENSG00000240015 | *RP11-90P5.5* | 0 | -0.68781 | 2.421221 |
| ENSG00000261218 | *RP11-960L18.1* | 0 | -1.96341 | 2.370914 |
| ENSG00000273413 | *RP11-96C23.15* | 0 | -2.39733 | 3.95539 |
| ENSG00000274902 | *RP1-197B17.4* | 0 | -0.87472 | 3.283364 |
| ENSG00000257475 | *RP11-983P16.2* | 0 | -1.21207 | 2.737438 |
| ENSG00000256299 | *RP11-989F5.3* | 0 | -0.13096 | 3.55961 |
| ENSG00000197815 | *RP1-253P7.4* | 0 | -1.40356 | 5.777448 |
| ENSG00000255575 | *RP1-267D11.1* | -0.14473 | 0 | 3.07418 |
| ENSG00000268575 | *RP1-283E3.8* | 0 | -0.77787 | 2.355051 |
| ENSG00000213279 | *RP1-29C18.9* | 0 | -1.26534 | 3.105338 |
| ENSG00000232194 | *RP1-313L4.3* | 0 | -0.72524 | 3.009481 |
| ENSG00000224374 | *RP1-32B1.4* | 0 | -0.68825 | 2.772691 |
| ENSG00000255916 | *RP13-895J2.7* | 0 | 0 | 4.59025 |
| ENSG00000272277 | *RP1-40E16.12* | 0 | -1.45246 | 2.308822 |
| ENSG00000274758 | *RP1-59D14.10* | 0 | 0 | 3.632417 |
| ENSG00000263345 | *RP1-59D14.5* | 0 | -0.60958 | 3.80427 |
| ENSG00000261251 | *RP3-388M5.9* | 0 | -0.0628 | 2.086632 |
| ENSG00000273253 | *RP3-402G11.26* | 0 | -1.09727 | 3.162344 |
| ENSG00000280184 | *RP3-466P17.3* | 0 | -0.59256 | 2.286614 |
| ENSG00000236266 | *RP3-467L1.4* | 0 | -0.90236 | 2.356029 |
| ENSG00000231485 | *RP4-535B20.1* | 0 | -2.2195 | 3.385702 |
| ENSG00000260498 | *RP4-536B24.4* | -0.78684 | 0 | 3.065286 |
| ENSG00000244198 | *RP4-545C24.1* | 0 | -2.52142 | 2.768353 |
| ENSG00000273204 | *RP4-549L20.3* | 0 | -0.41104 | 2.54643 |
| ENSG00000260940 | *RP4-575N6.5* | -0.10225 | 0 | 2.833826 |
| ENSG00000274825 | *RP4-616B8.5* | 0 | -1.92672 | 2.048033 |
| ENSG00000232528 | *RP4-673D20.3* | 0 | -1.40077 | 4.208926 |
| ENSG00000241073 | *RP4-714D9.2* | 0 | 0 | 4.028643 |
| ENSG00000226824 | *RP4-756H11.3* | 0 | -1.00332 | 2.005187 |
| ENSG00000280378 | *RP4-758J24.6* | 0 | -0.76314 | 3.296597 |
| ENSG00000225030 | *RP4-784A16.3* | 0 | 0 | 2.418021 |
| ENSG00000218018 | *RP4-800J21.3* | -0.79574 | 0 | 2.623077 |
| ENSG00000279738 | *RP5-1014D13.2* | 0 | -0.98094 | 2.0615 |
| ENSG00000270605 | *RP5-1092A3.4* | 0 | -0.0777 | 2.477252 |
| ENSG00000277425 | *RP5-1116H23.3* | 0 | -1.65237 | 2.147594 |
| ENSG00000269934 | *RP5-1139B12.3* | 0 | -2.26298 | 2.366577 |
| ENSG00000260121 | *RP5-1142A6.9* | 0 | -0.22581 | 2.163261 |
| ENSG00000280387 | *RP5-1153D9.5* | 0 | -1.72537 | 2.465407 |
| ENSG00000235329 | *RP5-1168A5.1* | 0 | 0 | 4.003196 |
| ENSG00000267457 | *RP5-837J1.4* | 0 | -2.5161 | 3.761123 |
| ENSG00000271806 | *RP5-892K4.1* | -0.87306 | 0 | 2.790701 |
| ENSG00000260179 | *RP5-902P8.12* | 0 | -2.47514 | 3.225037 |
| ENSG00000279306 | *RP5-915N17.11* | 0 | -1.18803 | 2.508984 |
| ENSG00000257529 | *RPL36A-HNRNPH2* | 0 | -0.24681 | 2.2812 |
| ENSG00000224315 | *RPL7P7* | 0 | -1.88002 | 2.146874 |
| ENSG00000179673 | *RPRML* | 0 | -3.28431 | 3.503872 |
| ENSG00000244295 | *RPS20P21* | -0.30942 | 0 | 2.39902 |
| ENSG00000236888 | *RPS20P5* | -0.70087 | 0 | 2.149462 |
| ENSG00000218351 | *RPS3AP23* | 0 | -1.36668 | 2.189418 |
| ENSG00000259105 | *RPS3AP4* | 0 | -0.5508 | 4.460913 |
| ENSG00000254985 | *RSF1-IT2* | 0 | -1.27399 | 2.575603 |
| ENSG00000239608 | *RUVBL1-AS1* | 0 | -0.12074 | 3.744065 |
| ENSG00000188322 | *SBK1* | 0 | -1.37832 | 2.160969 |
| ENSG00000064932 | *SBNO2* | 0 | -1.74918 | 2.180534 |
| ENSG00000126461 | *SCAF1* | 0 | -1.342 | 2.264607 |
| ENSG00000244486 | *SCARF2* | 0 | -2.21215 | 3.152138 |
| ENSG00000270066 | *SCARNA2* | -0.31867 | 0 | 9.042156 |
| ENSG00000214279 | *SCART1* | 0 | -1.53187 | 2.835981 |
| ENSG00000268751 | *SCGB1B2P* | 0 | -1.0888 | 3.484506 |
| ENSG00000169439 | *SDC2* | 0 | -0.03292 | 2.652076 |
| ENSG00000001617 | *SEMA3F* | 0 | -1.74418 | 2.678185 |
| ENSG00000137872 | *SEMA6D* | -0.11391 | 0 | 3.653876 |
| ENSG00000163935 | *SFMBT1* | -0.71933 | 0 | 3.006815 |
| ENSG00000118473 | *SGIP1* | 0 | -1.48519 | 2.121118 |
| ENSG00000166224 | *SGPL1* | 0 | -0.79507 | 2.139498 |
| ENSG00000181523 | *SGSH* | 0 | -0.39516 | 2.817611 |
| ENSG00000027869 | *SH2D2A* | 0 | -2.09179 | 2.364824 |
| ENSG00000125731 | *SH2D3A* | 0 | -1.6173 | 2.946835 |
| ENSG00000162105 | *SHANK2* | 0 | -0.89784 | 3.735366 |
| ENSG00000197046 | *SIGLEC15* | 0 | -1.70707 | 3.277787 |
| ENSG00000157933 | *SKI* | 0 | -1.37309 | 3.22071 |
| ENSG00000188779 | *SKOR1* | 0 | -0.50001 | 2.044235 |
| ENSG00000221955 | *SLC12A8* | 0 | -1.08621 | 2.365006 |
| ENSG00000079215 | *SLC1A3* | 0 | -0.6509 | 3.042784 |
| ENSG00000137266 | *SLC22A23* | 0 | -0.80011 | 3.079227 |
| ENSG00000181045 | *SLC26A11* | 0 | -1.1476 | 3.236241 |
| ENSG00000227533 | *SLC2A1-AS1* | 0 | -0.12041 | 2.058247 |
| ENSG00000136868 | *SLC31A1* | 0 | -0.26326 | 2.581386 |
| ENSG00000127526 | *SLC35E1* | 0 | -0.67113 | 2.36738 |
| ENSG00000114923 | *SLC4A3* | 0 | -3.38223 | 4.438281 |
| ENSG00000258186 | *SLC7A5P2* | 0 | 0 | 4.284552 |
| ENSG00000213599 | *SLX1A-SULT1A3* | 0 | -1.72672 | 3.843418 |
| ENSG00000245112 | *SMARCA5-AS1* | 0 | -0.34328 | 3.598917 |
| ENSG00000237296 | *SMG1P1* | 0 | 0 | 2.20573 |
| ENSG00000180747 | *SMG1P3* | 0 | 0 | 2.34247 |
| ENSG00000235169 | *SMIM1* | 0 | -1.62384 | 2.392548 |
| ENSG00000268182 | *SMIM17* | 0 | -0.80588 | 2.095425 |
| ENSG00000162804 | *SNED1* | 0 | -0.24478 | 2.433249 |
| ENSG00000225746 | *SNHG23* | -0.18601 | 0 | 4.846474 |
| ENSG00000271907 | *SNORA35* | 0 | 0 | 2.479092 |
| ENSG00000199787 | *SNORA42* | -0.56319 | 0 | 2.997798 |
| ENSG00000207001 | *SNORD116-2* | 0 | 0 | 2.404682 |
| ENSG00000278715 | *SNORD116-20* | 0 | -0.77085 | 3.445807 |
| ENSG00000252326 | *SNORD116-25* | 0 | 0 | 3.638348 |
| ENSG00000275529 | *SNORD116-4* | 0 | 0 | 2.464136 |
| ENSG00000231480 | *SNRPGP13* | -0.49828 | 0 | 3.065736 |
| ENSG00000162627 | *SNX7* | -0.1625 | 0 | 3.540638 |
| ENSG00000184985 | *SORCS2* | 0 | -2.20447 | 4.009866 |
| ENSG00000177732 | *SOX12* | 0 | -1.32345 | 2.530181 |
| ENSG00000161888 | *SPC24* | -0.03256 | 0 | 2.132363 |
| ENSG00000152253 | *SPC25* | 0 | -2.34568 | 3.431204 |
| ENSG00000072195 | *SPEG* | 0 | -0.16373 | 2.898102 |
| ENSG00000107742 | *SPOCK2* | -0.29021 | 0 | 2.232408 |
| ENSG00000179954 | *SSC5D* | 0 | -1.00655 | 2.752815 |
| ENSG00000197558 | *SSPO* | 0 | -1.95478 | 2.85569 |
| ENSG00000235374 | *SSR4P1* | -0.21164 | 0 | 2.149958 |
| ENSG00000260233 | *SSSCA1-AS1* | -0.01366 | 0 | 2.350855 |
| ENSG00000125046 | *SSUH2* | 0 | -0.52834 | 3.793649 |
| ENSG00000180953 | *ST20* | -0.06286 | 0 | 2.413475 |
| ENSG00000070526 | *ST6GALNAC1* | -0.24286 | 0 | 2.793565 |
| ENSG00000131748 | *STARD3* | 0 | -1.13784 | 2.359441 |
| ENSG00000130052 | *STARD8* | 0 | -0.60528 | 3.897597 |
| ENSG00000173597 | *SULT1B1* | 0 | -0.52942 | 2.466855 |
| ENSG00000205078 | *SYCE1L* | 0 | -1.79696 | 2.570095 |
| ENSG00000196074 | *SYCP2* | 0 | -0.50242 | 3.566609 |
| ENSG00000125755 | *SYMPK* | 0 | -0.95737 | 2.043734 |
| ENSG00000102003 | *SYP* | -0.05542 | 0 | 2.67863 |
| ENSG00000068354 | *TBC1D25* | 0 | -0.64262 | 2.053371 |
| ENSG00000146350 | *TBC1D32* | 0 | -0.17033 | 2.05937 |
| ENSG00000184058 | *TBX1* | 0 | -2.1939 | 2.633389 |
| ENSG00000240280 | *TCAM1P* | 0 | -1.62378 | 4.056696 |
| ENSG00000188396 | *TCTEX1D4* | 0 | -2.51799 | 2.3345 |
| ENSG00000105329 | *TGFB1* | 0 | -0.91887 | 2.263817 |
| ENSG00000260001 | *TGFBR3L* | 0 | -2.0514 | 3.221724 |
| ENSG00000100234 | *TIMP3* | 0 | -1.46495 | 2.445682 |
| ENSG00000160606 | *TLCD1* | -1.2971 | 0 | 3.641171 |
| ENSG00000196781 | *TLE1* | 0 | -1.13845 | 2.83948 |
| ENSG00000065717 | *TLE2* | -0.24751 | 0 | 2.826892 |
| ENSG00000140332 | *TLE3* | 0 | -0.51933 | 3.192777 |
| ENSG00000167895 | *TMC8* | 0 | -1.71511 | 2.613273 |
| ENSG00000183160 | *TMEM119* | 0 | -0.80747 | 3.337754 |
| ENSG00000006118 | *TMEM132A* | 0 | -0.45613 | 4.412961 |
| ENSG00000181264 | *TMEM136* | 0 | -0.54127 | 2.149936 |
| ENSG00000130748 | *TMEM160* | 0 | -1.92464 | 2.068831 |
| ENSG00000226287 | *TMEM191A* | 0 | -0.91952 | 2.739713 |
| ENSG00000206140 | *TMEM191C* | 0 | -0.07715 | 2.107187 |
| ENSG00000188760 | *TMEM198* | 0 | -0.34662 | 2.057042 |
| ENSG00000185863 | *TMEM210* | -1.83986 | 0 | 3.095997 |
| ENSG00000214128 | *TMEM213* | 0 | -2.42525 | 2.405428 |
| ENSG00000196932 | *TMEM26* | 0 | -0.2928 | 2.657015 |
| ENSG00000178821 | *TMEM52* | 0 | -2.17788 | 6.070508 |
| ENSG00000257167 | *TMPO-AS1* | 0 | -0.13879 | 2.088867 |
| ENSG00000254462 | *TMX2-CTNND1* | 0 | -0.83065 | 2.312546 |
| ENSG00000159958 | *TNFRSF13C* | 0 | -2.07912 | 2.178673 |
| ENSG00000049249 | *TNFRSF9* | -0.03317 | 0 | 2.294099 |
| ENSG00000125657 | *TNFSF9* | 0 | -2.2649 | 3.815576 |
| ENSG00000225155 | *TOMM22P5* | -0.76484 | 0 | 2.546484 |
| ENSG00000211802 | *TRAV22* | 0 | -0.8099 | 4.180629 |
| ENSG00000211809 | *TRAV27* | 0 | -3.75786 | 2.092729 |
| ENSG00000259092 | *TRAV30* | -0.55384 | 0 | 4.167867 |
| ENSG00000249978 | *TRGV7* | -0.01972 | 0 | 5.49289 |
| ENSG00000172425 | *TTC36* | 0 | -0.09694 | 2.463861 |
| ENSG00000198680 | *TUSC1* | -0.20705 | 0 | 2.968454 |
| ENSG00000176912 | *TYMSOS* | -0.00908 | 0 | 2.012557 |
| ENSG00000234200 | *U82671.8* | 0 | 0 | 7.824726 |
| ENSG00000144744 | *UBA3* | 0 | -0.29316 | 2.284591 |
| ENSG00000248049 | *UBA6-AS1* | 0 | -0.27576 | 2.328705 |
| ENSG00000228889 | *UBAC2-AS1* | 0 | -0.32732 | 2.208709 |
| ENSG00000263563 | *UBBP4* | 0 | -1.20334 | 3.540216 |
| ENSG00000274051 | *uc_338* | 0 | -1.63069 | 2.871528 |
| ENSG00000154277 | *UCHL1* | 0 | -2.36925 | 2.361266 |
| ENSG00000280213 | *UCKL1-AS1* | 0 | -0.82946 | 2.276942 |
| ENSG00000237512 | *UNC5B-AS1* | -0.47047 | 0 | 6.011764 |
| ENSG00000100024 | *UPB1* | 0 | -0.32758 | 2.236986 |
| ENSG00000243566 | *UPK3B* | 0 | -3.43269 | 2.648062 |
| ENSG00000130307 | *USHBP1* | 0 | -5.0203 | 4.478697 |
| ENSG00000106346 | *USP42* | 0 | -0.0689 | 2.084438 |
| ENSG00000235818 | *VN1R17P* | 0 | -1.14884 | 4.677488 |
| ENSG00000176428 | *VPS37D* | 0 | -1.68286 | 3.5955 |
| ENSG00000165633 | *VSTM4* | 0 | -1.22296 | 2.002418 |
| ENSG00000165171 | *WBSCR27* | -0.18882 | 0 | 2.072351 |
| ENSG00000123154 | *WDR83* | 0 | -0.29276 | 2.035058 |
| ENSG00000187260 | *WDR86* | 0 | -0.42606 | 2.931322 |
| ENSG00000127578 | *WFIKKN1* | 0 | -1.7553 | 2.589019 |
| ENSG00000135925 | *WNT10A* | 0 | -0.85272 | 4.283405 |
| ENSG00000232265 | *XXyac-YX155B6.5* | 0 | 0 | 5.68164 |
| ENSG00000199366 | *Y_RNA* | 0 | -0.32325 | 2.992739 |
| ENSG00000201990 | *Y_RNA* | 0 | 0 | 2.847142 |
| ENSG00000202533 | *Y_RNA* | -0.4311 | 0 | 2.309945 |
| ENSG00000200090 | *Y_RNA* | 0 | 0 | 2.157543 |
| ENSG00000202385 | *Y_RNA* | -1.83628 | 0 | 2.13598 |
| ENSG00000269482 | *Z69720.2* | 0 | -0.31856 | 2.719995 |
| ENSG00000178951 | *ZBTB7A* | 0 | -1.08114 | 2.233396 |
| ENSG00000184828 | *ZBTB7C* | 0 | -1.59391 | 2.454231 |
| ENSG00000163874 | *ZC3H12A* | 0 | -0.33801 | 2.473085 |
| ENSG00000169946 | *ZFPM2* | 0 | -1.08195 | 3.057684 |
| ENSG00000234230 | *ZFX-AS1* | 0 | -2.92224 | 2.966031 |
| ENSG00000004838 | *ZMYND10* | 0 | -0.57367 | 2.773371 |
| ENSG00000164631 | *ZNF12* | 0 | -0.32678 | 2.000692 |
| ENSG00000132010 | *ZNF20* | 0 | -0.75106 | 2.280858 |
| ENSG00000170260 | *ZNF212* | 0 | -0.59428 | 2.134243 |
| ENSG00000188283 | *ZNF383* | 0 | -0.71116 | 2.431929 |
| ENSG00000133250 | *ZNF414* | 0 | -1.27137 | 2.432645 |
| ENSG00000181444 | *ZNF467* | 0 | -2.12467 | 2.937149 |
| ENSG00000218891 | *ZNF579* | 0 | -1.00671 | 2.14949 |
| ENSG00000269343 | *ZNF587B* | 0 | -0.25217 | 2.066121 |
| ENSG00000197483 | *ZNF628* | 0 | -1.37382 | 2.14229 |
| ENSG00000160229 | *ZNF66* | 0 | -0.80474 | 2.42565 |
| ENSG00000164684 | *ZNF704* | 0 | -0.98695 | 2.118693 |
| ENSG00000213967 | *ZNF726* | 0 | -0.44243 | 2.197478 |
| ENSG00000214652 | *ZNF727* | 0 | -4.75297 | 2.84757 |
| ENSG00000214189 | *ZNF788* | 0 | -0.31127 | 2.130818 |
| ENSG00000224689 | *ZNF812P* | 0 | -0.03584 | 7.950938 |
| ENSG00000204514 | *ZNF814* | 0 | -0.11087 | 2.317486 |
| ENSG00000261221 | *ZNF865* | 0 | -1.49345 | 2.107352 |
| ENSG00000186187 | *ZNRF1* | 0 | -1.33067 | 2.389036 |
| ENSG00000152467 | *ZSCAN1* | 0 | 0 | 5.901601 |
| ENSG00000159840 | *ZYX* | 0 | -1.96596 | 2.254779 |

**Supplementary Table 3B: List of genes downregulated in mature T-ALL**

| **Ensembl_ID** | **Gene Name** | **Log_2_fold change in T-ALL subtypes** | | |
| --- | --- | --- | --- | --- |
|  |  | **Immature** | **Cortical** | **Mature** |
| ENSG00000204837 | *FGF7P3* | 0 | 1.817338 | -19.5704 |
| ENSG00000267648 | *RP11-686D22.3* | 0.191998 | 0 | -14.1956 |
| ENSG00000242616 | *GNG10* | 0 | 0.205863 | -13.4148 |
| ENSG00000277654 | *RP11-255M2.3* | 0 | 0.411488 | -13.222 |
| ENSG00000272822 | *RP11-302B13.5* | 0 | 0.628841 | -11.0991 |
| ENSG00000229807 | *XIST* | 1.237498 | 0 | -10.3441 |
| ENSG00000251002 | *AE000661.37* | 1.221508 | 0 | -10.006 |
| ENSG00000269711 | *CTD-3214H19.16* | 1.625316 | 0 | -7.85242 |
| ENSG00000153721 | *CNKSR3* | 0.530524 | 0 | -7.83277 |
| ENSG00000127325 | *BEST3* | 0 | 0.188563 | -7.40429 |
| ENSG00000274582 | *SNORA16A* | 1.812455 | 0 | -6.91346 |
| ENSG00000184005 | *ST6GALNAC3* | 0.914295 | 0 | -6.33278 |
| ENSG00000211695 | *TRGV9* | 1.445114 | 0 | -6.21541 |
| ENSG00000196664 | *TLR7* | 0 | 0.769935 | -5.91586 |
| ENSG00000229140 | *CCDC26* | 0 | 0.860764 | -5.79715 |
| ENSG00000105205 | *CLC* | 0.197814 | 0 | -5.41334 |
| ENSG00000105967 | *TFEC* | 0.264283 | 0 | -5.2217 |
| ENSG00000113088 | *GZMK* | 0.023079 | 0 | -5.18205 |
| ENSG00000139117 | *CPNE8* | 0 | 0.447833 | -5.13989 |
| ENSG00000163751 | *CPA3* | 0.327534 | 0 | -5.08587 |
| ENSG00000038945 | *MSR1* | 0.025214 | 0 | -5.0611 |
| ENSG00000138792 | *ENPEP* | 0 | 1.375286 | -5.03475 |
| ENSG00000267453 | *LINC01835* | 0.577113 | 0 | -5.0051 |
| ENSG00000154237 | *LRRK1* | 2.316872 | 0 | -4.9096 |
| ENSG00000152192 | *POU4F1* | 0.673623 | 0 | -4.84001 |
| ENSG00000170180 | *GYPA* | 0 | 0.000276 | -4.81355 |
| ENSG00000185736 | *ADARB2* | 1.224413 | 0 | -4.81336 |
| ENSG00000101938 | *CHRDL1* | 3.322689 | 0 | -4.78354 |
| ENSG00000156475 | *PPP2R2B* | 1.452561 | 0 | -4.78279 |
| ENSG00000064989 | *CALCRL* | 0 | 0.172663 | -4.78219 |
| ENSG00000086548 | *CEACAM6* | 0.01202 | 0 | -4.77891 |
| ENSG00000204789 | *ZNF204P* | 0.177751 | 0 | -4.75589 |
| ENSG00000130584 | *ZBTB46* | 1.881196 | 0 | -4.73822 |
| ENSG00000170627 | *GTSF1* | 0 | 1.660665 | -4.71772 |
| ENSG00000233204 | *MAPRE1P3* | 0 | 1.690879 | -4.71291 |
| ENSG00000169385 | *RNASE2* | 0 | 0.852067 | -4.66569 |
| ENSG00000136546 | *SCN7A* | 0 | 0.42054 | -4.65453 |
| ENSG00000165259 | *HDX* | 0 | 0.281927 | -4.64282 |
| ENSG00000081923 | *ATP8B1* | 0 | 0.179383 | -4.63125 |
| ENSG00000081985 | *IL12RB2* | 2.398114 | 0 | -4.49714 |
| ENSG00000168497 | *SDPR* | 2.088612 | 0 | -4.47243 |
| ENSG00000206557 | *TRIM71* | 1.542689 | 0 | -4.44481 |
| ENSG00000211747 | *TRBV20-1* | 1.034726 | 0 | -4.4296 |
| ENSG00000019991 | *HGF* | 1.195198 | 0 | -4.39829 |
| ENSG00000102445 | *RUBCNL* | 0.702177 | 0 | -4.33055 |
| ENSG00000256500 | *RP11-73M18.2* | 0 | 0.06795 | -4.30681 |
| ENSG00000116675 | *DNAJC6* | 0.995563 | 0 | -4.30494 |
| ENSG00000101333 | *PLCB4* | 0.341932 | 0 | -4.30348 |
| ENSG00000219891 | *ZSCAN12P1* | 0.062367 | 0 | -4.27929 |
| ENSG00000149289 | *ZC3H12C* | 0 | 0.208575 | -4.26056 |
| ENSG00000109686 | *SH3D19* | 0 | 2.591073 | -4.23318 |
| ENSG00000152217 | *SETBP1* | 0.727101 | 0 | -4.21271 |
| ENSG00000061676 | *NCKAP1* | 0 | 2.816908 | -4.19619 |
| ENSG00000125538 | *IL1B* | 1.068421 | 0 | -4.14464 |
| ENSG00000091128 | *LAMB4* | 0.364646 | 0 | -4.14146 |
| ENSG00000115590 | *IL1R2* | 1.74419 | 0 | -4.10289 |
| ENSG00000173530 | *TNFRSF10D* | 2.00625 | 0 | -4.09707 |
| ENSG00000164330 | *EBF1* | 0 | 0.254771 | -4.096 |
| ENSG00000163508 | *EOMES* | 0 | 0.033892 | -4.06778 |
| ENSG00000204179 | *PTPN20* | 0 | 0.361111 | -4.03896 |
| ENSG00000103569 | *AQP9* | 0.341761 | 0 | -4.02887 |
| ENSG00000154451 | *GBP5* | 1.537247 | 0 | -4.0269 |
| ENSG00000169397 | *RNASE3* | 0 | 0.329001 | -4.02373 |
| ENSG00000137959 | *IFI44L* | 1.084632 | 0 | -4.0113 |
| ENSG00000249138 | *SLED1* | 0 | 0.108505 | -3.98882 |
| ENSG00000241956 | *CTC-340A15.2* | 0 | 0.044154 | -3.91709 |
| ENSG00000250337 | *LINC01021* | 5.128458 | 0 | -3.90851 |
| ENSG00000163993 | *S100P* | 0.446433 | 0 | -3.8786 |
| ENSG00000164047 | *CAMP* | 0.155126 | 0 | -3.85562 |
| ENSG00000118785 | *SPP1* | 1.602847 | 0 | -3.80993 |
| ENSG00000204010 | *IFIT1B* | 1.372617 | 0 | -3.77909 |
| ENSG00000166450 | *PRTG* | 0 | 3.199745 | -3.76964 |
| ENSG00000153208 | *MERTK* | 1.528102 | 0 | -3.76659 |
| ENSG00000204161 | *C10orf128* | 2.784793 | 0 | -3.75211 |
| ENSG00000167779 | *IGFBP6* | 0.743354 | 0 | -3.75161 |
| ENSG00000101916 | *TLR8* | 0.464744 | 0 | -3.74142 |
| ENSG00000235033 | *RP11-61I13.3* | 0 | 2.988045 | -3.73337 |
| ENSG00000147459 | *DOCK5* | 0.487638 | 0 | -3.71631 |
| ENSG00000163221 | *S100A12* | 0.691683 | 0 | -3.70216 |
| ENSG00000124140 | *SLC12A5* | 1.019242 | 0 | -3.69581 |
| ENSG00000283235 | *RP11-844P9.6* | 0 | 1.911226 | -3.67422 |
| ENSG00000257674 | *RP11-76E16.2* | 0 | 1.061266 | -3.6626 |
| ENSG00000233327 | *USP32P2* | 0 | 0.244496 | -3.65925 |
| ENSG00000124491 | *F13A1* | 0.670314 | 0 | -3.64664 |
| ENSG00000262664 | *OVCA2* | 0.087646 | 0 | -3.63834 |
| ENSG00000133742 | *CA1* | 0.615425 | 0 | -3.6376 |
| ENSG00000179344 | *HLA-DQB1* | 1.072818 | 0 | -3.59466 |
| ENSG00000137801 | *THBS1* | 0.12846 | 0 | -3.54357 |
| ENSG00000152760 | *TCTEX1D1* | 4.10596 | 0 | -3.52226 |
| ENSG00000267156 | *TPMTP1* | 0.113767 | 0 | -3.5133 |
| ENSG00000255987 | *TOMM20P2* | 0.160631 | 0 | -3.51104 |
| ENSG00000198816 | *ZNF358* | 0.809234 | 0 | -3.48375 |
| ENSG00000211668 | *IGLV2-11* | 1.244495 | 0 | -3.4672 |
| ENSG00000134901 | *KDELC1* | 1.379194 | 0 | -3.4539 |
| ENSG00000172322 | *CLEC12A* | 1.890011 | 0 | -3.45079 |
| ENSG00000079263 | *SP140* | 0.918406 | 0 | -3.44976 |
| ENSG00000162551 | *ALPL* | 1.024659 | 0 | -3.44901 |
| ENSG00000170458 | *CD14* | 0.338865 | 0 | -3.43743 |
| ENSG00000171049 | *FPR2* | 0.931095 | 0 | -3.41852 |
| ENSG00000231749 | *ABCA9-AS1* | 0 | 0.655486 | -3.41676 |
| ENSG00000135046 | *ANXA1* | 0 | 0.928175 | -3.39869 |
| ENSG00000165029 | *ABCA1* | 1.804211 | 0 | -3.39777 |
| ENSG00000131724 | *IL13RA1* | 0.899965 | 0 | -3.34698 |
| ENSG00000241782 | *RP11-91P24.1* | 0 | 0.440189 | -3.31969 |
| ENSG00000102837 | *OLFM4* | 0 | 0.117276 | -3.30904 |
| ENSG00000239839 | *DEFA3* | 0.841267 | 0 | -3.30429 |
| ENSG00000267645 | *POLR2J2* | 0.06868 | 0 | -3.28755 |
| ENSG00000163563 | *MNDA* | 0.397712 | 0 | -3.28495 |
| ENSG00000267074 | *RP11-1094M14.5* | 0.730887 | 0 | -3.28389 |
| ENSG00000105974 | *CAV1* | 0.775723 | 0 | -3.28283 |
| ENSG00000186205 | *MARC1* | 0.28416 | 0 | -3.258 |
| ENSG00000143546 | *S100A8* | 0.602801 | 0 | -3.25418 |
| ENSG00000231351 | *AC111200.7* | 0.386296 | 0 | -3.24008 |
| ENSG00000149516 | *MS4A3* | 0.164004 | 0 | -3.22911 |
| ENSG00000259594 | *CTD-2034I4.2* | 0.990766 | 0 | -3.22318 |
| ENSG00000262877 | *RP11-1055B8.4* | 0 | 0.833803 | -3.21139 |
| ENSG00000232846 | *SLC25A6P3* | 0.403189 | 0 | -3.20377 |
| ENSG00000129226 | *CD68* | 0.187718 | 0 | -3.19405 |
| ENSG00000230438 | *SERPINB9P1* | 2.216582 | 0 | -3.17708 |
| ENSG00000119865 | *CNRIP1* | 1.860339 | 0 | -3.17594 |
| ENSG00000101162 | *TUBB1* | 1.456592 | 0 | -3.17345 |
| ENSG00000090104 | *RGS1* | 0.590842 | 0 | -3.16745 |
| ENSG00000211790 | *TRAV8-4* | 0 | 2.026743 | -3.1538 |
| ENSG00000083444 | *PLOD1* | 1.349548 | 0 | -3.14778 |
| ENSG00000259196 | *HMBOX1-IT1* | 0.085813 | 0 | -3.14203 |
| ENSG00000196656 | *AC004057.1* | 0 | 0.856293 | -3.11558 |
| ENSG00000180061 | *TMEM150B* | 0.71047 | 0 | -3.10929 |
| ENSG00000248773 | *RP11-231L11.3* | 0 | 0.055544 | -3.10835 |
| ENSG00000214145 | *LINC00887* | 0.33273 | 0 | -3.08844 |
| ENSG00000205592 | *MUC19* | 3.854791 | 0 | -3.0445 |
| ENSG00000257335 | *MGAM* | 0.956313 | 0 | -3.04093 |
| ENSG00000038427 | *VCAN* | 0 | 0.074089 | -3.02768 |
| ENSG00000177311 | *ZBTB38* | 0.694008 | 0 | -3.02324 |
| ENSG00000278196 | *IGLV2-8* | 1.380132 | 0 | -3.01529 |
| ENSG00000239951 | *IGKV3-20* | 1.320488 | 0 | -2.98885 |
| ENSG00000125968 | *ID1* | 1.378245 | 0 | -2.97712 |
| ENSG00000178222 | *RNF212* | 0.527796 | 0 | -2.97622 |
| ENSG00000273320 | *RP11-22N19.2* | 0.700573 | 0 | -2.96742 |
| ENSG00000110077 | *MS4A6A* | 0.582444 | 0 | -2.96343 |
| ENSG00000137462 | *TLR2* | 0.233523 | 0 | -2.94382 |
| ENSG00000067177 | *PHKA1* | 0 | 0.947135 | -2.9428 |
| ENSG00000237651 | *C2orf74* | 0.941027 | 0 | -2.9396 |
| ENSG00000138639 | *ARHGAP24* | 0 | 0.056934 | -2.92562 |
| ENSG00000158578 | *ALAS2* | 1.215926 | 0 | -2.92433 |
| ENSG00000100097 | *LGALS1* | 1.807373 | 0 | -2.92163 |
| ENSG00000088881 | *EBF4* | 0 | 0.102477 | -2.91042 |
| ENSG00000185955 | *C7orf61* | 0.743545 | 0 | -2.91032 |
| ENSG00000163220 | *S100A9* | 0.660178 | 0 | -2.90696 |
| ENSG00000123689 | *G0S2* | 0 | 0.458907 | -2.90219 |
| ENSG00000236656 | *RP11-144L1.4* | 0 | 3.771936 | -2.88968 |
| ENSG00000241679 | *RP11-80H8.4* | 0 | 0.418791 | -2.88848 |
| ENSG00000035720 | *STAP1* | 2.469829 | 0 | -2.85902 |
| ENSG00000174837 | *ADGRE1* | 1.07571 | 0 | -2.84563 |
| ENSG00000136869 | *TLR4* | 0.531003 | 0 | -2.84511 |
| ENSG00000185090 | *MANEAL* | 0.46691 | 0 | -2.84466 |
| ENSG00000183242 | *WT1-AS* | 1.057308 | 0 | -2.84064 |
| ENSG00000245954 | *RP11-18H21.1* | 0 | 0.07211 | -2.83957 |
| ENSG00000162747 | *FCGR3B* | 0.779394 | 0 | -2.83604 |
| ENSG00000204525 | *HLA-C* | 0.171666 | 0 | -2.8327 |
| ENSG00000234534 | *CSNK1G2P1* | 1.086085 | 0 | -2.82779 |
| ENSG00000274978 | *RNU11* | 0 | 0.481875 | -2.82482 |
| ENSG00000073792 | *IGF2BP2* | 0 | 0.589273 | -2.81726 |
| ENSG00000064652 | *SNX24* | 0.361056 | 0 | -2.81062 |
| ENSG00000169429 | *CXCL8* | 0.268837 | 0 | -2.8084 |
| ENSG00000074706 | *IPCEF1* | 1.216759 | 0 | -2.80636 |
| ENSG00000257285 | *RP11-298I3.1* | 0.072284 | 0 | -2.79349 |
| ENSG00000233602 | *ERI3-IT1* | 0.197975 | 0 | -2.79335 |
| ENSG00000096006 | *CRISP3* | 0.22301 | 0 | -2.78893 |
| ENSG00000259767 | *RP11-90B9.2* | 0.093995 | 0 | -2.78266 |
| ENSG00000112208 | *BAG2* | 0 | 0.247157 | -2.78084 |
| ENSG00000243806 | *RPL7P18* | 0 | 0.311647 | -2.77926 |
| ENSG00000255823 | *MTRNR2L8* | 2.401591 | 0 | -2.77595 |
| ENSG00000163736 | *PPBP* | 2.050834 | 0 | -2.77155 |
| ENSG00000211677 | *IGLC2* | 1.43263 | 0 | -2.74933 |
| ENSG00000234389 | *AC007278.3* | 0.893409 | 0 | -2.7472 |
| ENSG00000172264 | *MACROD2* | 0 | 0.749296 | -2.74192 |
| ENSG00000090382 | *LYZ* | 0 | 0.349614 | -2.73863 |
| ENSG00000278989 | *RP11-762L8.6* | 0 | 0.239043 | -2.72235 |
| ENSG00000241230 | *RN7SL801P* | 0.032152 | 0 | -2.72111 |
| ENSG00000003096 | *KLHL13* | 2.18739 | 0 | -2.71448 |
| ENSG00000226989 | *AL049758.2* | 0.02041 | 0 | -2.71374 |
| ENSG00000100079 | *LGALS2* | 0 | 0.506005 | -2.71143 |
| ENSG00000227001 | *NBPF2P* | 0 | 1.357405 | -2.70969 |
| ENSG00000166928 | *MS4A14* | 0.879772 | 0 | -2.70126 |
| ENSG00000180592 | *SKIDA1* | 2.679545 | 0 | -2.69671 |
| ENSG00000196277 | *GRM7* | 2.374547 | 0 | -2.69154 |
| ENSG00000114013 | *CD86* | 0.356024 | 0 | -2.69079 |
| ENSG00000273658 | *uc_338* | 0.250286 | 0 | -2.67819 |
| ENSG00000138678 | *GPAT3* | 0.470511 | 0 | -2.6717 |
| ENSG00000108932 | *SLC16A6* | 0.176976 | 0 | -2.66972 |
| ENSG00000243179 | *AC110769.3* | 0.599642 | 0 | -2.65781 |
| ENSG00000256968 | *SNRPEP2* | 0.210228 | 0 | -2.65636 |
| ENSG00000236779 | *RP11-430C7.2* | 0.398999 | 0 | -2.64726 |
| ENSG00000118113 | *MMP8* | 0.505673 | 0 | -2.64531 |
| ENSG00000134668 | *SPOCD1* | 0 | 1.850191 | -2.634 |
| ENSG00000234617 | *SNRK-AS1* | 0.419569 | 0 | -2.63279 |
| ENSG00000162645 | *GBP2* | 2.049884 | 0 | -2.61174 |
| ENSG00000109452 | *INPP4B* | 1.079448 | 0 | -2.61102 |
| ENSG00000174125 | *TLR1* | 1.638363 | 0 | -2.60729 |
| ENSG00000279415 | *RP11-1102P22.1* | 1.229252 | 0 | -2.6001 |
| ENSG00000173917 | *HOXB2* | 0 | 1.141965 | -2.59982 |
| ENSG00000123843 | *C4BPB* | 0 | 0.442519 | -2.5978 |
| ENSG00000132465 | *JCHAIN* | 1.574386 | 0 | -2.59611 |
| ENSG00000185477 | *GPRIN3* | 1.05699 | 0 | -2.58754 |
| ENSG00000198807 | *PAX9* | 0 | 0.668615 | -2.56734 |
| ENSG00000214870 | *AC004540.5* | 1.423496 | 0 | -2.56435 |
| ENSG00000149054 | *ZNF215* | 0 | 0.822519 | -2.55956 |
| ENSG00000272398 | *CD24* | 0.405202 | 0 | -2.55344 |
| ENSG00000112299 | *VNN1* | 0.864972 | 0 | -2.54974 |
| ENSG00000107796 | *ACTA2* | 2.790389 | 0 | -2.54819 |
| ENSG00000100453 | *GZMB* | 0.199007 | 0 | -2.5396 |
| ENSG00000117477 | *CCDC181* | 0 | 1.008363 | -2.53239 |
| ENSG00000269954 | *RP11-148O21.6* | 0.447826 | 0 | -2.53131 |
| ENSG00000182022 | *CHST15* | 0 | 0.12797 | -2.52915 |
| ENSG00000091106 | *NLRC4* | 1.03303 | 0 | -2.52725 |
| ENSG00000264653 | *MIR5194* | 0 | 0.211322 | -2.51978 |
| ENSG00000212195 | *U3* | 0.16041 | 0 | -2.5192 |
| ENSG00000124570 | *SERPINB6* | 2.595305 | 0 | -2.51756 |
| ENSG00000211679 | *IGLC3* | 1.718335 | 0 | -2.51659 |
| ENSG00000253293 | *HOXA10* | 2.137373 | 0 | -2.51429 |
| ENSG00000124429 | *POF1B* | 2.859997 | 0 | -2.51021 |
| ENSG00000149292 | *TTC12* | 0 | 0.11265 | -2.50588 |
| ENSG00000248994 | *RP11-259O2.1* | 0.687783 | 0 | -2.50169 |
| ENSG00000116701 | *NCF2* | 0.501639 | 0 | -2.49925 |
| ENSG00000248559 | *RP11-215P8.3* | 0.038436 | 0 | -2.49876 |
| ENSG00000100450 | *GZMH* | 0.04871 | 0 | -2.49595 |
| ENSG00000254979 | *RP11-872D17.8* | 0 | 0.75104 | -2.48788 |
| ENSG00000135114 | *OASL* | 0.631169 | 0 | -2.48528 |
| ENSG00000141433 | *ADCYAP1* | 2.049978 | 0 | -2.4819 |
| ENSG00000258101 | *RP11-977B10.2* | 1.216741 | 0 | -2.4791 |
| ENSG00000231621 | *AC013264.2* | 0 | 0.089525 | -2.46917 |
| ENSG00000211662 | *IGLV3-21* | 1.545258 | 0 | -2.46405 |
| ENSG00000019549 | *SNAI2* | 0 | 2.564905 | -2.46362 |
| ENSG00000243498 | *UBA52P5* | 0 | 0.181343 | -2.46138 |
| ENSG00000267546 | *RP11-666A8.8* | 0.296655 | 0 | -2.45614 |
| ENSG00000165168 | *CYBB* | 0.806555 | 0 | -2.44295 |
| ENSG00000213280 | *RP11-212P7.1* | 0 | 0.309228 | -2.44189 |
| ENSG00000197635 | *DPP4* | 0 | 0.77921 | -2.43989 |
| ENSG00000090376 | *IRAK3* | 1.565127 | 0 | -2.43791 |
| ENSG00000204682 | *CASC10* | 2.28613 | 0 | -2.43507 |
| ENSG00000070214 | *SLC44A1* | 0.510932 | 0 | -2.42594 |
| ENSG00000136193 | *SCRN1* | 0 | 0.306259 | -2.42315 |
| ENSG00000185527 | *PDE6G* | 1.076481 | 0 | -2.41623 |
| ENSG00000154864 | *PIEZO2* | 2.578161 | 0 | -2.41293 |
| ENSG00000157388 | *CACNA1D* | 0 | 0.633413 | -2.41041 |
| ENSG00000227730 | *MTND6P5* | 0 | 0.00081 | -2.40455 |
| ENSG00000108702 | *CCL1* | 4.630407 | 0 | -2.39899 |
| ENSG00000277887 | *SNORA50C* | 0.632903 | 0 | -2.39443 |
| ENSG00000119917 | *IFIT3* | 1.022804 | 0 | -2.39152 |
| ENSG00000132541 | *RIDA* | 0 | 0.053601 | -2.39113 |
| ENSG00000278351 | *RP11-139E19.3* | 0 | 1.09936 | -2.37716 |
| ENSG00000233547 | *RP11-57H14.2* | 0.509516 | 0 | -2.37634 |
| ENSG00000184937 | *WT1* | 0.672516 | 0 | -2.37608 |
| ENSG00000065534 | *MYLK* | 0.083097 | 0 | -2.37392 |
| ENSG00000141086 | *CTRL* | 0 | 0.400549 | -2.3727 |
| ENSG00000172164 | *SNTB1* | 0 | 0.408071 | -2.37158 |
| ENSG00000228643 | *AC079779.4* | 0.662892 | 0 | -2.3689 |
| ENSG00000254114 | *HMGN1P28* | 0.192511 | 0 | -2.36514 |
| ENSG00000172260 | *NEGR1* | 1.138878 | 0 | -2.36269 |
| ENSG00000142794 | *NBPF3* | 0 | 1.00326 | -2.35468 |
| ENSG00000005187 | *ACSM3* | 0.206142 | 0 | -2.35026 |
| ENSG00000275014 | *RN7SL166P* | 0 | 0.33769 | -2.3424 |
| ENSG00000160791 | *CCR5* | 1.32968 | 0 | -2.33865 |
| ENSG00000248015 | *AC005329.7* | 0.61317 | 0 | -2.3377 |
| ENSG00000207405 | *SNORA64* | 0.289215 | 0 | -2.33375 |
| ENSG00000227560 | *RPS15AP30* | 0.168729 | 0 | -2.33372 |
| ENSG00000249464 | *LINC01091* | 2.0472 | 0 | -2.32975 |
| ENSG00000244437 | *IGKV3-15* | 1.246219 | 0 | -2.32933 |
| ENSG00000106991 | *ENG* | 2.147756 | 0 | -2.32808 |
| ENSG00000115828 | *QPCT* | 0.126363 | 0 | -2.32716 |
| ENSG00000149294 | *NCAM1* | 3.745164 | 0 | -2.32716 |
| ENSG00000272529 | *RP11-415F23.4* | 0.342192 | 0 | -2.32579 |
| ENSG00000089177 | *KIF16B* | 0.262307 | 0 | -2.32045 |
| ENSG00000264346 | *SNORA77* | 0 | 0.094826 | -2.31984 |
| ENSG00000271893 | *RP11-762E8.1* | 0.854274 | 0 | -2.31597 |
| ENSG00000151917 | *BEND6* | 0.481104 | 0 | -2.31455 |
| ENSG00000262803 | *RP11-160A9.3* | 0.459534 | 0 | -2.31288 |
| ENSG00000230756 | *RHOQP3* | 0.53093 | 0 | -2.30666 |
| ENSG00000234160 | *RP11-613M10.6* | 0 | 0.430231 | -2.30645 |
| ENSG00000228340 | *MIR646HG* | 0 | 0.946833 | -2.3022 |
| ENSG00000255733 | *IFNG-AS1* | 1.651629 | 0 | -2.29701 |
| ENSG00000211821 | *TRDV2* | 0 | 0.124291 | -2.29031 |
| ENSG00000251095 | *RP11-115D19.1* | 1.13009 | 0 | -2.28593 |
| ENSG00000104043 | *ATP8B4* | 2.066399 | 0 | -2.28386 |
| ENSG00000106772 | *PRUNE2* | 0 | 1.031788 | -2.2824 |
| ENSG00000055130 | *CUL1* | 0.601199 | 0 | -2.28078 |
| ENSG00000227502 | *LINC01268* | 0.874708 | 0 | -2.27885 |
| ENSG00000250950 | *RP11-33B1.3* | 0.567237 | 0 | -2.27664 |
| ENSG00000270883 | *RP11-461L13.5* | 0 | 0.107503 | -2.27651 |
| ENSG00000172878 | *METAP1D* | 0 | 0.22013 | -2.27449 |
| ENSG00000134827 | *TCN1* | 0 | 0.081703 | -2.27195 |
| ENSG00000181616 | *OR52H1* | 0.742506 | 0 | -2.26212 |
| ENSG00000218730 | *RP3-453I5.2* | 0.462271 | 0 | -2.25054 |
| ENSG00000186265 | *BTLA* | 2.567973 | 0 | -2.24232 |
| ENSG00000263417 | *GTSCR1* | 1.856038 | 0 | -2.24184 |
| ENSG00000228651 | *RP11-556E13.1* | 1.128945 | 0 | -2.2406 |
| ENSG00000186407 | *CD300E* | 0 | 0.291664 | -2.23862 |
| ENSG00000201348 | *RNU105B* | 0.333881 | 0 | -2.23523 |
| ENSG00000075420 | *FNDC3B* | 1.284191 | 0 | -2.23457 |
| ENSG00000163823 | *CCR1* | 1.340237 | 0 | -2.2298 |
| ENSG00000102984 | *ZNF821* | 0.537828 | 0 | -2.22968 |
| ENSG00000236283 | *AC013463.2* | 0 | 1.841992 | -2.22931 |
| ENSG00000165060 | *FXN* | 0 | 0.012556 | -2.22701 |
| ENSG00000275645 | *RP11-817O13.9* | 0.022255 | 0 | -2.22625 |
| ENSG00000162882 | *HAAO* | 1.854255 | 0 | -2.22458 |
| ENSG00000272777 | *RP11-571L19.8* | 0 | 0.368443 | -2.21428 |
| ENSG00000168811 | *IL12A* | 1.188244 | 0 | -2.20372 |
| ENSG00000269243 | *CTD-2231E14.8* | 2.223395 | 0 | -2.20305 |
| ENSG00000115738 | *ID2* | 2.083285 | 0 | -2.20304 |
| ENSG00000253210 | *RP11-809O17.1* | 0.328553 | 0 | -2.19486 |
| ENSG00000188820 | *FAM26F* | 0.365721 | 0 | -2.1937 |
| ENSG00000138411 | *HECW2* | 1.856135 | 0 | -2.1895 |
| ENSG00000136531 | *SCN2A* | 0 | 1.393478 | -2.18944 |
| ENSG00000264269 | *RP11-15F12.1* | 0 | 1.396338 | -2.18699 |
| ENSG00000198585 | *NUDT16* | 1.223209 | 0 | -2.18585 |
| ENSG00000118777 | *ABCG2* | 0 | 0.273193 | -2.18364 |
| ENSG00000082397 | *EPB41L3* | 0 | 0.552354 | -2.17068 |
| ENSG00000211793 | *TRAV9-2* | 0 | 0.022978 | -2.16465 |
| ENSG00000224739 | *AC016735.1* | 1.861885 | 0 | -2.16393 |
| ENSG00000216316 | *RP3-354N19.3* | 2.158438 | 0 | -2.16201 |
| ENSG00000183691 | *NOG* | 1.579561 | 0 | -2.16096 |
| ENSG00000211699 | *TRGV3* | 0 | 0.359276 | -2.1537 |
| ENSG00000258227 | *CLEC5A* | 0 | 0.079433 | -2.15229 |
| ENSG00000279430 | *RP11-190A12.9* | 0.266566 | 0 | -2.14965 |
| ENSG00000118520 | *ARG1* | 0.164929 | 0 | -2.14292 |
| ENSG00000266237 | *RP11-25D3.1* | 0 | 0.10166 | -2.14061 |
| ENSG00000126262 | *FFAR2* | 0 | 0.266221 | -2.13923 |
| ENSG00000273108 | *RP11-416N2.4* | 2.593984 | 0 | -2.13611 |
| ENSG00000168229 | *PTGDR* | 0.348365 | 0 | -2.13425 |
| ENSG00000230823 | *CBX1P1* | 0 | 0.437571 | -2.13381 |
| ENSG00000120327 | *PCDHB14* | 0 | 0.595694 | -2.12475 |
| ENSG00000124019 | *FAM124B* | 0.410418 | 0 | -2.12431 |
| ENSG00000207205 | *RNVU1-15* | 1.672493 | 0 | -2.12247 |
| ENSG00000227118 | *BTF3P13* | 0 | 0.573703 | -2.12211 |
| ENSG00000267858 | *MZF1-AS1* | 0.419376 | 0 | -2.11549 |
| ENSG00000211592 | *IGKC* | 1.339537 | 0 | -2.10731 |
| ENSG00000155792 | *DEPTOR* | 0.824955 | 0 | -2.10639 |
| ENSG00000042980 | *ADAM28* | 1.583451 | 0 | -2.10395 |
| ENSG00000238160 | *AC116366.5* | 0 | 0.155838 | -2.10321 |
| ENSG00000100504 | *PYGL* | 1.468992 | 0 | -2.10157 |
| ENSG00000250135 | *RP4-622L5.2* | 0.113626 | 0 | -2.09984 |
| ENSG00000236525 | *AC007278.2* | 1.129167 | 0 | -2.09794 |
| ENSG00000138722 | *MMRN1* | 2.427299 | 0 | -2.09628 |
| ENSG00000121594 | *CD80* | 0.749303 | 0 | -2.09242 |
| ENSG00000163412 | *EIF4E3* | 0 | 0.653456 | -2.08975 |
| ENSG00000139278 | *GLIPR1* | 1.061663 | 0 | -2.08468 |
| ENSG00000136305 | *CIDEB* | 1.227632 | 0 | -2.08181 |
| ENSG00000214067 | *RP11-549L6.2* | 0 | 1.077682 | -2.08022 |
| ENSG00000115380 | *EFEMP1* | 0 | 3.237982 | -2.07838 |
| ENSG00000226751 | *AF127936.5* | 0.630177 | 0 | -2.07837 |
| ENSG00000060140 | *STYK1* | 0.466887 | 0 | -2.07527 |
| ENSG00000243094 | *RP11-1084A12.1* | 0.329454 | 0 | -2.07302 |
| ENSG00000258476 | *LINC02207* | 0 | 0.458105 | -2.06805 |
| ENSG00000163666 | *HESX1* | 0 | 0.106241 | -2.06538 |
| ENSG00000249057 | *MAST4-IT1* | 0.06253 | 0 | -2.06245 |
| ENSG00000214439 | *FAM185BP* | 0.50542 | 0 | -2.06168 |
| ENSG00000138772 | *ANXA3* | 0.53745 | 0 | -2.0597 |
| ENSG00000115165 | *CYTIP* | 1.627131 | 0 | -2.05963 |
| ENSG00000179144 | *GIMAP7* | 0.475501 | 0 | -2.05128 |
| ENSG00000228125 | *AKIRIN1P2* | 0.258284 | 0 | -2.04998 |
| ENSG00000254272 | *RP11-382J24.2* | 0.316795 | 0 | -2.049 |
| ENSG00000154319 | *FAM167A* | 1.740236 | 0 | -2.04538 |
| ENSG00000224388 | *BACE2-IT1* | 0.036544 | 0 | -2.04203 |
| ENSG00000256861 | *RP11-512M8.5* | 0 | 0.425797 | -2.04032 |
| ENSG00000167613 | *LAIR1* | 1.03 | 0 | -2.03755 |
| ENSG00000269925 | *RP3-467L1.6* | 0.359766 | 0 | -2.03446 |
| ENSG00000267430 | *RP11-635N19.2* | 0 | 0.012503 | -2.03264 |
| ENSG00000117228 | *GBP1* | 1.511687 | 0 | -2.03067 |
| ENSG00000142089 | *IFITM3* | 0.027525 | 0 | -2.03007 |
| ENSG00000228439 | *TSTD3* | 0 | 0.785272 | -2.02497 |
| ENSG00000270640 | *RP11-373D23.2* | 1.125295 | 0 | -2.02483 |
| ENSG00000251046 | *ZNF969P* | 0.718086 | 0 | -2.02369 |
| ENSG00000155629 | *PIK3AP1* | 1.574562 | 0 | -2.02264 |
| ENSG00000248161 | *RP11-499E18.1* | 0.742808 | 0 | -2.01825 |
| ENSG00000201558 | *RNVU1-6* | 0.053854 | 0 | -2.01519 |
| ENSG00000112303 | *VNN2* | 0.196882 | 0 | -2.0142 |
| ENSG00000250731 | *TPM3P6* | 0.254783 | 0 | -2.01017 |
| ENSG00000136634 | *IL10* | 2.990357 | 0 | -2.00098 |

**Supplementary Table 4: List of differentially expressed epigenetic modifiers in T-ALL**

| **Ensembl_ID** | **Gene Name** | **Fold change in T-ALL subtypes compared to normal thymus** | | |
| --- | --- | --- | --- | --- |
|  |  | **Immature** | **Cortical** | **Mature** |
| ENSG00000116539 | *ASH1L* | 3.642687 | 3.193563 | 5.571508 |
| ENSG00000149311 | *ATM* | 2.613504 | 3.283229 | 4.111753 |
| ENSG00000175054 | *ATR* | 3.385774 | 3.56361 | 2.8761 |
| ENSG00000178999 | *AURKB* | 0.163848 | 0.276891 | 0.512956 |
| ENSG00000009954 | *BAZ1B* | 1.847294 | 2.619659 | 2.021109 |
| ENSG00000185515 | *BRCC3* | 2.439942 | 3.105225 | 1.762091 |
| ENSG00000169679 | *BUB1* | 0.98646 | 1.993995 | 1.630197 |
| ENSG00000142453 | *CARM1* | 0.439167 | 0.358184 | 0.418145 |
| ENSG00000059758 | *CDK17* | 3.104329 | 2.790101 | 2.528206 |
| ENSG00000250506 | *CDK3* | 0.896619 | 0.835064 | 1.00085 |
| ENSG00000164885 | *CDK5* | 0.604542 | 0.654752 | 0.442145 |
| ENSG00000153046 | *CDYL* | 6.617546 | 4.214558 | 5.52929 |
| ENSG00000213341 | *CHUK* | 1.098208 | 1.117971 | 0.786397 |
| ENSG00000134852 | *CLOCK* | 0.869758 | 1.165048 | 1.006908 |
| ENSG00000005339 | *CREBBP* | 0.928932 | 0.9392 | 1.217274 |
| ENSG00000167657 | *DAPK3* | 0.831876 | 0.569725 | 0.685105 |
| ENSG00000104885 | *DOT1L* | 1.569123 | 1.059403 | 2.872172 |
| ENSG00000163840 | *DTX3L* | 2.517655 | 2.04019 | 2.104309 |
| ENSG00000120129 | *DUSP1* | 0.563203 | 0.514235 | 0.584927 |
| ENSG00000181090 | *EHMT1* | 1.970725 | 1.870916 | 2.342637 |
| ENSG00000134014 | *ELP3* | 2.699151 | 2.831862 | 2.140573 |
| ENSG00000100393 | *EP300* | 1.762245 | 1.409945 | 4.070125 |
| ENSG00000104313 | *EYA1* | 0.040325 | 0.040313 | 0 |
| ENSG00000064655 | *EYA2* | 0.000343 | 0 | 0 |
| ENSG00000158161 | *EYA3* | 1.727238 | 1.742018 | 1.307396 |
| ENSG00000108799 | *EZH1* | 1.001062 | 0.876191 | 0.90362 |
| ENSG00000106462 | *EZH2* | 0.527977 | 0.935674 | 0.819429 |
| ENSG00000177602 | *GSG2* | 0.487744 | 0.806718 | 1.081622 |
| ENSG00000082701 | *GSK3B* | 3.08943 | 3.996214 | 2.729012 |
| ENSG00000125484 | *GTF3C4* | 1.414599 | 1.594837 | 1.934299 |
| ENSG00000128708 | *HAT1* | 1.268752 | 1.753461 | 1.211899 |
| ENSG00000116478 | *HDAC1* | 0.427962 | 0.453005 | 0.333542 |
| ENSG00000100429 | *HDAC10* | 0.532417 | 0.197155 | 1.396719 |
| ENSG00000163517 | *HDAC11* | 0.489341 | 0.362187 | 0.370098 |
| ENSG00000196591 | *HDAC2* | 0.78296 | 0.873585 | 1.440213 |
| ENSG00000171720 | *HDAC3* | 0.422535 | 0.478496 | 0.383122 |
| ENSG00000068024 | *HDAC4* | 3.487609 | 5.10675 | 4.18151 |
| ENSG00000108840 | *HDAC5* | 0.731626 | 0.291268 | 1.043193 |
| ENSG00000094631 | *HDAC6* | 1.435091 | 1.080747 | 1.423007 |
| ENSG00000061273 | *HDAC7* | 0.345108 | 0.20562 | 1.186658 |
| ENSG00000147099 | *HDAC8* | 2.15911 | 1.693502 | 1.249144 |
| ENSG00000048052 | *HDAC9* | 9.580054 | 1.5455 | 2.626683 |
| ENSG00000159267 | *HLCS* | 2.754417 | 3.168554 | 1.329005 |
| ENSG00000086758 | *HUWE1* | 1.243103 | 1.328356 | 0.931505 |
| ENSG00000096968 | *JAK2* | 5.160092 | 3.373724 | 3.725367 |
| ENSG00000070495 | *JMJD6* | 0.981112 | 0.688942 | 0.525091 |
| ENSG00000108773 | *KAT2A* | 0.944002 | 0.615023 | 0.804913 |
| ENSG00000114166 | *KAT2B* | 0.698657 | 0.935126 | 0.453876 |
| ENSG00000172977 | *KAT5* | 0.670752 | 0.579984 | 0.609824 |
| ENSG00000004487 | *KDM1A* | 1.424779 | 1.682207 | 1.345008 |
| ENSG00000165097 | *KDM1B* | 1.230621 | 0.763675 | 0.539077 |
| ENSG00000173120 | *KDM2A* | 1.410758 | 1.340711 | 1.751358 |
| ENSG00000089094 | *KDM2B* | 1.312901 | 0.994061 | 2.783134 |
| ENSG00000115548 | *KDM3A* | 3.610185 | 2.858037 | 3.071289 |
| ENSG00000120733 | *KDM3B* | 0.85362 | 1.047881 | 1.152454 |
| ENSG00000066135 | *KDM4A* | 1.530221 | 1.475161 | 1.046249 |
| ENSG00000127663 | *KDM4B* | 1.465628 | 0.855259 | 3.186935 |
| ENSG00000107077 | *KDM4C* | 3.525156 | 3.411261 | 2.518964 |
| ENSG00000186280 | *KDM4D* | 0.448988 | 0.427718 | 0.045077 |
| ENSG00000073614 | *KDM5A* | 4.071739 | 4.438231 | 4.075337 |
| ENSG00000117139 | *KDM5B* | 3.119164 | 2.343263 | 3.593122 |
| ENSG00000126012 | *KDM5C* | 1.346236 | 1.060484 | 0.944099 |
| ENSG00000012817 | *KDM5D* | 1.345221 | 1.460088 | 1.487666 |
| ENSG00000147050 | *KDM6A* | 3.36311 | 2.641864 | 2.174633 |
| ENSG00000132510 | *KDM6B* | 0.636768 | 0.292723 | 1.607246 |
| ENSG00000182541 | *LIMK2* | 0.65388 | 0.610135 | 1.215669 |
| ENSG00000107968 | *MAP3K8* | 4.956923 | 2.000809 | 1.331842 |
| ENSG00000120539 | *MASTL* | 2.024594 | 3.766166 | 2.762154 |
| ENSG00000198408 | *MGEA5* | 1.485764 | 1.41486 | 1.411248 |
| ENSG00000162601 | *MYSM1* | 3.040849 | 3.179126 | 2.306519 |
| ENSG00000135372 | *NAT10* | 1.657484 | 1.663951 | 1.714987 |
| ENSG00000084676 | *NCOA1* | 2.552649 | 2.551289 | 2.885874 |
| ENSG00000124151 | *NCOA3* | 2.921242 | 2.412059 | 3.5801 |
| ENSG00000119408 | *NEK6* | 0.47185 | 0.11369 | 0.050697 |
| ENSG00000119638 | *NEK9* | 1.164554 | 0.98799 | 0.919295 |
| ENSG00000165671 | *NSD1* | 4.936615 | 5.847983 | 4.9437 |
| ENSG00000159339 | *PADI4* | 1.667039 | 0.772615 | 1.940257 |
| ENSG00000180370 | *PAK2* | 0.805783 | 0.879026 | 1.101362 |
| ENSG00000227345 | *PARG* | 2.400443 | 2.91329 | 2.224023 |
| ENSG00000143799 | *PARP1* | 1.038073 | 0.864497 | 0.592757 |
| ENSG00000172943 | *PHF8* | 1.196945 | 1.023929 | 0.995473 |
| ENSG00000123143 | *PKN1* | 1.284717 | 0.488986 | 5.964248 |
| ENSG00000170836 | *PPM1D* | 2.333324 | 2.258466 | 3.651304 |
| ENSG00000186298 | *PPP1CC* | 1.348795 | 1.911566 | 1.581206 |
| ENSG00000113575 | *PPP2CA* | 1.037492 | 1.548534 | 0.983023 |
| ENSG00000104695 | *PPP2CB* | 0.520586 | 0.549928 | 0.41922 |
| ENSG00000149923 | *PPP4C* | 0.606487 | 0.482944 | 2.035733 |
| ENSG00000011485 | *PPP5C* | 0.532216 | 0.753687 | 0.445883 |
| ENSG00000116731 | *PRDM2* | 2.142967 | 1.871866 | 2.903901 |
| ENSG00000166501 | *PRKCB* | 3.47136 | 2.685418 | 3.615516 |
| ENSG00000163932 | *PRKCD* | 0.479242 | 0.338221 | 0.345291 |
| ENSG00000253729 | *PRKDC* | 1.030337 | 1.3944 | 0.975975 |
| ENSG00000126457 | *PRMT1* | 0.77998 | 0.890605 | 0.694485 |
| ENSG00000160310 | *PRMT2* | 1.194677 | 0.854393 | 1.135363 |
| ENSG00000100462 | *PRMT5* | 1.229882 | 1.417755 | 1.51995 |
| ENSG00000198890 | *PRMT6* | 1.044083 | 1.011793 | 1.138412 |
| ENSG00000132600 | *PRMT7* | 0.56592 | 0.51314 | 0.793796 |
| ENSG00000166349 | *RAG1* | 0.056406 | 0.194757 | 0.080342 |
| ENSG00000100387 | *RBX1* | 0.396043 | 0.508224 | 0.299145 |
| ENSG00000163961 | *RNF168* | 10.60118 | 4.516607 | 8.655487 |
| ENSG00000121481 | *RNF2* | 3.073485 | 2.875637 | 1.93683 |
| ENSG00000155827 | *RNF20* | 2.463231 | 2.472633 | 1.742731 |
| ENSG00000103549 | *RNF40* | 1.331546 | 0.914096 | 0.998083 |
| ENSG00000112130 | *RNF8* | 2.197736 | 2.140424 | 1.429868 |
| ENSG00000162302 | *RPS6KA4* | 0.950111 | 0.417165 | 1.844956 |
| ENSG00000100784 | *RPS6KA5* | 2.655089 | 1.657355 | 4.403063 |
| ENSG00000099381 | *SETD1A* | 0.6987 | 0.666484 | 1.615169 |
| ENSG00000139718 | *SETD1B* | 0.666749 | 0.302682 | 1.705067 |
| ENSG00000181555 | *SETD2* | 4.005065 | 3.809482 | 3.440873 |
| ENSG00000145391 | *SETD7* | 4.267706 | 3.423692 | 1.754546 |
| ENSG00000143379 | *SETDB1* | 1.171516 | 1.043556 | 1.569009 |
| ENSG00000136169 | *SETDB2* | 3.8942 | 3.429886 | 2.78734 |
| ENSG00000170364 | *SETMAR* | 2.020022 | 1.498942 | 3.328134 |
| ENSG00000096717 | *SIRT1* | 1.697107 | 1.426441 | 1.939948 |
| ENSG00000068903 | *SIRT2* | 0.344055 | 0.231672 | 0.624233 |
| ENSG00000142082 | *SIRT3* | 0.72149 | 0.41616 | 1.090486 |
| ENSG00000077463 | *SIRT6* | 0.329913 | 0.260553 | 0.956189 |
| ENSG00000143499 | *SMYD2* | 0.869272 | 1.36822 | 0.980077 |
| ENSG00000185420 | *SMYD3* | 9.417278 | 3.317089 | 2.284052 |
| ENSG00000072786 | *STK10* | 1.957565 | 1.184424 | 2.48089 |
| ENSG00000101109 | *STK4* | 4.20366 | 3.27301 | 4.106149 |
| ENSG00000101945 | *SUV39H1* | 0.686009 | 0.642332 | 0.857477 |
| ENSG00000152455 | *SUV39H2* | 1.843242 | 2.724186 | 1.352424 |
| ENSG00000198586 | *TLK1* | 2.873522 | 2.553098 | 3.339644 |
| ENSG00000024048 | *UBR2* | 2.736301 | 2.113428 | 2.851414 |
| ENSG00000276043 | *UHRF1* | 0.833159 | 1.176305 | 0.839216 |
| ENSG00000156256 | *USP16* | 2.096304 | 2.470392 | 1.974231 |
| ENSG00000124422 | *USP22* | 1.454566 | 1.306445 | 1.605142 |
| ENSG00000140455 | *USP3* | 2.911197 | 2.827871 | 2.456724 |

**Supplementary Table 5: List of differentially expressed lncRNA in T-ALL subtypes.**

| **Ensembl_ID** | **lncRNA** | **Log2 Fold change in T-ALL subtypes compared to normal thymus** | | |
| --- | --- | --- | --- | --- |
|  |  | **Immature** | **Cortical** | **Mature** |
| ENSG00000227331 | *AC005042.2* | 2.932991 | 3.774302 | 4.600496 |
| ENSG00000239763 | *AC009120.3* | 0.698076 | 0.970412 | 0.717067 |
| ENSG00000232693 | *AC012370.2* | 2.813977 | 5.31884 | 3.531598 |
| ENSG00000226548 | *AC016722.3* | 1.633804 | -0.975 | 5.679268 |
| ENSG00000224739 | *AC016735.1* | 2.797128 | 0.935243 | -1.22869 |
| ENSG00000224959 | *AC017002.2* | 5.815676 | 6.524154 | 5.034563 |
| ENSG00000262477 | *AC021224.1* | -12.2362 | -11.0897 | 0 |
| ENSG00000255397 | *AC022182.2* | 0.556568 | 1.106803 | 0.800372 |
| ENSG00000214135 | *AC024560.3* | -0.07701 | -0.12241 | 0.260385 |
| ENSG00000226380 | *AC058791.1* | 4.116229 | 3.439109 | 4.61197 |
| ENSG00000235127 | *AC068286.1* | 2.638447 | 0 | 4.96537 |
| ENSG00000223973 | *AC068491.3* | 0.879836 | 0.84236 | 1.0999 |
| ENSG00000213963 | *AC074286.1* | 3.477998 | 3.489017 | 3.863428 |
| ENSG00000231747 | *AC079922.2* | -0.16698 | 0.609525 | 0.315806 |
| ENSG00000259820 | *AC083843.1* | 0.461917 | -0.01446 | -0.57815 |
| ENSG00000226791 | *AC109826.1* | 1.511781 | 2.468757 | 1.633702 |
| ENSG00000273117 | *AC144652.1* | 2.134686 | 2.050892 | 2.626344 |
| ENSG00000230606 | *AC159540.1* | 7.539011 | 7.727775 | 8.612216 |
| ENSG00000255139 | *AP000442.1* | -3.76796 | -3.27675 | -3.55748 |
| ENSG00000214293 | *APTR* | -0.16652 | -0.07096 | -0.52824 |
| ENSG00000281357 | *ARRDC3-AS1* | 1.568194 | 1.736157 | 2.583131 |
| ENSG00000228817 | *BACH1-IT2* | 4.748152 | 4.651637 | 4.488058 |
| ENSG00000282851 | *BISPR* | 1.008766 | 0.38259 | 1.713277 |
| ENSG00000213057 | *C1orf220* | 0.21744 | 0.272484 | 1.792338 |
| ENSG00000176659 | *C20orf197* | 0.30855 | 0.797904 | -0.37878 |
| ENSG00000188511 | *C22orf34* | 2.729814 | 2.064249 | 3.466017 |
| ENSG00000163009 | *C2orf48* | 0.340253 | -0.60312 | 3.284385 |
| ENSG00000270419 | *CAHM* | 1.243426 | 0.639937 | 1.783546 |
| ENSG00000272168 | *CASC15* | 3.386979 | 3.698911 | 4.191606 |
| ENSG00000229140 | *CCDC26* | 2.562204 | 3.422968 | -3.23495 |
| ENSG00000245694 | *CRNDE* | 1.475955 | 1.898117 | 1.796821 |
| ENSG00000222041 | *CYTOR* | 1.457916 | 1.695482 | 0.315568 |
| ENSG00000243701 | *DUBR* | 1.828023 | 2.686994 | 1.450325 |
| ENSG00000281649 | *EBLN3P* | 1.096467 | 1.078982 | 0.660708 |
| ENSG00000224032 | *EPB41L4A-AS1* | 1.105995 | 1.081136 | 2.221763 |
| ENSG00000233056 | *ERVH48-1* | 0.3328 | 2.508702 | 1.061236 |
| ENSG00000213468 | *FIRRE* | 3.145087 | 3.427043 | 3.438352 |
| ENSG00000237424 | *FOXD2-AS1* | -0.14775 | 0.056302 | -0.11992 |
| ENSG00000230590 | *FTX* | 3.003023 | 2.681669 | 3.004246 |
| ENSG00000228223 | *HCG11* | 1.331819 | 1.553069 | 1.208296 |
| ENSG00000259673 | *IQCH-AS1* | 2.102193 | 1.480699 | 0.839138 |
| ENSG00000225470 | *JPX* | 2.148687 | 1.742998 | 2.787898 |
| ENSG00000122548 | *KIAA0087* | 0.641654 | -5.14294 | 0 |
| ENSG00000225880 | *LINC00115* | -1.34969 | -1.42763 | 0.147018 |
| ENSG00000188185 | *LINC00265* | -1.66744 | -1.85622 | 0.230311 |
| ENSG00000231535 | *LINC00278* | 3.401601 | 3.347438 | 3.747079 |
| ENSG00000280798 | *LINC00294* | -1.26231 | -1.47121 | -1.47523 |
| ENSG00000180422 | *LINC00304* | 2.551673 | 0.517209 | 4.43214 |
| ENSG00000227456 | *LINC00310* | 3.305547 | 3.547423 | 3.859362 |
| ENSG00000232931 | *LINC00342* | 2.471908 | 2.273929 | 4.099299 |
| ENSG00000238121 | *LINC00426* | 1.793813 | 1.744843 | 2.039454 |
| ENSG00000236463 | *LINC00427* | 2.51759 | 2.652946 | 2.264386 |
| ENSG00000245526 | *LINC00461* | 2.19888 | 4.835579 | 0.966021 |
| ENSG00000153363 | *LINC00467* | 1.51394 | 1.812239 | 2.682272 |
| ENSG00000223414 | *LINC00473* | 2.777535 | 5.541343 | 5.576202 |
| ENSG00000175611 | *LINC00476* | 1.225775 | 1.112961 | 1.528567 |
| ENSG00000235621 | *LINC00494* | -1.82575 | -0.48988 | -1.01738 |
| ENSG00000264575 | *LINC00526* | -0.6669 | -0.51449 | -0.51024 |
| ENSG00000269220 | *LINC00528* | 1.293252 | 1.136749 | 2.709948 |
| ENSG00000224429 | *LINC00539* | 3.818731 | 2.477256 | 3.199852 |
| ENSG00000226067 | *LINC00623* | -1.35649 | -0.33795 | -0.15991 |
| ENSG00000223546 | *LINC00630* | 3.536068 | 3.583367 | 3.476922 |
| ENSG00000258701 | *LINC00638* | 0.839541 | -0.80001 | 1.484877 |
| ENSG00000205181 | *LINC00654* | 1.833706 | -0.27447 | 3.317776 |
| ENSG00000261824 | *LINC00662* | 2.631091 | 2.109872 | 2.926292 |
| ENSG00000266904 | *LINC00663* | -0.93786 | -1.11107 | 0.206406 |
| ENSG00000268658 | *LINC00664* | 3.162625 | 2.893765 | 4.937045 |
| ENSG00000232677 | *LINC00665* | 0.68562 | 0.281899 | 2.569688 |
| ENSG00000263753 | *LINC00667* | 0.827433 | 0.573636 | 1.703319 |
| ENSG00000178440 | *LINC00843* | 0.468087 | 1.079188 | 0.754797 |
| ENSG00000245060 | *LINC00847* | -0.5376 | -0.81935 | -0.27201 |
| ENSG00000245164 | *LINC00861* | 0.477573 | 0.815892 | -0.11345 |
| ENSG00000232229 | *LINC00865* | 1.012706 | 0.168867 | 1.360354 |
| ENSG00000241163 | *LINC00877* | 2.974616 | 2.636635 | 1.497501 |
| ENSG00000242759 | *LINC00882* | 0.805195 | -0.39446 | 3.813535 |
| ENSG00000214145 | *LINC00887* | 7.142623 | 6.809893 | 3.721449 |
| ENSG00000264247 | *LINC00909* | 0.147302 | -0.14438 | 0.574371 |
| ENSG00000188825 | *LINC00910* | 1.124873 | 0.978184 | 2.516653 |
| ENSG00000246898 | *LINC00920* | 1.817633 | 2.446649 | 2.224922 |
| ENSG00000281005 | *LINC00921* | 2.246449 | 1.555212 | 3.546278 |
| ENSG00000247982 | *LINC00926* | 0.031533 | -0.02325 | 1.286947 |
| ENSG00000226091 | *LINC00937* | 0.741674 | -0.73063 | 5.249397 |
| ENSG00000256128 | *LINC00944* | 1.344332 | 2.736731 | 0 |
| ENSG00000228784 | *LINC00954* | -0.38332 | 0.263777 | 0.712249 |
| ENSG00000242516 | *LINC00960* | 2.901441 | 4.531055 | 0 |
| ENSG00000261455 | *LINC01003* | 0.894003 | 0.378122 | 2.16915 |
| ENSG00000182648 | *LINC01006* | -2.45979 | -2.8305 | 1.637241 |
| ENSG00000225039 | *LINC01058* | -0.21084 | 0.215363 | 1.10637 |
| ENSG00000212694 | *LINC01089* | -0.18509 | -0.13178 | 1.582633 |
| ENSG00000281202 | *LINC01097* | 4.480884 | 5.010196 | 6.605496 |
| ENSG00000226673 | *LINC01108* | 6.401126 | 0.902826 | 1.966358 |
| ENSG00000223631 | *LINC01120* | 0.841827 | 2.969687 | 2.708117 |
| ENSG00000279873 | *LINC01126* | 0.954952 | 0.496954 | 1.3628 |
| ENSG00000281404 | *LINC01176* | 0.867698 | 0.562569 | 0.99836 |
| ENSG00000245937 | *LINC01184* | 0.406949 | 0.79064 | 0.597154 |
| ENSG00000271856 | *LINC01215* | 1.811963 | 2.375912 | 1.730853 |
| ENSG00000235492 | *LINC01221* | 7.527694 | 9.211739 | 7.729538 |
| ENSG00000233410 | *LINC01222* | 7.572938 | 8.405591 | 8.263786 |
| ENSG00000269416 | *LINC01224* | 5.185335 | 4.68585 | 7.095525 |
| ENSG00000132832 | *LINC01260* | 0.096476 | 1.060684 | -0.36856 |
| ENSG00000227502 | *LINC01268* | 5.290933 | 4.416226 | 2.137375 |
| ENSG00000231671 | *LINC01307* | 2.432817 | 4.057123 | 2.093081 |
| ENSG00000229891 | *LINC01315* | -2.63136 | -3.52258 | 1.153317 |
| ENSG00000250889 | *LINC01336* | 0.415544 | 0.785142 | 1.512663 |
| ENSG00000261326 | *LINC01355* | -0.3957 | -0.28326 | 0.276923 |
| ENSG00000261326 | *LINC01355* | -0.3957 | -0.28326 | 0.276923 |
| ENSG00000235172 | *LINC01366* | 0.382122 | 1.608193 | 1.244407 |
| ENSG00000235475 | *LINC01372* | 2.343911 | 1.81633 | 3.0811 |
| ENSG00000280560 | *LINC01374* | 4.447466 | -1.38746 | 0 |
| ENSG00000238113 | *LINC01410* | 2.232603 | 1.926629 | 1.682717 |
| ENSG00000223704 | *LINC01422* | 2.492124 | 1.778806 | 4.712832 |
| ENSG00000231106 | *LINC01436* | 1.218094 | -1.31923 | -1.82105 |
| ENSG00000237877 | *LINC01473* | 3.097933 | 2.777982 | 1.630861 |
| ENSG00000257613 | *LINC01481* | 2.912202 | 2.465754 | 3.157365 |
| ENSG00000257815 | *LINC01481* | 1.304183 | 1.08526 | 1.610553 |
| ENSG00000233901 | *LINC01503* | -3.85476 | -3.36186 | -2.61819 |
| ENSG00000228065 | *LINC01515* | 0.835091 | 2.492766 | 0.860057 |
| ENSG00000228065 | *LINC01515* | 0.835091 | 2.492766 | 0.860057 |
| ENSG00000213888 | *LINC01521* | 1.980817 | 1.330745 | 2.931322 |
| ENSG00000225975 | *LINC01534* | 1.352491 | 0.908338 | 0.530779 |
| ENSG00000246223 | *LINC01550* | 1.388931 | 1.966439 | 1.983448 |
| ENSG00000186960 | *LINC01551* | -8.40608 | 0 | 0 |
| ENSG00000196741 | *LINC01560* | -0.30636 | 0.004081 | -0.04922 |
| ENSG00000262468 | *LINC01569* | -0.43902 | -1.11988 | 0.902382 |
| ENSG00000261008 | *LINC01572* | 1.960245 | 2.810466 | 2.849037 |
| ENSG00000214900 | *LINC01588* | -0.80288 | -1.1881 | 1.025908 |
| ENSG00000226029 | *LINC01772* | 0.746627 | 0.313061 | 2.685474 |
| ENSG00000230415 | *LINC01786* | 1.770175 | 1.15192 | 1.386601 |
| ENSG00000267767 | *LINC01801* | 1.809598 | 1.272949 | 0.332628 |
| ENSG00000227403 | *LINC01806* | 2.179375 | 3.004108 | 0.81013 |
| ENSG00000231826 | *LINC01819* | 5.452175 | 5.654693 | 5.522189 |
| ENSG00000235576 | *LINC01871* | -1.35818 | -2.44668 | 0 |
| ENSG00000232164 | *LINC01873* | -0.23245 | -0.96956 | 6.414305 |
| ENSG00000226383 | *LINC01876* | 1.651684 | 3.163096 | 2.764424 |
| ENSG00000231682 | *LINC01891* | -0.19475 | 1.171063 | -1.92096 |
| ENSG00000234663 | *LINC01934* | 2.680171 | 3.751564 | 3.459274 |
| ENSG00000260804 | *LINC01963* | 0.887168 | 0.92888 | 0.580003 |
| ENSG00000204277 | *LINC01993* | -3.4119 | -4.06282 | -0.24116 |
| ENSG00000267321 | *LINC02001* | 0.531313 | 0.309103 | 2.560729 |
| ENSG00000272690 | *LINC02018* | 1.713504 | 1.124276 | 2.820123 |
| ENSG00000273356 | *LINC02019* | 2.451927 | 1.407663 | 4.49044 |
| ENSG00000249846 | *LINC02021* | 2.294061 | 2.014387 | 1.876051 |
| ENSG00000273033 | *LINC02035* | 0.690161 | 0.142278 | 1.004344 |
| ENSG00000248693 | *LINC02100* | 2.05122 | 1.575909 | 0 |
| ENSG00000262155 | *LINC02175* | 2.906905 | 1.498225 | 1.494724 |
| ENSG00000258476 | *LINC02207* | 2.429518 | 2.887623 | 0.361465 |
| ENSG00000251562 | *MALAT1* | 0 | 0 | 7.074527 |
| ENSG00000234608 | *MAPKAPK5-AS1* | 1.070894 | 1.267827 | 0.553125 |
| ENSG00000214548 | *MEG3* | -6.67992 | -5.68749 | -3.12162 |
| ENSG00000225783 | *MIAT* | 2.954316 | 3.481671 | 3.291549 |
| ENSG00000244625 | *MIATNB* | 2.347199 | 2.161742 | 2.313806 |
| ENSG00000234883 | *MIR155HG* | 0.920575 | 1.271183 | -0.54738 |
| ENSG00000229989 | *MIR181A1HG* | 8.49248 | 7.592929 | 6.867928 |
| ENSG00000270069 | *MIR222HG* | 4.000131 | 1.919168 | 1.679099 |
| ENSG00000253522 | *MIR3142HG* | 3.917443 | 1.907069 | 0.196736 |
| ENSG00000228526 | *MIR34AHG* | 0.389222 | -2.86228 | 2.391144 |
| ENSG00000172965 | *MIR4435-2HG* | 2.21598 | 3.239201 | 2.25526 |
| ENSG00000223749 | *MIR503HG* | -0.83278 | 0.195538 | -0.88127 |
| ENSG00000228340 | *MIR646HG* | 0.526948 | 1.473781 | -1.77525 |
| ENSG00000215386 | *MIR99AHG* | -0.52471 | -0.56621 | -0.49645 |
| ENSG00000197182 | *MIRLET7BHG* | 3.025979 | 1.855279 | 3.842981 |
| ENSG00000260455 | *NBAT1* | 0.34644 | 0.870996 | 1.17718 |
| ENSG00000245532 | *NEAT1* | 0.870901 | 0.011393 | 1.543789 |
| ENSG00000260032 | *NORAD* | 1.369106 | 1.161878 | 1.813297 |
| ENSG00000273344 | *PAXIP1-AS1* | -0.29321 | -0.15396 | 0.672515 |
| ENSG00000280623 | *PCAT14* | 1.069984 | 2.877035 | 6.388962 |
| ENSG00000265369 | *PCAT18* | 6.228909 | 6.281365 | 6.302088 |
| ENSG00000264954 | *PRR29-AS1* | 3.405443 | 2.857249 | 4.834126 |
| ENSG00000230487 | *PSMG3-AS1* | -1.47151 | -1.47417 | -0.96681 |
| ENSG00000249859 | *PVT1* | -1.33419 | -0.18123 | -0.61467 |
| ENSG00000257151 | *PWAR6* | 1.72292 | 1.941408 | 1.523459 |
| ENSG00000259905 | *PWRN1* | 6.976892 | 7.547998 | 8.855875 |
| ENSG00000246067 | *RAB30-AS1* | 0.906126 | 0.769566 | 0.954225 |
| ENSG00000273313 | *RBAKDN* | 0.75796 | 0.815851 | 4.551519 |
| ENSG00000205853 | *RFPL3S* | -0.77425 | -1.50835 | -0.5916 |
| ENSG00000232104 | *RFX3-AS1* | 4.053163 | 4.313363 | 3.787701 |
| ENSG00000269900 | *RMRP* | 8.100141 | 7.692535 | 9.942252 |
| ENSG00000277027 | *RMRP* | 13.97274 | 13.50735 | 12.90185 |
| ENSG00000245149 | *RNF139-AS1* | 0.083081 | 0.111251 | 0.042578 |
| ENSG00000234492 | *RPL34-AS1* | -0.27092 | 0.383531 | 1.416252 |
| ENSG00000245556 | *SCAMP1-AS1* | 0.503521 | 0.442648 | 1.205527 |
| ENSG00000268751 | *SCGB1B2P* | 1.7723 | 0.683498 | 5.256806 |
| ENSG00000228878 | *SEPT7-AS1* | 2.51827 | 2.193734 | 1.901337 |
| ENSG00000230438 | *SERPINB9P1* | 2.375045 | 0.158463 | -3.01862 |
| ENSG00000279078 | *SND1-IT1* | 4.890006 | 4.929317 | 4.784475 |
| ENSG00000232956 | *SNHG15* | 0.791149 | 1.038031 | 0.736574 |
| ENSG00000260260 | *SNHG19* | -1.57992 | -0.82854 | -1.99036 |
| ENSG00000266402 | *SNHG25* | 4.397821 | 4.582301 | 3.472634 |
| ENSG00000281398 | *SNHG4* | 1.624382 | 2.149148 | 1.744127 |
| ENSG00000269893 | *SNHG8* | 0.87702 | 1.216863 | 0.604612 |
| ENSG00000255198 | *SNHG9* | -0.61456 | -0.5505 | -0.06429 |
| ENSG00000248508 | *SRP14-AS1* | -0.69847 | -0.4985 | -1.66439 |
| ENSG00000182165 | *TP53TG1* | -1.10307 | -0.75882 | -1.19189 |
| ENSG00000225791 | *TRAM2-AS1* | 1.451466 | 1.093527 | 1.422619 |
| ENSG00000241657 | *TRBV11-2* | 0.114857 | -6.93916 | 0.345168 |
| ENSG00000241657 | *TRBV11-2* | 0.114857 | -6.93916 | 0.345168 |
| ENSG00000229236 | *TTTY10* | 2.848506 | 2.946504 | 2.64883 |
| ENSG00000176728 | *TTTY14* | -0.09675 | -0.05449 | 0.041305 |
| ENSG00000233864 | *TTTY15* | 2.500859 | 2.645211 | 2.753844 |
| ENSG00000237361 | *TUSC8* | -0.19174 | -5.0525 | -4.75439 |
| ENSG00000228889 | *UBAC2-AS1* | 0.613642 | 0.28632 | 2.822351 |
| ENSG00000256073 | *URB1-AS1* | 0.76525 | 0.361409 | 0.155017 |
| ENSG00000229807 | *XIST* | 6.701052 | 5.463554 | -4.88054 |
| ENSG00000267454 | *ZNF582-AS1* | -0.76271 | -1.56151 | -0.92688 |
| ENSG00000166770 | *ZNF667-AS1* | 0.316195 | -0.80376 | 0.552611 |
| ENSG00000230844 | *ZNF674-AS1* | 1.160446 | 1.223625 | 1.822583 |

**Supplementary Table 6:** **Multivariate Cox regression analysis for prognostic association of *BAALC* expression in T-ALL patients in the validation cohort**

| **Variables** | **Overall Survival** | | | **Event free survival** | | | **Relapse free survival** | | |
| --- | --- | --- | --- | --- | --- | --- | --- | --- | --- |
|  | **HR** | **P value** | **95% CI** | **HR** | **P value** | **95% CI** | **HR** | **P value** | **95% CI** |
| **Age** (Pediatrics vs Adults) | 0.310 | 0.322 | 0.031-3.147 | 1.224 | 0.758 | 0.339-4.415 | 1.211 | 0.807 | 0.260-5.651 |
| **Sex** (Male vs Female) | 0.000 | 1.000 | 0.000 | 0.644 | 0.701 | 0.068-6.091 | 0.909 | 0.938 | 0.082-10.111 |
| **TLC** (<50x10^9^/L vs >50x10^9^/L) | 0.920 | 0.919 | 0.187-4.526 | 1.568 | 0.436 | 0.506-4.861 | 2.662 | 0.156 | 0.688-10.297 |
| **NCI risk** (Standard vs high) | 2.566 | 0.422 | 0.257-25.579 | 2.587 | 0.278 | 0.464-14.41 | 0.286 | 0.140 | 0.054-1.508 |
| **Immunophenotype** | | | | | | | | | |
| Immature | REF |  |  | REF |  |  | REF |  |  |
| Cortical | 1.372 | 0.728 | 0.231-8.133 | 0.335 | 0.098 | 0.092-1.224 | 0.269 | 0.096 | 0.057-1.262 |
| Mature | 0.641 | 0.653 | 0.092-4.458 | 0.346 | 0.152 | 0.081-1.478 | 0.224 | 0.125 | 0.033-1.518 |
| **Prednisolone response**  (Sensitive vs resistant) | 0.370 | 0.267 | 0.064-2.141 | **0.238** | **0.042** | **0.060-0.948** | 0.190 | 0.061 | 0.034-1.077 |
| **MRD** (Negative vs Positive) | 0.205 | 0.054 | 0.041-1.028 | **0.184** | **0.017** | **0.046-0.736** | 2.300 | 0.390 | 0.344-15.362 |
| ***BAALC*** (Low vs  High) | **11.572** | **0.007** | **1.975-67.79** | **5.427** | **0.004** | **1.701-17.31** | **7.281** | **0.006** | **1.783-29.729** |

**Supplementary Table 7:** **Multivariate Cox regression analysis for prognostic association of *MEF2C* expression in T-ALL patients in the validation cohort**

| **Variables** | **Overall Survival** | | | **Event free survival** | | | **Relapse free survival** | | |
| --- | --- | --- | --- | --- | --- | --- | --- | --- | --- |
|  | **HR** | **P value** | **95% CI** | **HR** | **P value** | **95% CI** | **HR** | **P value** | **95% CI** |
| **Age** (Pediatrics vs Adults) | 0.467 | 0.478 | 0.057-3.824 | 2.721 | 0.059 | 0.962-7.699 | 2.596 | 0.114 | 0.795-8.474 |
| **Sex** (Male vs Female) | 0.317 | 0.312 | 0.034-2.943 | 0.460 | 0.340 | 0.093-2.267 | 0.747 | 0.729 | 0.144-3.889 |
| **TLC** (<50x10^9^/L vs >50x10^9^/L) | 1.051 | 0.940 | 0.285-3.880 | 1.904 | 0.213 | 0.691-5.244 | 2.684 | 0.097 | 0.837-8.606 |
| **NCI risk** (Standard vs high) | 1.754 | 0.501 | 0.341-9.016 | 1.446 | 0.611 | 0.349-5.997 | 1.775 | 0.516 | 0.314-10.02 |
| **Immunophenotype** | | | | | | | | | |
| Immature | REF |  |  | REF |  |  | REF |  |  |
| Cortical | 0.914 | 0.898 | 0.232-3.600 | 0.377 | 0.089 | 0.122-1.162 | 0.340 | 0.100 | 0.094-1.228 |
| Mature | 0.532 | 0.449 | 0.104-2.726 | 0.395 | 0.149 | 0.112-1.394 | 0.298 | 0.110 | 0.067-1.316 |
| **Prednisolone response**  (Sensitive vs resistant) | 0.863 | 0.854 | 0.180-4.138 | 0.433 | 0.197 | 0.121-1.544 | 0.433 | 0.285 | 0.094-2.007 |
| **MRD** (Negative vs Positive) | 0.321 | 0.102 | 0.082-1.252 | **0.286** | **0.034** | **0.090-0.910** | 0.283 | 0.070 | 0.072-1.110 |
| ***MEF2C*** (Low vs  High) | **4.642** | **0.024** | **1.222-17.63** | **2.752** | **0.049** | **1.003-7.55** | **4.585** | **0.022** | **1.247-16.85** |

**Supplementary Table 8: Multivariate Cox regression analysis for prognostic association of *HHEX* expression in T-ALL patients in the validation cohort**

| **Variables** | **Overall Survival** | | | **Event free survival** | | | **Relapse free survival** | | |
| --- | --- | --- | --- | --- | --- | --- | --- | --- | --- |
|  | **HR** | **P value** | **95% CI** | **HR** | **P value** | **95% CI** | **HR** | **P value** | **95% CI** |
| **Age** (Pediatrics vs Adults) | 0.637 | 0.683 | 0.073-5.547 | 2.759 | 0.075 | 0.901-8.445 | 2.715 | 0.134 | 0.735-10.027 |
| **Sex** (Male vs Female) | 0.000 | 1.000 | 0.000 | 0.287 | 0.267 | 0.032-2.594 | 0.431 | 0.453 | 0.048-3.885 |
| **TLC** (<50x10^9^/L vs >50x10^9^/L) | 0.652 | 0.591 | 0.136-3.112 | 1.534 | 0.449 | 0.507-4.642 | 2.579 | 0.143 | 0.725-9.171 |
| **NCI risk** (Standard vs high) | 1.316 | 0.811 | 0.139-12.49 | 2.178 | 0.391 | 0.368-12.895 | 1.684 | 0.603 | 0.236-12.031 |
| **Immunophenotype** | | | | | | | | | |
| Immature | REF |  |  | REF |  |  | REF |  |  |
| Cortical | 1.599 | 0.572 | 0.315-8.125 | 0.238 | 0.034 | 0.063-0.896 | 0.233 | 0.051 | 0.054-1.009 |
| Mature | 0.639 | 0.610 | 0.114-3.573 | 0.245 | **0.045** | **0.062-0.972** | **0.186** | **0.047** | **0.035-0.975** |
| **Prednisolone response**  (Sensitive vs resistant) | 0.454 | 0.338 | 0.090-2.285 | **0.223** | **0.033** | **0.056-0.883** | 0.251 | 0.102 | 0.048-1.314 |
| **MRD** (Negative vs Positive) | **0.179** | **0.035** | **0.036-0.883** | **0.224** | **0.030** | **0.058-0.862** | 0.319 | 0.152 | 0.067-1.522 |
| ***HHEX*** (Low vs  High) | **9.258** | **0.012** | **1.628-52.65** | **8.632** | **0.011** | **1.637-45.526** | **7.564** | **0.017** | **1.434-39.905** |

**Supplementary Table 9: Multivariate Cox regression analysis for prognostic association of *LYL1* expression in T-ALL patients in the validation cohort**

| **Variables** | **Overall Survival** | | | **Event free survival** | | | **Relapse free survival** | | |
| --- | --- | --- | --- | --- | --- | --- | --- | --- | --- |
|  | **HR** | **P value** | **95% CI** | **HR** | **P value** | **95% CI** | **HR** | **P value** | **95% CI** |
| **Age** (Pediatrics vs Adults) | 0.419 | 0.442 | 0.046-3.848 | **3.088** | **0.042** | **1.041-9.162** | **4.045** | **0.036** | **1.095-14.936** |
| **Sex** (Male vs Female) | 0.000 | 1.000 | 0.000 | 0.400 | 0.409 | 0.045-3.524 | 0.493 | 0.539 | 0.052-4.704 |
| **TLC** (<50x10^9^/L vs >50x10^9^/L) | 0.460 | 0.309 | 0.103-2.055 | 1.210 | 0.751 | 0.373-3.922 | 1.558 | 0.503 | 0.426-5.700 |
| **NCI risk** (Standard vs high) | 2.399 | 0.455 | 0.242-23.77 | 1.132 | 0.883 | 0.217-5.904 | 0.803 | 0.806 | 0.139-4.642 |
| **Immunophenotype** | | | | | | | | | |
| Immature | REF |  |  | REF |  |  | REF |  |  |
| Cortical | 2.346 | 0.354 | 0.386-14.258 | 0.365 | 0.123 | 0.101-1.314 | 0.299 | 0.121 | 0.065-1.374 |
| Mature | 0.592 | 0.572 | 0.096-3.652 | 0.494 | 0.276 | 0.139-1.758 | 0.421 | 0.266 | 0.092-1.932 |
| **Prednisolone response**  (Sensitive vs resistant) | 0.535 | 0.448 | 0.107-2.688 | **0.248** | **0.039** | **0.066-0.929** | 0.212 | 0.056 | 0.043-1.040 |
| **MRD** (Negative vs Positive) | 0.501 | 0.438 | 0.088-2.871 | 0.270 | 0.064 | 0.068-1.080 | 0.439 | 0.311 | 0.090-2.156 |
| ***LYL1*** (Low vs  High) | **6.669** | **0.010** | **1.562-28.46** | **3.517** | **0.014** | **1.285-9.627** | **6.354** | **0.002** | **1.987-20.321** |

**Supplementary Table 10: Multivariate Cox regression analysis for prognostic association of *ST20* expression in T-ALL patients in the validation cohort**

| **Variables** | **Overall Survival** | | | **Event free survival** | | | **Relapse free survival** | | |
| --- | --- | --- | --- | --- | --- | --- | --- | --- | --- |
|  | **HR** | **P value** | **95% CI** | **HR** | **P value** | **95% CI** | **HR** | **P value** | **95% CI** |
| **Age** (Pediatrics vs Adults) | 1.888 | 0.584 | 0.194-18.37 | **8.041** | **0.001** | **2.289-28.24** | **8.783** | **0.002** | **2.150-35.888** |
| **Sex** (Male vs Female) | 0.000 | 1.000 | 0.000 | 0.313 | 0.306 | 0.034-2.896 | 0.416 | 0.445 | 0.044-3.956 |
| **TLC** (<50x10^9^/L vs >50x10^9^/L) | 0.341 | 0.207 | 0.064-1.813 | 0.391 | 0.179 | 0.099-1.538 | 0.699 | 0.647 | 0.150-3.244 |
| **NCI risk** (Standard vs high) | 6.077 | 0.140 | 0.555-66.56 | 4.598 | 0.105 | 0.726-29.14 | 3.627 | 0.231 | 0.440-29.915 |
| **Immunophenotype** | | | | | | | | | |
| Immature | REF |  |  | REF |  |  | REF |  |  |
| Cortical | 1.854 | 0.518 | 0.286-12.03 | 0.765 | 0.706 | 0.190-3.078 | 0.664 | 0.617 | 0.134-3.299 |
| Mature | 0.675 | 0.721 | 0.078-5.830 | 0.376 | 0.194 | 0.086-1.643 | 0.309 | 0.186 | 0.054-1.761 |
| **Prednisolone response**  (Sensitive vs resistant) | 0.639 | 0.604 | 0.118-3.470 | 0.352 | 0.104 | 0.100-1.241 | 0.364 | 0.191 | 0.080-1.654 |
| **MRD** (Negative vs Positive) | 0.501 | 0.438 | 0.088-2.871 | 0.270 | 0.064 | 0.068-1.080 | 0.439 | 0.311 | 0.090-2.156 |
| ***ST20*** (Low vs  High) | **0.083** | **0.001** | **0.018-0.379** | **0.087** | **0.001** | **0.021-0.352** | **0.101** | **0.005** | **0.020-0.507** |

**Supplementary Table 11: Multivariate Cox regression analysis for prognostic association of *RAG1* expression in T-ALL patients in the validation cohort**

| **Variables** | **Overall Survival** | | | **Event free survival** | | | **Relapse free survival** | | |
| --- | --- | --- | --- | --- | --- | --- | --- | --- | --- |
|  | **HR** | **P value** | **95% CI** | **HR** | **P value** | **95% CI** | **HR** | **P value** | **95% CI** |
| **Age** (Pediatrics vs Adults) | 0.514 | 0.554 | 0.057-4.648 | 2.599 | 0.109 | 0.808-8.365 | 2.901 | 0.121 | 0.754-11.15 |
| **Sex** (Male vs Female) | 0.000 | 1.000 | 0.000 | 0.552 | 0.612 | 0.055-5.514 | 0.805 | 0.860 | 0.073-8.900 |
| **TLC** (<50x10^9^/L vs >50x10^9^/L) | 0.461 | 0.338 | 0.095-2.247 | 0.741 | 0.645 | 0.207-2.649 | 1.176 | 0.818 | 0.294-4.698 |
| **NCI risk** (Standard vs high) | 5.257 | 0.167 | 0.498-55.47 | 3.205 | 0.212 | 0.515-19.96 | 2.373 | 0.398 | 0.320-17.57 |
| **Immunophenotype** | | | | | | | | | |
| Immature | REF |  |  | REF |  |  | REF |  |  |
| Cortical | 1.078 | 0.932 | 0.190-6.124 | 0.373 | 0.173 | 0.090-1.543 | 0.267 | 0.113 | 0.052-1.365 |
| Mature | 0.632 | 0.636 | 0.094-4.232 | 0.420 | 0.201 | 0.111-1.588 | 0.312 | 0.151 | 0.064-1.530 |
| **Prednisolone response**  (Sensitive vs resistant) | 0.347 | 0.198 | 0.069-1.743 | **0.220** | **0.021** | **0.061-0.792** | **0.197** | **0.036** | **0.043-0.901** |
| **MRD** (Negative vs Positive) | 0.476 | 0.363 | 0.096-2.353 | 0.311 | 0.086 | 0.082-1.179 | 0.457 | 0.324 | 0.096-2.170 |
| ***RAG1*** (Low vs  High) | **0.204** | **0.021** | **0.053-0.790** | **0.264** | **0.017** | **0.089-0.788** | **0.207** | **0.014** | **0.059-0.729** |

**Supplementary Table 12: Multivariate Cox regression analysis for prognostic association of *LMO2* expression in T-ALL patients in the validation cohort**

| **Variables** | **Overall Survival** | | | **Event free survival** | | | **Relapse free survival** | | |
| --- | --- | --- | --- | --- | --- | --- | --- | --- | --- |
|  | **HR** | **P value** | **95% CI** | **HR** | **P value** | **95% CI** | **HR** | **P value** | **95% CI** |
| **Age** (Pediatrics vs Adults) | 1.203 | 0.868 | 0.135-10.683 | 2.941 | 0.071 | 0.912-9.487 | 3.144 | 0.100 | 0.802-12.324 |
| **Sex** (Male vs Female) | 0.000 | 1.000 | 0.000 | 0.319 | 0.302 | 0.037-2.788 | 0.397 | 0.419 | 0.042-3.731 |
| **TLC** (<50x10^9^/L vs >50x10^9^/L) | 0.680 | 0.664 | 0.120-3.858 | 0.690 | 0.599 | 0.173-2.748 | 1.015 | 0.985 | 0.218-4.729 |
| **NCI risk** (Standard vs high) | 3.521 | 0.272 | 0.372-33.30 | 2.606 | 0.287 | 0.447-15.186 | 2.528 | 0.367 | 0.337-18.981 |
| **Immunophenotype** | | | | | | | | | |
| Immature | REF |  |  | REF |  |  | REF |  |  |
| Cortical | 2.047 | 0.431 | 0.344-12.17 | 0.357 | 0.100 | 0.105-1.216 | **0.246** | **0.043** | **0.063-0.957** |
| Mature | 1.237 | 0.825 | 0.189-8.099 | 0.259 | 0.050 | 0.067-1.000 | **0.161** | **0.027** | **0.032-0.810** |
| **Prednisolone response**  (Sensitive vs resistant) | 0.338 | 0.202 | 0.064-1.790 | 0.409 | 0.150 | 0.121-1.380 | 0.501 | 0.345 | 0.119-2.105 |
| **MRD** (Negative vs Positive) | 0.322 | 0.154 | 0.068-1.530 | 0.362 | 0.128 | 0.098-1.338 | 0.477 | 0.351 | 0.101-2.261 |
| ***LMO2*** (Low vs  High) | 0.267 | 0.147 | 0.045-1.589 | **3.343** | **0.040** | **1.057-10.573** | **6.052** | **0.011** | **1.49-24.424** |

**Supplementary Table 13: Multivariate Cox regression analysis for prognostic association of *DOT1L* expression in T-ALL patients in the validation cohort**

| **Variables** | **Overall Survival** | | | **Event free survival** | | | **Relapse free survival** | | |
| --- | --- | --- | --- | --- | --- | --- | --- | --- | --- |
|  | **HR** | **P value** | **95% CI** | **HR** | **P value** | **95% CI** | **HR** | **P value** | **95% CI** |
| **Age** (Pediatrics vs Adults) | 1.033 | 0.977 | 0.115-9.281 | **5.454** | **0.006** | **1.639-18.144** | **10.163** | **0.003** | **2.223-46.470** |
| **Sex** (Male vs Female) | 0.000 | 1.000 | 0.000 | 0.249 | 0.229 | 0.026-2.399 | 0.383 | 0.409 | 0.039-3.728 |
| **TLC** (<50x10^9^/L vs >50x10^9^/L) | 0.830 | 0.846 | 0.128-5.385 | 1.890 | 0.359 | 0.485-7.360 | **5.826** | **0.037** | **1.110-30.59** |
| **NCI risk** (Standard vs high) | 3.214 | 0.301 | 0.351-29.41 | 1.379 | 0.717 | 0.242-7.847 | 0.433 | 0.419 | 0.057-3.301 |
| **Immunophenotype** | | | | | | | | | |
| Immature | REF |  |  | REF |  |  | REF |  |  |
| Cortical | 1.401 | 0.703 | 0.247-7.933 | 0.532 | 0.326 | 0.151-1.874 | 0.604 | 0.487 | 0.146-2.499 |
| Mature | 0.251 | 0.244 | 0.024-2.575 | 0.280 | 0.082 | 0.066-1.178 | 0.379 | 0.206 | 0.084-1.704 |
| **Prednisolone response**  (Sensitive vs resistant) | 0.206 | 0.168 | 0.022-1.949 | 0.180 | 0.031 | 0.038-0.858 | 0.351 | 0.214 | 0.067-1.834 |
| **MRD** (Negative vs Positive) | 0.301 | 0.149 | 0.059-1.537 | **0.145** | **0.007** | **0.035-0.596** | **0.119** | **0.006** | **0.026-0.551** |
| ***DOT1L*** (Low vs  High) | 1.530 | 0.568 | 0.356-6.585 | 1.488 | 0.472 | 0.504-4.388 | **4.968** | **0.035** | **1.117-22.104** |

**Supplementary Table 14: Multivariate Cox regression analysis for prognostic association of PCAT14 expression in T-ALL patients in the validation cohort**

| **Variables** | **Overall Survival** | | | **Event free survival** | | | **Relapse free survival** | | |
| --- | --- | --- | --- | --- | --- | --- | --- | --- | --- |
|  | **HR** | **P value** | **95% CI** | **HR** | **P value** | **95% CI** | **HR** | **P value** | **95% CI** |
| **Age** (Pediatrics vs Adults) | 1.285 | 0.832 | 0.127-12.97 | **5.096** | **0.011** | **1.449-17.91** | **7.125** | **0.012** | **1.533-33.121** |
| **Sex** (Male vs Female) | 0.000 | 1.000 | 0.000 | 0.098 | 0.069 | 0.008-1.196 | 0.084 | 0.069 | 0.006-1.207 |
| **TLC** (<50x10^9^/L vs >50x10^9^/L) | 3.311 | 0.396 | 0.209-52.45 | 3.003 | 0.207 | 0.544-16.57 | 11.829 | 0.053 | 0.965-145.03 |
| **NCI risk** (Standard vs high) | 4.098 | 0.236 | 0.398-42.24 | 2.146 | 0.384 | 0.385-11.97 | 1.109 | 0.916 | 0.162-7.579 |
| **Immunophenotype** | | | | | | | | | |
| Immature | REF |  |  | REF |  |  | REF |  |  |
| Cortical | 1.609 | 0.588 | 0.288-8.995 | 0.514 | 0.322 | 0.137-1.920 | 0.441 | 0.275 | 0.102-1.918 |
| Mature | 0.106 | 0.193 | 0.004-3.122 | **0.164** | **0.041** | **0.029-0.925** | **0.079** | **0.037** | **0.007-0.854** |
| **Prednisolone response**  (Sensitive vs resistant) | 0.199 | 0.176 | 0.019-2.067 | **0.143** | **0.021** | **0.027-0.747** | 0.230 | 0.097 | 0.041-1.305 |
| **MRD** (Negative vs Positive) | 0.345 | 0.301 | 0.046-2.584 | **0.121** | **0.011** | **0.024-0.616** | **0.128** | **0.030** | **0.020-0.816** |
| ***PCAT14*** (Low vs  High) | 0.078 | 0.067 | 0.005-1.201 | 0.221 | 0.063 | 0.045-1.089 | **0.067** | **0.027** | **0.006-0.738** |
