# Supplementary material for: Decoding the genetic symphony: Profiling protein-coding and long noncoding RNA expression in T-acute lymphoblastic leukemia for clinical insights
Source: PNAS Nexus. 2024 Jan 12;3(2):pgae011. doi: 10.1093/pnasnexus/pgae011 (PMC10847906; doi:10.1093/pnasnexus/pgae011)
Supplement: pgae011_Supplementary_Data [file pgae011_supplementary_data.zip › PNASNEXUS-PNASNEXUS-2023-00530R-s02.docx]

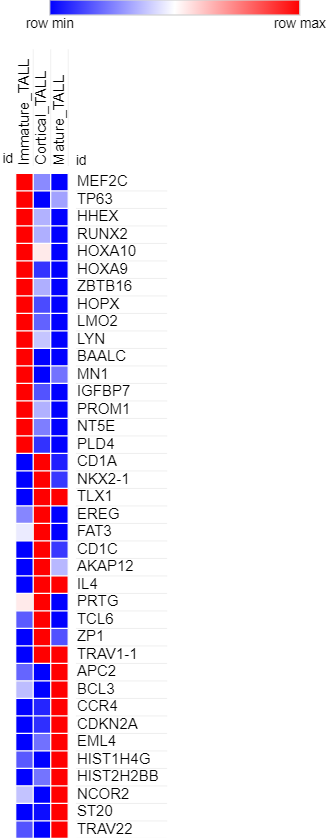

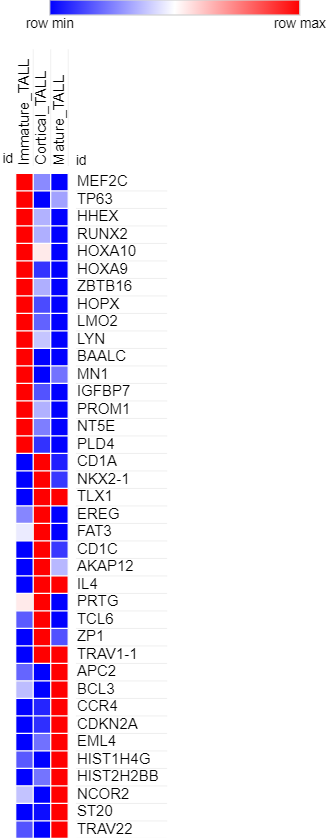

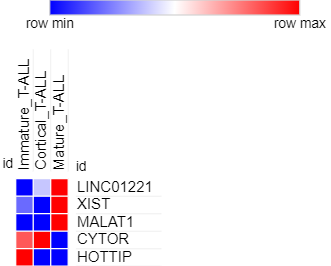


**A**

**B**


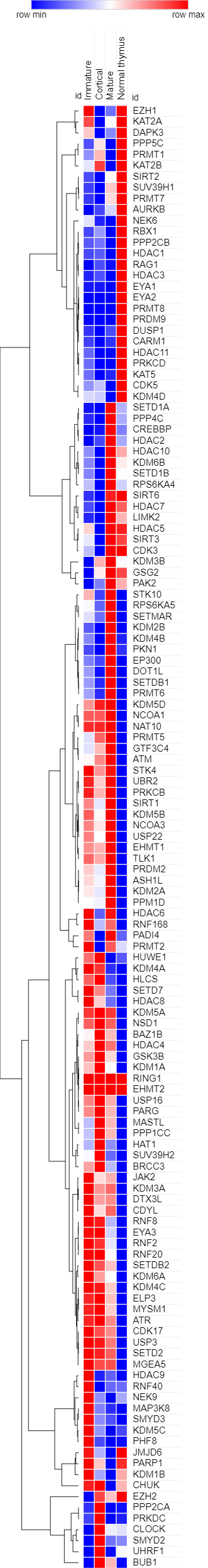


**C**

**Supplementary figure 1.** Heatmap showing selected differentially expressed **A.** Protein-coding genes **B.** Epigenetic modifiers in T-ALL subgroups as compared to normal Thymus. **C.** Long non-coding RNAs in three T-ALL subgroups: immature, cortical, mature in the discovery cohort. Gene expression values are normalized and color coded, as indicated by the scale beneath the graph. The heatmaps are plotted with the normalized log_2_ (count) values.

**
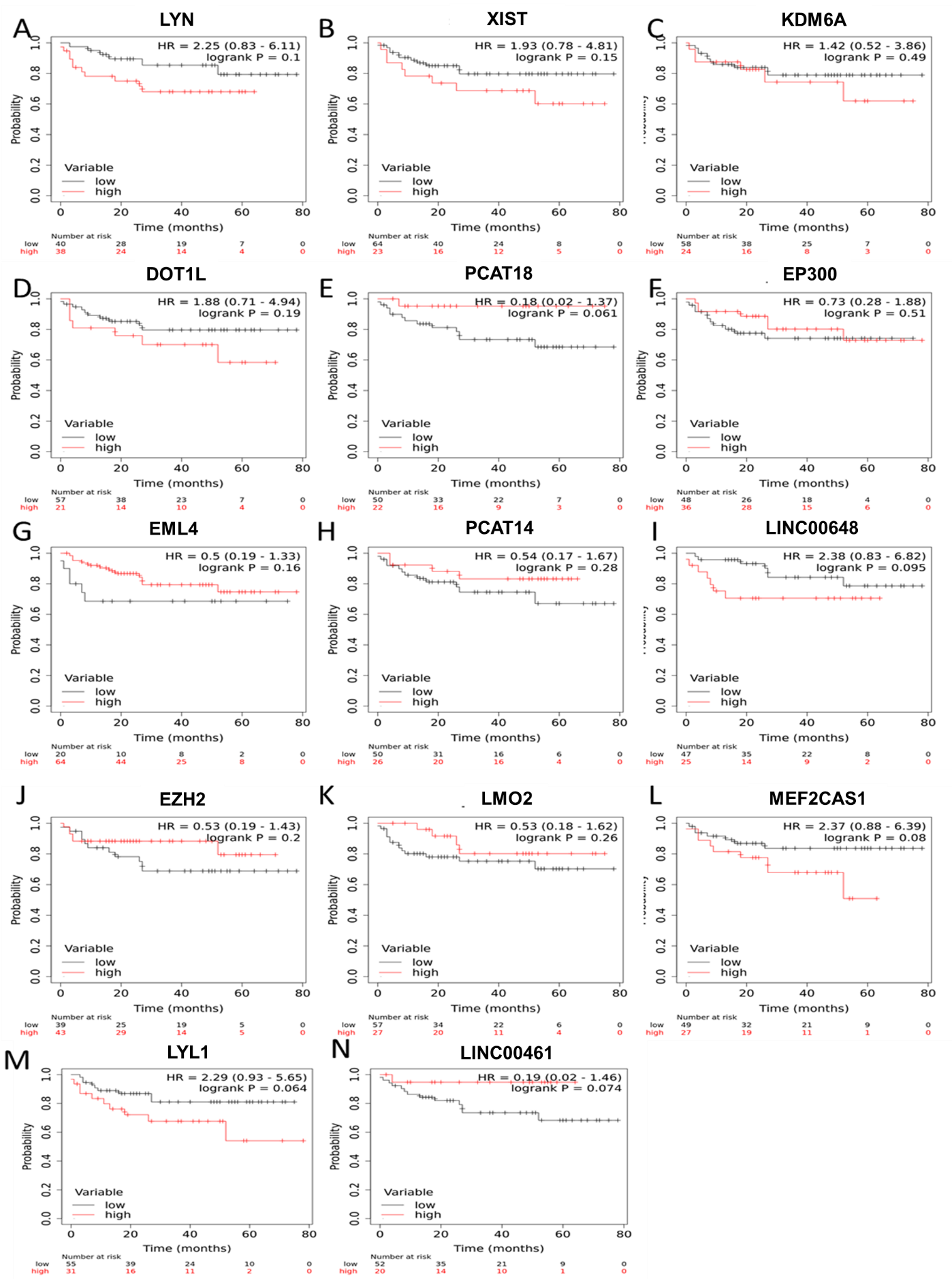
**

**Supplementary Figure 2:** Kaplan Meier analysis for overall survival for expression of **A.** *LYN*, **B.** *XIST*, **C.** *KDM6A*, **D.** *DOT1L*, **E.** *PCAT18*, **F.** *EP300*, **G.** *EML4*, **H.** *PCAT14*, **I.** *LINC00648*, **J.** *EZH2*, **K.** *LMO2*, **L.** *MEF2CAS1*, **M.** *LYL1*, **N.** *LINC00461* in T-ALL patients in the validation cohort.


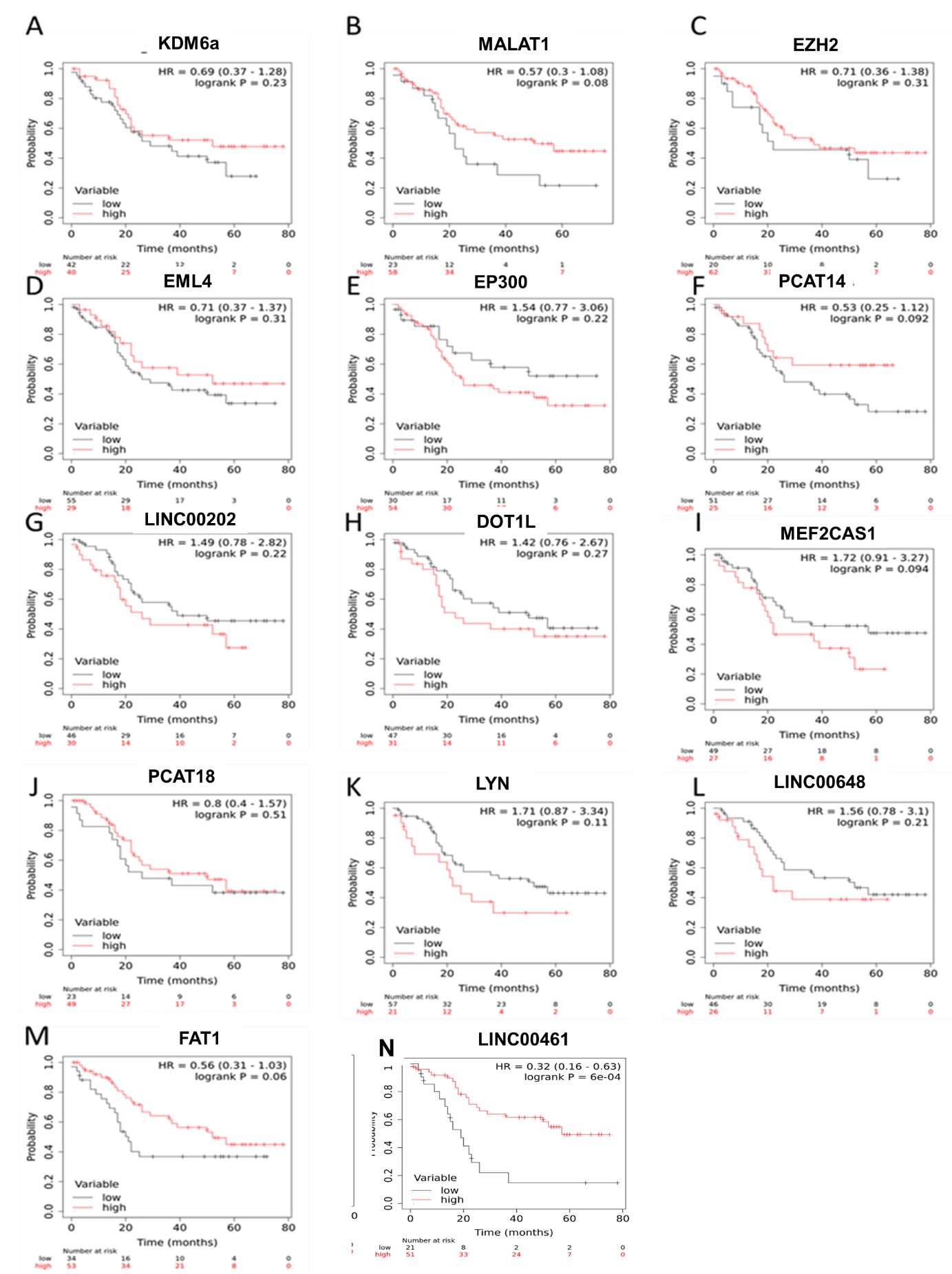


**Supplementary figure 3:** Kaplan Meier analysis for Event free survival for expression of **A.** *KDM6A*, **B.** *MALAT1*, **C.** *EZH2*, **D.** *EML4*, **E.** *EP300*, **F.** *PCAT14,* **G.** *LINC00202,* **H.** *DOTIL1,* **I.** *MEF2CAS1,* **J.** *PCAT18,* **K.** *LYN,* **L.** *LINC00648,* **M.** *FAT1* and **N.** *LINC00461* in T-ALL patients in the validation cohort.


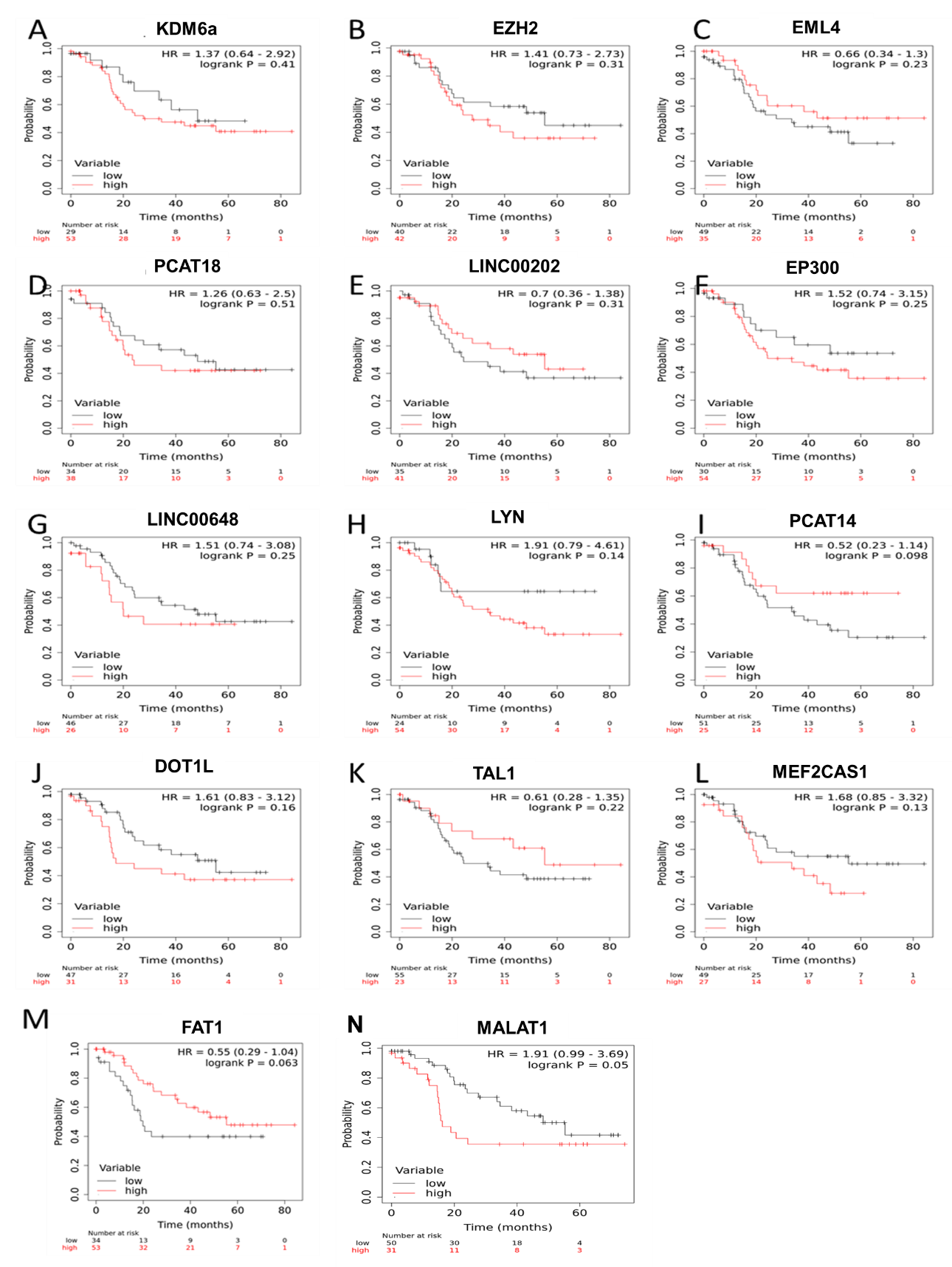


**Supplementary figure 4:** Kaplan Meier analysis for relapse free survival for expression of **A.** *KDM6A*, **B.** *EZH2*, **C.** *EML4*, **D.** *PCAT18*, **E.** *LINC00202*, **F.** *EP300,* **G.** *LINC00648,* **H.** *LYN,* **I.** *PCAT14,* **J.** *DOT1L,* **K.** *TAL1,* **L.** *MEF2CAS1,* **M.** *FAT1* and **N*.*** *MALAT1* in T-ALL patients in the validation cohort.
